# Mechanistically Guided Design of an Efficient and Enantioselective Aminocatalytic α-Chlorination of Aldehydes

**DOI:** 10.1021/jacs.1c02997

**Published:** 2021-04-30

**Authors:** George Hutchinson, Carla Alamillo-Ferrer, Jordi Burés

**Affiliations:** Department of Chemistry, The University of Manchester, Oxford Road, M13 9PL Manchester, U.K.

## Abstract

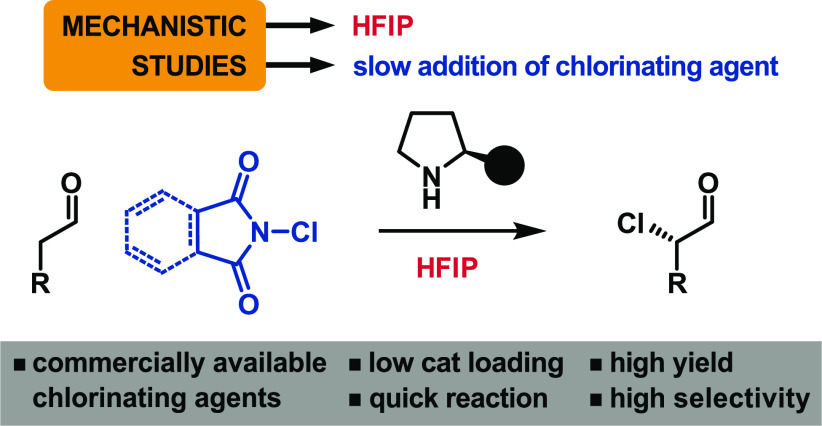

The enantioselective aminocatalytic α-chlorination of aldehydes is a challenging reaction because of its tendency to proceed through neutral intermediates in unselective pathways. Herein we report the rational shift to a highly selective reaction pathway involving charged intermediates using hexafluoroisopropanol as solvent. This change in mechanism has enabled us to match and improve upon the yields and enantioselectivities displayed by previous methods while using cheaper aminocatalysts and chlorinating agents, 80–95% less amount of catalyst, convenient temperatures, and shorter reaction times.

Over the past 20 years, many different enantioselective aminocatalytic reactions have been developed to functionalize carbonyl compounds.^1−3^ This field has expanded rapidly because of its relevance and operational simplicity, but emphasis has been placed on the discovery of diverse reactions over their optimization. Consequently, the currently available aminocatalytic reactions often require atypical reagents, high catalyst loadings, low temperatures, and long reaction times. For example, the asymmetric α-chlorination of aldehydes requires nonideal reaction conditions to avoid several pathways that erode the intrinsic stereoselectivity of the aminocatalyst and reduce its turnover frequency.^4−7^

Mechanistic studies of aminocatalytic reactions have shown the existence of unexpected reaction pathways involving neutral diastereotopic downstream intermediates.^4−7^ Investigation into the α-chlorination reaction demonstrated that although the facial selectivity of the enamine in the chlorination step is almost perfect (Scheme 1), the final enantioselectivity of the product is low due to a posterior bifurcation of the reaction pathway. Catalytic intermediate **1** is involved in fast equilibria with the diastereoisomeric 1,2-aminal adducts *syn***-2** and *anti***-2** (Scheme 1).^5^ Under standard reaction conditions, the 1,2-aminal adducts are the resting state of the reaction because of their higher stability with respect to the charged iminium salt (**1** in Scheme 1). As a consequence, the reaction proceeds through the pathways involving 1,2-aminals (red pathways in Scheme 1), which are intrinsically slower than the one through the direct hydrolysis of the iminium ion (blue pathway in Scheme 1). In addition, the stereospecific elimination of the diastereoisomeric 1,2-aminals leads to diastereomeric enamines, *Z***-3** and *E***-3**, which ultimately form products with opposite stereochemistry at the α-center to the carbonyl (*R***-4** and *S***-4**). Pioneering enantioselective aminocatalytic chlorinating methods solved this problem by using aminocatalysts that favor one of the diastereomeric 1,2-aminals,^8^ using chlorinating agents that result in poorly coordinating counterions,^9,10^ using SOMO catalysis (single occupied molecular orbital),^11^ or using a combination of a very sterically hindered catalyst, *N*-chloro-4-nitrophthalimide and a mixture of trifluoroacetic and acetic acid.^12^ All these solutions led to highly enantio-enriched products, but they require expensive or noncommercially available aminocatalysts and chlorinating agents, high catalyst loadings, and, in some cases, low temperatures (−30 °C) and very long reaction times (48 h).^13^ Herein we report the use of hexafluoroisopropanol (HFIP) to invert the standard stability of aminocatalytic intermediates in organic solvents, which enables the efficient and enantioselective aminocatalytic α-chlorination of aldehydes.

**Scheme 1:**
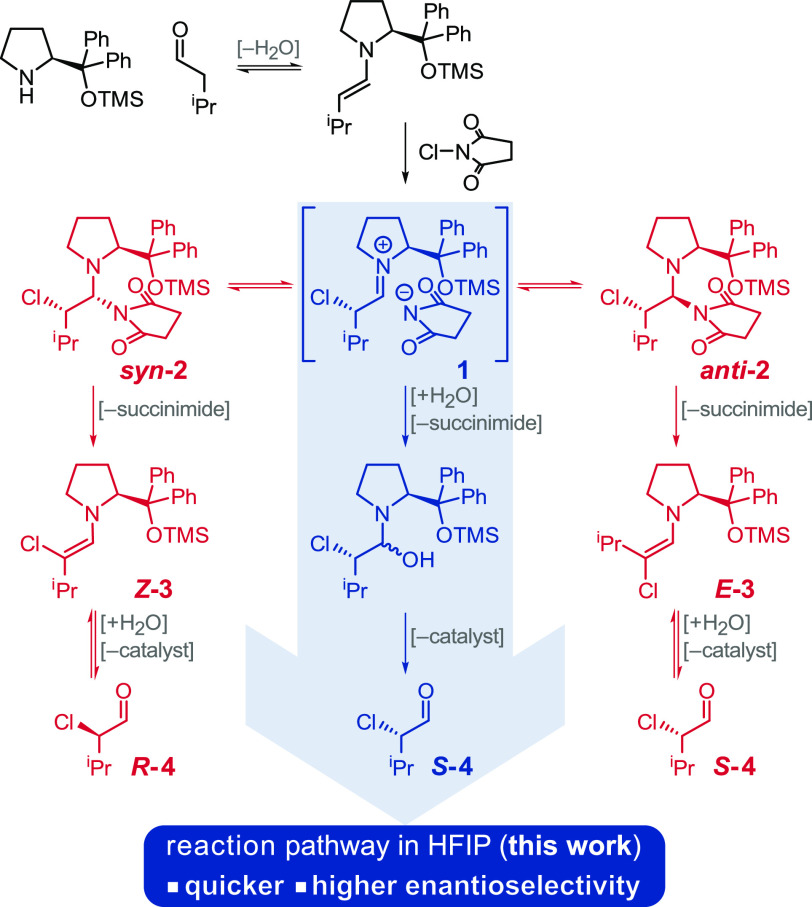
Key Steps That Determine the Enantioselectivity of the Aminocatalytic α-Chlorination of Aldehydes

Hexafluoroisopropanol is well-known to stabilize cations because of its high dielectric constant and low nucleophilicity.^14^ Therefore, we envisioned that HFIP could stabilize iminium ions with respect to neutral 1,2-aminal downstream intermediates. To test this hypothesis, we mixed hydrocinnamaldehyde with the Jørgensen–Hayashi type catalyst **3a** in HFIP (Scheme 2). We observed, by ^1^H NMR spectroscopy, quantitative conversion of the catalyst to the iminium ion of the hydrocinnamaldehyde.^13^ The remarkable preference for the iminium ion contrasts with the exclusive formation of the corresponding enamine that we observed in all the other solvents we tried: CD_2_Cl_2_, CDCl_3_, CD_3_CN, THF-*d*_8_, methyl *tert*-butyl ether (MTBE), toluene-*d*_8_, DMSO-*d*_6_, CD_3_OD, and even isopropanol.^13^

**Scheme 2:**
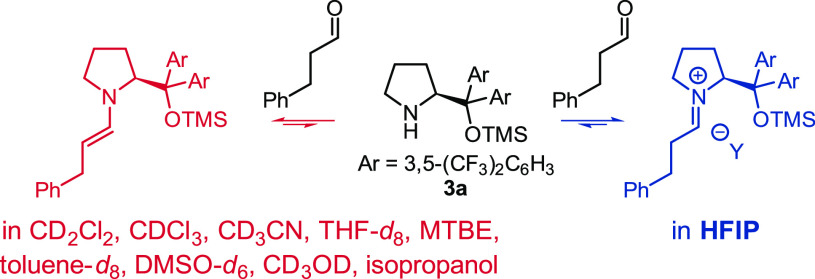
Enamine and Iminium Ion Stability in Different Organic Solvents

Encouraged by the capacity of HFIP to stabilize iminium ions, we attempted the α-chlorination reaction in HFIP. We initially obtained a disappointing 2.4% of the monochlorinated product and 5.9% of dichlorinated product (71% of dichlorination) in 12 h when using *N*-chlorosuccinimide (NCS), 2.5 equiv of hydrocinnamaldehyde, and 2 mol % of catalyst **3a** (Figure 1). To understand the reasons for this discouraging result and to improve the reaction yield, we monitored the reaction by ^1^H NMR spectroscopy. The rate of formation of dichlorinated product was not proportional to the concentration of monochlorinated product (Figure 1a), which suggested that the dichlorinated product was mainly generated from the overchlorination of a catalytic intermediate instead of the subsequent chlorination of the released monochlorinated product. We reasoned that the addition of water should reduce the percentage of dichlorination because water is involved in the hydrolysis of the chlorinated iminium but not in its equilibration with the chlorinated enamine and subsequent dichlorination reaction. When we ran the reaction with 11.15 M of water, the percentage of dichlorinated product satisfactorily decreased from 71% to 3% (Figure 1b). While the addition of water solved the dichlorination problem, it further reduced the overall yield to 3%. We attributed this even lower yield to deactivation processes involving the free catalyst because we observed that water shifted the iminium formation equilibrium toward the free catalyst.^13^

**Figure 1:**
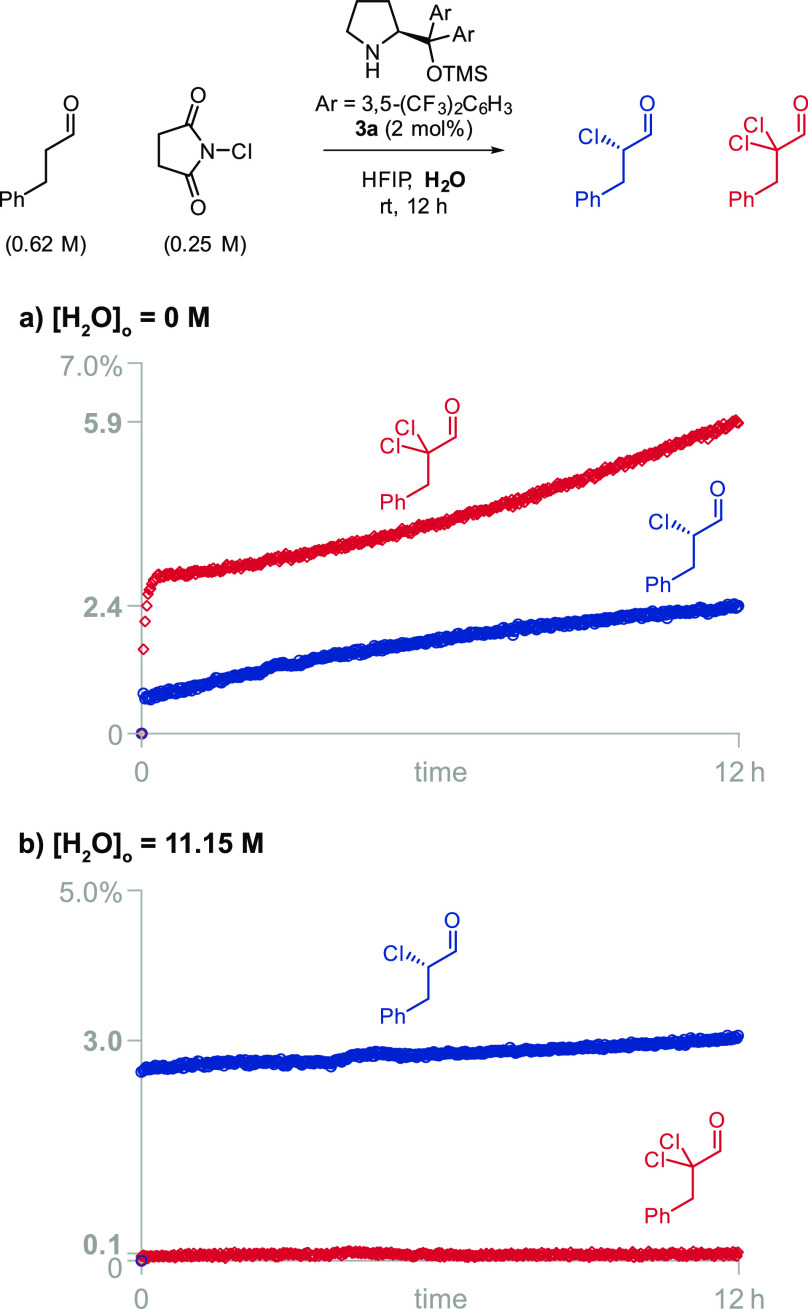
Addition of water reduces the amount of dichlorinated product, but it also decreases the overall yield after 12 h.

To better understand the deactivation processes, we focused our attention on the ^1^H NMR signals of the catalytic species observed during the reaction. The spectroscopic data acquired during the reaction showed a quick and near-quantitative formation of a catalytic intermediate without signals corresponding to the aldehyde chain. Consequently, we investigated the potential deactivation pathways arising from reaction of the free catalyst with the chlorinating agent. We monitored, by ^1^H NMR spectroscopy, the reaction of four different Jørgensen–Hayashi type catalysts (**3a**–**3d**, Scheme 3) with NCS in HFIP. We observed immediate chlorination of all the catalysts, which led to a Grob-type fragmentation^15,16^ in the case of the catalysts bearing two phenyl groups, **3c** and **3d** (Scheme 3). We hypothesize that the 3,5-bis(trifluoromethyl)phenyl groups in catalysts **3a** and **3b** disfavor the required conformation for a Grob-type fragmentation, and as a consequence, the corresponding chlorinated catalysts are stable for more than 16 h.^13^ To the best of our knowledge, this is the first Grob-type fragmentation described for Jørgensen–Hayashi type catalysts and may also be relevant for other aminocatalytic reactions.

**Scheme 3:**
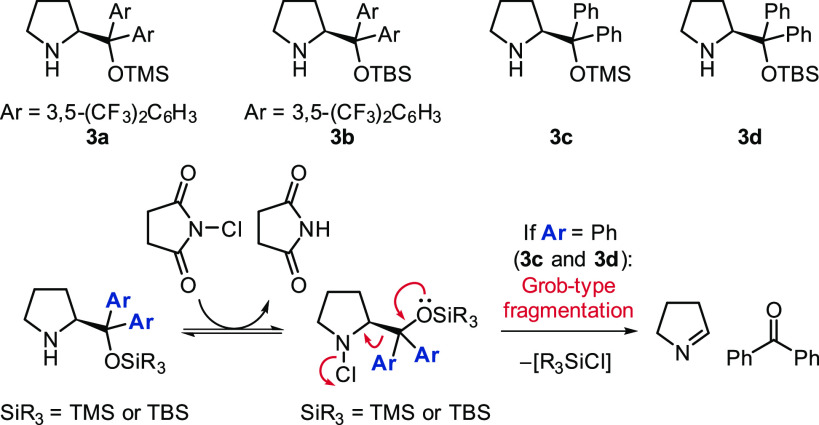
Reversible and Irreversible Deactivation Processes of Jørgensen–Hayashi Type Catalysts

As we found that the chlorination of the catalyst is reversible,^13^ we tried to shift the chlorination equilibrium toward the active catalyst by adding succinimide to the reaction. Unfortunately, even running the reaction in HFIP saturated with succinimide was not enough to accelerate the reaction sufficiently (20% yield in 10 h with 0.3 M of succinimide added).^13^

Finally, we attempted to mitigate the catalyst deactivation by dosing the NCS slowly. This strategy was previously used by Hein, Armstrong, and Blackmond to minimize the reversible reaction of prolinate salts with diethyl azodicarboxylate.^17^ On the basis of our newfound understanding of the reaction in HFIP, we expected low concentrations of NCS to disfavor the formation of inactive chlorinated catalyst and to reduce the percentage of dichlorination. To monitor the progress of the reaction during the slow addition of chlorinating agent, we used an *in situ* FT-IR probe, which allowed us to continuously measure the concentration of NCS and succinimide. The instantaneous addition of NCS to a reaction containing 2.7 M of water (Figure 2a) led to the quick chlorination of the catalyst and the stagnation of the reaction at 9% yield with 14% of the product being dichlorinated. Longer addition times (4 min, Figure 2b) led to a gradual accumulation of NCS, which resulted in a 52% yield with 2% of dichlorination. When the NCS was added sufficiently slowly (19 min, Figure 2c), the generation of succinimide perfectly matched the addition of NCS; the reaction was complete in just 19 min, and only 1% of the product was dichlorinated. *By dosing NCS at the adequate rate, we increased the yield of the reaction from 9% to 100% and decreased the percentage of dichlorination from 14% to 1%*.^13^

**Figure 2:**
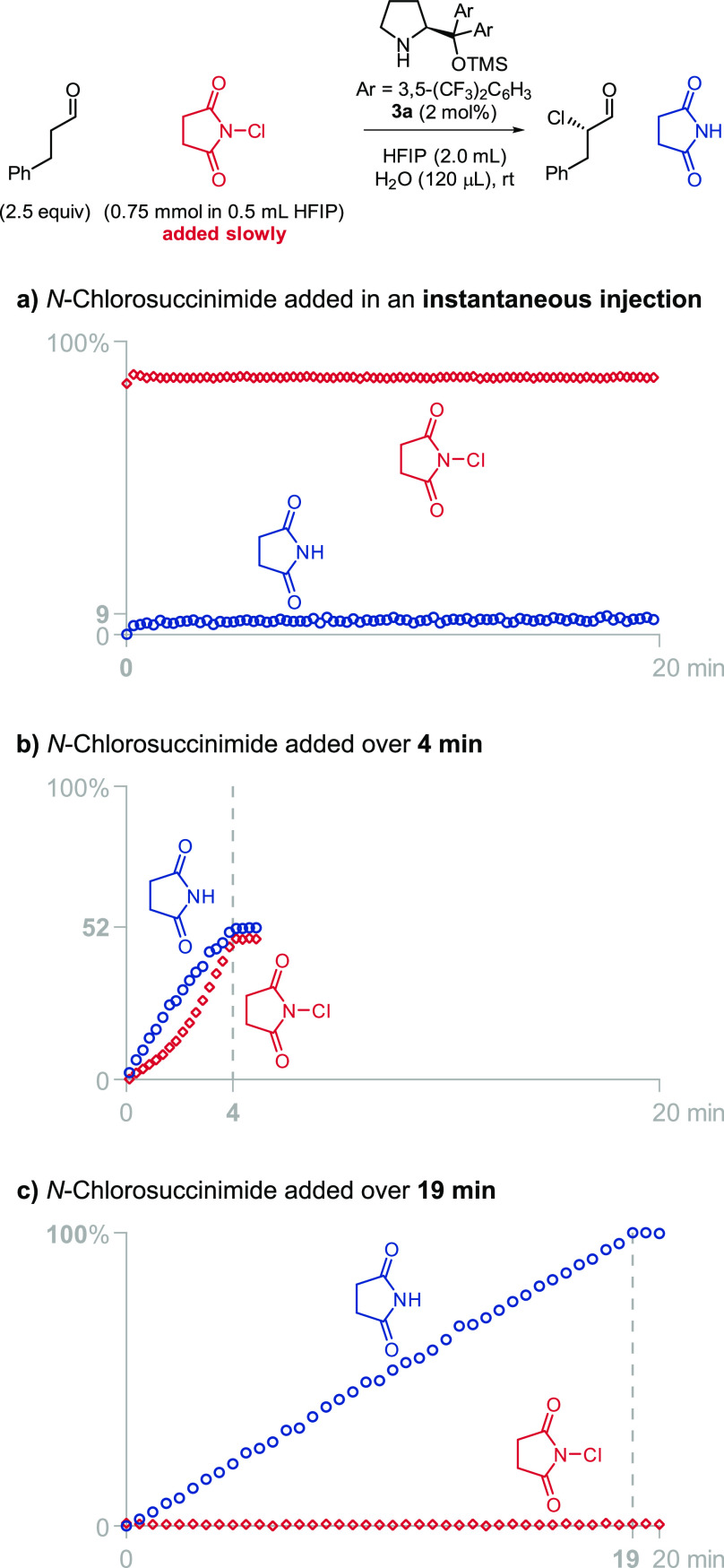
Slow addition of the chlorinating agent enables the completion of the reaction.

We identified the concentration of water and rate of addition of the chlorinating agent as key parameters to control the overall yield of the reaction, the enantiomeric ratio of the product, and the ratio of mono- and dichlorinated aldehyde.^7^ Higher concentrations of water decreased the percentage of dichlorinated product and increased the chlorination of the catalyst.^13^ Lower rates of addition reduced the percentage of dichlorinated product and the chlorination of the catalyst, but too-low rates allowed the racemization of the product by the free catalyst.^13^ We also observed that higher percentages of dichlorination usually correlated to slight increases in the final enantiomeric ratio of the product, probably because of a kinetic resolution analogous to the one described by Jørgensen in the α,α-difluorination of aldehydes.^18^

The mechanistic understanding acquired during this study enabled us to demonstrate excellent results under several practical reaction conditions (Table 1). The amount of water and rate of addition of the chlorinating agent were quickly tuned for each set of reaction conditions following a standard procedure detailed in the Supporting Information.^13^ For the hydrocinnamaldehyde, we obtained excellent yields and enantioselectivities in just 60 min, at 0 °C, using only 2 mol % of catalyst **3b** and standard *N*-chlorophthalimide (NCP) as chlorinating agent (Table 1, entry 1). We also attained exceptional results using NCS as the chlorinating agent (Table 1, entry 2). The slightly smaller enantioselectivity obtained with NCS may be due to the partial competition of the less selective reaction pathway, which was shown to be quicker when using NCS instead of NCP.^5^ Additionally, we achieved similar enantioselectivities with the typically less selective catalyst **3a** by sacrificing some yield (Table 1, entry 3). We were able to produce comparable results despite reducing the amount of catalyst by half, to 1 mol %, through longer addition times of the chlorinating agent and the addition of phthalimide at the beginning of the reaction to reduce catalyst chlorination (Table 1, entry 4). We achieved excellent results running the reaction at room temperature, conditions under which the reaction is complete in just 20 min (Table 1, entry 5). We obtained remarkable results even using hydrocinnamaldehyde as limiting reagent, by adding the chlorinating agent over 150 min (Table 1, entry 6). The longer addition time (150 min) is required to avoid catalyst deactivation due to the higher percentage of free catalyst present at lower concentrations of aldehyde. To show the ease of tuning the amount of water and rate of addition of chlorinating agent for new substrates, we also chlorinated dodecanal (Table 1, entry 7), octanal (Table 1, entry 8), pentanal (Table 1, entry 9), propanal (Table 1, entry 10), isovaleraldehyde (Table 1, entry 11), and 5-bromopentanal (Table 1, entry 12) with excellent yields and enantioselectivities. We were even able to chlorinate with good yield and exquisite selectivity the δ-valerolactol (Table 1, entry 13), a poorly reactive substrate due to the predominance of its hemiacetal form.

**Table 1:**
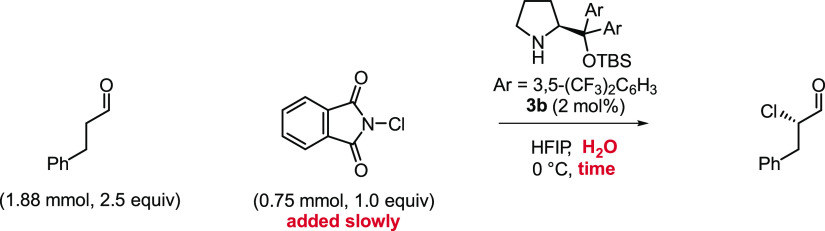
Example of Optimized Reaction Conditions for the α-Chlorination of Aldehydes

entry	deviation from above	H_2_O (μL)	time of addition of the chlorinating agent (min)	yield^a^ (%)	er^b^
1	none	35	60	85	99:1
2	**NCS** instead of NCP	35	50	84	97:3
3	**catalyst3a** instead of **3b**	10	60	70	98:2
4^c^	**1mol %** of **3b** instead of 2 mol %	35	150	85	98:2
5	**rt** instead of 0 °C	30	20	91	97:3
6^d^	**0.76 mmol of hydrocinnamaldehyde** instead of 1.88 mmol	40	150	68	97:3
7	**dodecanal** instead of hydrocinnamaldehyde	75	60	77	99:1
8	**octanal** instead of hydrocinnamaldehyde	65	60	76	99:1
9	**pentanal** instead of hydrocinnamaldehyde	70	75	66	99:1
10	**propanal** instead of hydrocinnamaldehyde	100	75	78	98:2
11^e^	**isovaleraldehyde** instead of hydrocinnamaldehyde	48	25	80	99:1
12	**5-bromopentanal** instead of hydrocinnamaldehyde	70	75	80	98:2
13^f^	**δ-valerolactol** instead of hydrocinnamaldehyde	20	1440	68	99:1

a Yield of α-chloroaldehyde measured by standard addition with the ReactIR 15.^19^

b Enantiomeric ratio determined after reduction of the α-chloroaldehyde to the α-chloroalcohol.

c 0.76 mmol of phthalimide added at the beginning of the reaction.

d 0.90 mmol (1.2 equiv) of NCP infused over the time of addition.

e Reaction run at room temperature and 80% of the scale.

f 5 mol % of catalyst **3b** at 8 °C and yield measured by qNMR with an internal standard.

In conclusion, hexafluoroisopropanol switches the natural reaction pathway of the α-aminocatalytic chlorination reaction in organic solvents by stabilizing charged catalytic intermediates. Originally, this change in mechanism engendered enantio-enriched products at the cost of high levels of dichlorination and catalyst deactivation. Both these complications have been mitigated by tuning the amount of water and the rate of addition of the chlorinating agent. The resulting synthetic methodology achieves better overall yields and enantioselectivities than current methods while using more convenient catalysts and cheaper chlorinating agents in shorter reaction times and at milder temperatures.

## Supporting Information Available

Experimental procedures, spectral and chromatographic, data, summary of relevant previous aminocatalytic methods, and kinetic data (PDF)
