# Supplementary material for: Mechanistically Guided Design of an Efficient and Enantioselective Aminocatalytic α-Chlorination of Aldehydes
Source: J Am Chem Soc. 2021 Apr 30;143(18):6805–9. doi: 10.1021/jacs.1c02997 (PMC8297727; doi:10.1021/jacs.1c02997)
Supplement: Supplementary file 1 — ja1c02997_si_001.pdf [file ja1c02997_si_001.pdf]

Supporting Information

Mechanistically Guided Design of an Efficient and Enantioselective Aminocatalytic α-Chlorination of Aldehydes

George Hutchinson, Carla Alamillo-Ferrer and Jordi Burés*

The University of Manchester, Department of Chemistry
Oxford Road, Manchester, M13 9PL, UK
E-mail: jordi.bures@manchester.ac.uk

Table of Contents

1. General information S-3

2. Previous studies on the mechanism of the aminocatalytic α-chlorination of aldehydes S-4

3. Previous methodologies for the aminocatalytic α-chlorination of aldehydes S-5

3.1. MacMillan et al. J. Am. Chem. Soc. 2004, 126, 13, 4108–4109 S-6

3.2. Jørgensen et al. J. Am. Chem. Soc. 2004, 126, 15, 4790–4791 S-6

3.3. MacMillan et al. Angew. Chem., Int. Ed. 2009, 48, 5121–5124 S-7

3.4. Renaud et al. J. Org. Chem. 2016, 81, 1251–1255 S-7

3.5. Christmann et al. Angew. Chem., Int. Ed. 2018, 57, 11683–11687 S-8

4. Study of the stability of enamines and iminium ions S-9

4.1. Spectroscopic description of the enamine in standard organic solvents S-10

4.2. Spectroscopic description of the iminium ion in HFIP S-12

4.3. Example of enamine characterization: CD_2Cl_2 S-13

4.4. Iminium ion characterization: HFIP S-15

5. Aminocatalytic α-chlorination of aldehydes in HFIP monitored by ^1H NMR S-18

6. Effect of water on the percentage of dichlorinated product S-19

6.1. Reaction with $[H_2O]_0$ = 2.22 M S-19

6.2. Reaction with $[H_2O]_0$ = 11.15 M S-20

6.3. Study of the effect of water on the equilibrium of iminium ion formation S-21

7. Study of the deactivation of the aminocatalyst by NCS S-22

7.1. Reaction of catalyst **3c** with NCS in HFIP S-25

7.2. Study of the reversibility of the chlorination reaction of the aminocatalyst S-27

8. Aminocatalytic α-chlorination with slow addition of the chlorinating agent S-29

8.1. NCS added in an instantaneous injection S-30

8.2. Rate of addition of NCS = 0.1875 mmol/min S-31

8.3. Rate of addition of NCS = 0.0395 mmol/min S-32

8.4. Simulated kinetic reaction profile with slow addition of NCS S-33

9. General method to determine the optimal rate of addition of chlorinating agent and the amount of added water S-36

10. Showcase aminocatalytic α-chlorination of aldehyde reactions in HFIP S-38

10.1. Standard reaction conditions S-39

10.2. Reaction with NCS as the chlorinating reagent S-41

10.3. With catalyst 3a S-43

10.4. Reaction with 1 mol% catalyst loading S-45

10.5. Reaction at room temperature S-47

10.6. Reaction with aldehyde as the limiting reagent S-49

10.7. Reactions with different aldehydes S-51

10.7.1. Octanal S-51

10.7.2. Isovaleraldehyde S-54

10.7.3. Propanal S-56

10.7.4. δ-Valerolactol S-58

10.7.5. Pentanal S-60

10.7.6. 5-bromopentanal S-62

10.7.7. Dodecanal S-64

11. Example of the calibration of FTIR data S-66

12. Method and data for the assessment of yield by NMR S-68

13. NMR spectra of enamines and α-chlorinated products S-83

14. Determination of the enantiomeric ratio of α-chloroalcohol products S-99

15. References S-113

1. General information

Commercially available aldehydes were carefully distilled under vacuum prior to use. The δ-valerolactol was synthesized by DIBAL-H reduction of δ-valerolactone and was purified by column chromatography (9:1 hexane:EtOAc). The 5-bromopentanal was synthesized by PCC oxidation of 5-bromopentanol and purified by column chromatography (3:1 hexane:Et_2O). The *N*-chlorosuccinimide (NCS) and *N*-chlorophthalimide (NCP) were recrystallized from methanol. (*S*)-α,α-bis[3,5-bis(trifluoromethyl)-phenyl]-2-pyrrolidinemethanol trimethylsilyl ether (**3a**) and (*S*)-α,α-bis[3,5-bis(trifluoromethyl)-phenyl]-2-pyrrolidinemethanol *tert*-butyldimethylsilyl ether (**3b**) catalysts were purified from commercial sources by flash column chromatography (CH_2Cl_2) to remove any deprotected alcohol. Hexafluoroisopropanol (HFIP) was dried over activated molecular sieves (4 Å). All other reagents and solvents were used as purchased from Merck, Fluorochem, Alfa Aesar and TCI.

All IR spectra were taken with a Mettler Toledo ReactIR 15 equipped with a LN_2 MCT detector and a 9.5 mm AgX Fibre DiComp probe. The FTIR data underwent 2nd derivative processing using the standard function in the Mettler-Toledo iC IR software. This function applies a 7-point Savitzky-Golay filter and an inversion. The following characteristic resonances were used for the monitoring of reaction progress: 1003 cm^{-1} (succinimide), 967 cm^{-1} (NCS), 869 cm^{-1} (NCP) and 721 cm^{-1} (phthalimide). The data was calibrated using a standard addition of a stock solution of either succinimide or phthalimide. All NMR spectra were recorded on a Bruker AVII 500 MHz spectrometer or a Bruker AVIII HD 400 MHz spectrometer with BBO prodigy probe. ^{1}H NMR and ^{13}C NMR chemicals shifts (δ) are quoted in ppm relative to residual solvent peaks (for ^{1}H and ^{13}C respectively, given in ppm: $CDCl_3$: 7.26, 77.16; CD_2Cl_2: 5.32, 54.00; toluene-d_8: 2.08, 20.40; methanol-d_4: 3.31, 49.00; DMSO-d_6: 2.50, 39.52; THF-d_8: 3.58, 67.57; MeCN-d_3: 1.94, 1.32). The non-deuterated NMR spectra were recorded after shimming on the solvent peak closest to the middle of the spectrum and are reported with respect to the shift of this solvent peak aligned with its position in $CDCl_3$ (for ^{1}H and ^{13}C respectively, given in ppm: MTBE: 1.19, 49.50; IPA: 1.20, 25.60; HFIP: 4.49, 69.20). Slow additions were carried out using a Harvard Apparatus standard infuse/withdraw pump 11 elite programmable syringe pump calibrated to the syringe; either a Henke-Sans-Wolfe Air-tight 2.5 mL or Hamilton 1700 series 500 µL.

Flash column chromatography was performed using 230-400 mesh silica, with the indicated solvent system according to standard techniques. Analytical thin-layer chromatography (TLC) and preparative thin-layer chromatography were performed on precoated glass-backed silica gel plates (Supelco TLC Silica gel 60 F_{254}). Visualization of the developed chromatogram was performed by UV absorbance (254 nm), aqueous potassium permanganate solution or anisaldehyde stain.

Yields of chlorinated aldehydes were calculated from the FTIR data and the mono:dichlorination ratio from crude ^{1}H NMR. The yields were confirmed by NMR with an internal standard (trimethoxybenzene or tetrachloroethane). Reported ^{1}H and ^{13}C NMR data for all compounds matched literature data. Enantiomeric ratios were measured from a sample prepared from the same stock solutions and run concurrently with the ReactIR sample but quenched by reduction immediately at the end of the slow addition to minimize potential product racemization. Chiral HPLC was carried out on an Agilent 1260 Infinity II LC equipped with a diode array detector.

2. Previous studies on the mechanism of the aminocatalytic α-chlorination of aldehydes

The mechanism of aminocatalytic reactions proceeding via enamines have been studied extensively by Blackmond, Seebach, Wennemers, Pápai and Reiher.[1a–f] More specifically, mechanistic studies of α-chlorination reactions were performed by Blackmond *et al.* They demonstrated that though the reaction is zero order in chlorinating agent, the overall rate of reaction changed depending on whether NCS or NCP were used. They also observed and characterized 1,2-aminal adducts (***E*** and ***Z*** below), the equilibrium ratio of which correlated directly with the enantiomeric ratio of the chlorinated product.

These two observations, together with the knowledge from other reactions, allowed Blackmond to propose the following catalytic cycle (red and blue pathways). Here, it is not just the intrinsic facial selectivity of the enamine that determines the enantiomeric ratio of the product, but the position of the equilibrium between the two rapidly exchanging diastereomeric adducts.

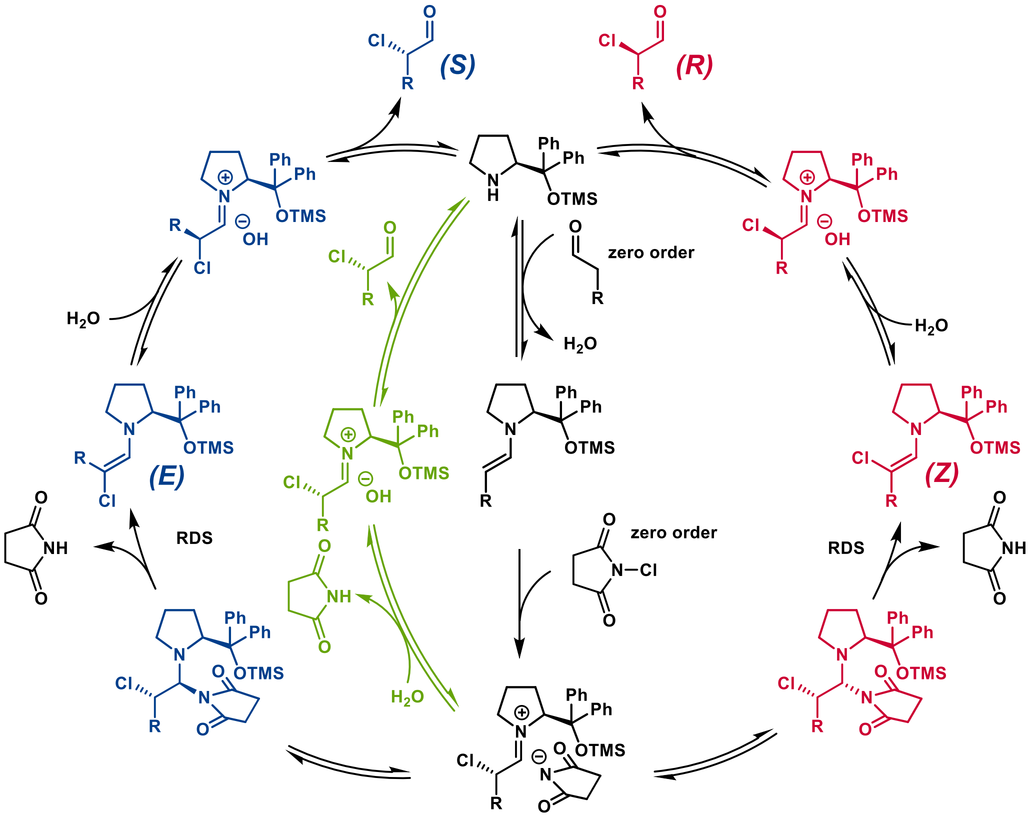

Scheme 1 – The catalytic cycle demonstrates the negative effect of downstream pathway bifurcation on the enantioenrichment of the product.

Based on this mechanism we proposed that any selective methodology for the aminocatalytic α-chlorination of aldehydes must favor a single diastereomer of the 1,2-aminal adducts or omit them from the catalytic cycle entirely. To achieve this, we suggested the use of HFIP to stabilize the charged iminium ion intermediate versus the aminal species and allow its direct hydrolysis to highly enantioenriched product (green pathway).

3. Previous methodologies for the aminocatalytic α-chlorination of aldehydes

Several methodologies for the aminocatalytic α-chlorination of aldehydes have been developed that provide excellent selectivities and yields. To achieve these results, the procedures must allow deviations from the natural reaction pathway in normal organic solvents. To do so, the authors have developed methodologies that display some drawbacks. In the case of entry 1 (Table 1)[2] the chlorinating agent is unusual (expensive and has poor atom economy when compared to NCS) and the required temperatures are impractical. The methodology shown in entry 2[3] uses a peculiar aminocatalyst. The SOMO methodology (entry 3)[4] requires a very high catalyst loading and 50 mol% of $Cu(TFA)_2$. The methodology shown in entry 4[5] also employs a very high catalyst loading and an expensive source of chlorine. The final example (entry 5)[6] required a non-commercial catalyst and chlorinating agent, low temperature and a very long reaction time.

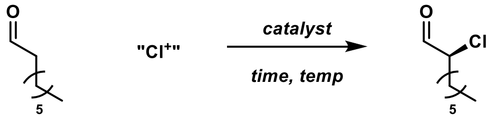

Entry	Catalyst	Cat / mol%	"Cl+"	Temp / °C	Time / h	Yield / %	ee / %	Method
1	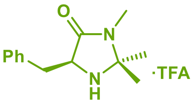	5	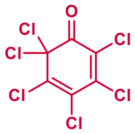	**–30**	6	71	92	MacMillan *et al.* *J. Am. Chem. Soc.* **2004**, 4108
2	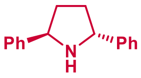	**10**	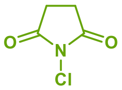	0	6	99	95	Jørgensen *et al.* *J. Am. Chem. Soc.* **2004**, 4790
3	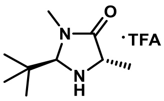	**20**	LiCl 50 mol% $Cu(TFA)_2$	10	4	90	**96**	MacMillan *et al.* *Angew. Chem. Int. Ed.* **2009**, 5121
4	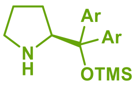	**20**	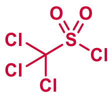	**rt**	**2**	89	93	Renaud *et al.* *J. Org. Chem.* **2016**, 1251
5	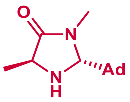 **not commercially available**	5	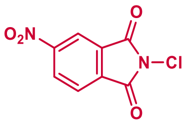 **not commercially available**	**–30**	**48**	78	**97**	Christmann *et al.* *Angew. Chem. Int. Ed.* **2018**, 11683
6	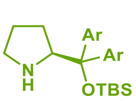	**2**	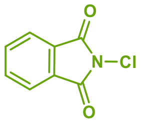	**0**	**1**	**76**	**98**	**This work**

Table 1 - Examples shown for octanal, as it was common to all procedures.

In contrast, the method we describe (Entry 6) uses commercially available catalysts and chlorinating agents (NCS or NCP). We report short reaction times and very low catalyst loading while using readily attainable temperatures. Potential downsides include the use of HFIP, but this is volatile and easily recoverable, and the need for slow addition of the chlorinating agent.

3.1. Michael P. Brochu, Sean P. Brown and David W. C. MacMillan. *J. Am. Chem. Soc.* **2004**, *126*, 13, 4108–4109

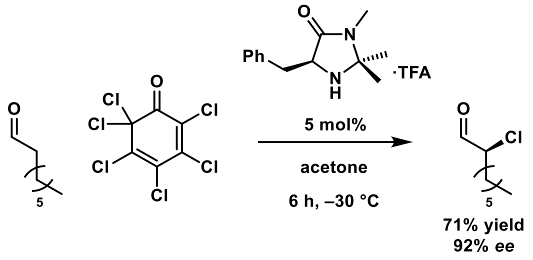

MacMillan *et al.* described the first enantioselective aminocatalytic α-chlorination of aldehydes using an unusual chlorinating agent, 2,2,3,4,5,6-hexachlorocyclohexadienone. This chlorinating agent forms, as a byproduct, the non-coordinating counterion pentachlorophenolate, for which 1,2-aminal adducts have not been observed. Therefore, the enantiomeric ratio of the products may be determined predominantly by the intrinsic facial selectivity of the enamine in the chlorination step and the erosion of the enantioselectivity of the overall reaction is prevented.

This methodology employs 2,2,3,4,5,6-hexachlorocyclohexadienone, an uneconomical and atom inefficient chlorinating agent, and impractical temperatures (– 30 °C).

3.2. Nis Halland, Alan Braunton, Stephan Bachmann, Mauro Marigo and Karl Anker Jørgensen. *J. Am. Chem. Soc.* **2004**, *126*, 15, 4790–4791

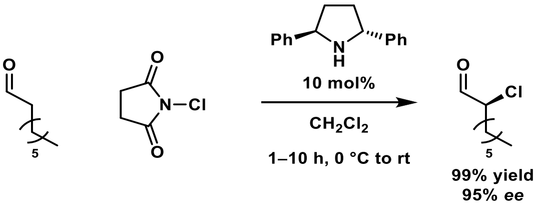

Jørgensen *et al*. proposed a method with NCS, a more convenient chlorinating agent than 2,2,3,4,5,6-hexachlorocyclohexadienone. However, to obtain excellent enantioselectivities and yields they required 10 mol% of an unusual aminocatalyst, (2*R*,5*R*)-diphenylpyrrolidine. This catalyst favors one of the two possible 1,2-aminal adducts during the reaction, as shown by Blackmond *et al.*[1a]

The (2R,5R)-diphenylpyrrolidine catalyst currently costs £674/mmol, maybe because it is less commonly used than the Jørgensen-Hayashi type catalysts.*

*https://www.tcichemicals.com/GB/en/p/D3185 accessed 04/21

3.3. Muriel Amatore, Teresa D. Beeson, Sean P. Brown and David W. C. MacMillan. *Angew. Chem. Int. Ed.* **2009**, *48*, 5121–5124

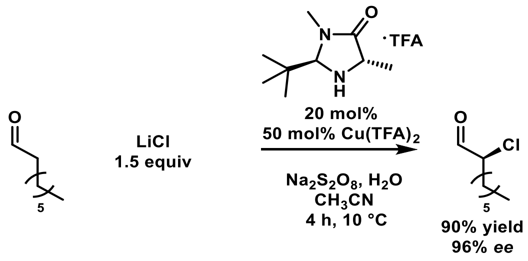

MacMillan *et al.* used an innovative SOMO methodology to chlorinate aldehydes enantioselectively. The mechanism involves the formation of radical species and it probably avoids the formation of the 1,2-aminal adducts as on-cycle catalytic intermediates.

MacMillan et al. use 50 mol% of expensive $Cu(TFA)_2$, leading to potential metal contamination, and a high aminocatalyst loading (20 mol%). Additionally, the more common and commercially available MacMillan aminocatalyst ((2S,5S)-(–)-2-tert-Butyl-3-methyl-5-benzyl-4-imidazolidinone) gave poor selectivity with this methodology.

3.4. Ciril Jimeno, Lidong Cao and Philippe Renaud. *J. Org. Chem.* **2016**, *81*, 1251–1255

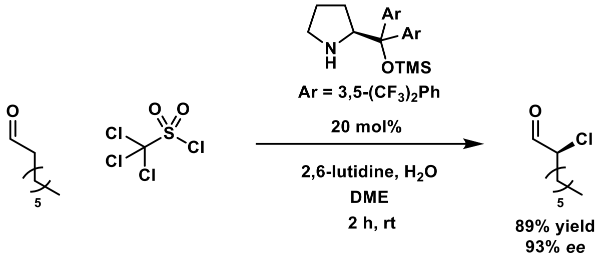

Renaud *et al.* used trichloromethanesulfonyl chloride in their α-chlorination of aldehydes. The chlorination step does not generate a coordinating species as a byproduct and therefore, the formation 1,2-aminal adducts is avoided.

Trichloromethanesulfonyl chloride is expensive. In addition, the aminocatalyst loading is high (20%).

3.5. Sebastian Ponath, Martina Menger, Lydia Grothues, Manuela Weber, Dieter Lentz, Carsten Strohmann and Mathias Christmann. *Angew. Chem. Int. Ed.* **2018**, *57*, 11683–11687

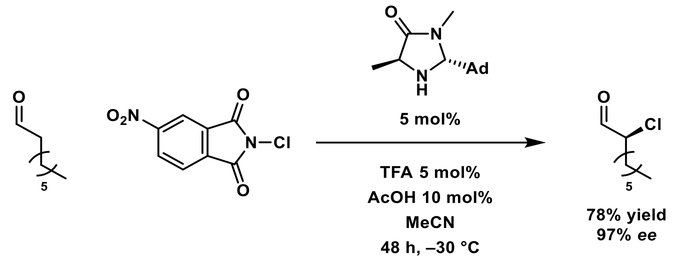

Christmann *et al.* reported the most recent method for the highly enantioselective aminocatalytic chlorination of aldehydes. They used a very sterically hindered aminocatalyst, which may disfavor the elimination of the 1,2-aminal adducts with respect to less congested aminocatalysts. They also used a modified, electron-poor *N*-chlorophthalimide and a combination of acids to reduce the percentage of catalyst as 1,2-aminal adducts.

The optimized reaction conditions described by Christmann et al. used a non-commercially available chlorinating agent and aminocatalyst. In addition, the temperature (–30 °C) is impractical and the reaction time is very long (48 h).

4. Study of the stability of enamines and iminium ions

To prove that HFIP is unique in its capacity for iminium ion stabilization, mixtures of catalyst **3a** (9.0 mg, 0.015 mmol, 4 mol%) and hydrocinnamaldehyde (49.7 mg, 0.37 mmol, 1.0 equiv) were prepared in various solvents (total volume 0.6 mL). In each solvent, a new set of signals, different to the signals of the hydrocinnamaldehyde and free catalyst, appeared in each ^{1}H NMR spectrum. We assigned these new signals to the enamine or iminium ion of catalyst **3a** and hydrocinnamaldehyde using 2D NMR spectroscopy including HSQC, HMBC and COSY experiments. The distribution of catalytic species in each solvent, determined by the ratio of integrals of the TMS signals in the first ^{1}H NMR spectra, is displayed in Table 2. The iminium ion was only observed in HFIP.

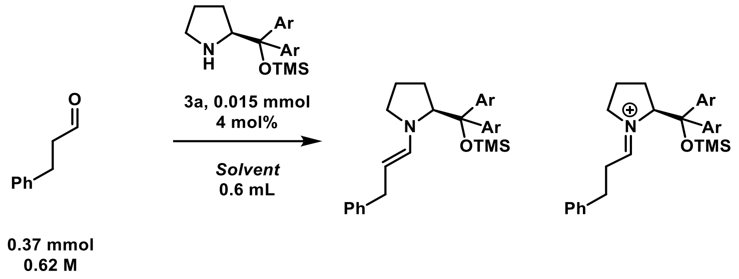

Solvent	Catalytic species / %		
	Enamine	Iminium ion	Free Catalyst
**CD_2Cl_2**	61	–	39
**$CDCl_3$**	61	–	39
**MeCN-d_3**	92	–	8
**toluene-d_8**	71	–	29
**methanol-d_4[a]**	57	–	43
**DMSO-d_6**	80	–	20
**THF-d_8**	93	–	7
**MTBE**	90	–	10
**IPA**	66	–	34
**HFIP**	–	**97**	**3**

*Table 2 – HFIP is the only solvent with an observable iminium ion of catalyst **3a** and hydrocinnamaldehyde. a: Characterized with the assistance of low temperature (–50 °C) 2D NMR to avoid self-aldol reaction.*

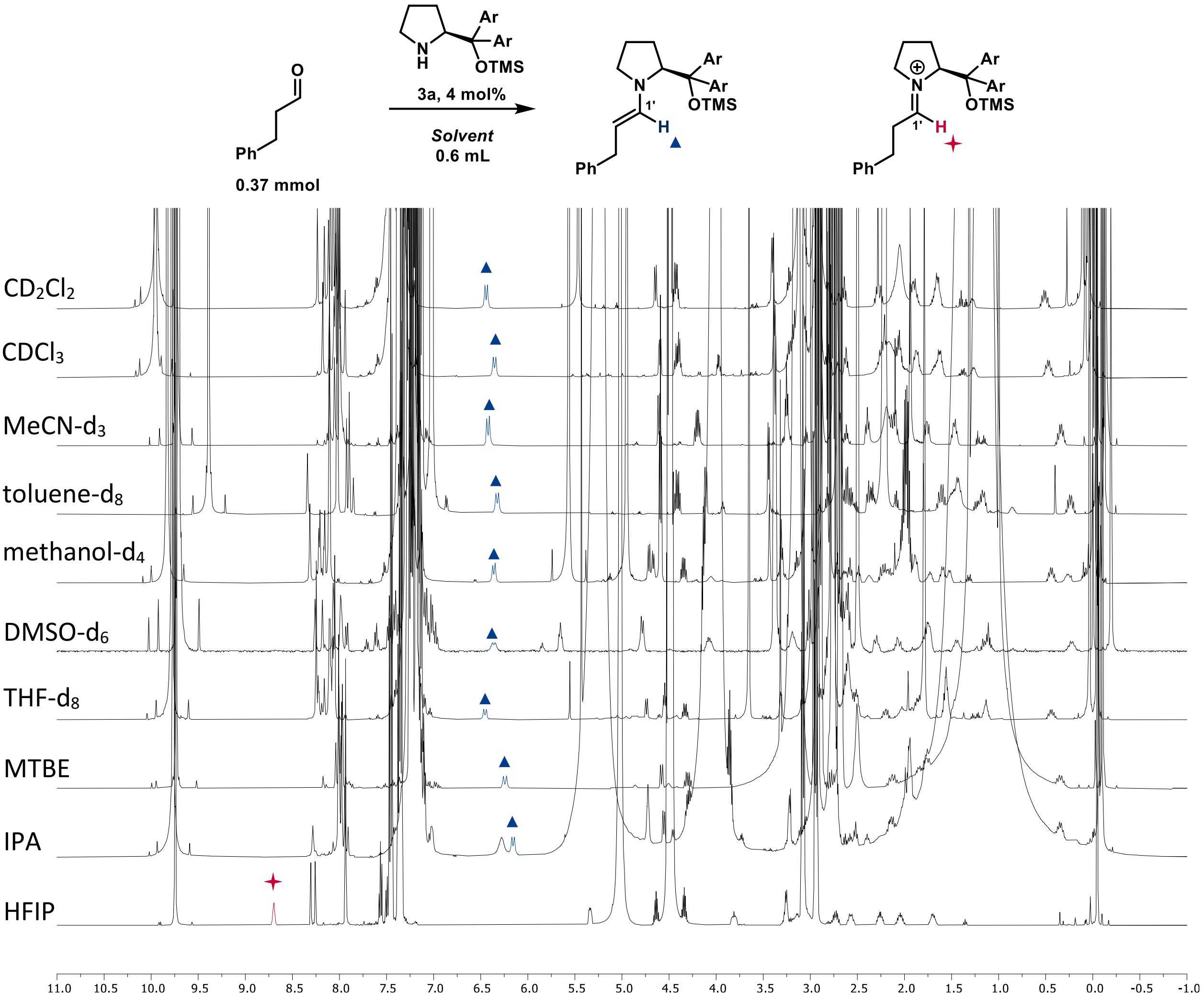

Figure 1 – Stacked ^1H NMR (500 MHz) spectra highlight the enamine/iminium ion formation in different solvents via characteristic H(1').

4.1. Spectroscopic description of the enamine in standard organic solvents

^1H NMR (500 MHz, CD_2Cl_2) δ (ppm) 8.01 (s, 1H; ArH), 7.97 (s, 1H; ArH), 7.95 (s, 2H; ArH), 7.92 (s, 2H; ArH), 7.35 – 7.26 (m, 5H, PhH), 6.30 (d, *J* = 13.4 Hz, 1H; C(1')H), 4.50 (dd, *J* = 9.3, 2.1 Hz, 1H; C(2)H), 4.32 (dt, *J* = 13.4, 7.2 Hz, 1H; C(2')H), 3.26 (d, *J* = 7.2 Hz, 2H; C(3')H), 2.82 (m, 1H, C(5)H), 2.49 (td, *J* = 9.2, 2.7 Hz, 1H; C(5)H), 2.17 – 2.09 (m, 1H; C(3)H), 1.79 – 1.72 (m, 1H; C(3)H), 1.57 – 1.47 (m, 1H; C(4)H), 0.41 – 0.32 (m, 1H; C(4)H), -0.10 (s, 9H, OTMS). **^{13}C NMR (125 MHz, CD_2Cl_2)** δ (ppm) 145.2, 143.1, 137.8, 130.1, 129.9, 127.7, 126.5, 122.2, 121.7, 99.0, 82.9, 69.9, 49.7, 37.0, 27.8, 22.9, 1.8 (CF_3 carbon not identified).

^1H NMR (500 MHz, $CDCl_3$) δ (ppm) 8.01 (s, 1H, ArH), 7.97 (s, 1H, ArH), 7.95 (s, 2H, ArH), 7.92 (s, 2H, ArH), 7.40 – 7.21 (m, 5H, PhH), 6.26 (d, *J* = 13.4 Hz, 1H, C(1')H), 4.50 (m, 1H, C(2)H), 4.32 (dt, *J* = 13.4, 6.8 Hz, 1H, C(2')H), 3.29 (d, *J* = 6.8 Hz, 2H, C(3')H), 2.83 (m, 1H, C(5)H), 2.55 – 2.51 (m, 1H, C(5)H), 2.19 – 2.11 (m, 1H, C(3)H), 1.81 – 1.75 (m, 1H, C(3)H), 1.57 – 1.50 (m, 1H, C(4)H), 0.41 – 0.33 (m, 1H, C(4)H), -0.08 (s, 9H, OTMS). **^{13}C NMR (125 MHz, $CDCl_3$)** δ (ppm) 144.1, 143.0, 137.5, 129.8, 128.5, 128.0, 126.3, 124.4, 122.3, 99.0, 83.6, 69.7, 49.0, 36.7, 28.0, 22.5, 1.7 (CF_3 carbon not identified).

^{1}H NMR (500 MHz, MeCN-*d*$_3$) δ (ppm) 8.11 (s, 1H, ArH), 8.05 (s, 1H, ArH), 8.00 (s, 4H, ArH), 7.33 – 7.16 (m, 5H, PhH) 6.42 (d, *J* = 13.7 Hz, 1H, C(1')H), 4.61 (dd, *J* = 9.4, 2.0 Hz, 1H, C(2)H), 4.20 (dt, *J* = 13.7, 6.9 Hz, 1H, C(2')H), 3.25 (t, *J* = 6.9 Hz, 2H, C(3')H), 2.76–2.69 (m, 1H, C(5)H), 2.40 (td, *J* = 9.1, 2.7 Hz, 1H, C(5)H), 2.18–2.11 (m, 1h, C(3)H), 1.76 (ddt, *J* = 13.5, 8.5, 2.2 Hz, 1H, C(3)H), 1.52 – 1.41 (m, 1H, C(4)H), 0.42 – 0.24 (m, 1H, C(4)H), -0.13 (s, 9H, OTMS). **^{13}C NMR (125 MHz, MeCN-*d*$_3$)** δ (ppm) 146.5, 145.5, 144.4, 139.2, 131.0, 130.9, 129.2, 129.1, 126.6, 98.5, 84.6, 70.1, 50.0, 37.5, 28.1, 23.4, 1.9 (CF_3 Carbon not identified).

^{1}H NMR (500 MHz, toluene-*d*$_8$) δ (ppm) 7.92 (d, *J* = 1.6 Hz, 4H, ArH), 7.82 (s, 1H, ArH), 7.79 (s, 1H, ArH), 7.29 – 7.19 (m, 5H, PhH), 6.22 (d, *J* = 13.7 Hz, 1H, C(1')H), 4.31 (dt, *J* = 13.7, 7.0 Hz, 1H, C(2')H), 4.01 (dd, *J* = 9.3, 2.1 Hz, 1H, C(2)H), 3.34 (d, *J* = 7.0 Hz, 2H, C(3')H), 2.26 – 2.20 (m, 1H, C(5)H), 2.28 (m, 1H, C(5)H), 1.55 – 1.46 (m, 1H, C(3)H), 1.33 (m, 1H, C(3)H), 1.10 – 1.02 (m, 1H, C(4)H), 0.18 – 0.08 (m, 1H, C4)H), -0.23 (s, 9H, OTMS). **^{13}C NMR (125 MHz, toluene-*d*$_8$)** δ (ppm) 145.7, 143.1, 137.4, 130.0, 128.4, 128.0, 125.8, 124.9, 122.0, 99.2, 83.5, 69.3, 49.3, 37.2, 27.2, 22.5, 1.5 (CF_3 carbon not identified).

^{1}H NMR (500 MHz, methanol-*d*$_4$) δ (ppm) 8.22 (s, 1H, ArH), 8.11 (s, 1H, ArH), 8.01 (s, 4H, ArH), 7.47 – 7.08 (m, 5H, PhH), 6.26 (d, *J* = 13.5 Hz, 1H, C(1')H), 4.61 (dd, *J* = 9.2, 2.0 Hz, 1H, C(2)H), 4.24 (dt, *J* = 13.5, 7.1 Hz, 1H, C(2')H), 3.20 (d, *J* = 7.1 Hz, 2H, C(3')H), 2.75 (m, 1H, C(5)H), 2.50 – 2.46 (m, 1H, C(5)H), 2.18 – 2.10 (m, 1H, C(3)H), 1.81 – 1.77 (m, 1H, C(3)H), 1.53 – 1.47 (m, 1H, C(4)H), 0.39 – 0.29 (m, 1H, C(4)H), -0.11 (s, 9H, OTMS). **^{13}C NMR (125 MHz, methanol-*d*$_4$)** δ (ppm) 145.7, 141.2, 129.8, 129.7, 128.2, 126.1, 124.6, 122.2, 121.8, 96.1, 82.3, 71.4, 46.1, 42.0, 30.9, 21.0, 0.9 (CF_3 carbon not identified).

^{1}H NMR (500 MHz, DMSO-*d*$_6$) δ (ppm) 8.26 (s, 1H, ArH), 8.18 (s, 1H, ArH), 8.07 (s, 2H, ArH), 7.99 (s, 2H, ArH), 7.54 – 6.91 (m, 5H, PhH), 6.33 (d, *J* = 13.8 Hz, 1H, C(1')H), 4.78 (dd, *J* = 9.3, 1.8 Hz, 1H, C(2)H), 4.07 (dt, *J* = 13.8, 7.1 Hz, 1H, C(2')H), 3.21 – 3.17 (m, 2H, C(3')H), 2.70 – 2.57 (m, 1H, C(5)H), 2.32 – 2.27 (m, 1H, C(5)H), 2.10 – 2.01 (m, 1H, C(3)H), 1.78 – 1.72 (m, 1H, C(3)H), 1.48 – 1.40 (m, 1H, C(4)H), 0.27 – 0.18 (m, 1H, C(4)H), -0.19 (s, 9H, OTMS). **^{13}C NMR (125 MHz, DMSO-*d*$_6$)** δ (ppm) 145.7, 143.1, 138.3, 130.2, 128.3, 126.4, 124.8, 124.8, 122.3, 97.1, 83.8, 68.6, 49.2, 36.7, 27.2, 22.6, 1.6 (CF_3 carbon not identified).

^{1}H NMR (500 MHz, THF-*d*$_8$) δ (ppm) 8.20 (s, 1H, ArH), 8.14 (s, 1H, ArH), 8.09 (s, 2H, ArH), 8.08 (s, 2H, ArH), 7.43 – 7.08 (m, 5H, PhH), 6.39 (d, *J* = 13.8 Hz, 1H, C(1')H), 4.71 (dd, *J* = 9.5, 2.0 Hz, 1H, C(2)H), 4.31 (dt, *J* = 13.8, 7.2 Hz, 1H, C(2')H), 3.28 (dd, *J* = 7.2, 3.2 Hz, 2H, C(3')H), 2.78 (m, 1H, C(5)H), 2.48 (m, 1H, C(5)H), 2.22 – 2.13 (m, 1H, C(3)H), 1.87 – 1.81 (m, 1H, C(3)H), 1.56 – 1.50 (m, 1H, C(4)H), 0.47 – 0.37 (m, 1H, C(4)H), -0.06 (s, 9H, OTMS). **^{13}C NMR (125 MHz, THF-*d*$_8$)** δ (ppm) 145.3, 143.8, 137.9, 129.6, 128.2, 127.8, 125.7, 124.5, 121.9, 98.4, 83.8, 69.2, 49.2, 36.6, 27.2, 22.6, 0.8 (CF_3 carbon not identified).

^{1}**H NMR (500 MHz, MTBE)** δ (ppm) 8.06 (s, 1H, ArH), 8.02 (s, 2H, ArH), 8.00 (s, 1H, ArH), 7.99 (s, 2H, ArH), 7.45 – 7.01 (m, 5H, PhH), 6.25 (d, *J* = 13.8 Hz, 1H, C(1')H), 4.59 (dd, *J* = 9.3, 1.7 Hz, 1H, C(2)H), 4.33 (dt, *J* = 13.8, 7.0 Hz, 1H, C(2')H), 3.27 (m, 2H, C(3')H), 2.88 – 2.76 (m, 1H, C(5)H), 2.61 – 2.49 (m, 1H, C(5)H), 2.20 – 2.10 (m, 1H, C(3)H), 1.81 – 1.77 (m, 1H, C(3)H), 1.54 (m, 1H, C(4)H), 0.41 – 0.31 (m, 1H, C(4)H), -0.09 (s, 9H, OTMS). ^{13}**C NMR (125 MHz, MTBE)** δ (ppm) 145.7, 140.6, 133.7, 129.5, 128.3, 126.9, 125.8, 124.8, 121.5, 99.3, 83.8, 69.6, 49.5, 36.0, 29.9, 23.2, 1.5 (CF_3 carbon not identified).

^{1}**H NMR (500 MHz, IPA)** δ (ppm) 7.91 (s, 4H, ArH), 7.88 (s, 2H, ArH), 7.40 – 7.21 (m, 5H, PhH), 6.05 (d, *J* = 13.8 Hz, 1H, C(1')H), 4.46 (d, *J* = 9.0 Hz, 1H, C(2)H), 4.20 (dt, *J* = 13.8, 6.7 Hz, 1H, C(2')H), 3.11 – 3.02 (m, 2H, C(3')H), 2.68 (m, 1H, C(5)H), 2.44 – 2.40 (m, 1H, C(5)H), 2.09 – 2.00 (m, 1H, C(3)H), 1.70 – 1.65 (m, 1H, C(3)H), 1.40 (m, 1H, C(4)H), 0.29 – 0.20 (m, 1H, C(4)H), -0.19 (s, 9H, OTMS). ^{13}**C NMR (125 MHz, IPA)** δ (ppm) 146.3, 138.4, 130.7, 130.1, 128.4, 128.3, 125.9, 121.5, 99.4, 83.2, 69.9, 48.9, 37.4, 26.4, 21.9, 12.4, 0.7 (CF_3 carbon not identified).

4.2. Spectroscopic description of the iminium ion in HFIP

^{1}**H NMR (500 MHz, HFIP)** δ (ppm) 8.69 (br, 1H; C(1')H), 8.30 (s, 1H; ArH), 8.25 (s, 1H; ArH), 7.93 (s, 4H; ArH), 7.56 – 7.25 (m, 5H, PhH), 5.33 (dd, *J* = 9.2, 5.5 Hz, 1H; C(2)H), 3.81 (ddd, *J* = 14.2, 9.2, 5.5 Hz, 1H; C(5)H), 3.23 (dd, *J* = 6.4, 5.9 Hz, 2H; C(3')H), 3.15 (m, 1H; C(2')H), 2.92 (m, 1H; C(2')H), 2.76 – 2.69 (m, 1H; C(3)H), 2.57 (dt, *J* = 15.3, 9.2 Hz, 1H; C(5)H), 2.25 (ddt, *J* = 14.2, 9.2, 5.5 Hz, 1H; C(3)H), 2.09 – 1.99 (m, 1H; C(4)H), 1.69 (dtt, *J* = 14.2, 9.2, 5.5 Hz, 1H; C(4)H), -0.05 (s, 9H; OTMS). ^{13}**C NMR (125 MHz, HFIP)** δ (ppm) 181.5, 140.1, 136.3, 131.0, 127.9, 127.7, 124.9, 124.1, 119.0, 82.1, 78.2, 53.7, 35.1, 30.0, 25.3, 21.5, -0.8 (CF_3 carbon not identified).

4.3 Example of enamine characterization: CD_2Cl_2

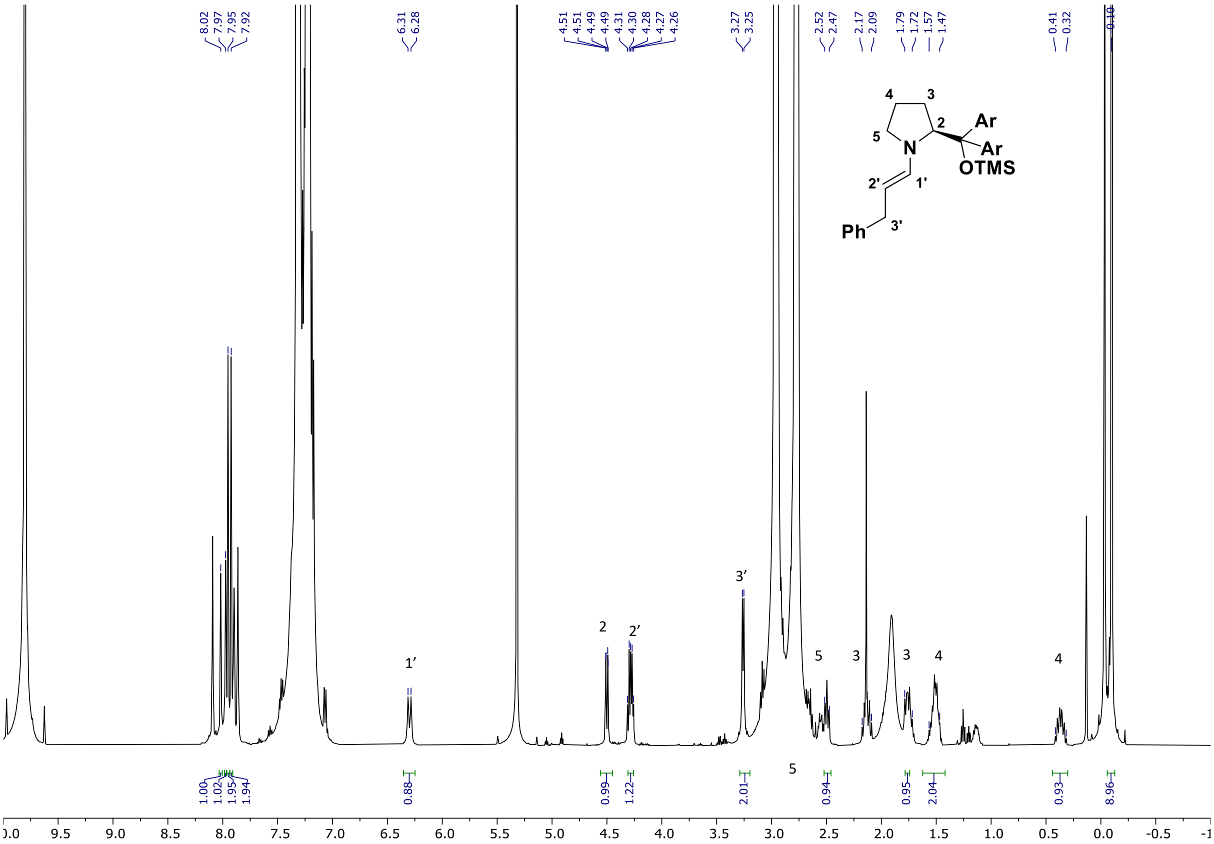

*Figure 2 – ^1H NMR (500 MHz, CD_2Cl_2) spectrum of the enamine of hydrocinnamaldehyde with catalyst **3a**.*

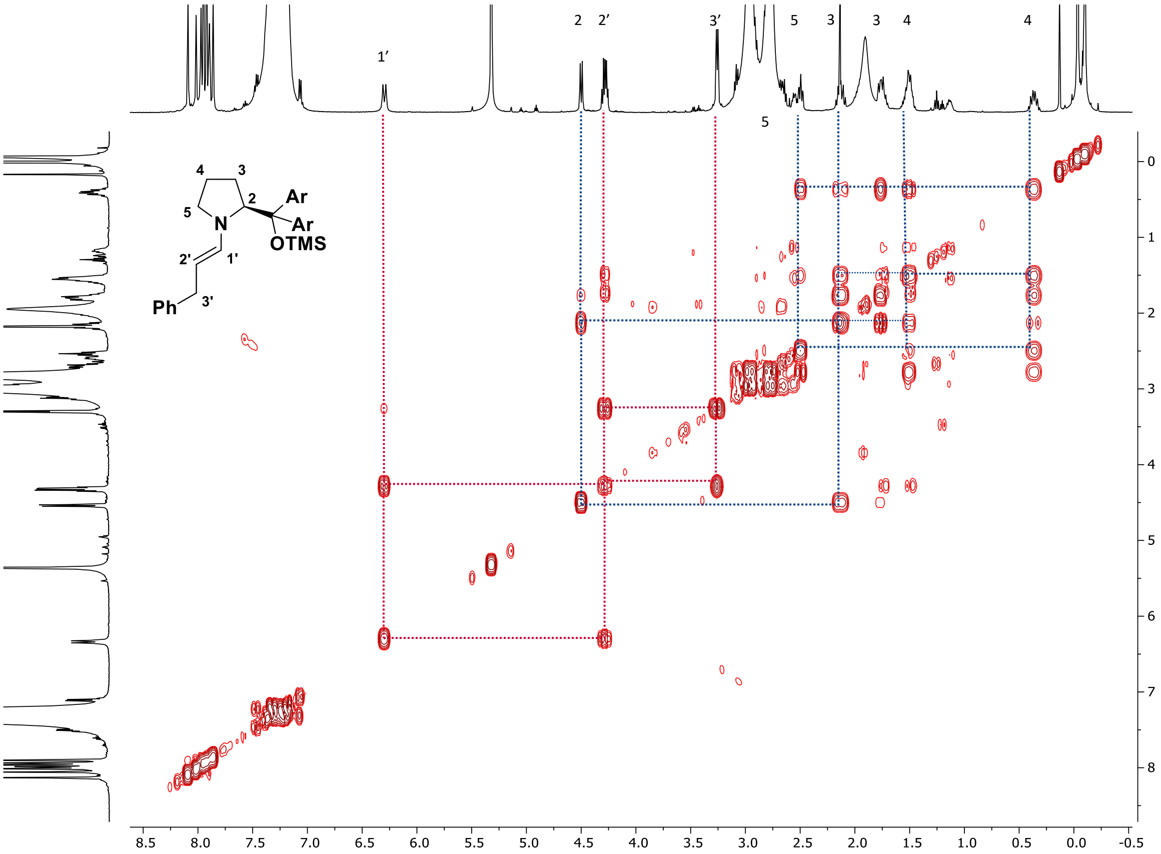

*Figure 3 – COSY NMR (500 MHz, CD_2Cl_2) spectrum of the enamine of hydrocinnamaldehyde with catalyst **3a**.*

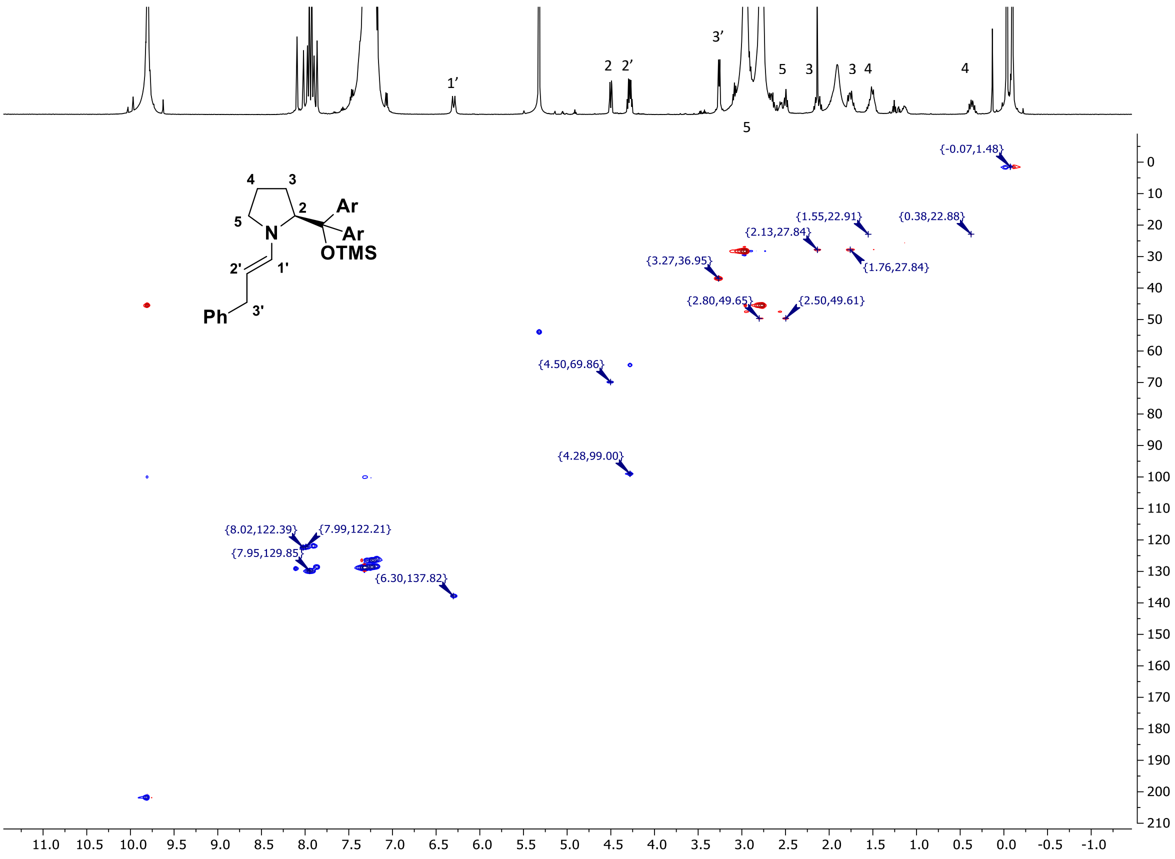

*Figure 4 – HSQC NMR (500 MHz, CD_2Cl_2) spectrum of the enamine of hydrocinnamaldehyde with catalyst **3a**.*

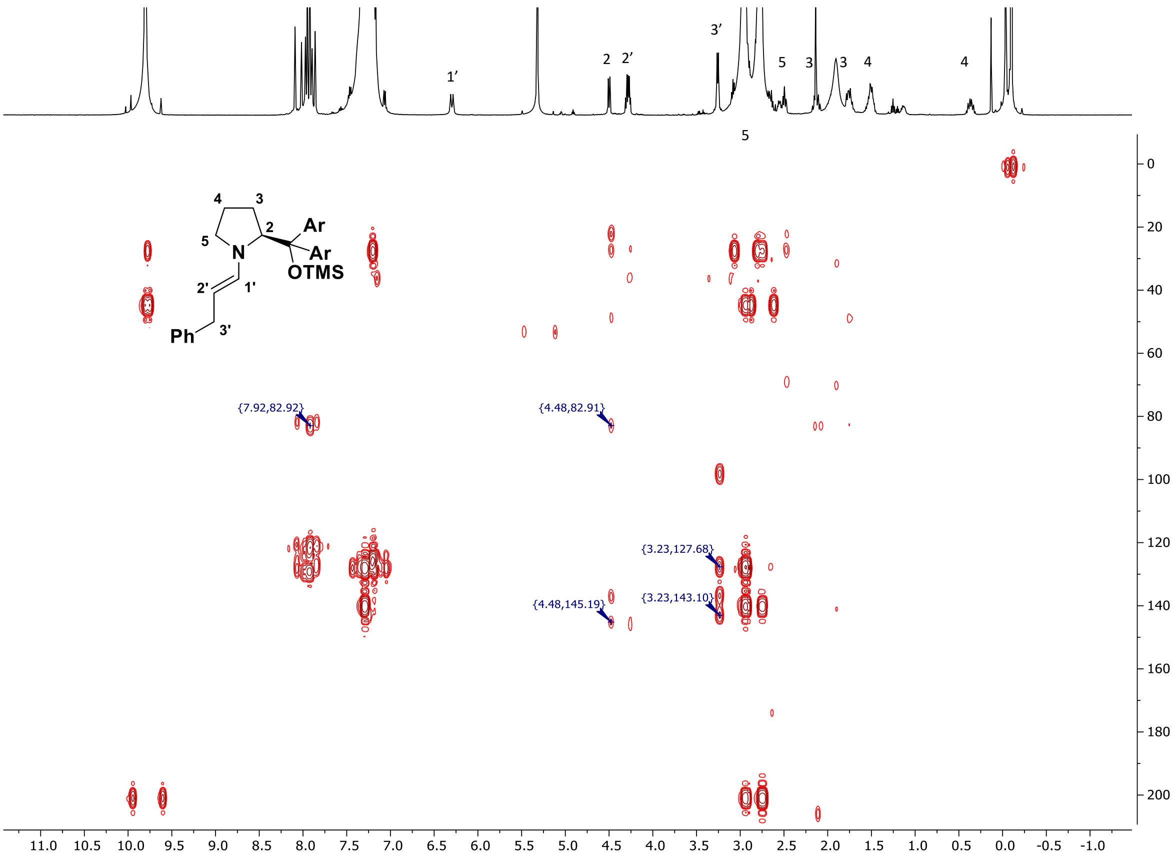

*Figure 5 – HMBC NMR (500 MHz, CD_2Cl_2) spectrum of the enamine of hydrocinnamaldehyde with catalyst **3a**.*

4.4. Iminium ion characterization: HFIP

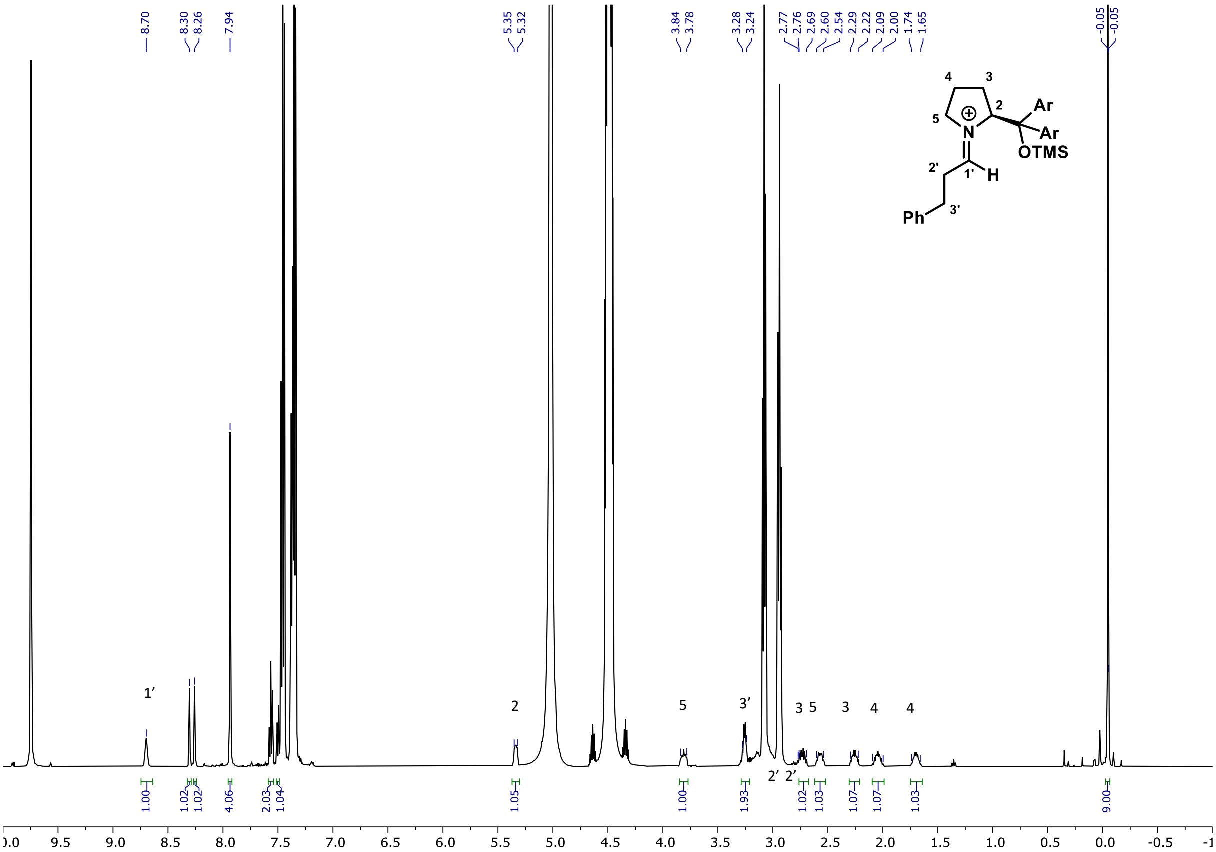

Figure 6 – ^{1}H NMR (500 MHz, HFIP) spectrum of the iminium ion of hydrocinnamaldehyde with catalyst **3a**.

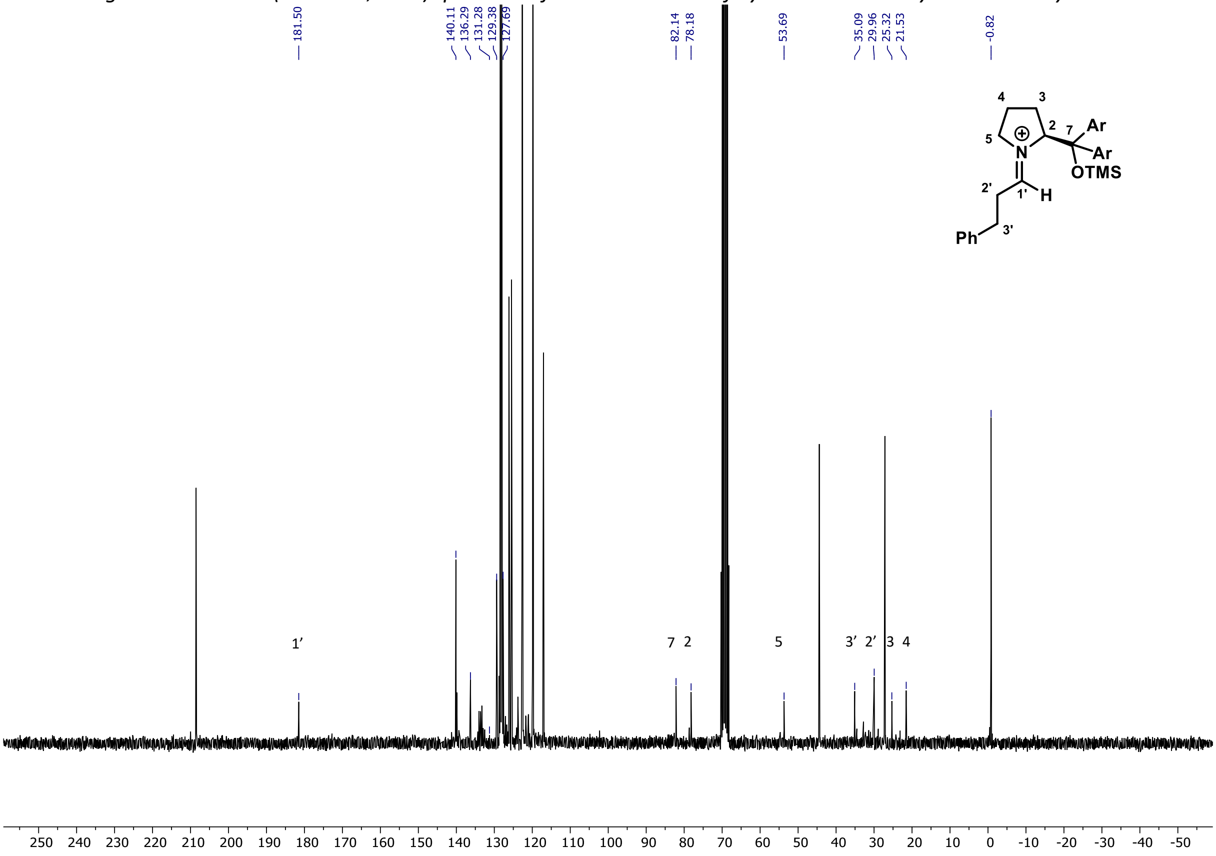

Figure 7 – ^{13}C NMR (125 MHz, HFIP) spectrum of the iminium ion of hydrocinnamaldehyde with catalyst **3a**.

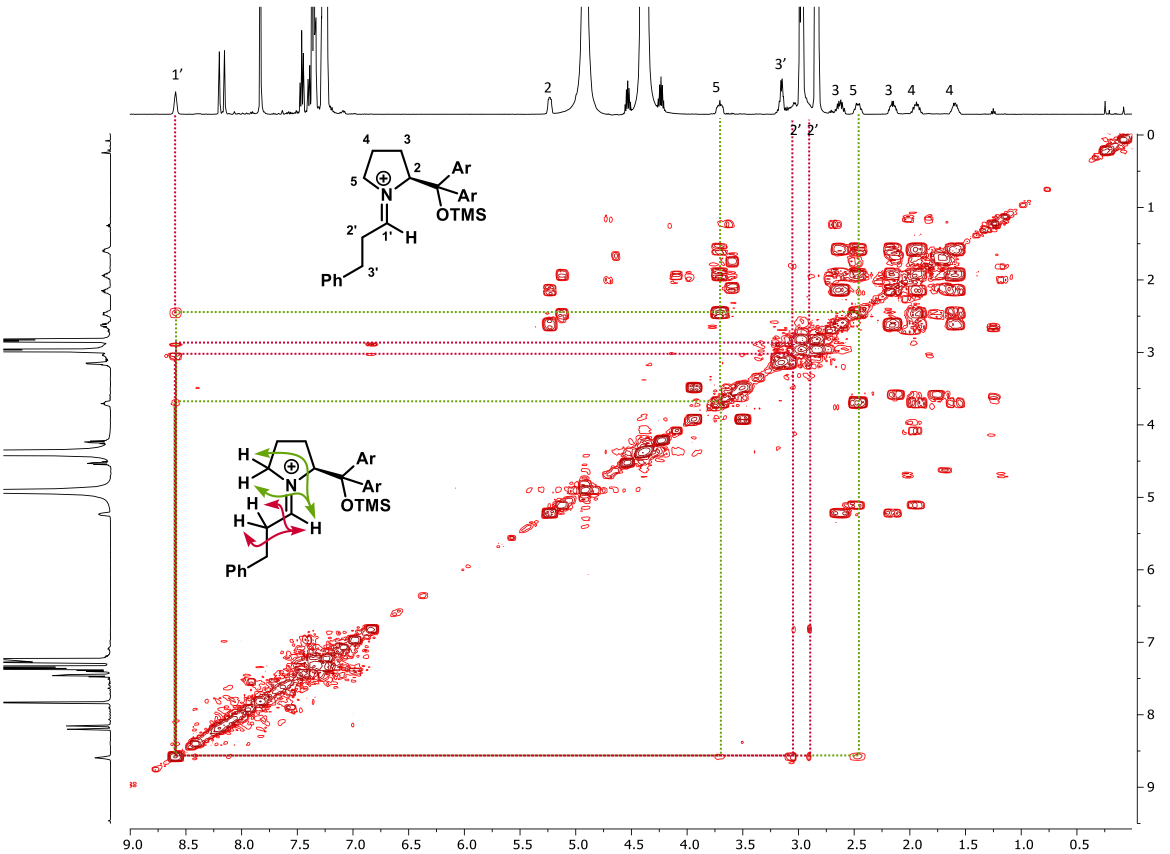

*Figure 8 – COSY NMR (500 MHz, HFIP) spectrum of the iminium ion of hydrocinnamaldehyde with catalyst **3a**. The highlighted coupling between H(1′) and both H(5)s and H(2′)s shows the connectivity between the pyrolidine ring and the aldehyde chain.*

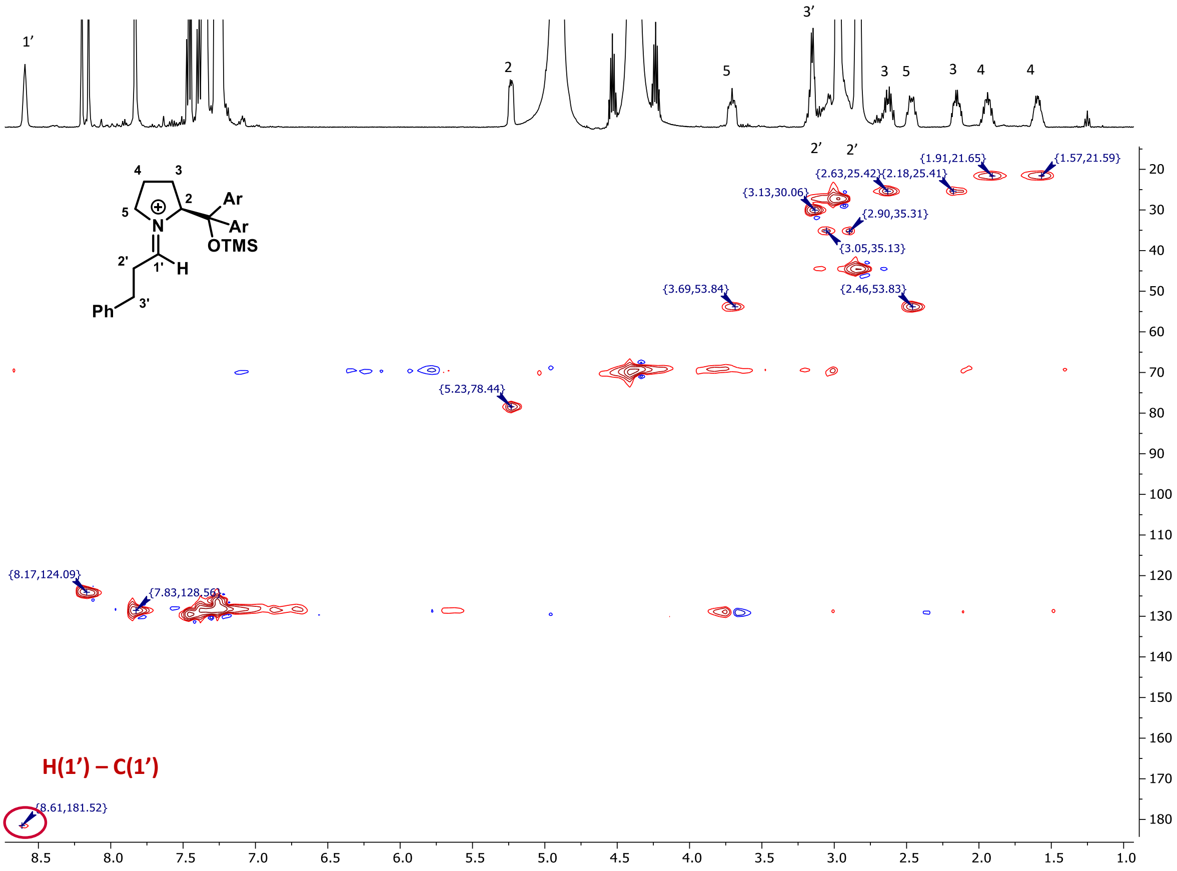

*Figure 9 – HSQC NMR (500 MHz, HFIP) spectrum of the iminium ion of hydrocinnamaldehyde with catalyst **3a**.*

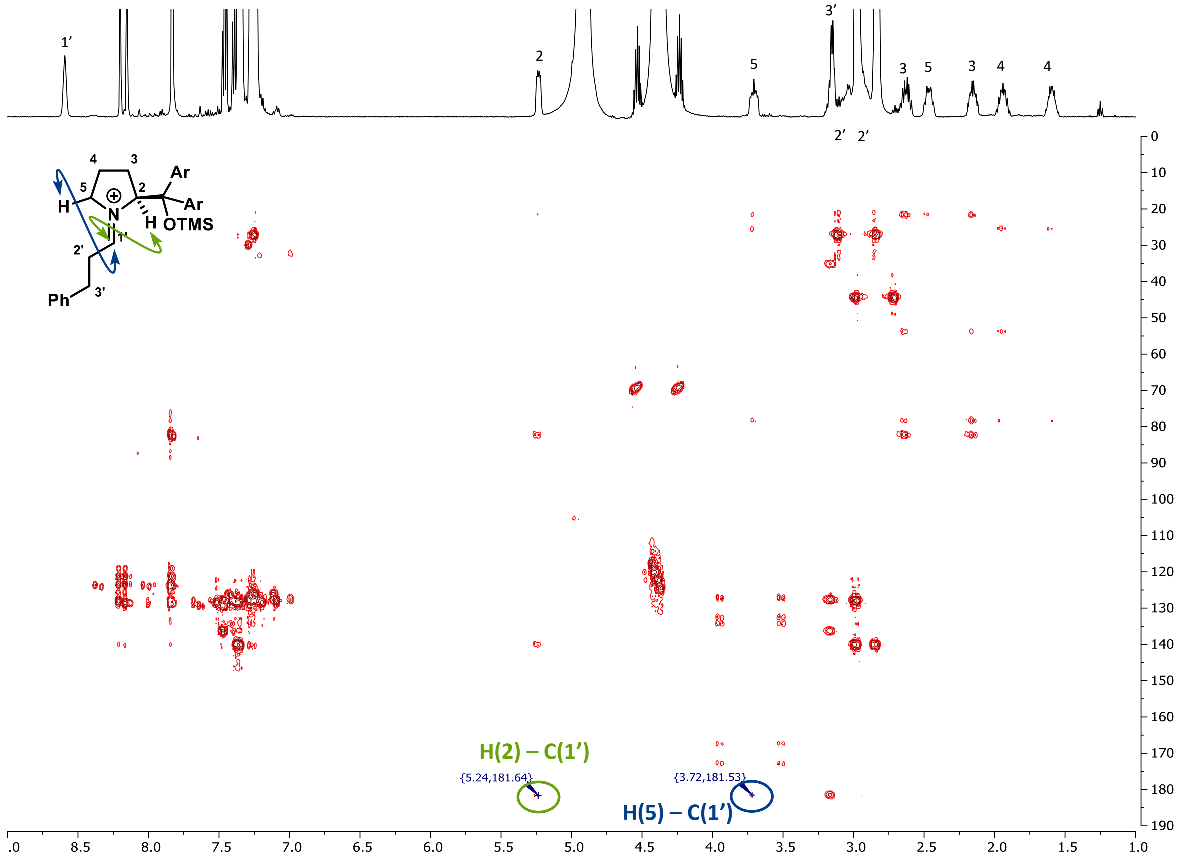

*Figure 10 – HMBC NMR (500 MHz, HFIP) spectrum of the iminium ion of hydrocinnamaldehyde with catalyst **3a**.*

5. Aminocatalytic α-chlorination of aldehydes in HFIP monitored by ^{1}H NMR

Having confirmed that HFIP stabilizes iminium ions, we used HFIP as the solvent for the α-chlorination reaction. The following stock solutions were prepared in HFIP.

SL	Component	Volume / mL	Mass / mg	Concentration / M
**A**	Hydrocinnamaldehyde	1.0	203.0	1.51
**B**	NCS	1.0	79.2	0.59
**C**	cat **3a**	1.0	18.8	0.03

Stock solutions **A** (250 µL, 0.38 mmol, 2.5 equiv) and **B** (250 µL, 0.15 mmol, 1.0 equiv) were added to an NMR tube and placed into the spectrometer to pre-shim. No background reaction was observed. Stock solution **C** (100 µL, 3.0 µmol, 2 mol%) was added to the tube, which was placed into the spectrometer and shimmed as rapidly as possible. A series of ^{1}H NMR experiments (16 scans) with a duration of 90 s each were consecutively run for 12 h. Characteristic aldehyde peaks (9.71, t, starting material; 9.51 ppm, d, monochlorinated product; 9.33 ppm, s, dichlorinated product) were integrated to approximate the conversion for this reaction.

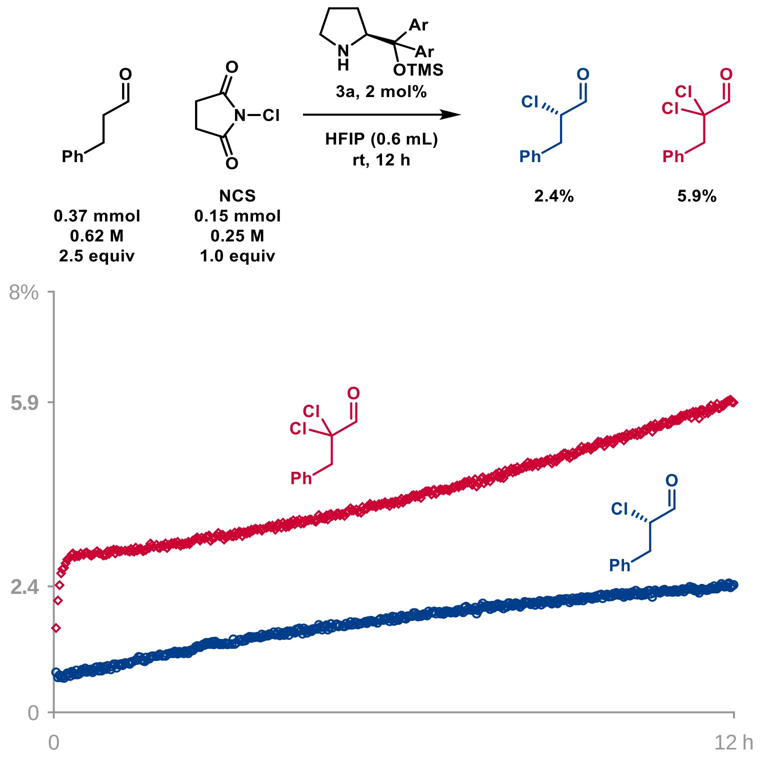

Figure 11 – In the absence of added water, the reaction demonstrated poor yield with a majority of dichlorinated product.

The overall consumption of NCS was 14.2% (=5.9*2+2.4). The majority of product (71% of the total product) was dichlorinated. The rate of formation of dichlorinated product does not correlate with the amount of monochlorinated product, which suggested that the majority of dichlorinated product is formed from the chlorination of a catalytic intermediate.

6. Effect of water on the percentage of dichlorinated product

To minimize the formation of undesired dichlorinated product, water was added to the reaction. The purpose of this addition was to hydrolyze the monochlorinated iminium ion before its corresponding enamine undergoes a second chlorination. Two reactions were run with different initial concentrations of water and identical concentrations of all other species. These reactions were prepared from the following common stock solutions in HFIP.

SL	Component	Volume / mL	Mass / mg	Concentration / M
**A**	Hydrocinnamaldehyde	1.0	252.9	1.88
**B**	NCS	1.0	201.4	1.51
**C**	cat **3a**	1.0	35.9	0.06
**D**	H_2O	1.0	120.3	6.68
**E**	H_2O	1.0	602.1	33.45

6.1. Reaction with $[H_2O]_o = 2.22$ M

Stock solutions **A** (250 µL, 0.47 mmol, 2.5 equiv), **B** (100 µL, 0.15 mmol, 1.0 equiv) and **D** (200 µL, 1.34 mmol) were added to an NMR tube and placed into the spectrometer to pre-shim. Stock solution **C** (50 µL, 3.0 µmol, 2 mol%) was added to the tube, which was shimmed as rapidly as possible. A series of consecutive ^{1}H NMR experiments (16 scans) with a duration of 90 s each were run for 12 h. Characteristic aldehyde (9.71, t, starting material; 9.51 ppm, d, monochlorinated product; 9.33 ppm, s, dichlorinated product) peaks were integrated to approximate the conversion.

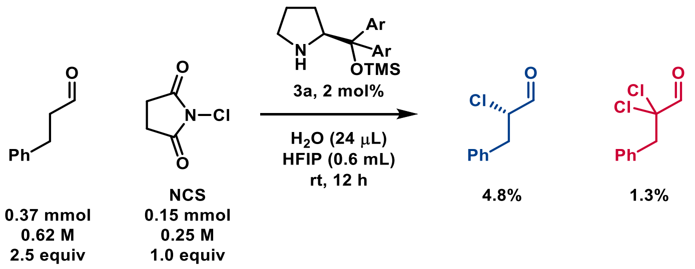

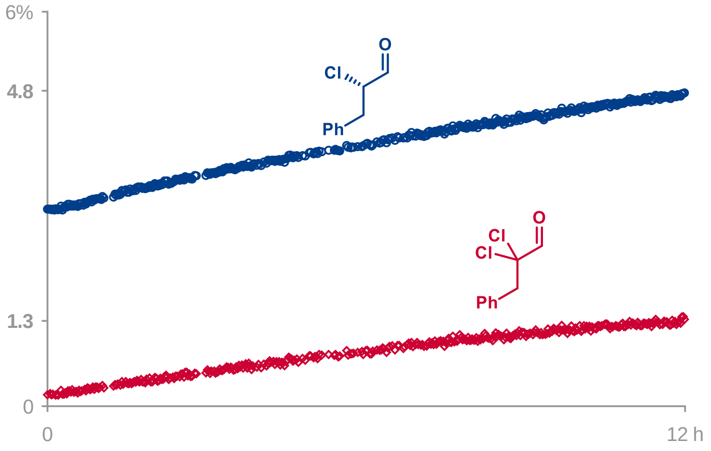

Figure 12 – Water increased the yield of monochlorinated product but reduced the overall conversion.

These conditions significantly reduced the percentage of product that was dichlorinated from 71% (without added water) to 21% ($[H_2O]_o = 2.22$ M), though the dichlorination was not eliminated entirely. The consumption of NCS (7.4%) and formation of monochlorinated product (4.8%) were still low.

6.2. Reaction with $[H_2O]_o$ = 11.15 M

Stock solutions **A** (250 µL, 0.47 mmol, 2.5 equiv), **B** (100 µL, 0.15 mmol, 1.0 equiv) and **E** (200 µL, 6.69 mmol) were added to an NMR tube and placed into the spectrometer to pre-shim. Stock solution **C** (50 µL, 3.0 µmol, 2 mol%) was added to the tube, which was placed into the spectrometer and shimmed as rapidly as possible. A series of consecutive ^{1}H NMR experiments (16 scans) with a duration of 90 s each were run for 12 h. Characteristic aldehyde peaks (9.71, t, starting material; 9.51 ppm, d, monochlorinated product; 9.33 ppm, s, dichlorinated product) were integrated to approximate the conversion for this reaction.

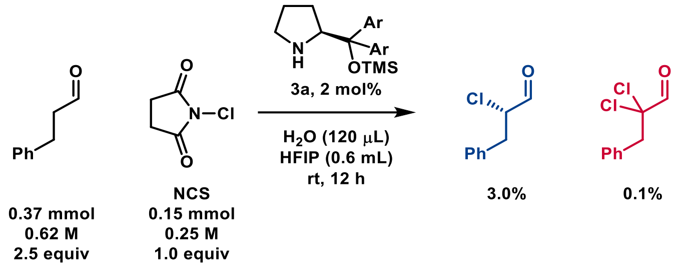

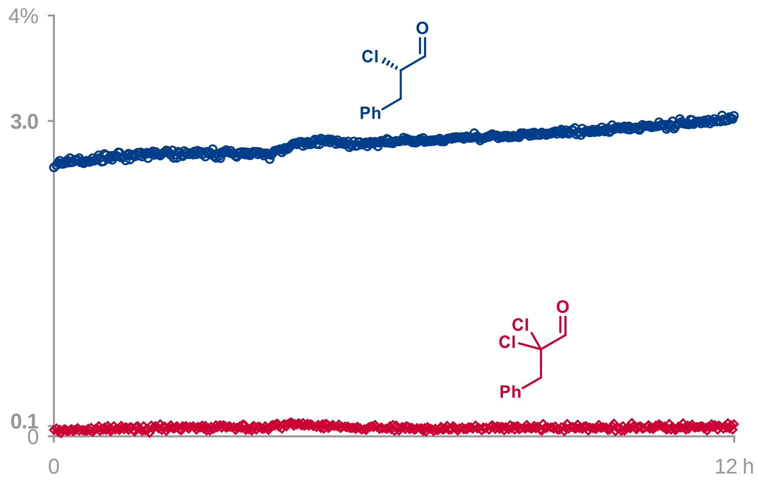

Figure 13 – High initial concentration of water (11.15 M) almost completely eliminated the formation of dichlorinated product.

This experiment showed minimal formation of dichlorinated product (0.1%, 3% of the total product) over 12 h, but also displayed the lowest consumption of NCS (3.2%) of all the series of experiments with different initial concentrations of water. The formation of monochlorinated product was also low (3.0%).

6.3. *Study of the effect of water on the equilibrium of iminium ion formation*

While the addition of water mitigated the formation of dichlorinated product, it also drastically reduced the yield. We proposed that the deactivation process observed in previous reaction was increased due to the shift of the equilibrium of iminium ion formation back to free catalyst by increased concentrations of water. To test this hypothesis, eight NMR tubes for each of catalysts **3a** and **3c** were prepared with different initial concentrations of water. Characteristic TMS peaks were integrated to determine the ratio of free catalyst versus iminium ion. The following stock solutions in HFIP were used.

SL	Component	Volume / mL	Mass / mg	Concentration / M
**A**	Hydrocinnamaldehyde	2.0	1004.2	3.74
**B**	cat **3c**	1.0	18.2	0.03
**C**	cat **3a**	1.0	9.8	0.03

Stock solutions **A** (100 µL, 0.37 mmol, 1.0 equiv) and **B** (100 µL, 3 µmol) or **C** (100 µL, 3 µmol) were added to each tube. The desired quantity of water was added using a micropipette and the total volume was made up to 500 µL with HFIP.

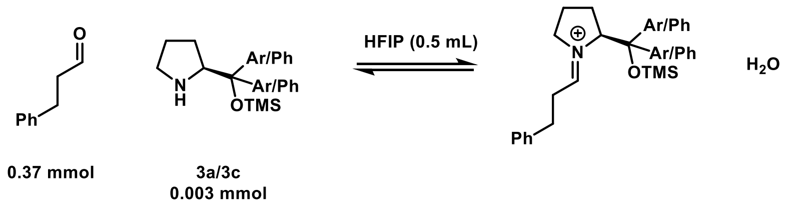

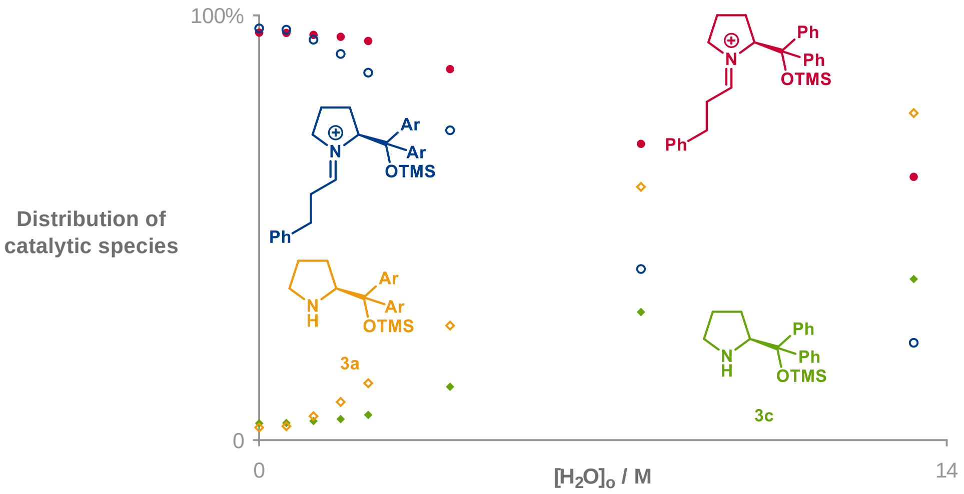

Figure 14 – Increasing the amount of water reduced the proportion of iminium ion in equilibrium.

A higher concentration of water reduced the amount of iminium ion present for both catalysts, though the equilibrium for catalyst **3a** was further shifted to free catalyst, under the same initial concentrations of water, than catalyst **3c**. Simplistically, these equilibriums suggest that the diphenyl Jørgensen-Hayashi type catalysts (**3c** and **3d**) may be less prone to deactivation pathways involving the free catalyst than the analogous bis-trifluoromethylphenyl catalysts (**3a** and **3b**).

7. Study of the deactivation of the aminocatalyst by NCS

We suggested that the deactivation of the catalyst was a result of direct reaction with the chlorinating agent. To test this hypothesis, Jørgensen-Hayashi type catalysts **3a**, **3b**, **3c** and **3d** (0.03 mmol) were individually mixed with NCS (8.0 mg, 0.06 mmol) in HFIP (0.5 mL total volume). In order to follow the reaction, ^{1}H NMR spectra were recorded at the indicated time intervals.

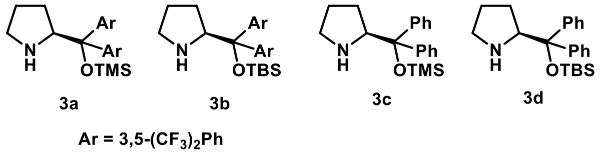

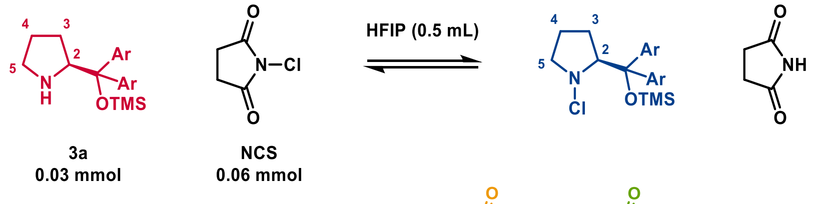

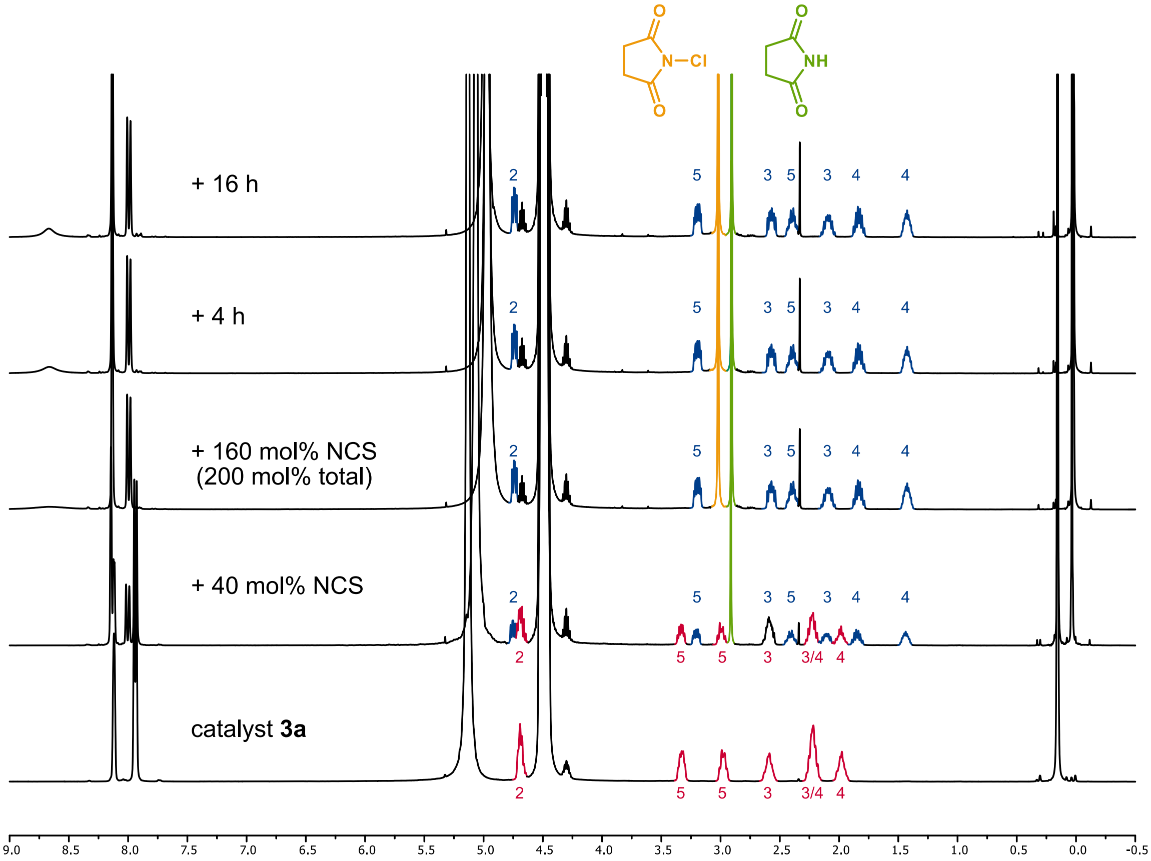

*Figure 15 – Chlorinated and free amine are the only two species observed during the reaction of catalyst **3a** with NCS.*

For catalyst **3a**, two species were observed over the 16 h period, free catalyst and chlorinated catalyst. Key peaks corresponding to each species were identified. The lack of new peaks over time indicated that the catalyst does not undergo further decomposition.

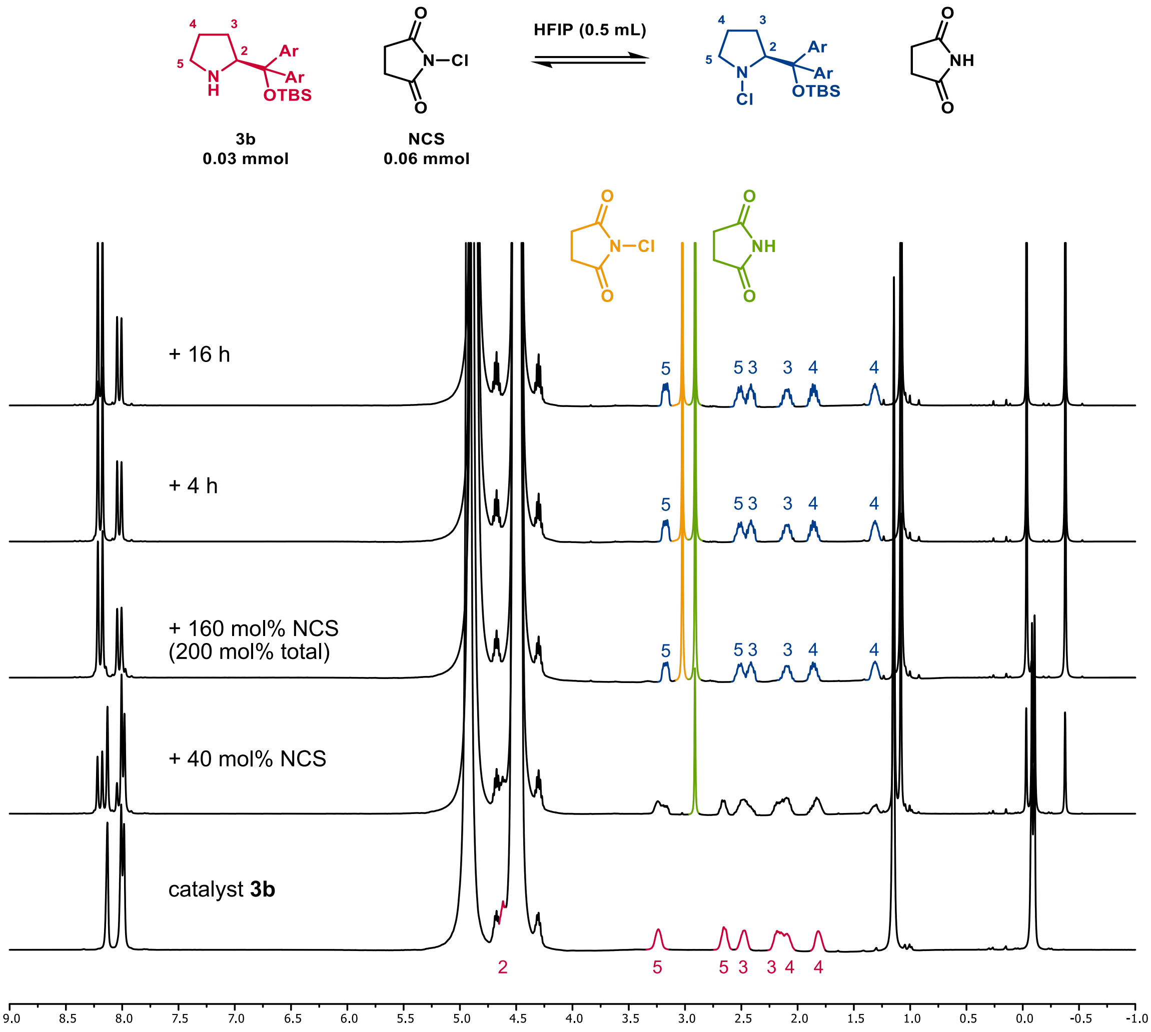

*Figure 16 – Chlorinated and free amine are the only two species observed during the reaction of catalyst **3b** with NCS.*

In a similar manner to **3a**, catalyst **3b** displayed two clear species, assigned as free catalyst and chlorinated catalyst. No decomposition of the chlorinated catalyst was observed.

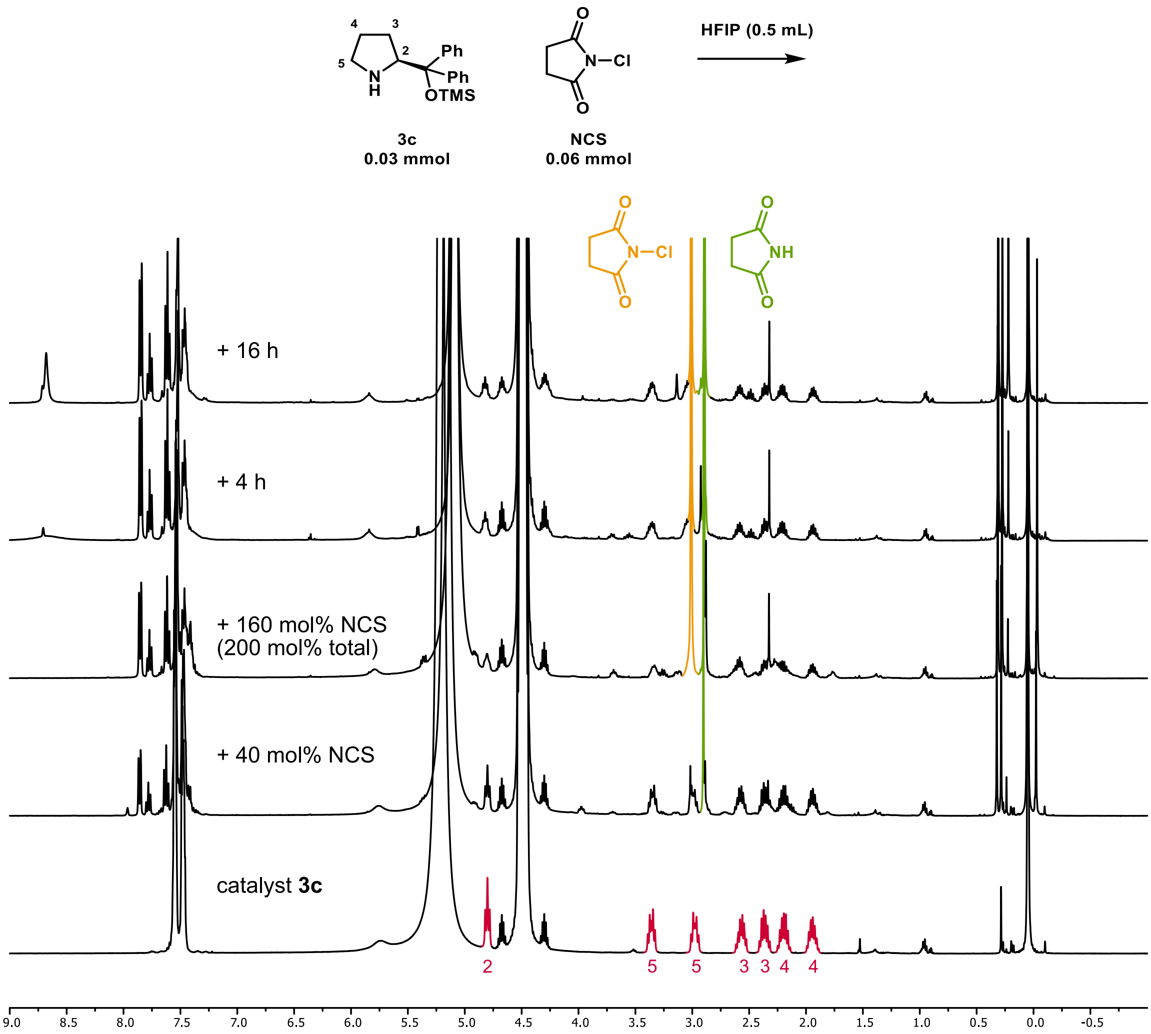

*Figure 17 – Multiple species are observed during the reaction of catalyst **3c** with NCS.*

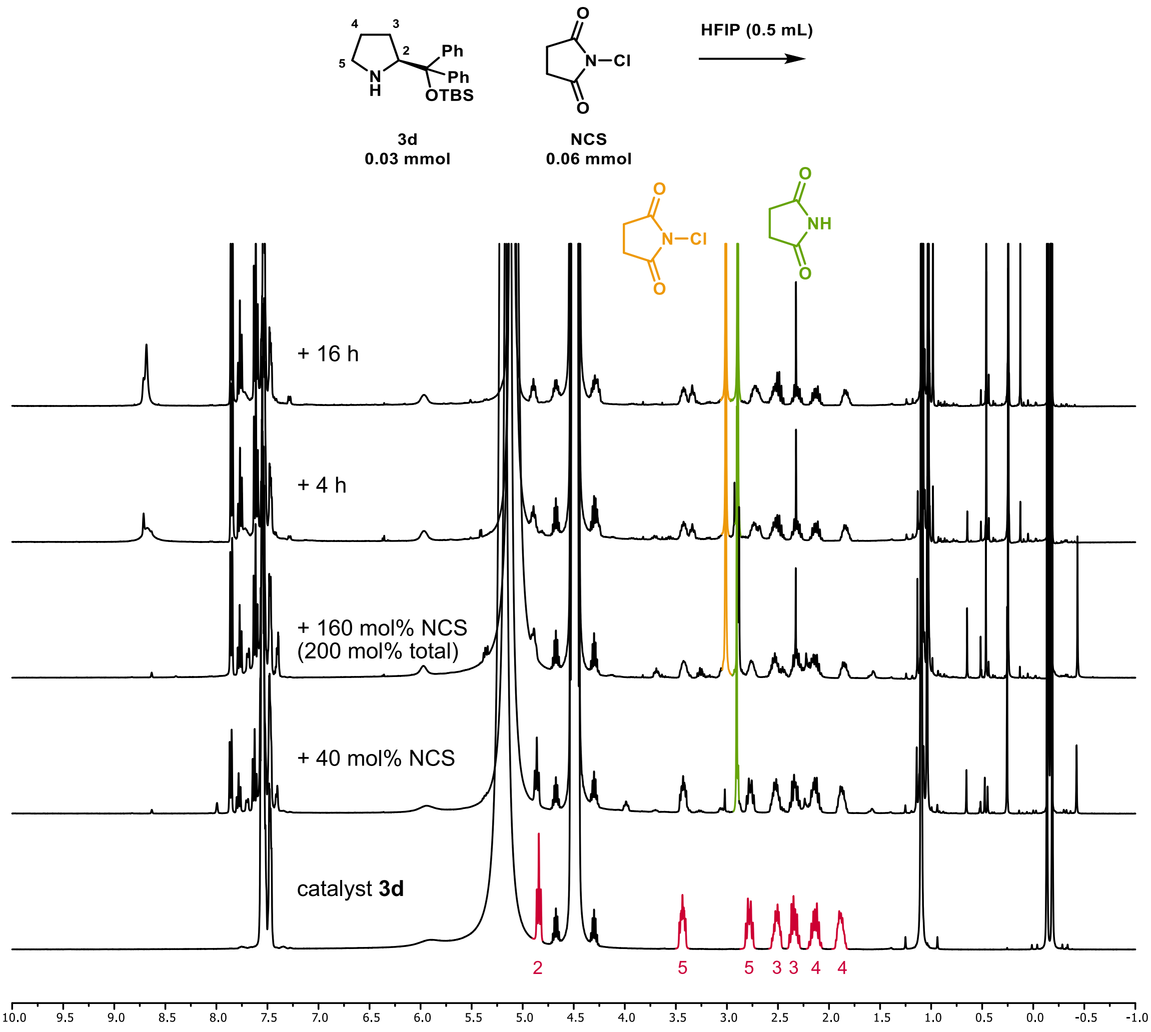

*Figure 18 – Multiple species are observed when catalyst **3d** was mixed with NCS in HFIP.*

For both catalysts **3c** and **3d**, the result of the reactions with NCS were less clear, with multiple TMS/TBS species observed. Preparative TLC of these crude reaction mixtures resulted in the identification of benzophenone (R_f = 0.51, CH_2Cl_2), a species indicative of catalyst decomposition.

*7.1. Reaction of catalyst **3c** with NCS in HFIP*

During the reaction of catalysts **3c** and **3d** with NCS, we identified benzophenone as a byproduct. The appearance of benzophenone suggested a Grob-type fragmentation. To further investigate this hypothesis, we weighed catalyst **3c** (40.0 mg, 0.12 mmol) into an NMR tube and added HFIP (0.6 mL). Solid NCS (24.0 mg, 0.18 mmol) was added to initiate the reaction and the sample was placed in the spectrometer, with ^{1}H NMR spectra recorded continuously. After the catalyst decomposition was deemed to have stopped, Cs_2CO_3 (16.3 mg, 0.05 mmol) was added to probe the suggestion that one of the observed species was protonated catalyst.

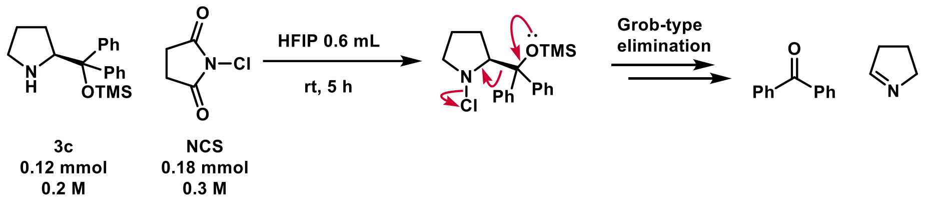

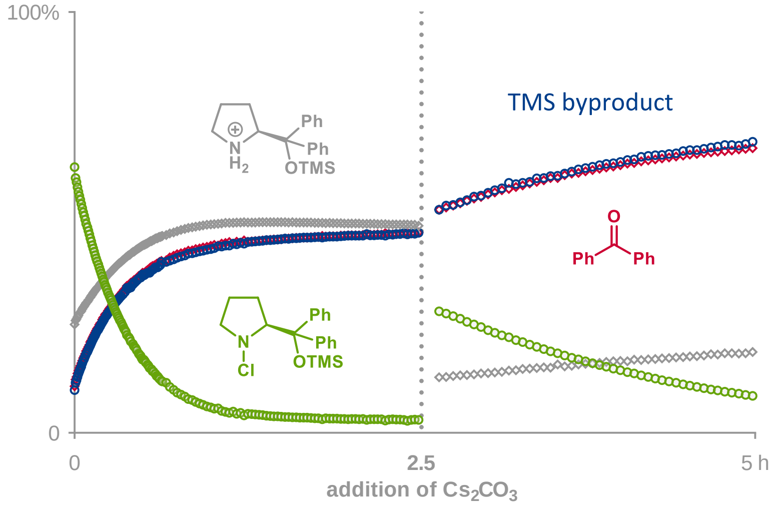

Figure 19 – The concentration of new TMS-containing species closely matches the concentration of benzophenone. Additionally, the decomposition reaction is restarted by base addition.

The addition of Cs_2CO_3 neutralized the acidic media and restarted the reaction. This neutralization freed up the previously protonated catalyst and allowed it to undergo chlorination and subsequent decomposition. The consumption of NCS matched the appearance of chlorinated catalyst, which indicated that the protonated species was not yet chlorinated. Plausibly, 1,1,1,3,3,3-hexafluoro-2-trimethylsiloxypropane is formed during the reaction, consistent with Röschenthaler's formation of the same species from HFIP and trimethylsilyl chloride.[8]

7.2. Study of the reversibility of the chlorination reaction of the aminocatalyst

As catalyst **3a** did not undergo irreversible decomposition when chlorinated, we decided it was worthwhile to study the potential reversibility of the chlorination reaction. If the chlorination reaction was reversible, the addition of succinimide would shift the catalyst chlorination equilibrium towards free catalyst and increase the rate of reaction. The following stock solutions in HFIP were used for these reactions.

SL	Component	Volume / mL	Mass / mg	Concentration / M
**A**	Hydrocinnamaldehyde	1.0	203.0	1.51
**B**	NCS	1.0	79.2	0.59
**C**	cat **3a**	1.0	18.8	0.03

Two NMR tubes, one with added succinimide (14.7 mg, 0.15 mmol, 1.0 equiv), and the other without, were each injected with the stock solutions **A** (250 µL, 0.38 mmol, 2.5 equiv), **B** (250 µL, 0.15 mmol, 1.0 equiv) and **C** (100 µL, 3.0 µmol, 2 mol%). The samples were placed in an NMR spectrometer and ^{1}H NMR experiments, of duration 90 s, were run consecutively for a period of 10 h. Characteristic aldehyde peaks (9.71, t, starting material; 9.51 ppm, d, monochlorinated product; 9.33 ppm, s, dichlorinated product) were integrated to approximate the conversion for this reaction.

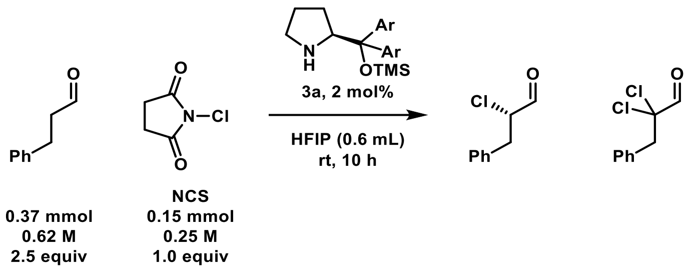

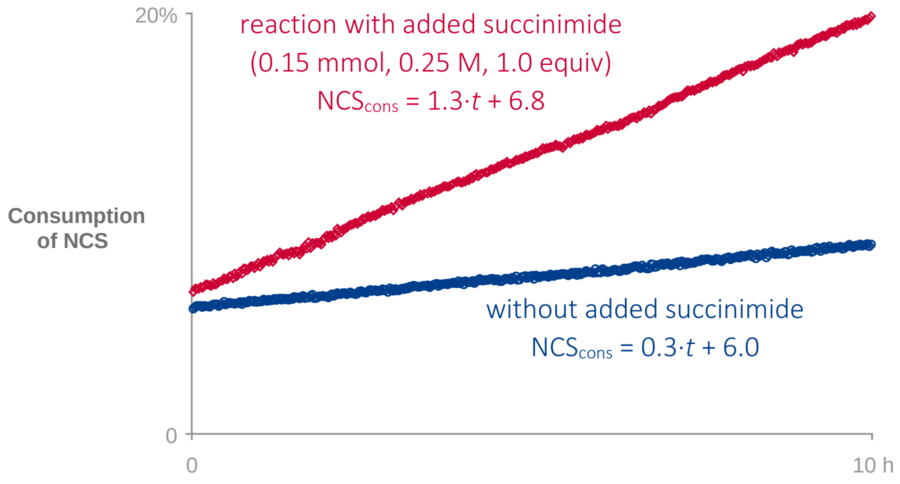

Figure 20 – The reaction with added succinimide is quicker than the reaction without.

Figure 21 shows that the formation of product, once the equilibrium between free and chlorinated catalyst has been established, is 4.3x faster for the reaction with added succinimide. In a further experiment, hydrocinnamaldehyde (20.1 mg, 0.15 mmol) was added to a solution of pre-chlorinated catalyst (0.03 mmol), NCS (4.0 mg, 0.03 mmol) and succinimide (3.0 mg, 0.03 mmol). Continuous ^{1}H NMR spectroscopy indicated reaction progress that is quenched by addition of further NCS (16.0 mg, 0.12 mmol).

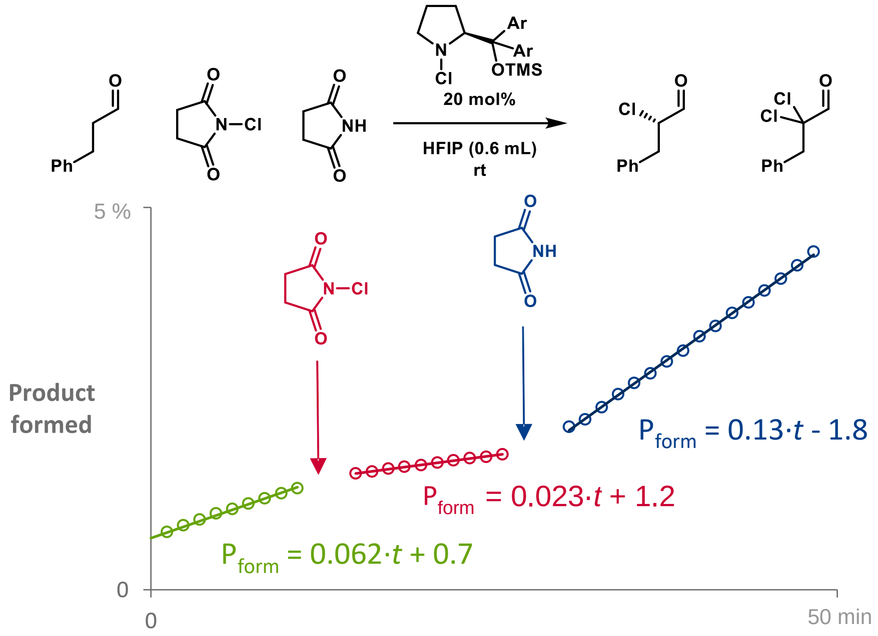

Figure 21 – The rate of reaction is dependent on the concentrations of both NCS and NHS.

The subsequent addition of succinimide (11.9 mg, 0.12 mmol) enhanced the rate of reaction to double that of the original rate. This result confirmed the reversibility of the catalyst chlorination step.

8. Aminocatalytic α-chlorination with slow addition of the chlorinating agent

The addition of succinimide accelerated the reaction by shifting the equilibrium of the catalyst chlorination step towards the active catalyst, however, the reaction is still too slow to be practical. We proposed that the slow addition of chlorinating agent would be a better alternative. The low concentration of chlorinating agent would disfavor the formation of chlorinated catalyst. We demonstrated that the slow addition of chlorinating agent presented a second advantage; a lower amount of water was required to reduce dichlorination to an acceptable level when compared to experiments where all the NCS was added in an instantaneous injection.

We used an in-situ FTIR probe (ReactIR 15) to monitor the progress of the reaction. We recorded the peaks in the IR spectra corresponding to NCS and succinimide over time. The conditions at the end of the reactions run with slow additions (Sections 8.2 and 8.3) were comparable to the conditions of the reaction run without slow addition (Section 8.1).

SL	Component	Volume / mL	Mass / mg	Concentration / M
**A**	Hydrocinnamaldehyde	2.0	1011.2	3.77
**B**	NCS	2.0	403.7	1.51
**C**	cat **3a**	2.0	36.3	0.03
**D**	H_2O	2.0	481.5	13.37
**E**	Succinimide	1.0	145.8	1.47

For each of the two addition rates below, a vial containing HFIP (500 µL), a magnetic stirrer and fitted with the ReactIR probe was successively charged with stock solutions **A** (500 µL, 1.89 mmol, 2.5 equiv), **C** (500 µL, 0.015 mmol, 2 mol%) and **D** (500 µL, 6.69 mmol). We collected 5 FTIR spectra (of 41 or 82 scans each, dependent on the time of addition) before starting the addition of NCS. We added 500 µL of stock solution **B** (0.75 mmol, 1.0 equiv) with a syringe pump at the indicated rates. Once the addition had ended and a further 4 points were collected, 250 µL of stock solution **E** (0.37 mmol or 500 µL, 0.75 mmol for the reaction where the NCS was added in a single injection) was added to calibrate the FTIR data. The reaction mixture was quenched in a stirred solution of $NaBH_4$ (750 mg, 19.8 mmol) in MeOH (5 mL). After stirring for 5 min, brine (5 mL) and H_2O (5 mL) were added. The mixture was extracted with CH_2Cl_2 (4 x 15 mL), before the collected organic phase was washed with brine (15 mL), dried over $MgSO_4$ and concentrated on a rotary evaporator.

8.1. NCS added in an instantaneous injection

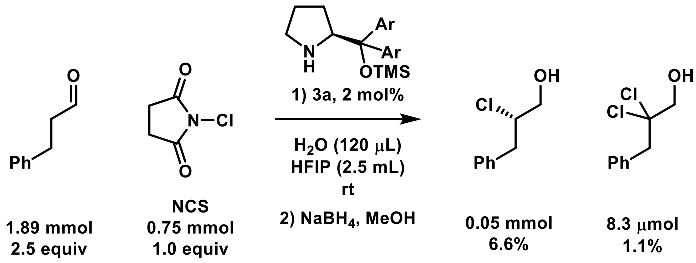

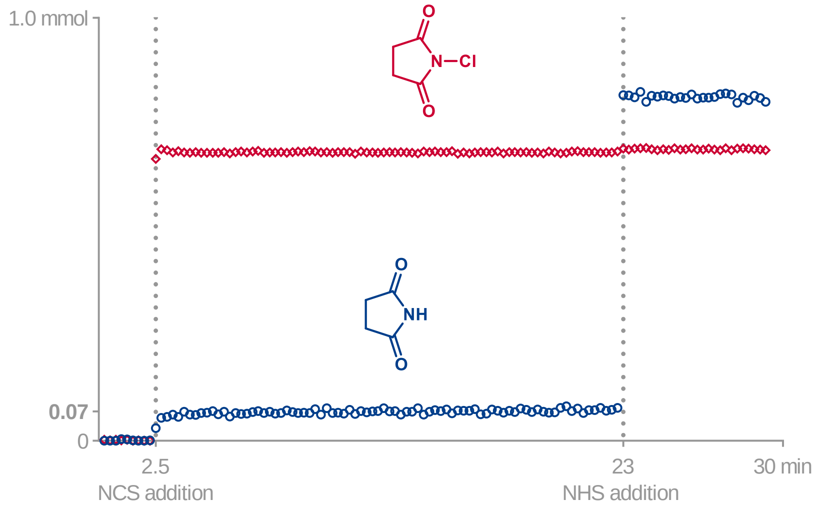

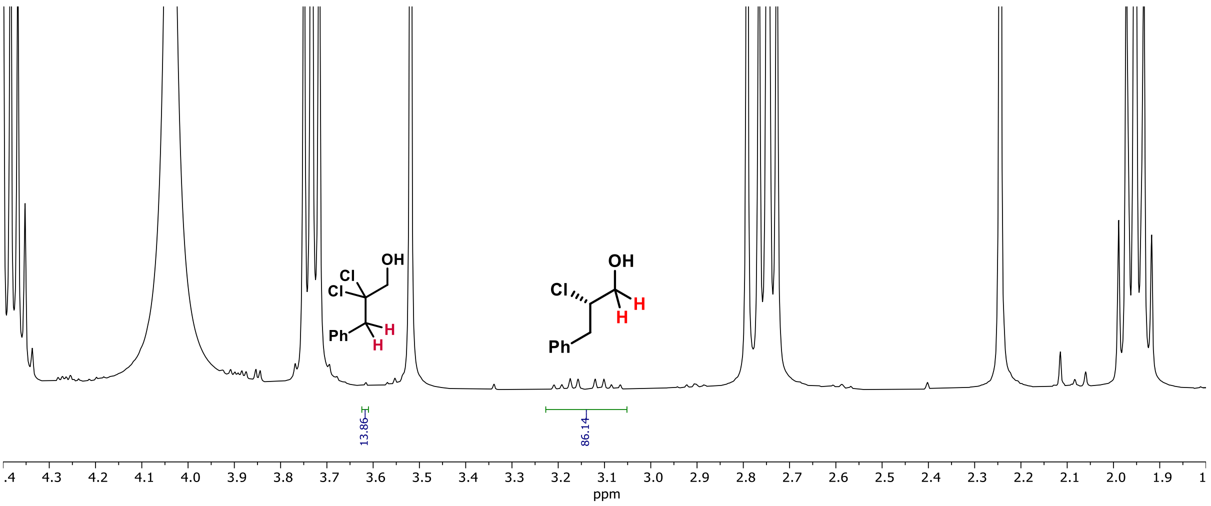

Figure 22. The reaction yield is poor when the chlorinating agent is added in a single injection.

The observable jump in amount of succinimide at the end of the reaction profile (23 min) is due to the standard addition of stock solution **E** (see Section 11). The FTIR data indicated that a small amount of the chlorinating agent was consumed (0.07 mmol). The crude NMR, taken post-reduction, indicated that the ratio of mono to dichlorinated product was 86:14. As each molecule of dichlorinated product accounts for two molecules of NCS, the monochlorinated product accounts for 75% (F_{mono}) of the NCS consumed.

$$\text{Yield} = 100\frac{\text{NCS}_{\text{consumed}} \cdot \text{F}_{\text{mono}}}{\text{NCS}_{\text{added}}} = 100\frac{0.07 \cdot 0.75}{0.75} = 7\%$$

8.2. Rate of addition of NCS = 0.1875 mmol/min

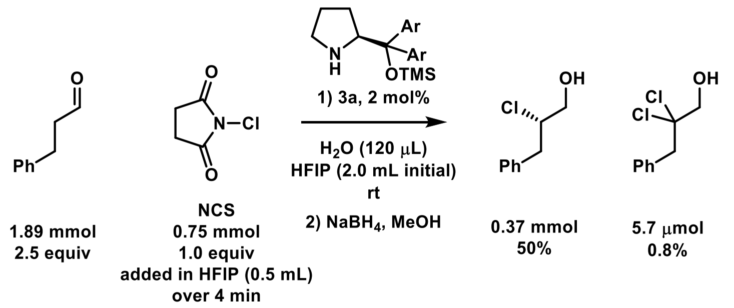

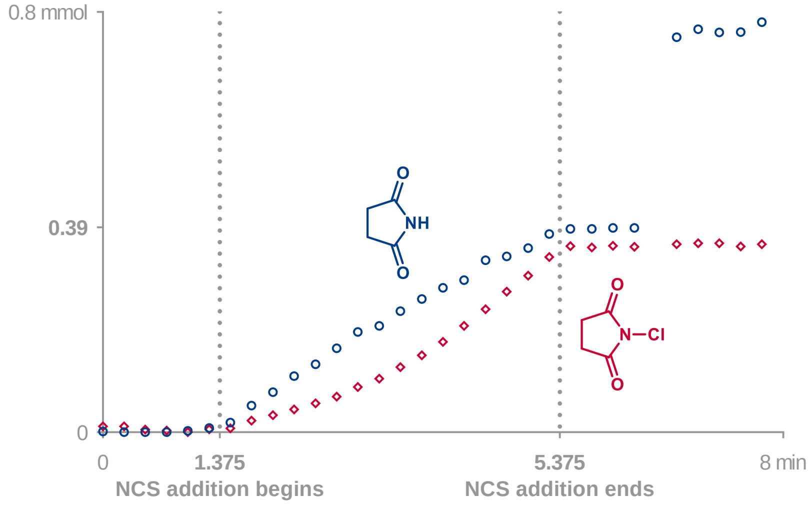

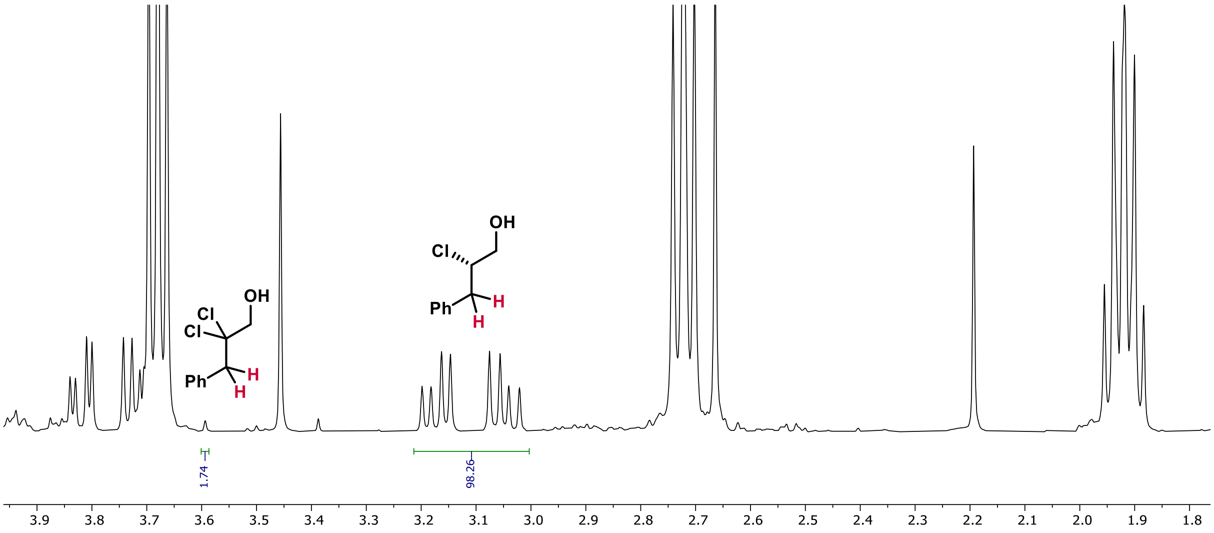

Figure 23 – Yield was improved by the slow addition of NCS, but addition was still too fast.

The FTIR data indicated that some of the chlorinating agent was consumed (0.39 mmol). The crude NMR, taken post-reduction, indicated that the ratio of mono to dichlorinated product was 98:2. As each molecule of dichlorinated product accounts for two molecules of NCS, the monochlorinated product accounts for 96% (F_{mono}) of the NCS consumed.

$$\text{Yield} = 100\frac{\text{NCS}_{\text{consumed}} \cdot \text{F}_{\text{mono}}}{\text{NCS}_{\text{added}}} = 100\frac{0.39 \cdot 0.96}{0.75} = 50\%$$

8.3. Rate of addition = 0.0395 mmol/min

We used a slower addition rate to achieve complete consumption of NCS. The calibrated FTIR data (82 scans) shows a perfect match between the amount of added NCS and the amount of formed succinimide. No build-up of NCS was observed.

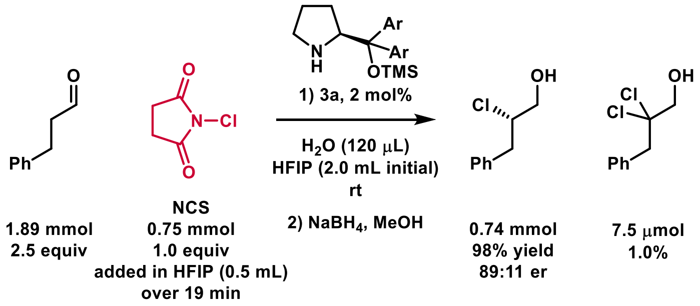

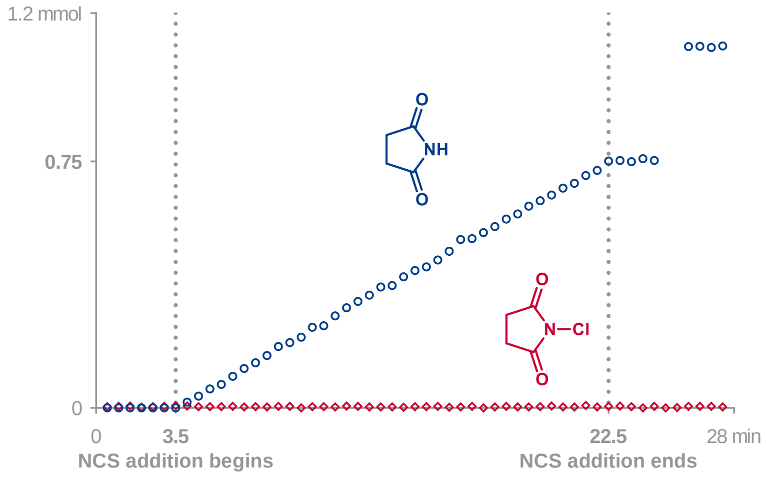

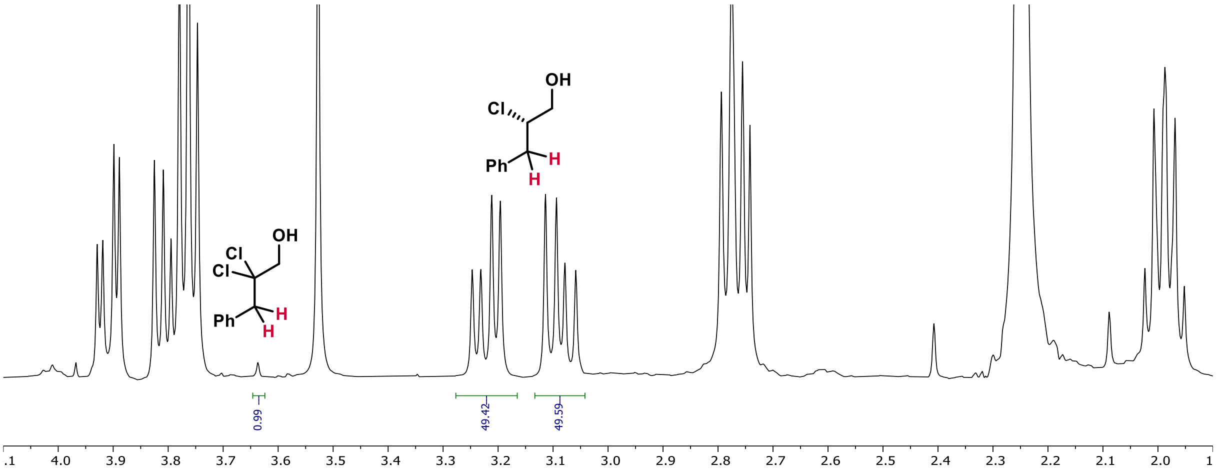

Figure 24 – A further reduced rate of additon leads to complete consumption of NCS and an excellent yield.

The FTIR data indicated that all of the chlorinating agent was consumed (0.75 mmol). The crude NMR, taken post-reduction, indicated that the ratio of mono to dichlorinated product was 99:1. As each molecule of dichlorinated product accounts for two molecules of NCS, the monochlorinated product accounts for 98% (F_{mono}) of the NCS consumed.

$$\text{Yield} = 100\,\frac{\text{NCS}_{\text{consumed}} \cdot \text{F}_{\text{mono}}}{\text{NCS}_{\text{added}}} = 100\,\frac{0.75 \cdot 0.98}{0.75} = 98\%$$

8.4. Simulated kinetic reaction profile of the slow addition of NCS

The catalytic cycle shown below was input into COPASI,[9] a kinetic modelling software, as a series of reaction steps, reversible or irreversible as indicated. Initial concentrations were included and matched those used in the slow additions above. The slow addition was modelled as a constant flux of NCS over the experiment time, with an accompanying increase in the container volume.

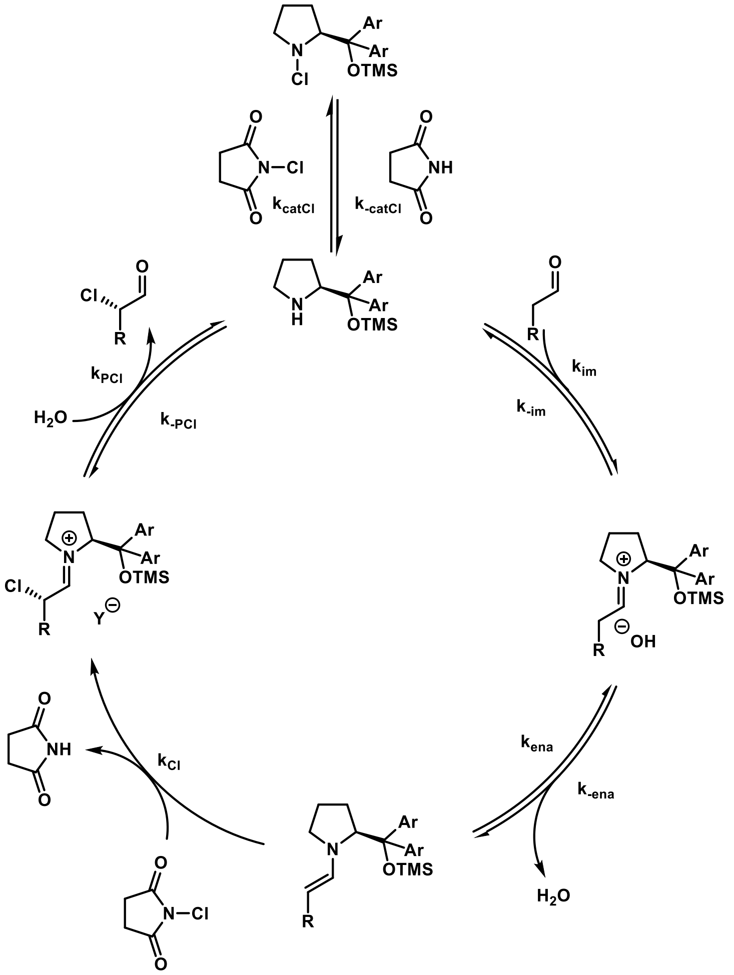

$[CHO]_o$	0.9375 M
$[H_2O]_o$	3.3333 M
$[Cat]_o$	0.0075 M

Figure 25 – Catalytic cycle and initial concentrations used in COPASI simulation.

The FTIR data from the reactions performed with two different rates of dosing NCS was used to perform the parameter estimation task in COPASI. We allowed the program to estimate the kinetic constants shown in the catalytic cycle (Figure 25). The simulated data generated from these estimated constants matched the experimental data, which confirms that the proposed catalytic cycle is plausible. However, given that this simulation involved estimating 9 variables with 2 data sets, the coefficients generated are not necessarily representative of the real values.

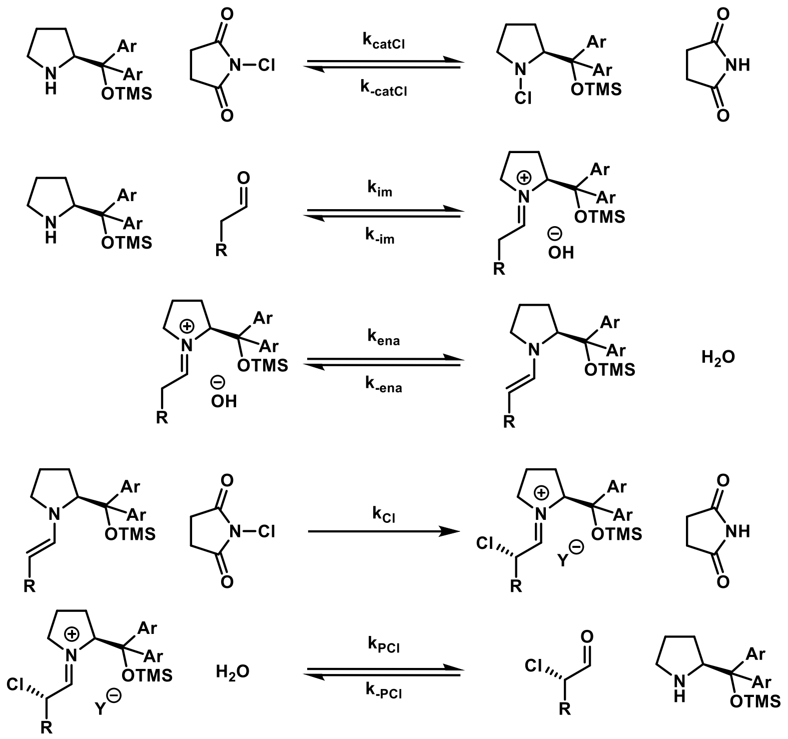

Parameter	Value estimated by COPASI
k_{catCl}	0.03466
k_{-catCl}	4.165×10^{-5}
k_{im}	0.1628
k_{-im}	0.02291
k_{ena}	1.997
k_{-ena}	1.067×10^{-5}
k_{Cl}	556.9
k_{PCl}	8.017×10^{5}
k_{-PCl}	0.01857

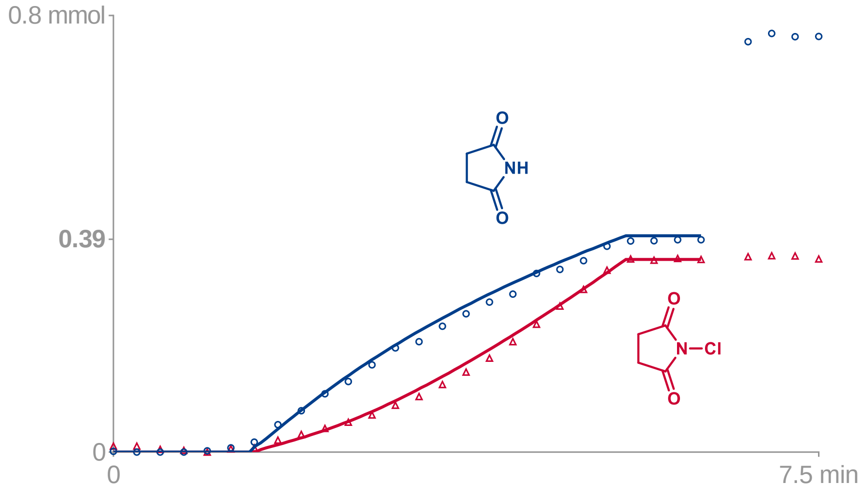

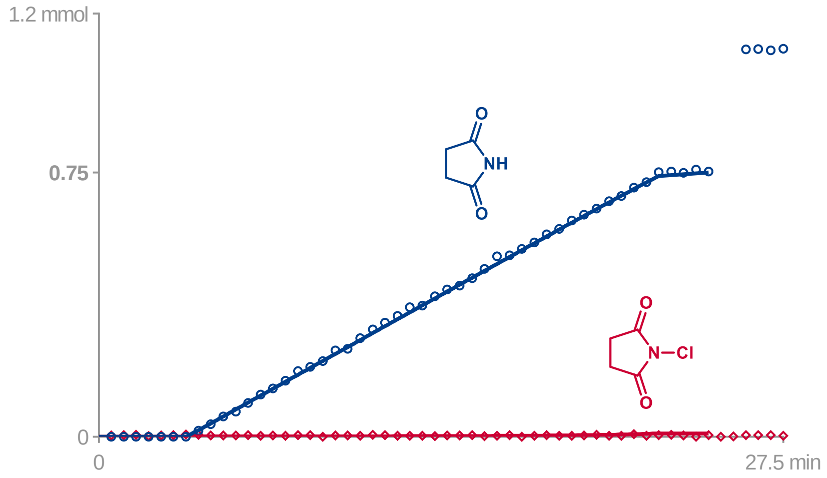

Figure 26 – Good agreement between simulated (lines) and experimental (points) data.

9. General method to determine the optimal rate of addition of chlorinating agent and the amount of added water

Optimization of the rate of addition and amount of water can be carried out easily and with few experiments, using only the crude ^{1}H NMR post reduction and without the need for continuous FTIR data. The process can be considered as two stages, firstly to optimize yield and then optimize enantiomeric excess. Initial conditions should be selected from the most similar aldehyde.

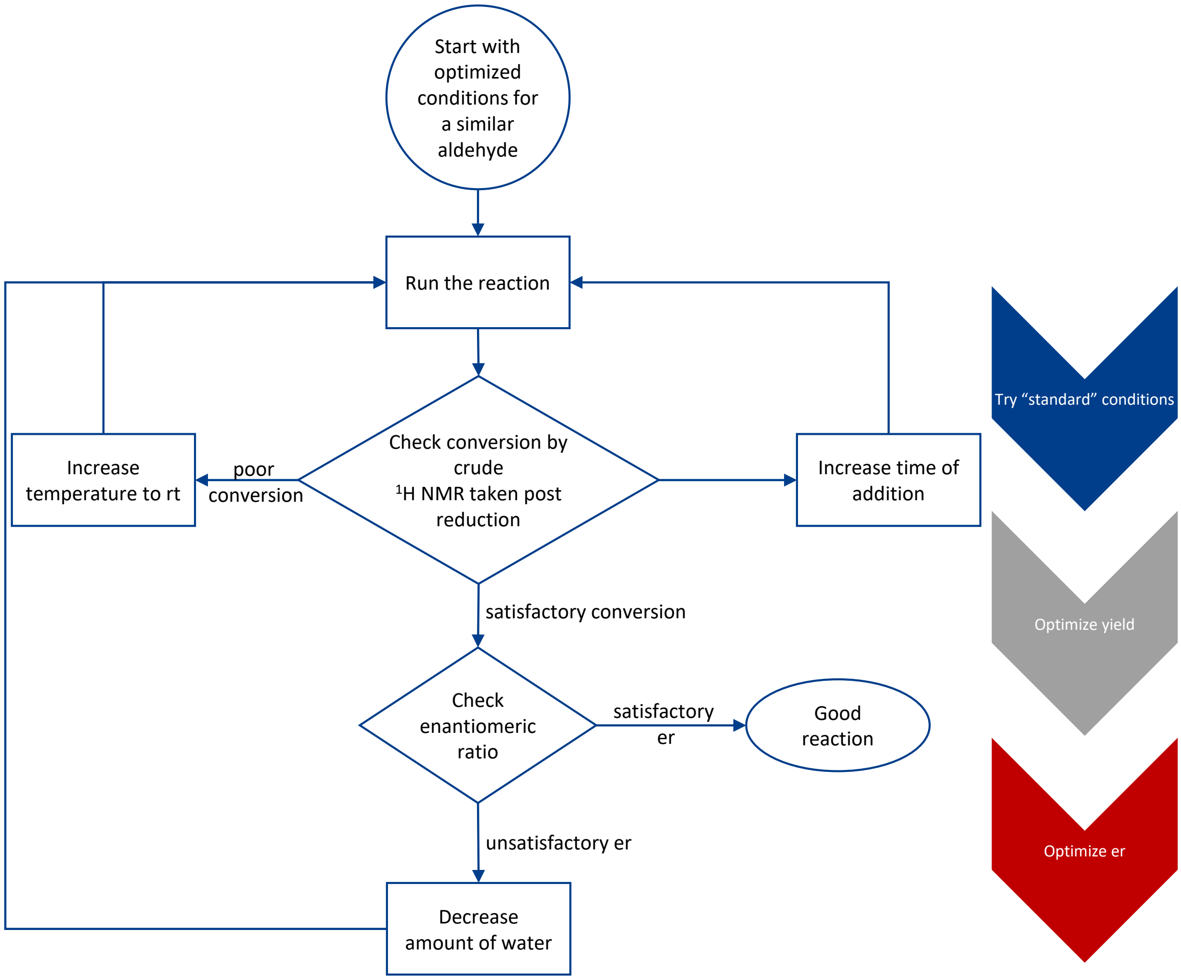

Time of addition is optimized first, because the amount of added water minimally affects the required time of addition. The time of addition is increased until all chlorinating agent is deemed consumed. If there is a significant excess of aldehyde, a slightly too slow addition will not significantly racemize the product. However, in the case of aldehydes with β-substitution or aldehydes in equilibrium, such as lactols, increasing the time by periods by small increments will not significantly improve yields. In these cases, carrying out the reaction at room temperature is advised, or with larger increments of addition-time increase, as necessary.

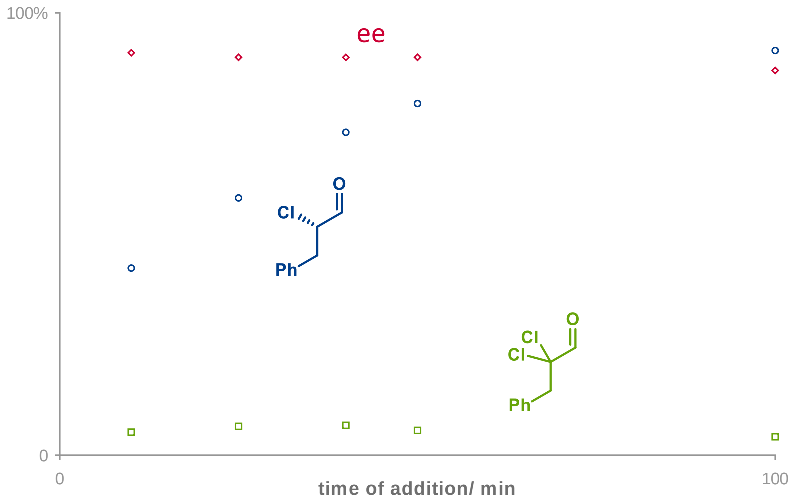

Figure 27 – The ee of the product remains constant despite increases to the time of addition, though the yield and conversion increase.

The mono:dichlorinated ratio is determined by integration of characteristic peaks in the ^{1}H NMR taken post reduction. Increasing the initial concentration of water increases the monochlorinated product relative to the dichlorinated but also reduces the ee of the product. Conversely, if a higher ee is desired, the initial concentration of water should be reduced to increase ee via a partial resolution through dichlorination.

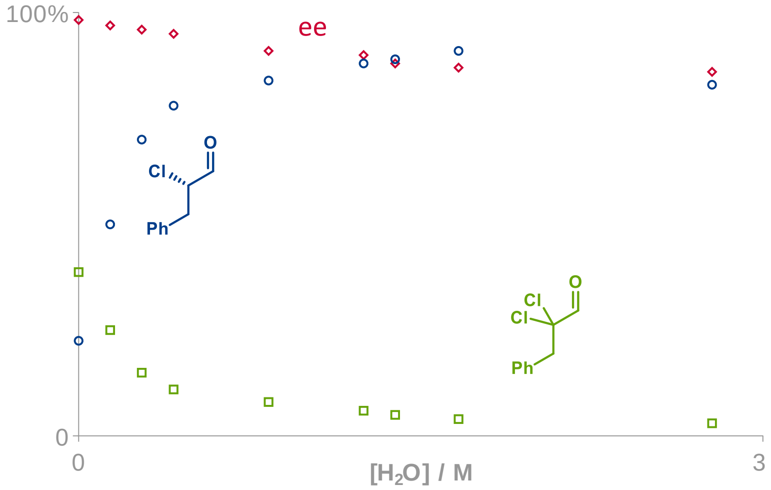

Figure 28 – The ee of the product decreases as the proportion of dichlorinated product is reduced by increasing $[H_2O]_o$.

10. Showcase aminocatalytic α-chlorination of aldehyde reactions in HFIP

The enhanced understanding from the previous experiments allowed us to achieve excellent yields and enantioselectivities for the aminocatalytic α-chlorination of aldehydes under a broad range of practical conditions. The aim was to provide optimized conditions based on a particular need, be it high er, low catalyst loading or using aldehyde as the limiting reagent. The results are summarized below, with full details of experimental procedures in the following sections.

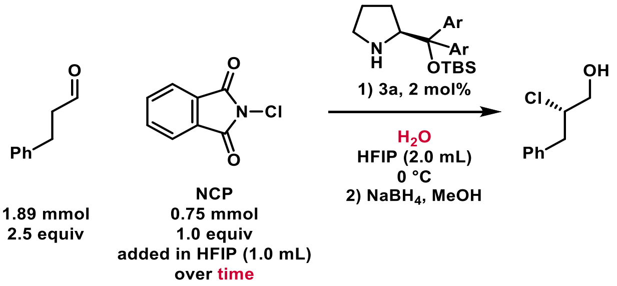

Deviation from above	H_2O / µL	Time of addition / min	Yield / %[a]	Dichlorination / %[a]	er
none	35	60	85	7.5	99:1
NCS instead of NCP	35	50	84	8.1	97:3
cat **3a** instead of cat **3b**	10	60	70	15	98:2
1 mol% of cat **3b**[b]	35	150	85	4.7	98:2
room temperature	30	20	91	4.7	97:3
0.76 mmol of hydrocinnamaldehyde[c]	40	150	68	10	97:3
octanal	65	60	76	12	99:1
isovaleraldehyde[d]	48	25	80	9.9	99:1
propanal	100	75	78	8.4	98:2
δ-valerolactol[e]	20	1440	68	--[f]	99:1
pentanal	70	75	66	16	99:1
5-bromopentanal	70	75	80	9.2	98:2
dodecanal	75	60	77	12	99:1

[a]Calculated with respect to the limiting reagent. [b]0.76 mmol of phthalimide added before the beginning of the reaction. [c]0.90 mmol (1.2 equiv) of NCP infused in 1.2 mL HFIP. [d]Reaction run at room temperature and 80% of the normal scale. [e]5 mol% of catalyst was used and the reaction was run at 8 °C. [f]Not quantified.

10.1. Standard reaction conditions

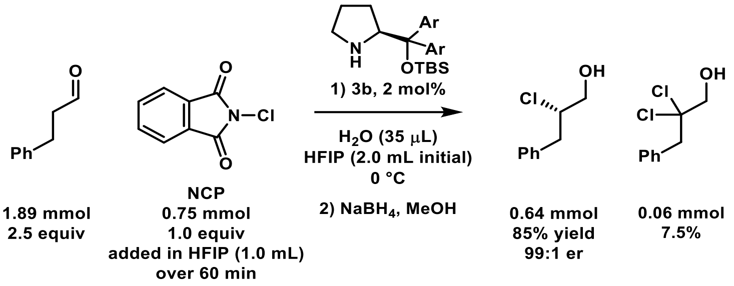

Stock solutions, made up to the prescribed volume with HFIP, were used in the preparation of this reaction.

SL	Component	Volume / mL	Mass / mg	Concentration / M
**A**	Hydrocinnamaldehyde	1.0	507.6	3.78
**B**	NCP	2.0	273.1	0.75
**C**	cat **3b**	1.0	19.2	0.03
**D**	H_2O	1.0	69.8	3.88
**E**	Phthalimide	1.0	104.5	0.71

A vial containing 500 µL of HFIP, a magnetic stirrer and fitted with the ReactIR probe was charged with stock solutions **A** (500 µL, 1.89 mmol, 2.5 equiv), **C** (500 µL, 0.015 mmol, 2 mol%) and **D** (500 µL, 1.94 mmol). The reaction mixture was cooled to 0 °C using an ice bath, stirred for 8 min and consecutive FTIR spectra of 122 scans were taken with the ReactIR. Stock solution **B** (1.0 mL, 0.75 mmol, 1.0 equiv) was added using a syringe pump over 60 min. After the addition ended, a further 4 spectra were collected before stock solution **E** (500 µL, 0.36 mmol, 0.5 equiv) was added for calibration. A final 5 spectra were collected, before the reaction mixture was quenched in a stirred solution of $NaBH_4$ (750 mg, 19.8 mmol) in MeOH (5 mL). After stirring for 5 min, brine (5 mL) and H_2O (5 mL) were added. The mixture was extracted with CH_2Cl_2 (4 x 15 mL), before the collected organic phase was washed with brine (15 mL), dried over $MgSO_4$ and concentrated on a rotary evaporator. The product, 2-chloro-3-phenylpropan-1-ol (R_f = 0.38, CH_2Cl_2), was isolated as a colorless oil after purification by preparative TLC.

^{1}H NMR (500 MHz, Chloroform-*d*) δ (ppm) 7.45 – 7.28 (m, 5H), 4.29 (dtd, *J* = 6.9, 6.3, 3.9 Hz, 1H), 3.87 (ddd, *J* = 12.1, 6.3, 3.9 Hz, 1H), 3.76 (dt, *J* = 12.1, 6.3 Hz, 1H), 3.22 (dd, *J* = 14.1, 6.9 Hz, 1H), 3.11 (dd, *J* = 14.1, 6.3 Hz, 1H), 2.84 (t, *J* = 6.2 Hz, 1H). **^{13}C NMR (125 MHz, Chloroform-*d*)** δ (ppm) 137.1, 129.3, 128.6, 127.0, 65.8, 64.6, 40.6.[2]

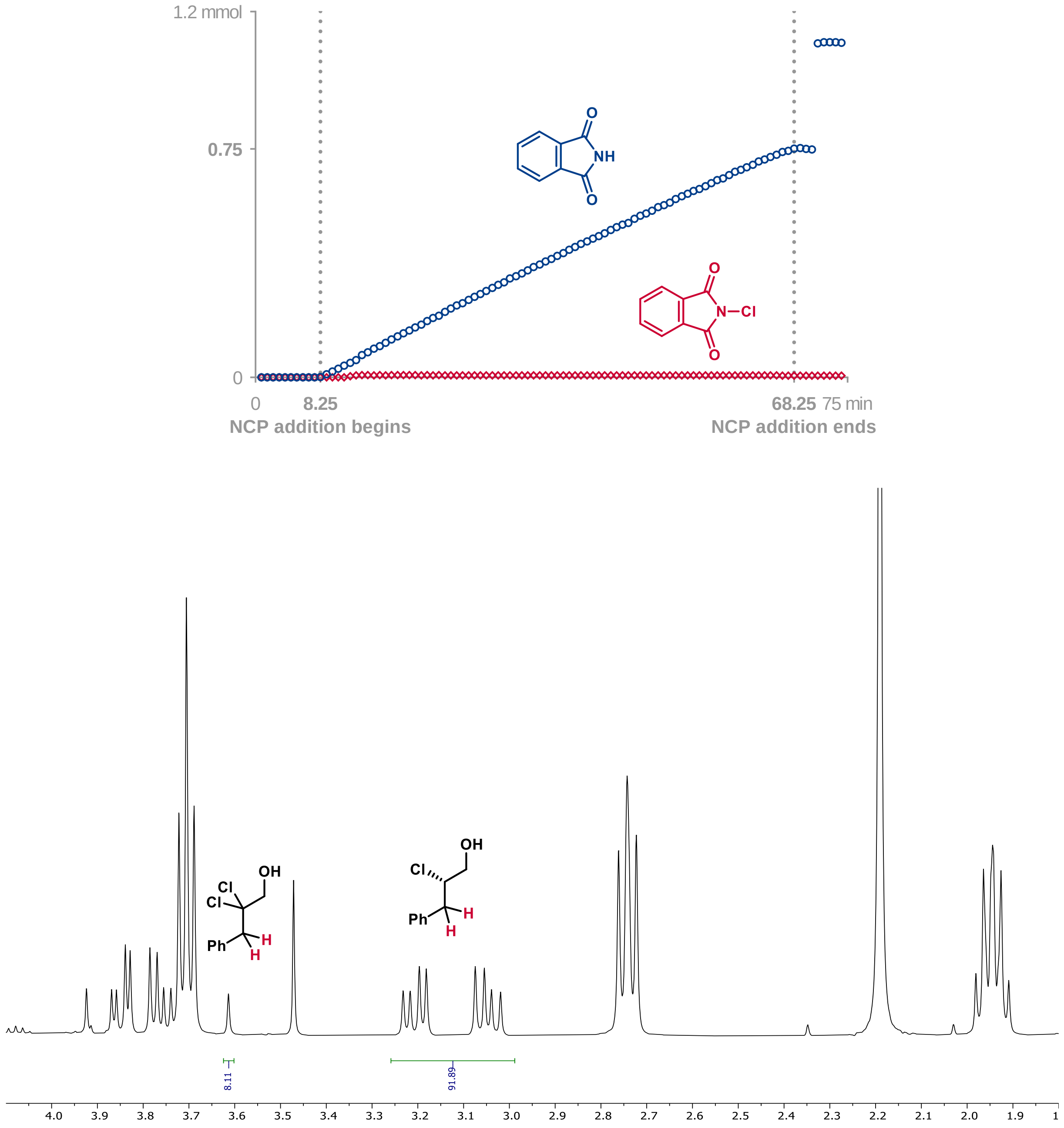

*Figure 29 – The reaction with catalyst **3a** showed excellent yield and selectivity.*

The FTIR data indicated that all NCP added was consumed (0.75 mmol). The crude NMR, taken post-reduction, indicated that the ratio of mono to dichlorinated product was 92:8. As each molecule of dichlorinated product accounts for two molecules of NCP, the monochlorinated product accounts for 85% of the NCP consumed.

$$\text{Yield} = 100\frac{\text{NCP}_{\text{consumed}} \cdot \text{F}_{\text{mono}}}{\text{NCP}_{\text{added}}} = 100\frac{0.75 \cdot 0.85}{0.75} = 85\%$$

The er, assessed by chiral HPLC, was 99:1 (S–100).

10.2. Reaction with NCS as a chlorinating reagent

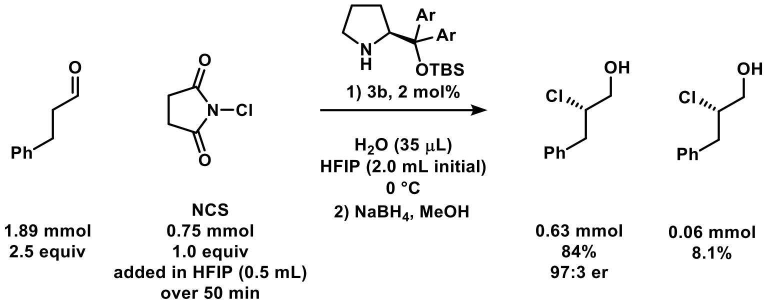

We have also demonstrated the use of NCS, instead of NCP, to achieve good yields and excellent enantioselectivity. Stock solutions in HFIP were used in the preparation of this reaction.

SL	Component	Volume / mL	Mass / mg	Concentration / M
**A**	Hydrocinnamaldehyde	1.0	502.7	3.75
**B**	NCS	2.0	198.8	1.49
**C**	cat **3b**	1.0	19.6	0.03
**D**	H_2O	1.0	71.0	3.94
**E**	Succinimide	1.0	143.7	1.45

A vial containing 500 µL of HFIP, a magnetic stirrer and fitted with the ReactIR probe was charged with stock solutions **A** (500 µL, 1.89 mmol, 2.5 equiv), **C** (500 µL, 0.015 mmol, 2 mol%) and **D** (500 µL, 1.97 mmol). The reaction mixture was cooled to 0 °C using an ice bath, stirred for 9 min and consecutive FTIR spectra of 122 scans were taken with the ReactIR. Stock solution **B** (500 µL, 0.75 mmol, 1.0 equiv) was added using a syringe pump over 50 min. After the addition ended, a further 6 spectra were collected before stock solution **E** (500 µL, 0.73 mmol, 1.0 equiv) was added for calibration. A final 4 spectra were collected, before the reaction mixture was quenched in a stirred solution of $NaBH_4$ (750 mg, 19.8 mmol) in MeOH (5 mL). After stirring for 5 min, brine (5 mL) and H_2O (5 mL) were added. The mixture was extracted with CH_2Cl_2 (4 x 15 mL), before the collected organic phase was washed with brine (15 mL), dried over $MgSO_4$ and concentrated on a rotary evaporator. The product, 2-chloro-3-phenylpropan-1-ol (R_f = 0.38, CH_2Cl_2), was isolated as a colorless oil after purification by preparative TLC.

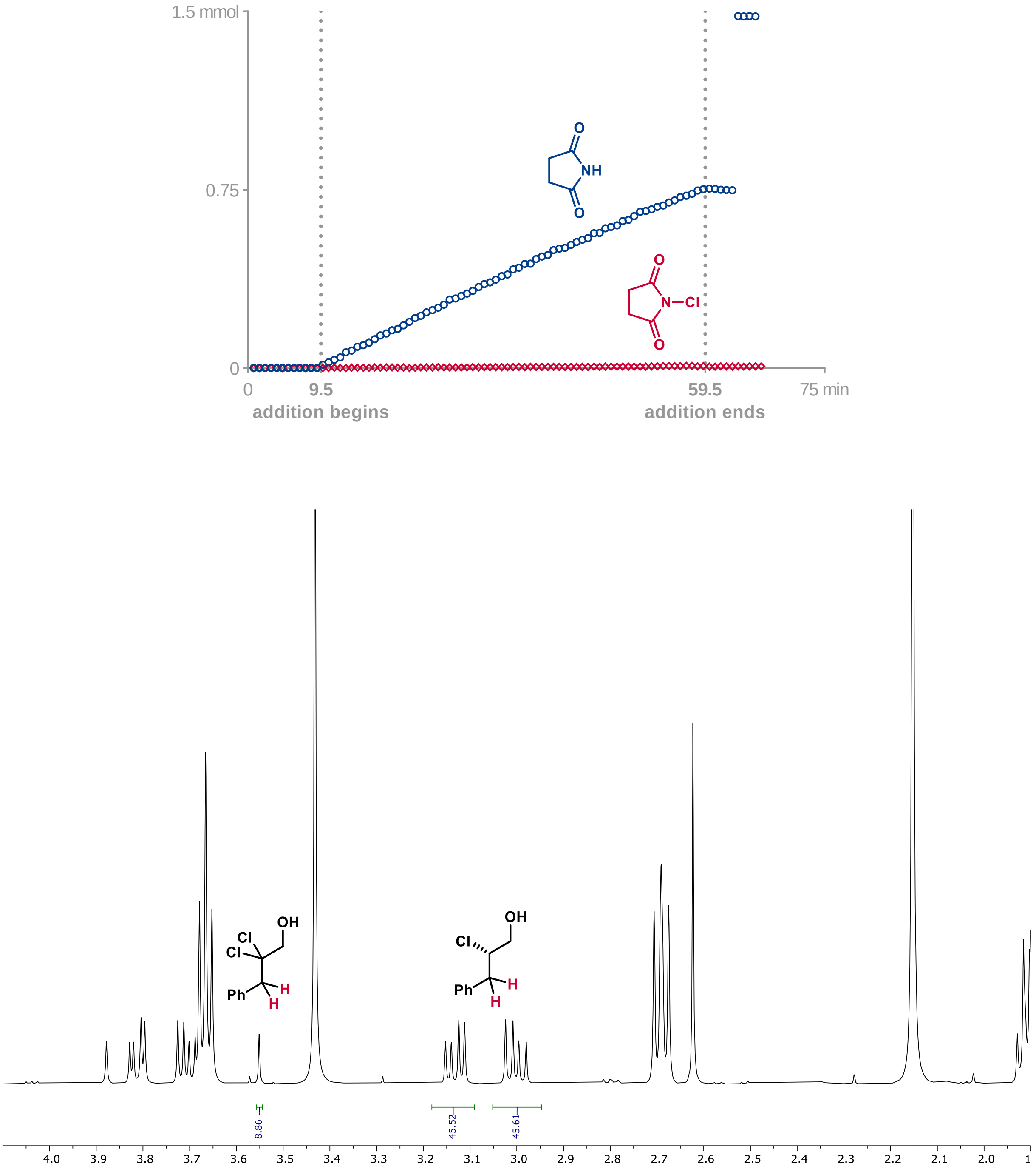

Figure 30 – The use of NCS as a chlorinating agent displayed a good yield.

The FTIR data indicated that all NCS added was consumed (0.75 mmol). The crude NMR, taken post-reduction, indicated that the ratio of mono to dichlorinated product was 91:9. As each molecule of dichlorinated product accounts for two molecules of NCS, the monochlorinated product accounts for 84% of the NCS consumed.

$$\text{Yield} = 100\frac{\text{NCS}_{\text{consumed}} \cdot \text{F}_{\text{mono}}}{\text{NCS}_{\text{added}}} = 100\frac{0.75 \cdot 0.84}{0.75} = 84\%$$

The er, assessed by chiral HPLC, was 97:3 (S–101).

10.3. *With catalyst* **3a**

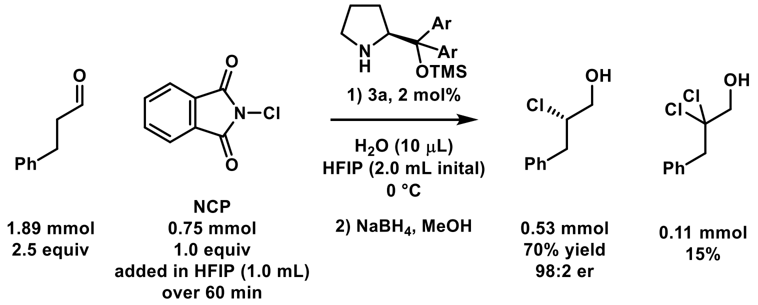

To achieve a high enantiomeric ratio with the use of a less sterically congested catalyst **3a**, a slight amount of monochlorinated product was sacrificed as dichlorinated product. The over chlorination of a slight amount of monochlorinated product acted as a kinetic resolution and increased the enantiomeric ratio of the remaining product. Dichlorinated product could easily be separated from monochlorinated. Multiple initial concentrations of water were tested with 60 min additions of NCP to determine which provides the best balance between high er (> 97.5:2.5) and overall yield.

Stock solutions in HFIP were used in the preparation of this reaction.

SL	Component	Volume / mL	Mass / mg	Concentration / M
**A**	Hydrocinnamaldehyde	1.0	504.5	3.76
**B**	NCP	2.0	272.4	0.75
**C**	cat **3a**	1.0	18.1	0.03
**D**	H_2O	1.0	19.7	1.09
**E**	Phthalimide	1.0	103.2	0.70

A vial containing 500 µL of HFIP, a magnetic stirrer and fitted with the ReactIR probe was charged with stock solutions **A** (500 µL, 1.89 mmol, 2.5 equiv), **C** (500 µL, 0.015 mmol, 2 mol%) and **D** (500 µL, 0.55 mmol). The reaction mixture was cooled to 0 °C using an ice bath, stirred for 14 min and consecutive FTIR spectra of 122 scans were taken with the ReactIR. Stock solution **B** (1.0 mL, 0.75 mmol, 1.0 equiv) was added using a syringe pump over 60 min. After the addition ended, a further 12 spectra were collected before stock solution **E** (500 µL, 0.35 mmol, 0.5 equiv) was added for calibration. A final 6 spectra were collected, before the reaction mixture was quenched in a stirred solution of $NaBH_4$ (750 mg, 19.8 mmol) in MeOH (5 mL). After stirring for 5 min, brine (5 mL) and H_2O (5 mL) were added. The mixture was extracted with CH_2Cl_2 (4 x 15 mL), before the collected organic phase was washed with brine (15 mL), dried over $MgSO_4$ and concentrated on a rotary evaporator. The product, 2-chloro-3-phenylpropan-1-ol (R_f = 0.38, CH_2Cl_2), was isolated as a colorless oil after purification by preparative TLC.

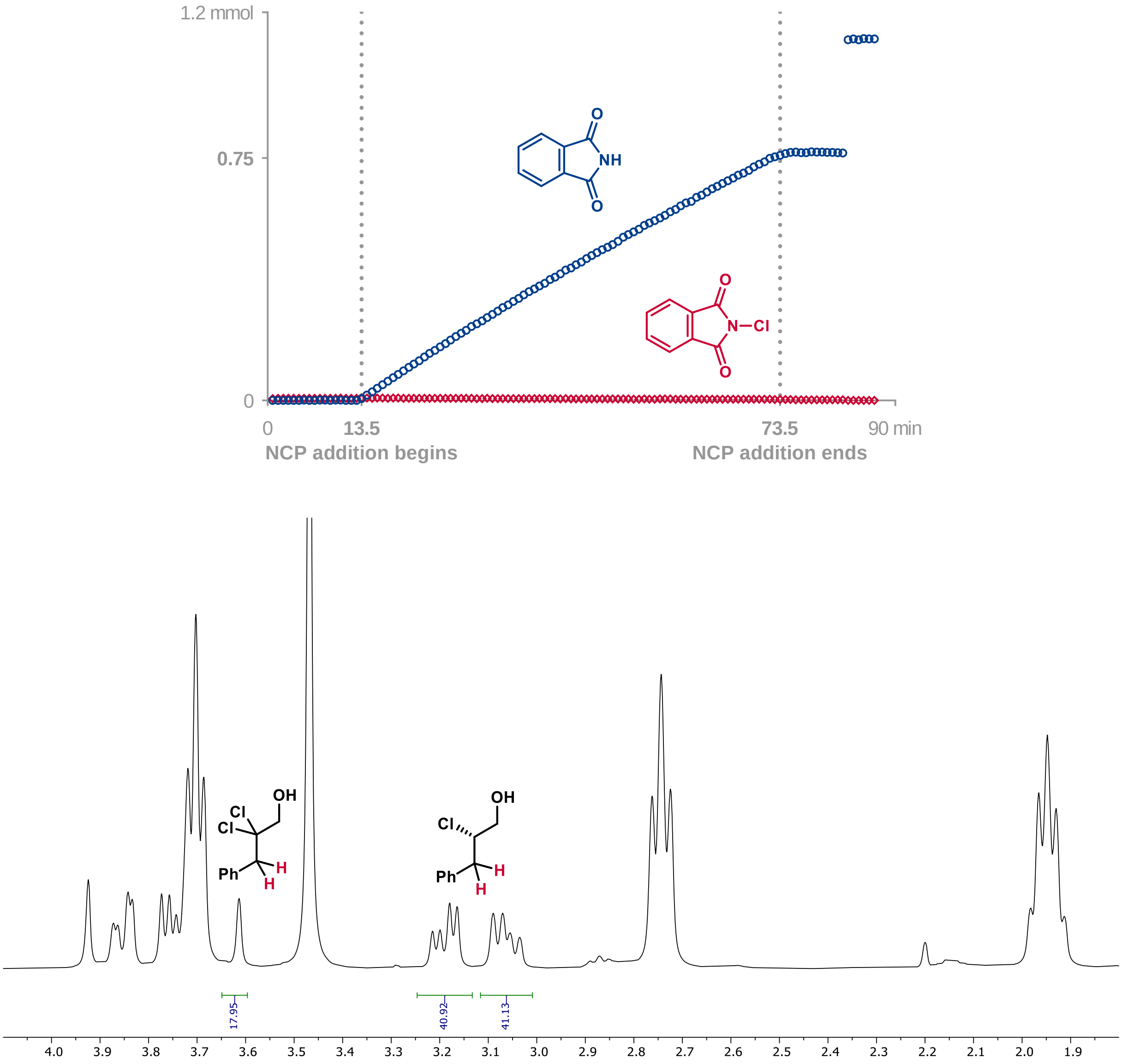

*Figure 31 – In order to obtain a good er with catalyst **3a**, substantial yield was sacrificed to dichlorination.*

The FTIR data indicated that all NCP added was consumed (0.75 mmol). The crude NMR, taken post-reduction, indicated that the ratio of mono to dichlorinated product was 82:18. As each molecule of dichlorinated product accounts for two molecules of NCP, the monochlorinated product accounts for 70% of the NCP consumed.

$$\mathrm{Yield} = 100\frac{\mathrm{NCP_{consumed}} \cdot \mathrm{F_{mono}}}{\mathrm{NCP_{added}}} = 100\frac{0.75 \cdot 0.70}{0.75} = 70\%$$

The er, assessed by chiral HPLC, was 98:2 (S–102).

10.4. Reaction with 1 mol% catalyst loading

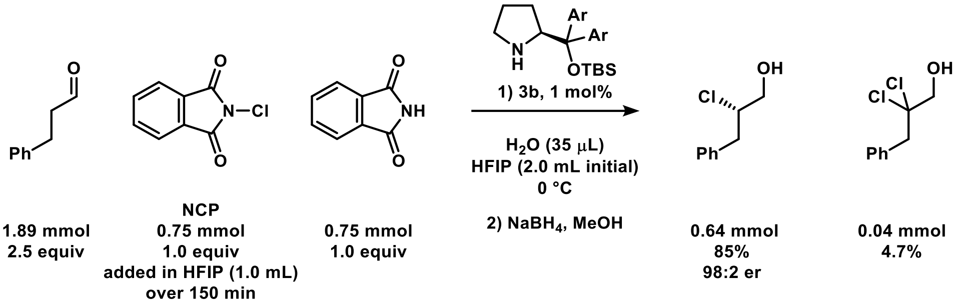

Further reduction in catalyst loading beyond 2 mol% initially proved challenging, though the reversibility of the catalyst chlorination step allowed us to propose a solution. Through the addition of 1.0 equiv of phthalimide prior to the start of the reaction, we observed a reduction in the rate of NCP build up. This outcome indicated that the proportion of free catalyst versus chlorinated was increased, and the reaction proceeded with a good yield and enantioselectivity. The following stock solutions in HFIP were used.

SL	Component	Volume / mL	Mass / mg	Concentration / M
**A**	Hydrocinnamaldehyde	1.0	504.5	3.76
**B**	NCP	2.0	272.4	0.75
**C**	cat **3b**	2.0	18.5	0.015
**D**	H_2O	1.0	73.0	4.05

A vial containing 500 µL of HFIP, a magnetic stirrer and fitted with the ReactIR probe was charged with stock solutions **A** (500 µL, 1.89 mmol, 2.5 equiv), **C** (500 µL, 7.5 µmol, 1 mol%) and **D** (500 µL, 2.02 mmol). The reaction mixture was cooled to 0 °C using an ice bath, stirred for 4 min and consecutive FTIR spectra of 122 scans were taken with the ReactIR. Phthalimide (111.4 mg, 0.76 mmol) was added as a solid to the reaction vessel. This phthalimide loading was used to calibrate the FTIR data. After the phthalimide had dissolved, five FTIR spectra were collected. This addition of phthalimide was also used to calibrate the FTIR data. Stock solution **B** (0.5 mL, 0.75 mmol, 1.0 equiv) was added using a syringe pump over 150 min. A final five spectra were collected, before the reaction mixture was quenched in a stirred solution of $NaBH_4$ (750 mg, 19.8 mmol) in MeOH (5 mL). After stirring for 5 min, brine (5 mL) and H_2O (5 mL) were added. The mixture was extracted with CH_2Cl_2 (4 x 15 mL), before the collected organic phase was washed with brine (15 mL), dried over $MgSO_4$ and concentrated on a rotary evaporator. The product, 2-chloro-3-phenylpropan-1-ol (R_f = 0.38, CH_2Cl_2), was isolated as a colorless oil after purification by preparative TLC.

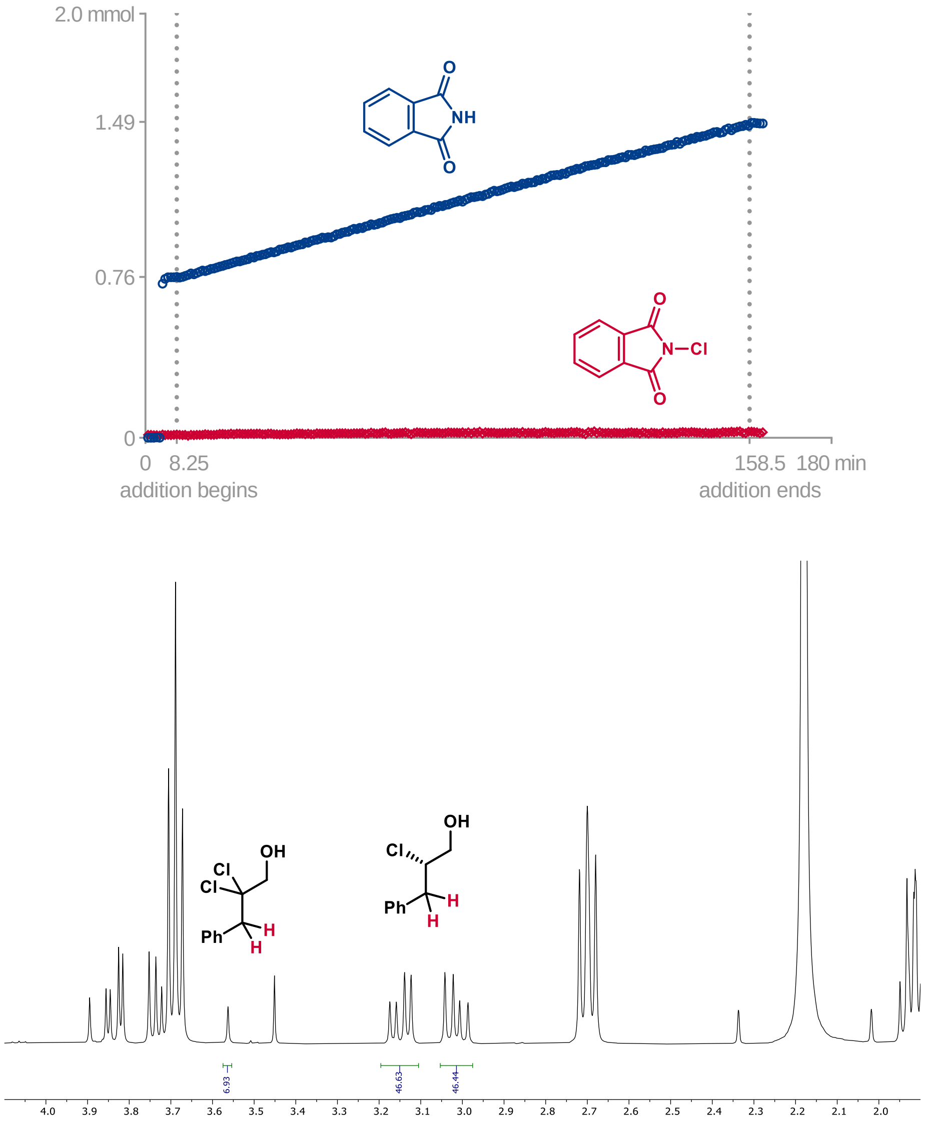

Figure 32 – Added phthalimide allowed the reduction of catalyst loading while maintaining good yields.

The FTIR data indicated that almost all NCP added was consumed (0.73 mmol). The crude NMR, taken post-reduction, indicated that the ratio of mono to dichlorinated product was 93:7. As each molecule of dichlorinated product accounts for two molecules of NCP, the monochlorinated product accounts for 87% of the NCP consumed.

$$\text{Yield} = 100\frac{\text{NCP}_{\text{consumed}} \cdot \text{F}_{\text{mono}}}{\text{NCP}_{\text{added}}} = 100\frac{0.73 \cdot 0.87}{0.75} = 85\%$$

The er, assessed by chiral HPLC, was 98:2 (S–103).

10.5. Reaction at room temperature

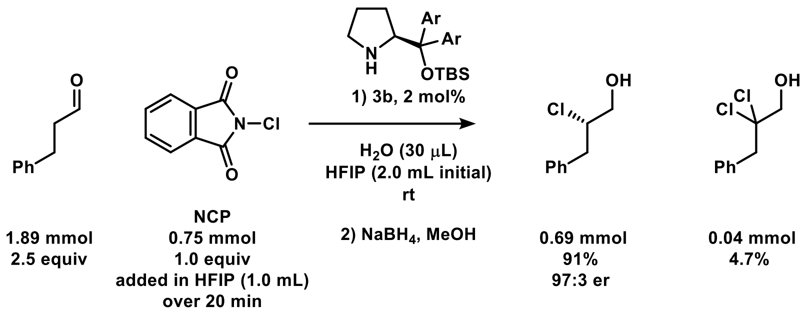

When the reaction was carried out at room temperature, the yield was increased but the selectivity slightly reduced.

SL	Component	Volume / mL	Mass / mg	Concentration / M
**A**	Hydrocinnamaldehyde	1.0	506.4	3.77
**B**	NCP	2.0	272.4	0.75
**C**	cat **3b**	1.0	19.1	0.03
**D**	H_2O	1.0	61.5	3.42
**E**	Phthalimide	1.0	107.3	0.73

A vial containing 500 µL of HFIP, a magnetic stirrer and fitted with the ReactIR probe was charged with stock solutions **A** (500 µL, 1.89 mmol, 2.5 equiv), **C** (500 µL, 0.015 mmol, 2 mol%) and **D** (500 µL, 1.71 mmol). The reaction mixture was stirred for 4 min and consecutive FTIR spectra of 122 scans were taken with the ReactIR. Stock solution **B** (1.0 mL, 0.75 mmol, 1.2 equiv) was added using a syringe pump over 20 min. After the addition ended, a further 5 spectra were collected before stock solution **E** (500 µL, 0.365 mmol, 0.5 equiv) was added for calibration. A final 5 spectra were collected, before the reaction mixture was quenched in a stirred solution of $NaBH_4$ (750 mg, 19.8 mmol) in MeOH (5 mL). After stirring for 5 min, brine (5 mL) and H_2O (5 mL) were added. The mixture was extracted with CH_2Cl_2 (4 x 15 mL), before the collected organic phase was washed with brine (15 mL), dried over $MgSO_4$ and concentrated on a rotary evaporator. The product, 2-chloro-3-phenylpropan-1-ol (R_f = 0.38, CH_2Cl_2), was isolated as a colorless oil after purification by preparative TLC.

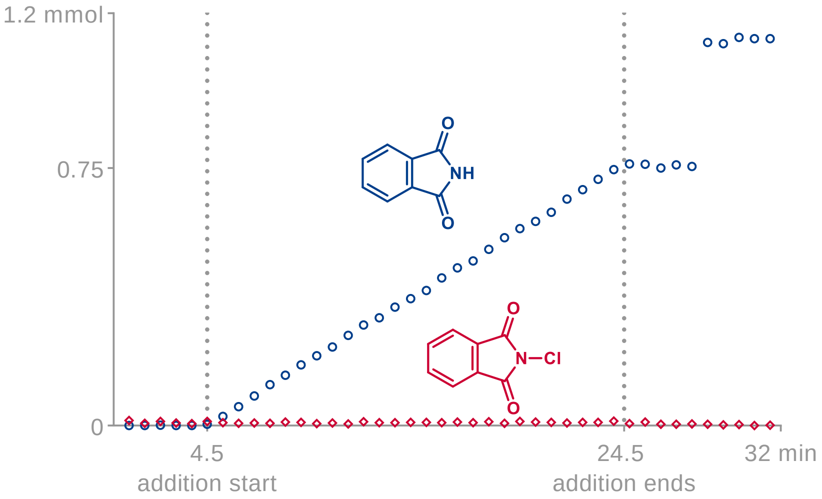

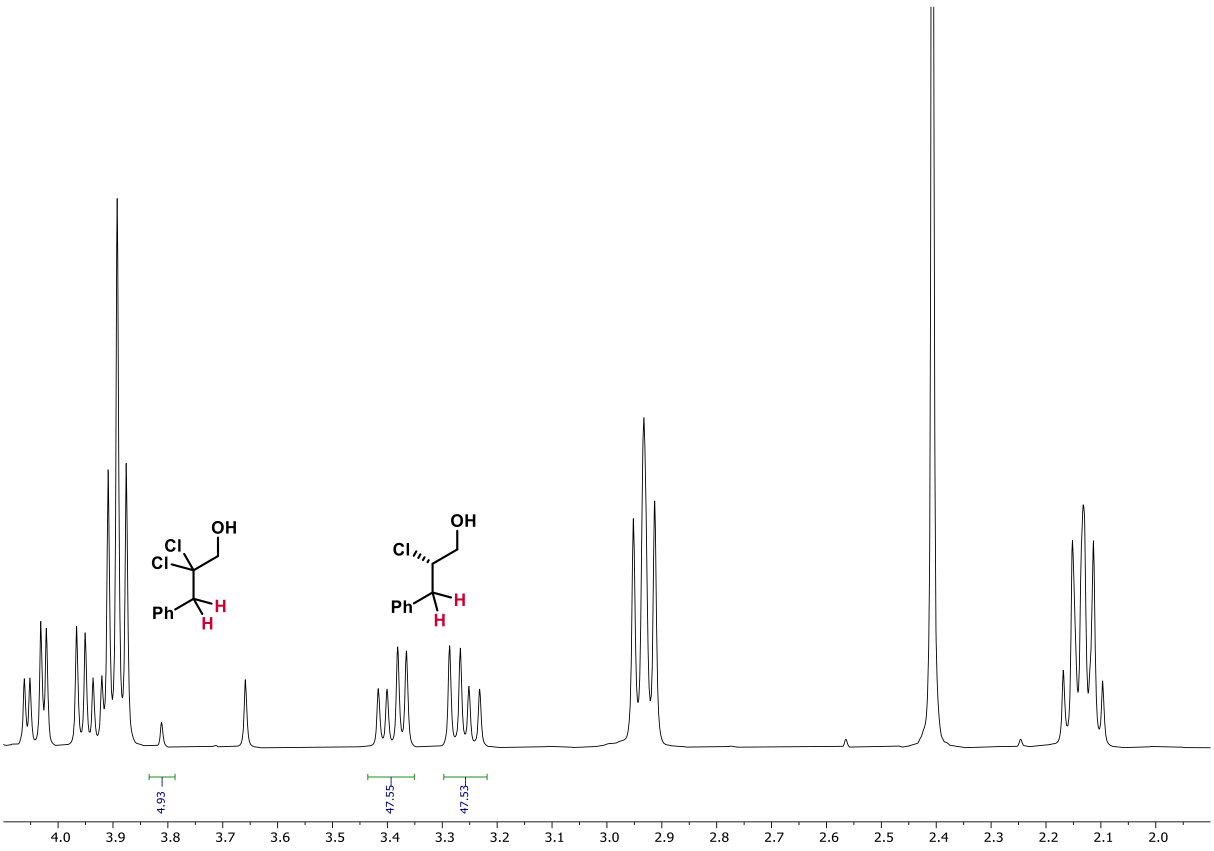

Figure 33 – Room temperature reaction displayed an excellent yield.

The FTIR data indicates that all NCP added was consumed (0.75 mmol). The crude NMR, taken post-reduction, indicates that the ratio of mono to dichlorinated product was 95:5. As each molecule of dichlorinated product accounts for two molecules of NCP, the monochlorinated product accounts for 91% of the NCP consumed.

$$\text{Yield} = 100\frac{\text{NCP}_{\text{consumed}} \cdot \text{F}_{\text{mono}}}{\text{NCP}_{\text{added}}} = 100\frac{0.75 \cdot 0.91}{0.75} = 91\%$$

The er, assessed by chiral HPLC, was 97:3 (S–104).

10.6. Reaction with aldehyde as the limiting reagent

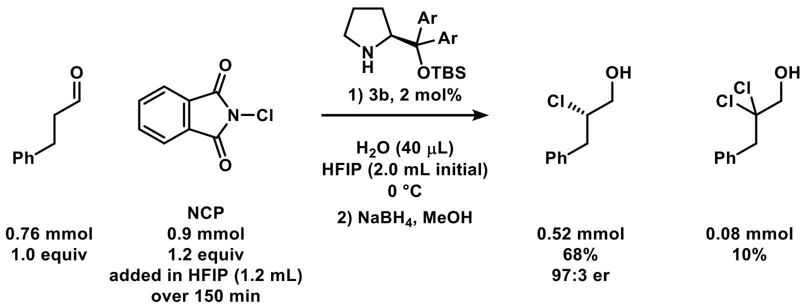

Reducing the amount of aldehyde present had two inherent problems. Firstly, the product was more likely to be racemized towards the end of the reaction, an inherent problem with any aminocatalytic reaction where the product has the same functional groups as the starting material. Therefore, the product will compete with the starting material for the catalyst. Secondly, the catalyst was chlorinated more rapidly because there was a higher proportion of free catalyst in equilibrium. This reaction required a slower rate of addition than usual and an excess of chlorinating agent to be added, as the chlorinated catalyst cannot effectively racemize the product. This excess of NCP also mitigated the need to quench the reaction at the ideal moment. The following stock solutions in HFIP were used for this reaction.

SL	Component	Volume / mL	Mass / mg	Concentration / M
**A**	Hydrocinnamaldehyde	1.0	203.1	1.52
**B**	NCP	2.0	273.0	0.75
**C**	cat **3b**	1.0	18.1	0.03
**D**	H_2O	1.0	82.3	4.57
**E**	Phthalimide	1.0	103.6	0.70

A vial containing 500 µL of HFIP, a magnetic stirrer and fitted with the ReactIR probe was charged with stock solutions **A** (500 µL, 0.76 mmol, 1.0 equiv), **C** (500 µL, 0.015 mmol, 2 mol%) and **D** (500 µL, 2.24 mmol). The reaction mixture was cooled to 0 °C using an ice bath, stirred for 12 min and consecutive FTIR spectra of 122 scans were taken with the ReactIR. Stock solution **B** (1.2 mL, 0.90 mmol, 1.2 equiv) was added using a syringe pump over 150 min. After the addition ended, a further 6 spectra were collected before stock solution **E** (500 µL, 0.35 mmol, 0.47 equiv) was added for calibration. A final 4 spectra were collected, before the reaction mixture was quenched in a stirred solution of $NaBH_4$ (750 mg, 19.8 mmol) in MeOH (5 mL). After stirring for 5 min, brine (5 mL) and H_2O (5 mL) were added. The mixture was extracted with CH_2Cl_2 (4 x 15 mL), before the collected organic phase was washed with brine (15 mL), dried over $MgSO_4$ and concentrated on a rotary evaporator. The product, 2-chloro-3-phenylpropan-1-ol (R_f = 0.38, CH_2Cl_2), was isolated as a colorless oil after purification by preparative TLC.

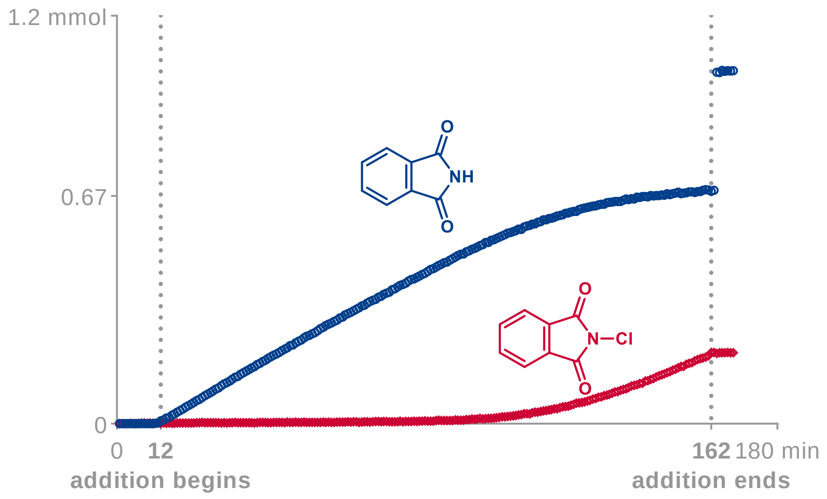

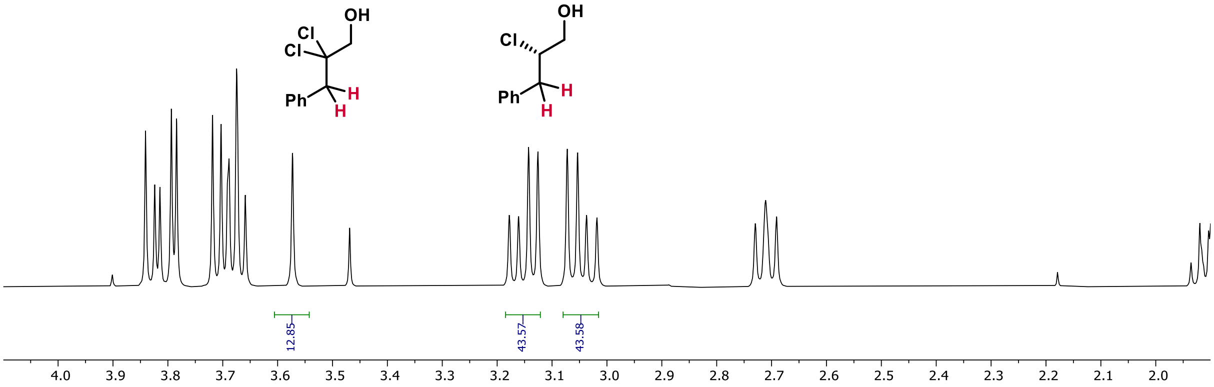

Figure 34 – The reaction with aldehyde as the limiting reagent had a reduced yield.

The FTIR data indicates that some of the NCP added was consumed (0.67 mmol). The crude NMR, taken post-reduction, indicates that the ratio of mono to dichlorinated product was 87:13. As each molecule of dichlorinated product accounts for two molecules of NCP, the monochlorinated product accounts for 77% of the NCP consumed.

$$\text{Yield} = 100\frac{\text{NCP}_{\text{consumed}} \cdot \text{F}_{\text{mono}}}{\text{CHO}_{\text{added}}} = 100\frac{0.67 \cdot 0.77}{0.76} = 68\%$$

The er, assessed by chiral HPLC, was 97:3 (S–105).

10.7. Reactions with different aldehydes

To ensure the good yields and excellent selectivity we had demonstrated could be achieved with a small range of aldehydes, further reactions were carried out with octanal, isovaleraldehyde, propanal, δ-valerolactol, pentanal, 5-bromopentanal and dodecanal as substrates. These reactions were carried out after following the rapid and convenient optimization procedure described in Section 9.

10.7.1. Octanal

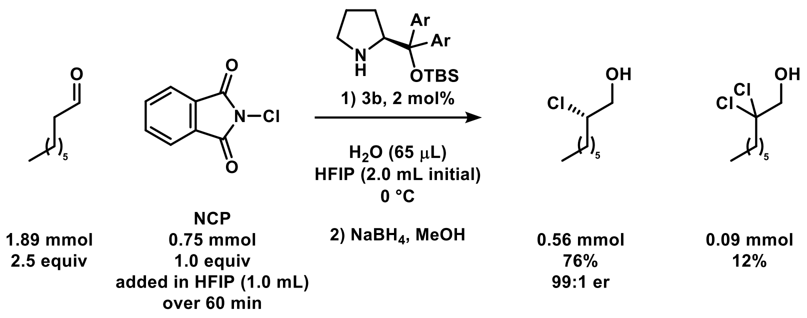

Optimization for octanal required no adjustment to addition time but required more water than hydrocinnamaldehyde to reduce dichlorination to an acceptable level.

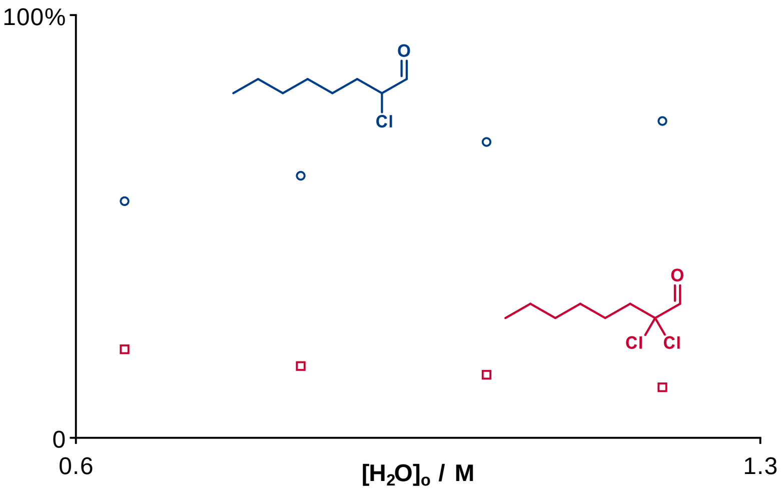

Figure 35 – Octanol required an increased $[H_2O]_o$ to limit dichlorination.

Stock solutions were used in the preparation of this reaction.

SL	Component	Volume / mL	Mass / mg	Concentration / M
**A**	Octanal	1.0	487.9	3.75
**B**	NCP	2.0	272.6	0.75
**C**	cat **3b**	1.0	18.1	0.03
**D**	H_2O	1.0	130.1	7.23
**E**	Phthalimide	1.0	113.7	0.77

A vial containing 500 µL of HFIP, a magnetic stirrer and fitted with the ReactIR probe was charged with stock solutions **A** (500 µL, 1.89 mmol, 2.5 equiv), **C** (500 µL, 0.015 mmol, 2 mol%) and **D** (500 µL, 3.61 mmol). The reaction mixture was cooled to 0 °C using an ice bath, stirred for 6 min and consecutive FTIR spectra of 122 scans were taken by the ReactIR. Stock solution **B** (1.0 mL, 0.75 mmol, 1.0 equiv) was added using a syringe pump over 60 min. After the addition ended, a further 5 data points were collected before stock solution **E** (500µL, 0.38 mmol) was added for calibration. A final 5 data points were collected, before the reaction mixture was quenched in a stirred solution of $NaBH_4$ (750 mg, 19.8 mmol) in MeOH (5 mL). After stirring for 5 min, brine (5 mL) and H_2O (5 mL) were added. The mixture was extracted with CH_2Cl_2 (4 x 15 mL), before the collected organic phase was washed with brine (15 mL), dried over $MgSO_4$ and concentrated on a rotary evaporator. The product, 2-chloro-octan-1-ol, was isolated as a colorless oil after purification by preparative TLC. The er was determined by esterification with Mosher's acid chloride, following the procedure by Suzuki *et al.*[10] and examination of the ^{19}F NMR peaks against that of a racemic sample (S–106).

^{1}H NMR (500 MHz, Chloroform-*d*) δ (ppm) 4.03 (dddd, *J* = 8.7, 7.3, 5.1, 3.5 Hz, 1H), 3.84 –3.76 (m, 1H), 3.66 (ddd, *J* = 11.4, 6.8, 3.5 Hz, 1H), 1.98 (t, J = 6.8 Hz 1H), 1.83 – 1.64 (m, 2H), 1.56 – 1.57 (m, 1H), 1.46 –1.36 (m, 1H), 1.35 –1.24 (m, 6H), 0.89 (t, *J* = 6.9 Hz, 3H). **^{13}C NMR (125 MHz, Chloroform-*d*)** δ (ppm) 67.2, 65.7, 34.4, 31.8, 28.9, 26.5, 22.7, 14.2.[7]

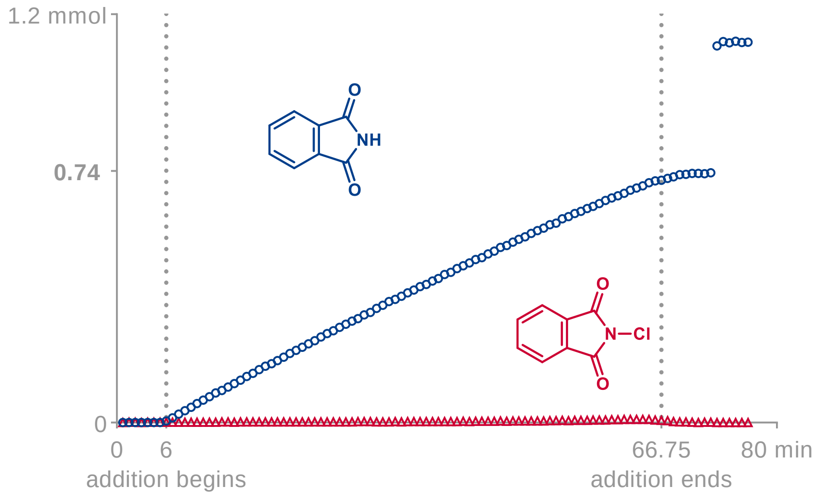

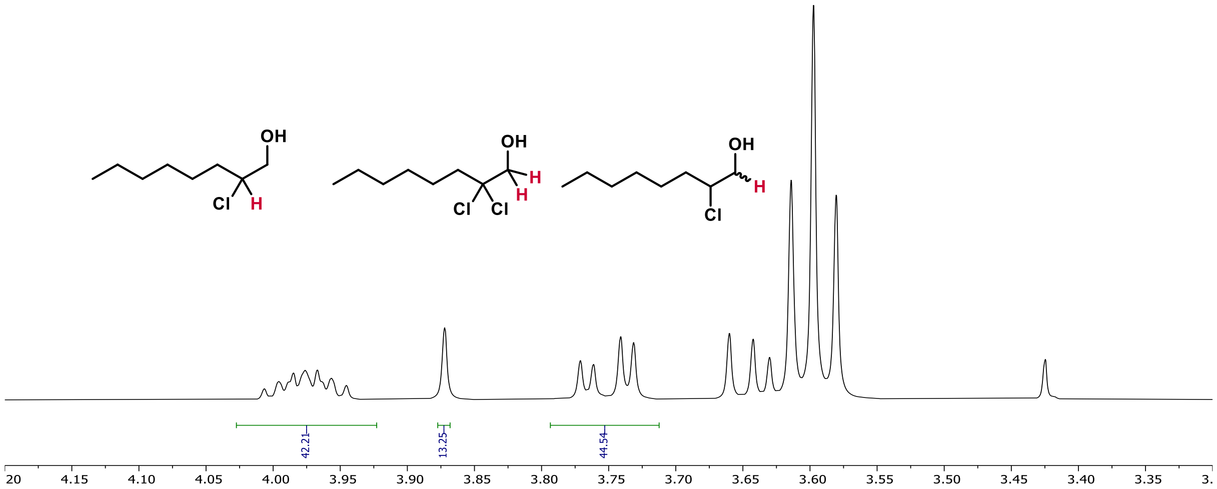

Figure 36 – The α-chlorination of octanal was successful, with a good yield.

The FTIR data indicates that all NCP added was consumed (0.74 mmol). The crude NMR, taken post-reduction, indicates that the ratio of mono to dichlorinated product was 87:13. As each molecule of dichlorinated product accounts for two molecules of NCP, the monochlorinated product accounts for 77% of the NCP consumed.

$$\text{Yield} = 100\frac{\text{NCP}_{\text{consumed}} \cdot \text{F}_{\text{mono}}}{\text{NCP}_{\text{added}}} = 100\frac{0.74 \cdot 0.77}{0.75} = 76\%$$

The er, measured by ^{19}F NMR of the Mosher ester derivative, was 99:1 (S–106).

10.7.2. Isovaleraldehyde

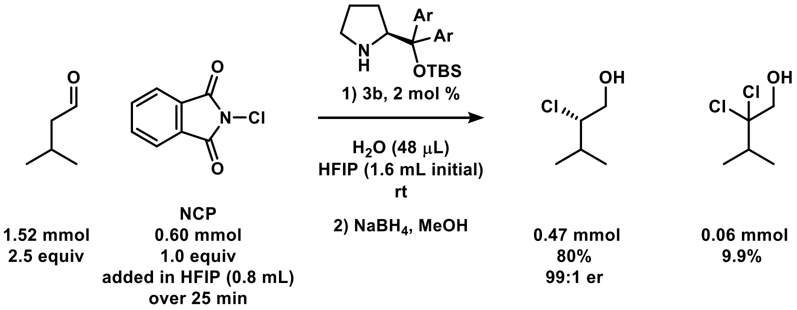

We proposed that β-substitution of the substrate resulted in an increased time of addition as there was less binding between the aldehyde and catalyst due to increased steric hindrance. We carried out multiple additions at 0 ^{o}C and found that increasing the time of addition only slightly increased the conversion. Indeed, even the addition of phthalimide with the aim of limiting catalyst chlorination did not significantly improve the yield.

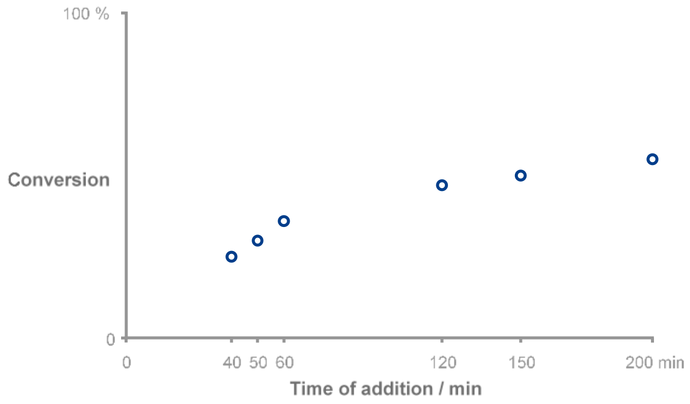

However, when we trialed room temperature reaction, we achieved excellent yield with addition times of just 25 minutes. The amount of water did not require significant adjustment to achieve a good er. The following stock solutions were used in the preparation of this reaction. The reaction was performed on a slightly smaller scale (0.60 mmol compared to 0.75 mmol)

SL	Component	Volume / mL	Mass / mg	Concentration / M
**A**	Isovaleraldehyde	1.0	328.1	3.81
**B**	NCP	2.0	272.6	0.75
**C**	cat **3b**	1.0	19.4	0.03
**D**	H_2O	1.0	120.9	6.72
**E**	Phthalimide	1.0	107.2	0.73

A vial containing 400 µL of HFIP, a magnetic stirrer and fitted with the ReactIR probe was charged with stock solutions **A** (400 µL, 1.52 mmol, 2.5 equiv), **C** (400 µL, 0.012 mmol, 2 mol%) and **D** (400 µL, 2.67 mmol). The reaction mixture was stirred for 6 min and consecutive FTIR spectra of 41 scans were taken by the ReactIR. Stock solution **B** (800 µL, 0.6 mmol, 1.0 equiv) was added using a syringe pump over 25 min. After the addition ended, a further 5 data points were collected before stock solution **E** (250 µL, 0.18 mmol) was added for calibration. A final 5 data points were collected, before the reaction

mixture was quenched in a stirred solution of $NaBH_4$ (750 mg, 19.8 mmol) in MeOH (5 mL). After stirring for 5 min, brine (5 mL) and H_2O (5 mL) were added. The mixture was extracted with CH_2Cl_2 (4 x 15 mL), before the collected organic phase was washed with brine (15 mL), dried over $MgSO_4$ and concentrated on a rotary evaporator. The product, 2-chloro-3-methylbutan-1-ol, was isolated as a colorless oil after purification by preparative TLC. The er was determined by esterification using Mosher's acid chloride, and examination of the ^{19}F NMR peaks against that of a racemic sample.

^{1}H NMR (400 MHz, Chloroform-*d*) δ (ppm) 3.92 (ddd, *J* = 7.9, 5.2, 3.8 Hz, 1H), 3.80 (ddd, *J* = 12.0, 8.3, 3.8 Hz, 1H), 3.73 (ddd, *J* = 12.0, 7.9, 4.8 Hz, 1H), 2.10 – 2.02 (m, 1H), 1.98 (dd, *J* = 8.3, 4.8 Hz, 1H), 1.03 (dd, *J* = 8.03, 6.07 Hz, 6H). **^{13}C NMR (125 MHz, Chloroform-*d*)** δ (ppm) 65.6, 31.6, 20.1, 18.3.[4]

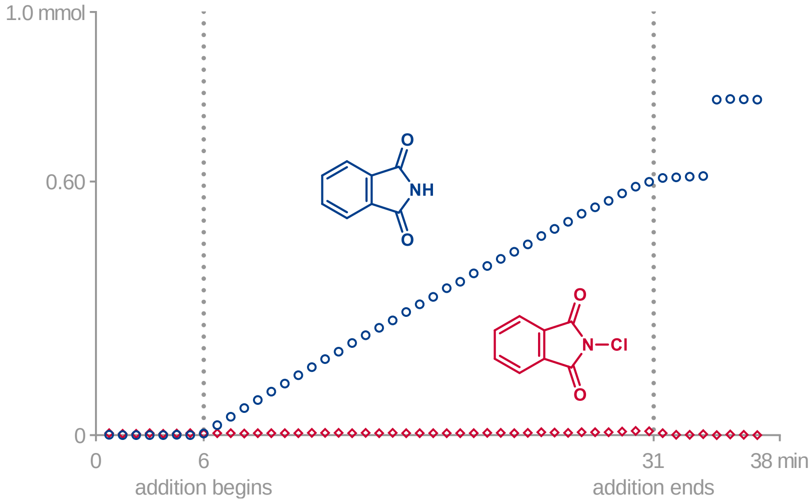

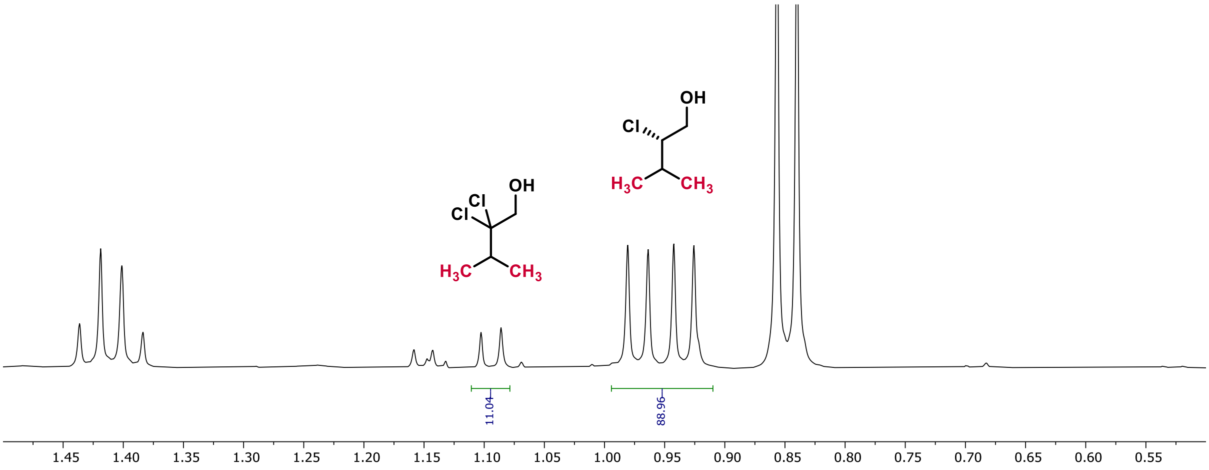

Figure 37 – The α-chlorination of isovaleraldehyde was carried out at rt and showed good yield.

The FTIR data indicates that all NCP added was consumed (0.60 mmol). The crude NMR, taken post-reduction, indicates that the ratio of mono to dichlorinated product was 89:11. As each molecule of dichlorinated product accounts for two molecules of NCP, the monochlorinated product accounts for 80% of the NCP consumed.

$$\text{Yield} = 100\frac{\text{NCP}_{\text{consumed}} \cdot \text{F}_{\text{mono}}}{\text{NCP}_{\text{added}}} = 100\frac{0.60 \cdot 0.80}{0.60} = 80\%$$

The er, measured by ^{19}F NMR of the Mosher ester derivative, was 99:1. (S–107).

10.7.3. Propanal

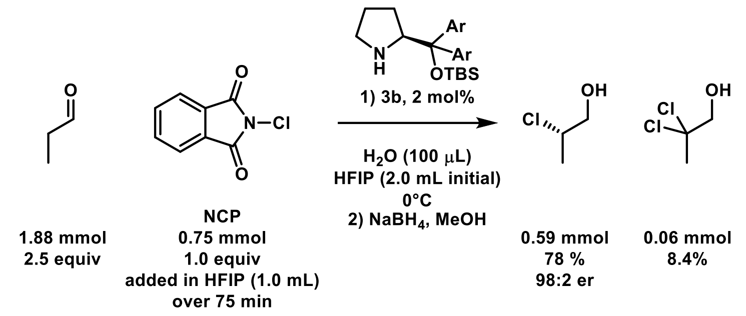

Optimization for propanal involved minor changes in the amount of water present, but an increase in the time of addition to prevent catalyst deactivation. The following stock solutions were used for this reaction.

SL	Component	Volume / mL	Mass / mg	Concentration / M
**A**	Propanal	1.0	219.0	3.77
**B**	NCP	2.0	273.0	0.75
**C**	cat **3b**	1.0	19.2	0.03
**D**	H_2O	1.0	198.7	11.0
**E**	Phthalimide	1.0	110.3	0.75

A vial containing 500 µL of HFIP, a magnetic stirrer and fitted with the ReactIR probe was charged with stock solutions **A** (500 µL, 1.88 mmol, 2.5 equiv), **C** (500 µL, 0.015 mmol, 2 mol%) and **D** (500 µL, 5.5 mmol). The reaction was cooled to 0 °C using an ice bath, stirred for 9 min and consecutive FTIR spectra of 122 scans were taken by the ReactIR. Stock solution **B** (1.0 mL, 0.75 mmol, 1.0 equiv) was added using a syringe pump over 75 min. After the addition ended, a further 5 data points were collected before stock solution **E** (500 µL, 0.375 mmol) was added for calibration. A final 5 data points were collected, before the reaction mixture was quenched in a stirred solution of $NaBH_4$ (750 mg, 19.8 mmol) in MeOH (0.5 mL, minimal methanol ensured easier separation of the benzoylated product). After stirring for 5 min, brine (5 mL) and H_2O (5 mL) were added. The mixture was extracted with CH_2Cl_2 (6 x 15 mL), before the collected organic phase was washed with brine (15 mL), dried over $MgSO_4$ and concentrated on a rotary evaporator. The product, 2-chloro-propan-1-ol, was not isolated due to its volatility and was benzoylated directly. The er was determined by chiral HPLC after benzoylation (S–108).

^{1}H NMR (400 MHz, Chloroform-*d*) δ (ppm) 8.07 (dd, *J* = 7.7, 1.4 Hz, 2H), 7.59 (t, *J* = 7.7 Hz, 1H), 7.46 (t, *J* = 7.7 Hz, 2H), 4.44 (s, 1H), 4.43 (s, 1H), 4.36 – 4.29 (m, 1H), 1.61 (d, *J* = 6.6 Hz, 3H). **^{13}C NMR (125 MHz, Chloroform-*d*)** δ (ppm) 166.2, 133.4, 129.9, 128.6, 69.1, 54.2, 21.8 [12]

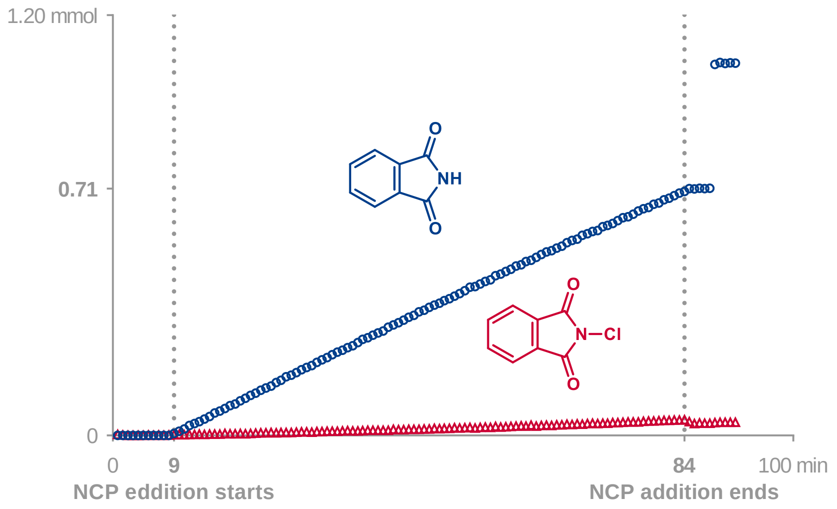

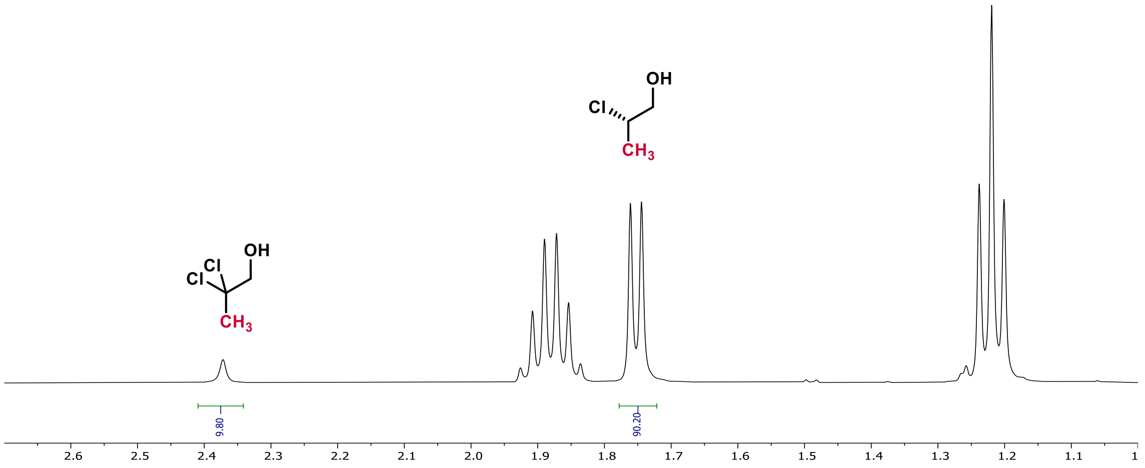

Figure 38 – The α-chlorination of propanal displays a good yield and excellent er.

The FTIR data indicated that almost all NCP added was consumed (0.71 mmol). The crude NMR, taken post-reduction, indicated that the ratio of mono to dichlorinated product was 90:10. As each molecule of dichlorinated product accounts for two molecules of NCP, the monochlorinated product accounts for 82% of the NCP consumed.

$$\text{Yield} = 100\frac{\text{NCP}_{\text{consumed}} \cdot \text{F}_{\text{mono}}}{\text{NCP}_{\text{added}}} = 100\frac{0.71 \cdot 0.82}{0.75} = 78\%$$

The er, measured by chiral HPLC of the benzoylated derivative, was 98:2. (S–108).

10.7.4. δ-Valerolactol

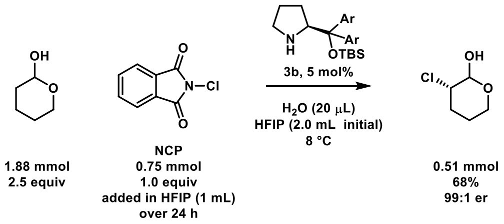

The lactol exists in equilibrium with 5-hydroxypentanal (approx. 10% aldehyde in HFIP determined by ^{1}H NMR) and can also form stable hemiaminal ethers with Jørgensen-Hayashi type catalysts.[11] The formation of these adducts and the low availability of aldehyde meant that the chlorinating agent needed to be added very slowly to ensure complete reaction and avoid catalyst chlorination.

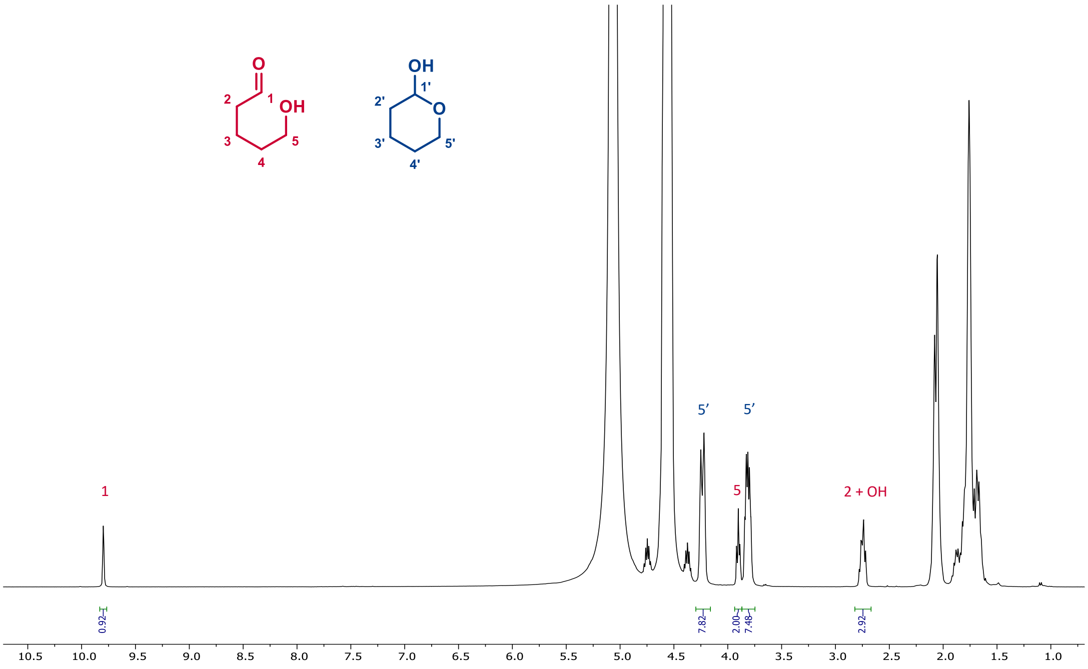

Figure 39 - Lactol/aldehyde equilibrium demonstrated in HFIP.

We attempted the α-chlorination of the lactol at room temperature but found that the product has a disappointing er (90:10). As such, we carried out the reaction at 8 °C, with the aid of an immersion chiller, and used 5 mol% of catalyst to ensure the reaction was completed in a practical amount of time. The following stock solutions in HFIP were used in the preparation of this reaction.

SL	Component	Volume / mL	Mass / mg	Concentration / M
A	δ-valerolactol	1.0	384.9	3.77
B	NCP	2.0	273.1	0.75
C	cat **3b**	1.0	47.9	0.08
D	H_2O	1.0	40.1	2.23
E	1,1,2,2-tetrachloroethane	1.0	125.6	0.75

A vial containing a magnetic stirrer was charged with stock solutions **A** (500 µL, 1.88 mmol, 2.5 equiv), **C** (500 µL, 0.04 mmol, 5 mol%), **D** (500 µL, 1.11 mmol) and **E** (500 µL, 0.38 mmol). Stock solution **B** (1.0 mL, 0.75 mmol, 1.0 equiv) was added using a syringe pump over 24 h. Yield was determined by qNMR (68%, S-79). The product, (3*S*)-3-chlorotetrahydro-2H-pyran-2-ol, was isolated as a colorless oil after preparative TLC. The product obtained after flash-column purification or preparative TLC (9:1 hexane:EtOAc) was racemic, so the er was determined by chiral HPLC after reduction to 2-chloropentane-1,5-diol and subsequent dibenzoyaltion (S–109).

^{1}H NMR (400 MHz, $CDCl_3$) δ (ppm) Cis epimer: 4.90 (d, *J* = 6.8 Hz, 1H), 4.15 – 4.12 (m, 1H), 4.06 – 4.00 (m, 1H), 3.62 – 3.54 (m, 1H), 3.08 (d, *J* = 8.3 Hz, 1H), 2.25 – 2.18 (m, 1H), 2.07 – 2.00 (m, 1H), 1.97 – 1.86 (m, 1H), 1.68 – 1.50 (m, 1H). Trans epimer: 4.79 (d, *J* = 4.9 Hz, 1H), 4.06 – 4.00 (m, 1H), 3.78 – 3.74 (m, 1H), 3.62 – 3.54 (m, 1H), 2.94 (s, 1H), 2.38 – 2.31 (m, 1H), 1.97 – 1.86 (m, 2H) 1.68 – 1.50 (m, 1H).

^{13}C NMR (125 MHz, $CDCl_3$) δ (ppm) 97.3, 93.0, 64.3, 63.6, 60.2, 58.6, 31.0, 29.5, 24.1, 22.1.[13]

10.7.5. Pentanal

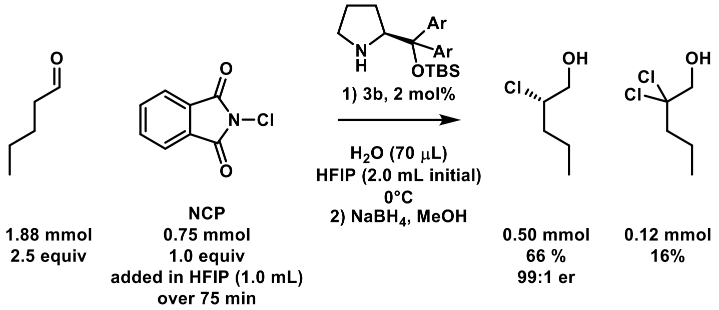

Optimization for pentanal began with the conditions used for propanal. Less water was used to ensure a high enantiomeric excess. The following stock solutions were used for this reaction.

SL	Component	Volume / mL	Mass / mg	Concentration / M
**A**	Pentanal	1.0	323.1	3.75
**B**	NCP	2.0	272.9	0.75
**C**	cat **3b**	1.0	19.4	0.03
**D**	H_2O	1.0	142.9	7.94
**E**	Phthalimide	1.0	112.6	0.75

A vial containing 500 µL of HFIP, a magnetic stirrer and fitted with the ReactIR probe was charged with stock solutions **A** (500 µL, 1.88 mmol, 2.5 equiv), **C** (500 µL, 0.015 mmol, 2 mol%) and **D** (500 µL, 4.0 mmol). The reaction was cooled to 0 °C using an ice bath, stirred for 5 min and consecutive FTIR spectra of 122 scans were taken by the ReactIR. Stock solution **B** (1.0 mL, 0.75 mmol, 1.0 equiv) was added using a syringe pump over 75 min. After the addition ended, a further 5 data points were collected before stock solution **E** (500 µL, 0.375 mmol) was added for calibration. A final 5 data points were collected, before the reaction mixture was quenched in a stirred solution of $NaBH_4$ (750 mg, 19.8 mmol) in MeOH (5 mL). After stirring for 5 min, brine (5 mL) and H_2O (5 mL) were added. The mixture was extracted with CH_2Cl_2 (6 x 15 mL), before the collected organic phase was washed with brine (15 mL), dried over $MgSO_4$ and concentrated on a rotary evaporator. The product, 2-chloro-pentan-1-ol, was isolated after preparative TLC. The er was determined by chiral HPLC after benzoylation (S–110).

^{1}H NMR (400 MHz, $CDCl_3$) δ (ppm) 4.06 – 4.01 (m, 1H), 3.78 (dd, *J* = 12.0, 3.7 Hz, 1H), 3.66 (dd, *J* = 12.0, 7.1 Hz, 1H), 2.08 (s, br, 1H), 1.75 – 1.69 (m, 2H), 1.61 – 1.51 (m, 1H), 1.49 – 1.40 (m, 1H), 0.94 (t, *J* = 7.3 Hz, 3H). **^{13}C NMR (101 MHz, $CDCl_3$)** δ (ppm) 67.2, 65.2, 36.4, 19.7, 13.7. [14]

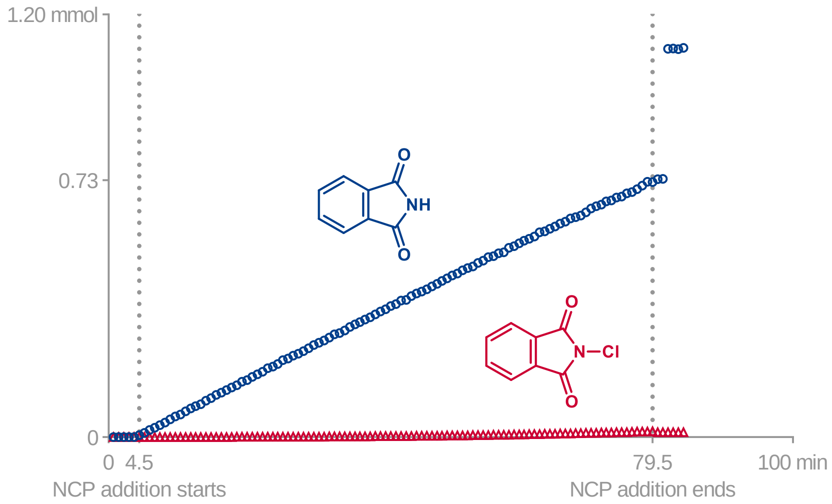

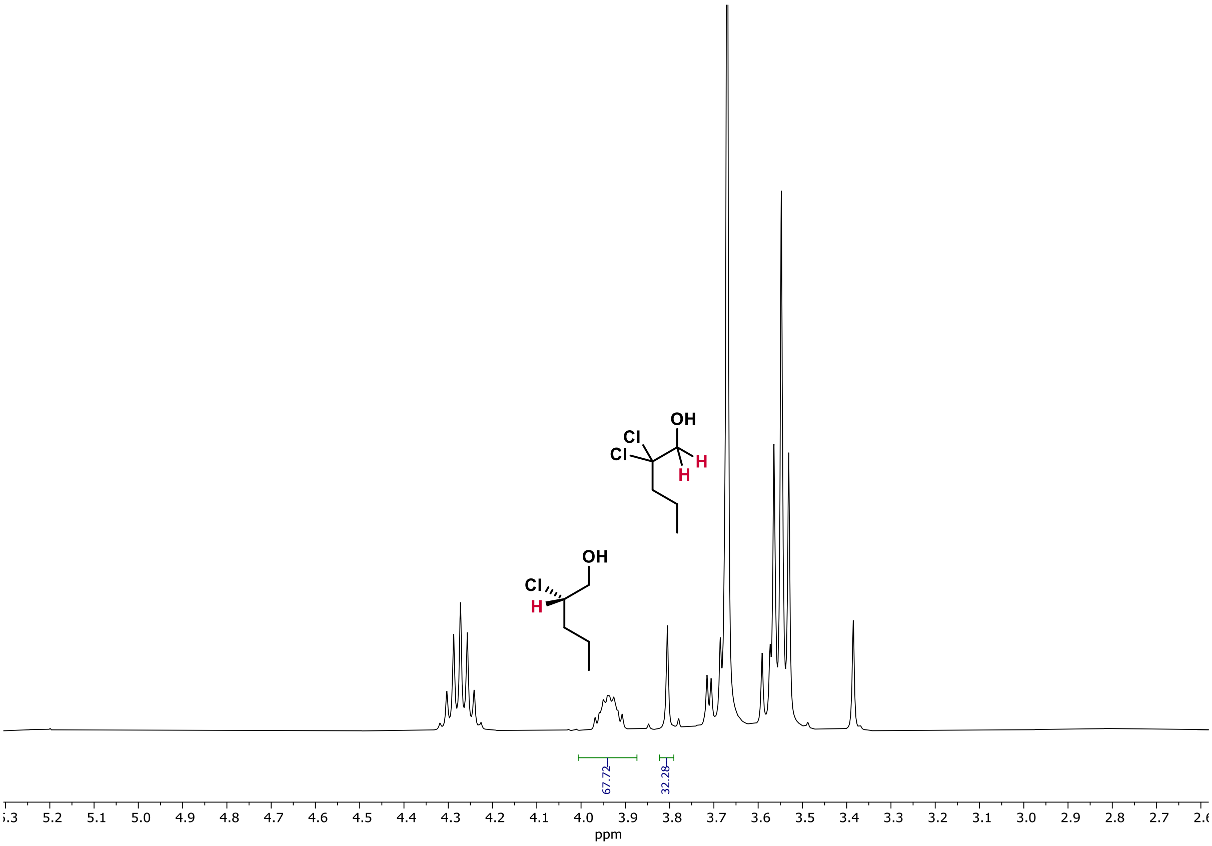

Figure 40 – The α-chlorination of pentanal displays a good yield and excellent er

The FTIR data indicated that all NCP added was consumed (0.73 mmol). The crude NMR, taken post-reduction, indicated that the ratio of mono to dichlorinated product was 68:16. As each molecule of dichlorinated product accounts for two molecules of NCP, the monochlorinated product accounts for 68% of the NCP consumed.

$$\text{Yield} = 100\frac{\text{NCP}_{\text{consumed}} \cdot \text{F}_{\text{mono}}}{\text{NCP}_{\text{added}}} = 100\frac{0.73 \cdot 0.68}{0.75} = 66\%$$

The er, measured by chiral HPLC of the benzoylated derivative, was 99:1 (S–110).

10.7.6. 5-Bromopentanal

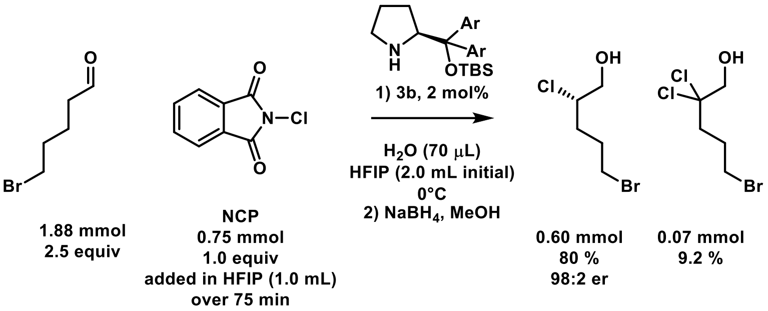

The conditions were identical to those used for pentanal. The following stock solutions were used for this reaction.

SL	Component	Volume / mL	Mass / mg	Concentration / M
**A**	5-bromopentanal	1.0	634.3	3.78
**B**	NCP	2.0	272.0	0.75
**C**	cat **3b**	1.0	19.6	0.03
**D**	H_2O	1.0	141.8	7.94
**E**	Phthalimide	1.0	106.1	0.72

A vial containing 500 µL of HFIP, a magnetic stirrer and fitted with the ReactIR probe was charged with stock solutions **A** (500 µL, 1.89 mmol, 2.5 equiv), **C** (500 µL, 0.015 mmol, 2 mol%) and **D** (500 µL, 4.0 mmol). The reaction was cooled to 0 °C using an ice bath, stirred for 5 min and consecutive FTIR spectra of 122 scans were taken by the ReactIR. Stock solution **B** (1.0 mL, 0.75 mmol, 1.0 equiv) was added using a syringe pump over 75 min. After the addition ended, a further 5 data points were collected before stock solution **E** (500 µL, 0.36 mmol) was added for calibration. A final 5 data points were collected, before the reaction mixture was quenched in a stirred solution of $NaBH_4$ (750 mg, 19.8 mmol) in MeOH (5 mL). After stirring for 5 min, brine (5 mL) and H_2O (5 mL) were added. The mixture was extracted with CH_2Cl_2 (6 x 15 mL), before the collected organic phase was washed with brine (15 mL), dried over $MgSO_4$ and concentrated on a rotary evaporator. The product, 2-chloro-5-bromopentan-1-ol, was isolated after preparative TLC. The er was determined by chiral HPLC after benzoylation (S–111).

^{1}H NMR (400 MHz, $CDCl_3$) δ (ppm) 4.06 – 4.00 (m, 1H), 3.80 (dd, *J* = 12.0, 4.0 Hz, 1H), 3.70 (dd, *J* = 12.0, 6.7 Hz, 1H), 3.44 (t, *J* = 6.3 Hz, 2H), 2.20 – 2.09 (m, 1H), 2.05 – 1.94 (m, 3H), 1.90 – 1.78 (m, 1H). **^{13}C NMR (101 MHz, $CDCl_3$)** δ (ppm) 67.1, 64.1, 33.0, 32.8, 29.5. [7]

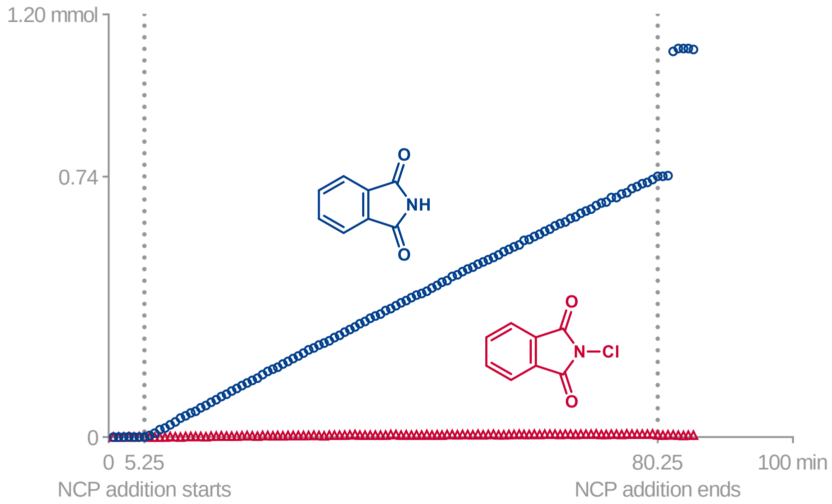

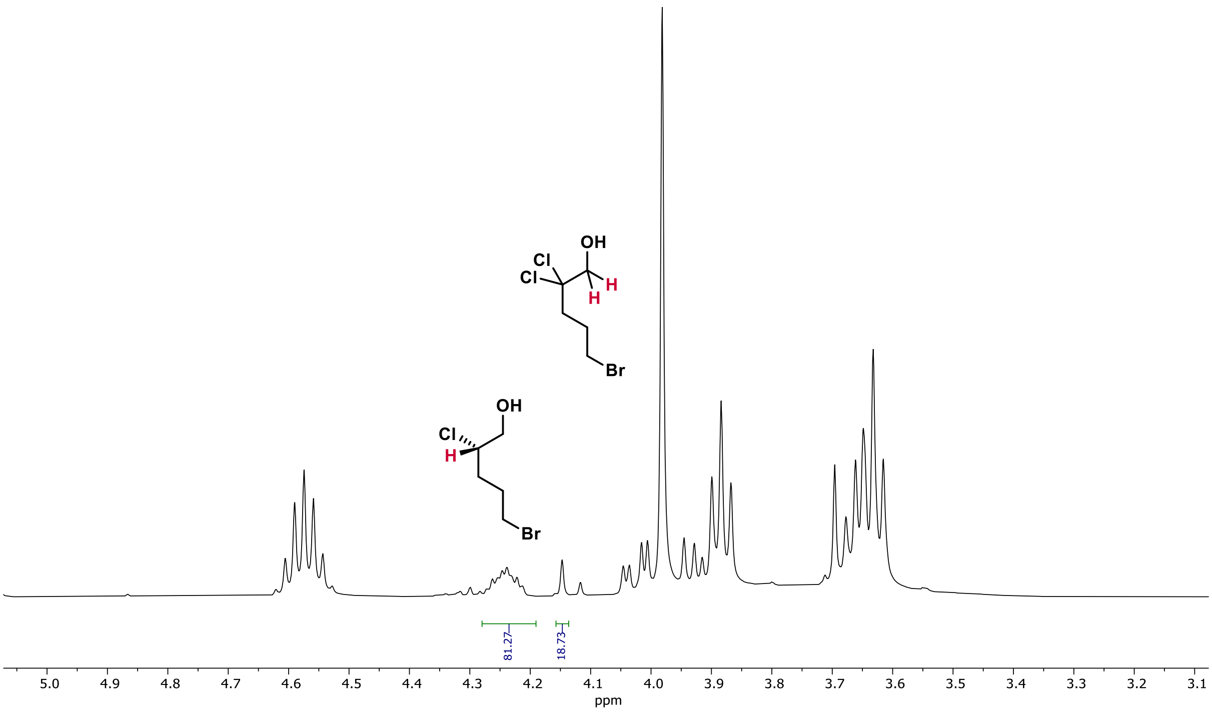

Figure 41 – The α-chlorination of 5-bromopentanal displays a good yield and er

The FTIR data indicated that all NCP added was consumed (0.74 mmol). The crude NMR, taken post-reduction, indicated that the ratio of mono to dichlorinated product was 81:9.5. As each molecule of dichlorinated product accounts for two molecules of NCP, the monochlorinated product accounts for 81% of the NCP consumed.

$$\text{Yield} = 100\frac{\text{NCP}_{\text{consumed}} \cdot \text{F}_{\text{mono}}}{\text{NCP}_{\text{added}}} = 100\frac{0.74 \cdot 0.81}{0.75} = 80\%$$

The er, measured by chiral HPLC of the benzoylated derivative, was 98:2 (S–111).

10.7.7. Dodecanal

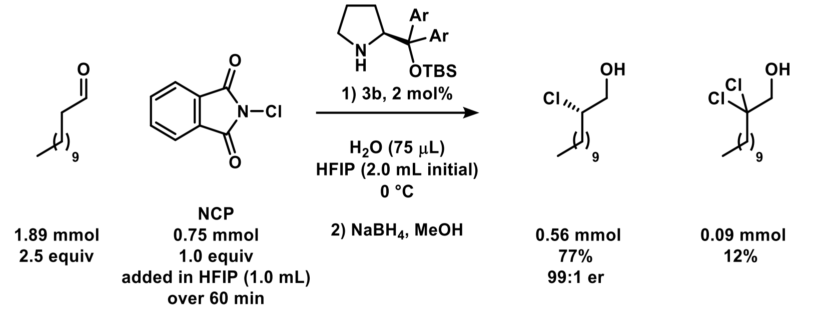

Optimization of conditions began with those identified for octanal. The following stock solutions were used for this reaction.

SL	Component	Volume / mL	Mass / mg	Concentration / M
**A**	Dodecanal	1.0	685.0	3.78
**B**	NCP	2.0	272.6	0.75
**C**	cat **3b**	1.0	19.6	0.03
**D**	H_2O	1.0	148.4	7.94
**E**	Phthalimide	1.0	106.5	0.72

A vial containing 500 μL of HFIP, a magnetic stirrer and fitted with the ReactIR probe was charged with stock solutions **A** (500 μL, 1.89 mmol, 2.5 equiv), **C** (500 μL, 0.015 mmol, 2 mol%) and **D** (500 μL, 4.0 mmol). The reaction was cooled to 0 °C using an ice bath, stirred for 5 min and consecutive FTIR spectra of 122 scans were taken by the ReactIR. Stock solution **B** (1.0 mL, 0.75 mmol, 1.0 equiv) was added using a syringe pump over 60 min. After the addition ended, a further 7 data points were collected before stock solution **E** (500 μL, 0.36 mmol) was added for calibration. A final 5 data points were collected, before the reaction mixture was quenched in a stirred solution of $NaBH_4$ (750 mg, 19.8 mmol) in MeOH (5 mL). After stirring for 5 min, brine (5 mL) and H_2O (5 mL) were added. The mixture was extracted with CH_2Cl_2 (6 x 15 mL), before the collected organic phase was washed with brine (15 mL), dried over $MgSO_4$ and concentrated on a rotary evaporator. The product, 2-chlorododecan-1-ol, was isolated after preparative TLC. The er was determined by chiral HPLC after benzoylation (S–112).

^{1}H NMR (400 MHz, $CDCl_3$) δ (ppm) 4.04 – 3.98 (m, 1H), 3.77 (dd, *J* = 12.0, 3.8 Hz, 1H), 3.65 (dd, *J* = 12.0, 7.1 Hz, 1H), 2.21 (s, br, 1H), 1.80 – 1.64 (m, 2H), 1.57 – 1.20 (m, 18H), 0.87 (t, *J* = 6.8 Hz, 3H). **^{13}C NMR (101 MHz, $CDCl_3$)** δ (ppm) 67.1, 65.5, 34.4, 32.0, 29.7, 29.7, 29.6, 29.4, 29.2, 26.5, 22.8, 14.2. [15]

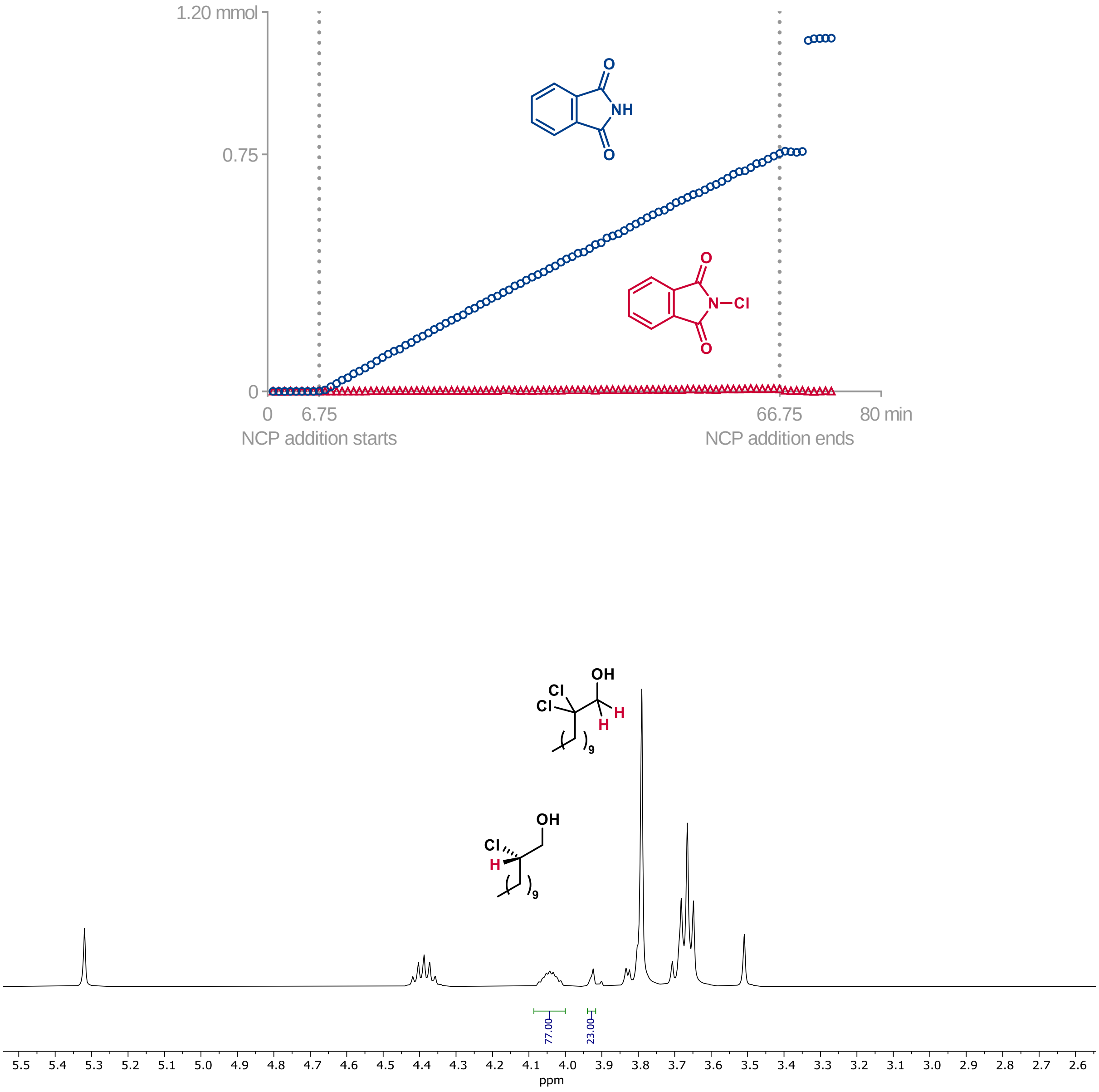

Figure 42 – The α-chlorination of dodecanal displays a good yield and er

The FTIR data indicated that all NCP added was consumed (0.75 mmol). The crude NMR, taken post-reduction, indicated that the ratio of mono to dichlorinated product was 77:11.5. As each molecule of dichlorinated product accounts for two molecules of NCP, the monochlorinated product accounts for 77% of the NCP consumed.

$$\text{Yield} = 100\frac{\text{NCP}_{\text{consumed}} \cdot \text{F}_{\text{mono}}}{\text{NCP}_{\text{added}}} = 100\frac{0.75 \cdot 0.77}{0.75} = 77\%$$

The er, measured by chiral HPLC of the benzoylated derivative, was 99:1 (S–112).

11. The calibration of FTIR data

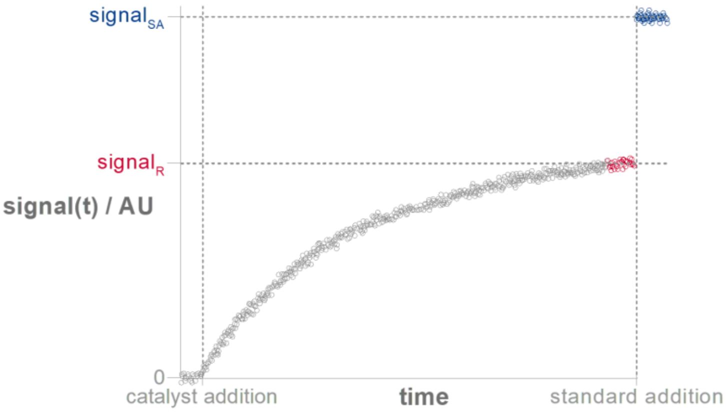

Figure 43 – FTIR data highlights the key values required for standard addition calibration.

We adapted the standard addition methodology used to calibrate FTIR data from our previous work[16]

The standard addition calibration relies on the fact that although the response factor (RF) may change in absolute value between reaction runs, the signal for the standard (S) is still proportional to the concentration of S:

$$\mathrm{signal}(t) = ([\mathrm{S}]_t \cdot \mathrm{RF_S})$$

The amount of standard present after the standard addition, ($[S]_{SA} \cdot (V_{SA} + V_R)$), must be equal to the amount present at the end of the reaction, ($[S]_R \cdot V_R$) plus that added in the standard addition (n_{SA}):

$$([\mathrm{S}]_{\mathrm{SA}} \cdot \mathrm{V_{SA}}) = ([\mathrm{S}]_{\mathrm{E}} \cdot \mathrm{V_E}) + \mathrm{n_{SA}}$$

Substituting gives the response factor of the standard (RF_S) in terms of known values:

$$\mathrm{RF_S} = \frac{\big((\mathrm{V_{SA}}) \cdot \mathrm{signal_{SA}}\big) - (\mathrm{V_R} \cdot \mathrm{signal_R})}{\mathrm{n_{SA}}}$$

Therefore, the concentration of standard present at each time point can be calculated:

$$[\mathrm{S}]_t = \frac{\mathrm{signal}(t) \cdot \mathrm{n_{SA}}}{\big((\mathrm{V_R} + \mathrm{V_{SA}}) \cdot \mathrm{signal_{SA}}\big) - (\mathrm{V_R} \cdot \mathrm{signal_R})}$$

If the substance chosen as the standard accurately represents the advance of the reaction, $[S]_t=[P]_t$, where P is the product. In these formulas, $signal_{SA}$ is the signal height after the standard addition, $signal_R$ is the signal height at the end of the reaction, n_{SA} is the amount of standard added, V_R is the volume of the reaction and V_{SA} is the volume of standard solution added.

Since the chlorination reaction requires a slow addition, the volume of the reaction is not constant. We therefore adapted the derivation accordingly. We decided to use amounts, not concentrations, as we found it more intuitive to work with, and used phthalimide (NHP) as the standard.

$$\mathrm{mmol}_{\mathrm{NHP}}(t) = \frac{\mathrm{signal}(t) \cdot (\mathrm{V}_0 + \mathrm{r}_{\mathrm{add}} \cdot t) \cdot \mathrm{n}_{\mathrm{SA}}}{\left(\mathrm{signal}_{\mathrm{SA}} \cdot (\mathrm{V}_0 + \mathrm{r}_{\mathrm{add}} \cdot \mathrm{t}_{\mathrm{f}} + \mathrm{V}_{\mathrm{SA}})\right) - \left(\mathrm{signal}_{\mathrm{R}} \cdot (\mathrm{V}_0 + \mathrm{r}_{\mathrm{add}} \cdot \mathrm{t}_{\mathrm{f}})\right)}$$

Where V_0 is the volume at the start of the reaction, r_{add} is the rate of addition and t_f is the total time of addition. The amount calculated for phthalimide at the end of addition was deducted from the total amount of NCP added, giving a value for the final amount of NCP present.

13. Method and data for the assessment of yield by NMR

General procedure: After the addition of chlorinating agent had ceased, the reaction mixture was added to a stirred solution of $NaBH_4$ (750 mg, 19.8 mmol) in MeOH (10 mL). After stirring for 5 min, brine (5 mL) and H_2O (5 mL) were added successively. A stock solution of trimethoxybenzene (TMB, 250 µL, 0.125mmol) in HFIP (0.5 M) was then added. The reaction mixture was extracted with CH_2Cl_2 (5 x 25 mL), and the combined organic phases were collected, washed with brine, dried over $MgSO_4$ and concentrated on a rotary evaporator, minimizing product loss. Results are tabulated below, and all are in good agreement with the calculated FTIR yield. Differences between the FTIR and NMR yields might be because of product remaining in the aqueous phase during work up.

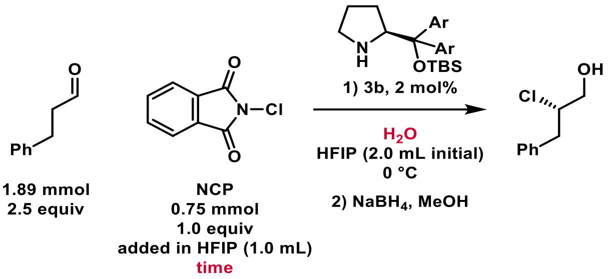

Deviation from above	Yield by NMR / %[a]	Yield by FTIR / %[a]
none	88	85
NCS instead of NCP	82	84
cat **3a** instead of cat **3b**	68	70
1 mol% of cat **3b**[b]	79	85
rt	87	91
0.76 mmol of hydrocinnamaldehyde[c]	62	68
octanal	71	76
isovaleraldehyde[d]	78	80
propanal	75	78
δ-valerolactol[e]	68	N/A
pentanal	65	66
5-bromopentanal	74	80
dodecanal	77	77

[a]Calculated with respect to the limiting reagent. [b]0.76 mmol of phthalimide added before the beginning of the reaction. [c]0.90 mmol (1.2 equiv) of NCP infused in 1.2 mL HFIP. [d]Reaction run at room temperature and 80% of the normal scale. [e]5 mol% of catalyst was used and the reaction was run at 8 °C.

Yields were then calculated using the following formula, taking an average integration of two protons in the product molecule, where possible, against the internal standard signal.

$$\text{Yield by NMR} = \frac{[\text{IS}] \cdot \text{v}_{\text{IS}} \cdot \text{n}_{\text{IS}} \cdot \text{signal(mCl)}}{[\text{NCP}] \cdot \text{v}_{\text{NCP}}}$$

where:

$[\text{IS}]$ = concentration of internal standard (M)
v_{IS} = volume of internal standard (mL)
n_{IS} = number of protons in the internal standard signal
signal(mCl) = the average integral for the product when the integral for IS is set to 100
$[\text{NCP}]$ = concentration of NCP solution slowly added (M)
v_{NCP} = volume of NCP solution slowly added (mL)

For reaction 10.1. – Standard reaction conditions

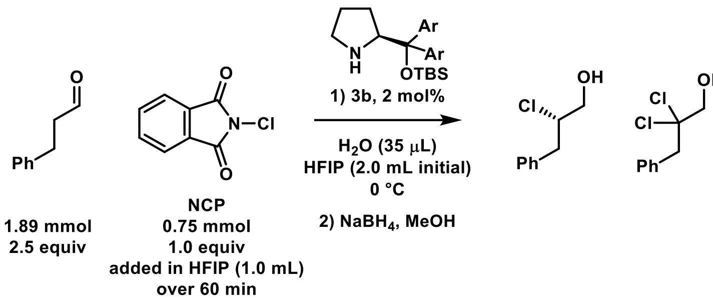

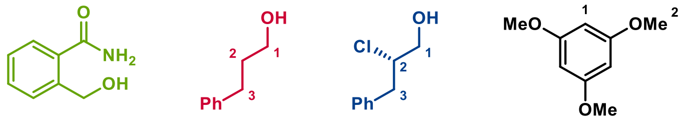

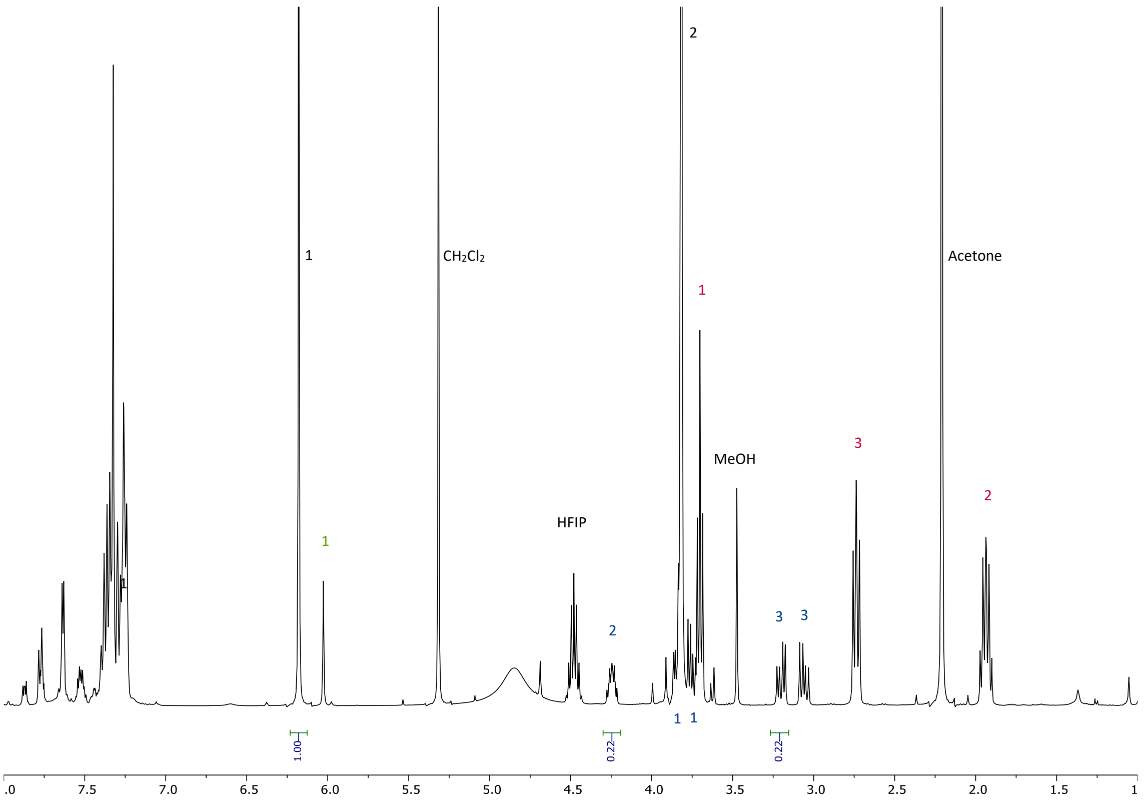

$[\mathrm{IS}] = 2.00\ \mathrm{M}$

$v_{\mathrm{IS}} = 500\ \mu\mathrm{L}$

$n_{\mathrm{IS}} = 3$

$\mathrm{signal(mCl)} = 0.22$

$[\mathrm{NCP}] = 0.75\ \mathrm{M}$

$v_{\mathrm{NCP}} = 1.0\ \mathrm{mL}$

$\mathrm{Yield} = 88\%$

For reaction 10.2. – Reaction with NCS as the chlorinating agent

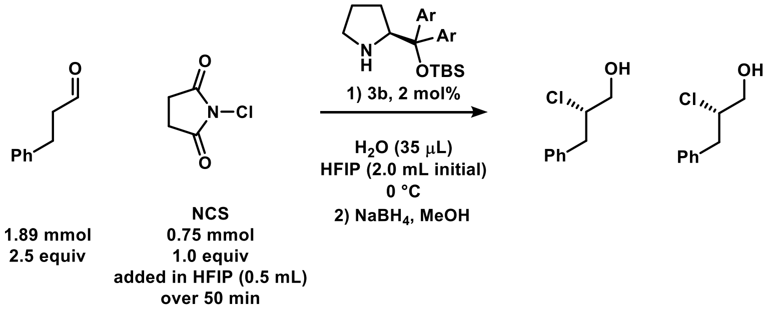

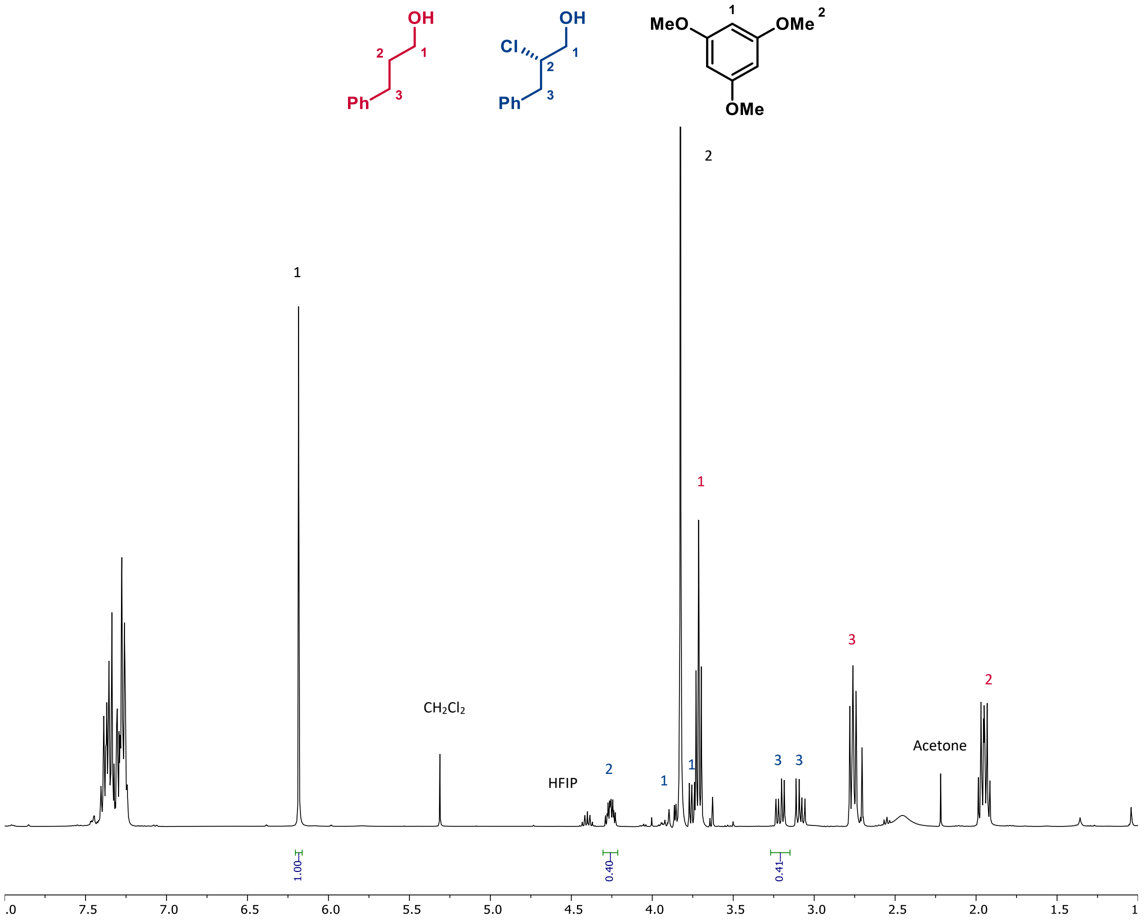

$[IS] = 1.00\ M$

$v_{IS} = 500\ \mu L$

$n_{IS} = 3$

$signal(mCl) = 0.41$

$[NCS] = 1.50\ M$

$v_{NCS} = 0.5\ mL$

$Yield = 82\%$

For reaction 10.3. – With catalyst **3a**

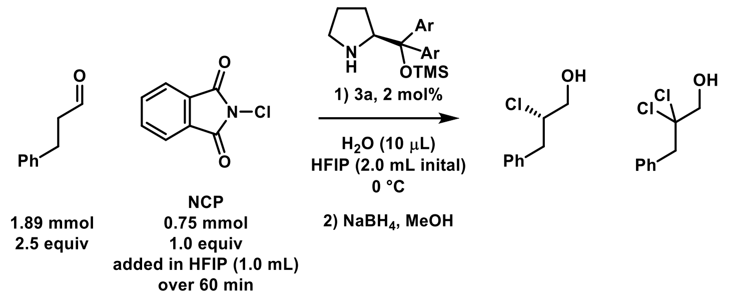

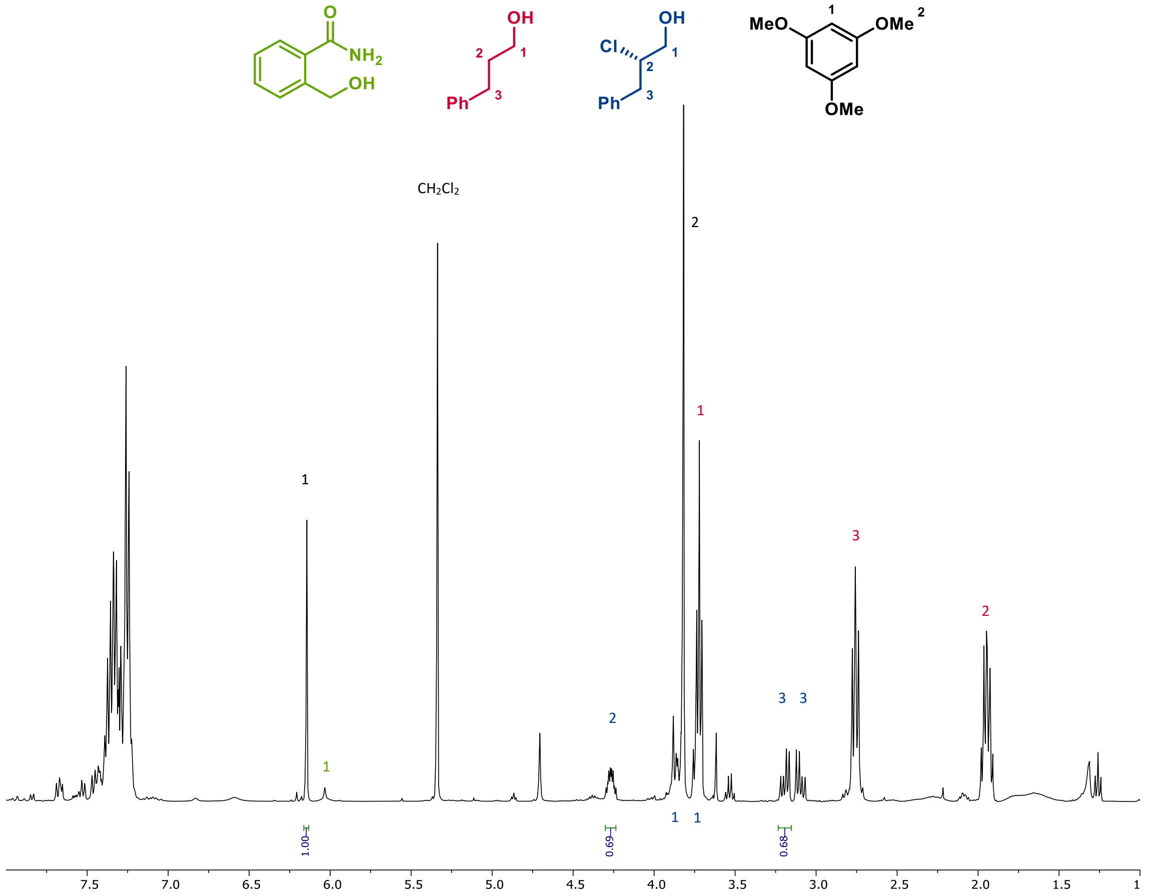

$[IS] = 0.50\ M$

$v_{IS} = 500\ \mu L$

$n_{IS} = 3$

$signal(mCl) = 0.68$

$[NCP] = 0.75\ M$

$v_{NCP} = 1.0\ mL$

$Yield = 68\%$

For reaction 10.4. – Reaction with 1 mol% catalyst loading

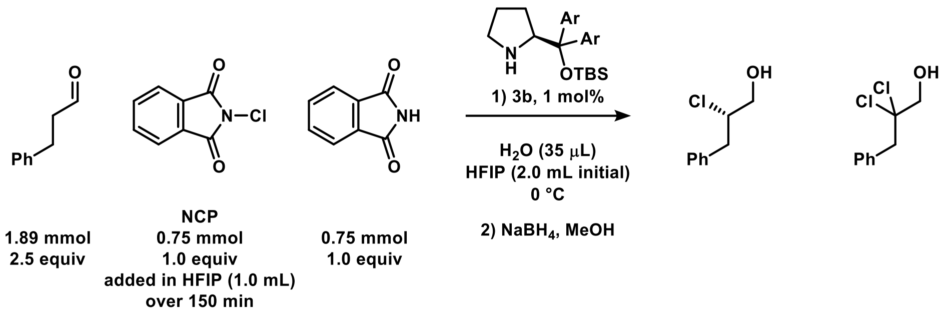

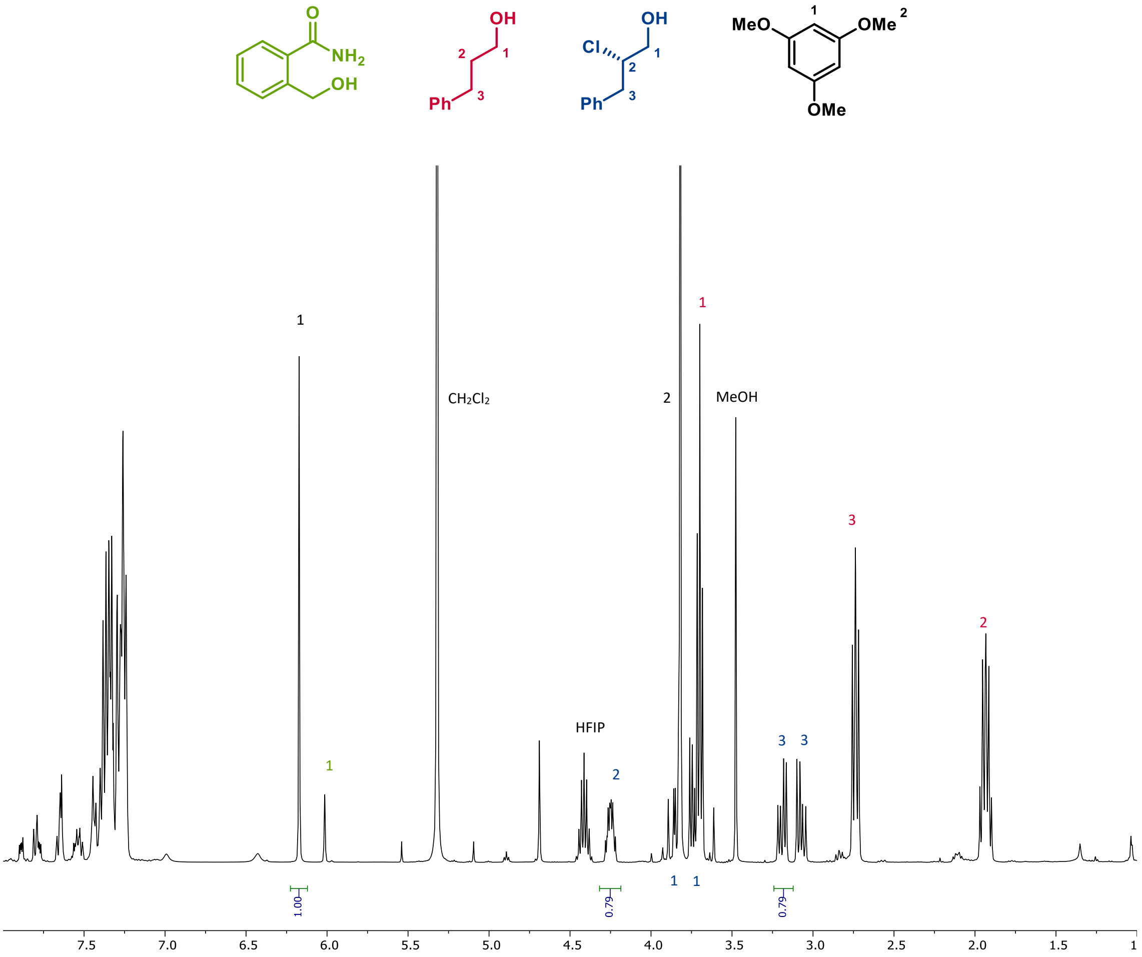

$[IS] = 0.50$ M

$v_{IS} = 500$ μL

$n_{IS} = 3$

signal(mCl) = 0.79

$[NCP] = 0.75$ M

$v_{NCP} = 1.0$ mL

Yield = 79%

Reaction 10.5. – Reaction at room temperature

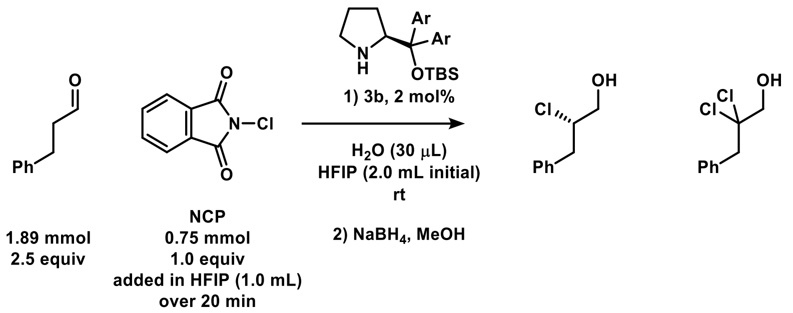

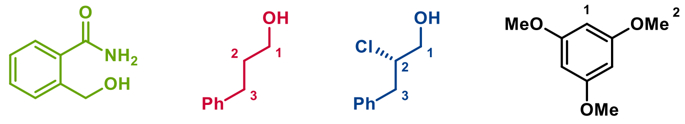

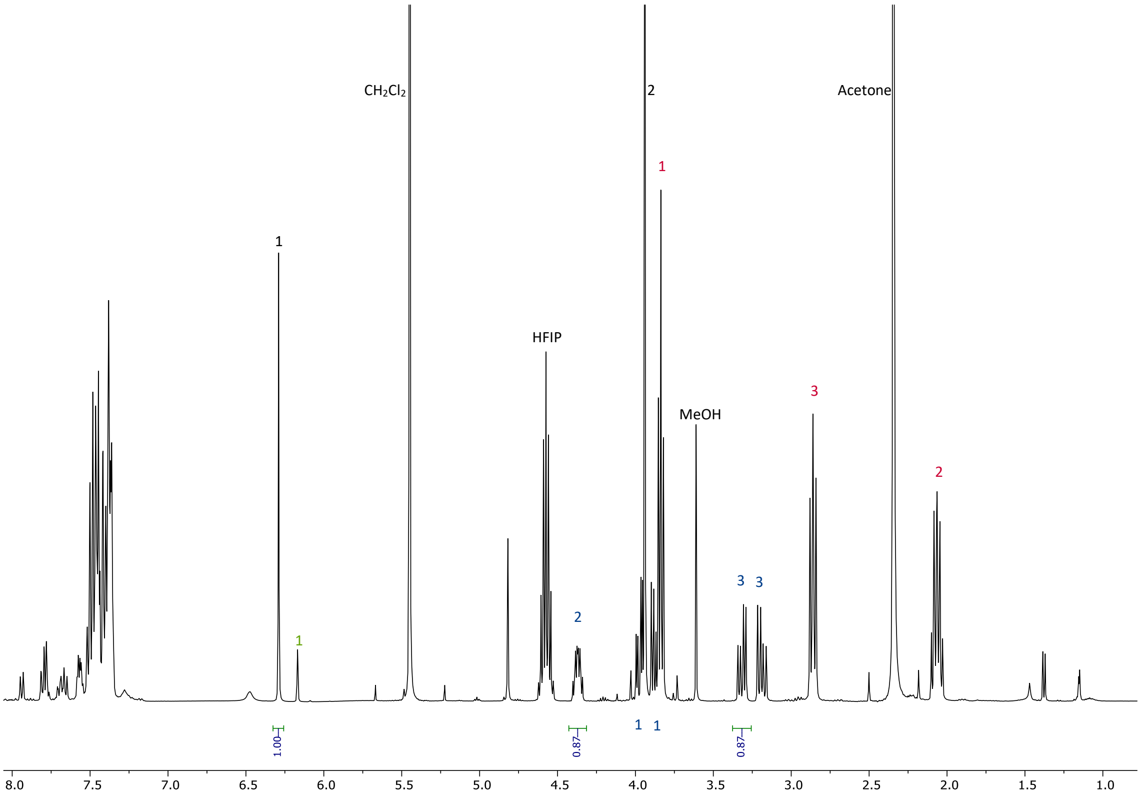

$[IS] = 0.50\ M$

$v_{IS} = 500\ \mu L$

$n_{IS} = 3$

$signal(mCl) = 0.87$

$[NCP] = 0.75\ M$

$v_{NCP} = 1.0\ mL$

$Yield = 87\%$

Reaction 10.6. – Reaction with 1.0 equiv aldehyde

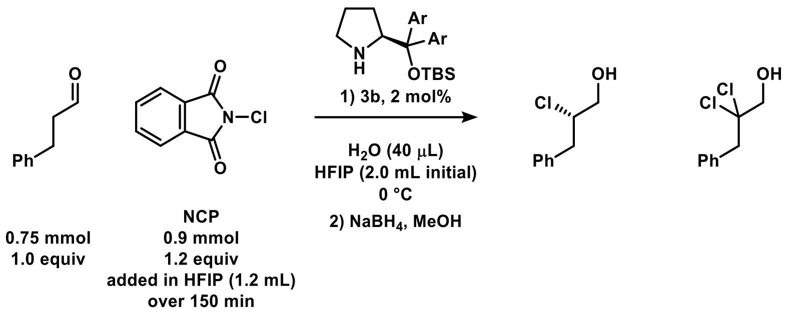

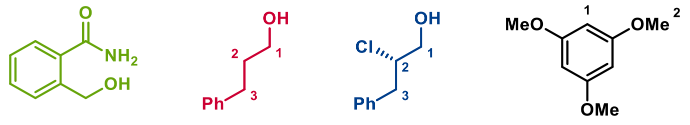

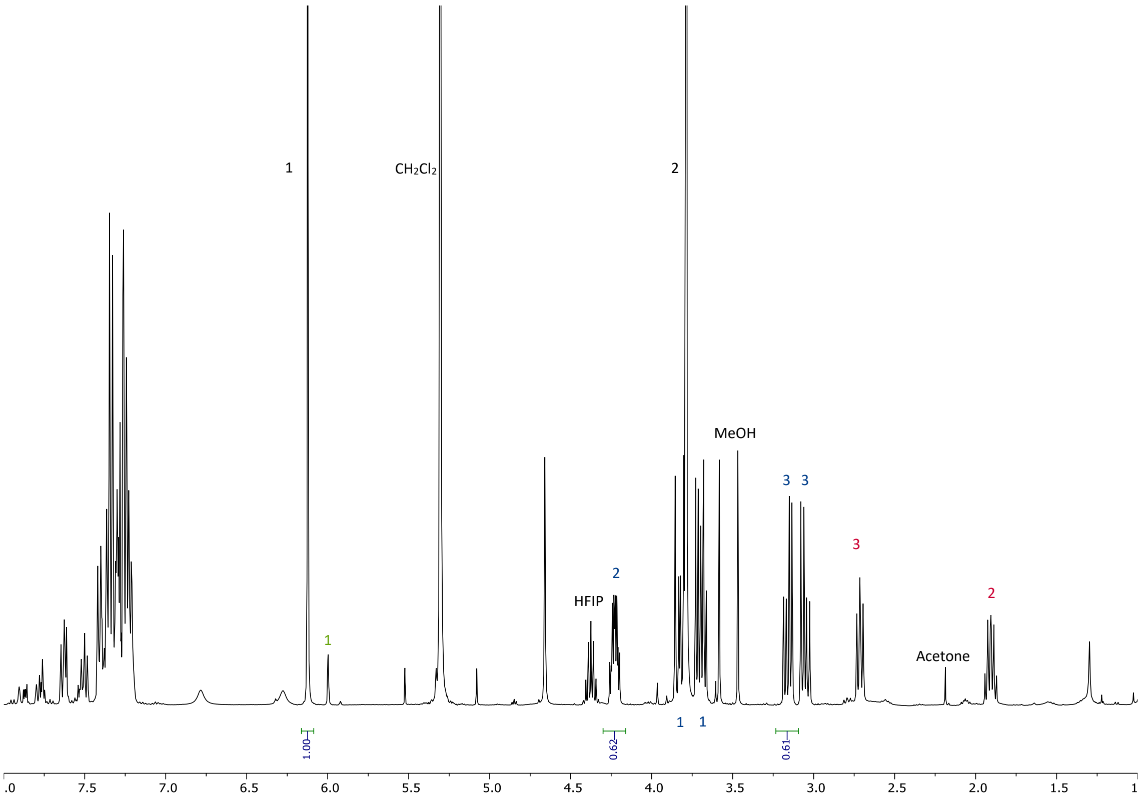

$[\text{IS}] = 0.50\ \text{M}$

$v_{\text{IS}} = 500\ \mu\text{L}$

$n_{\text{IS}} = 3$

$\text{signal(mCl)} = 0.62$

$\text{Maximum yield} = 0.76\ \text{mmol}$

$\text{Yield} = 62\%$

Reaction 10.7.1. – Octanal

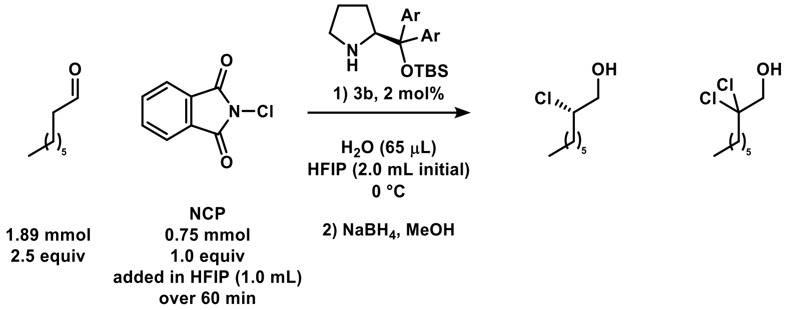

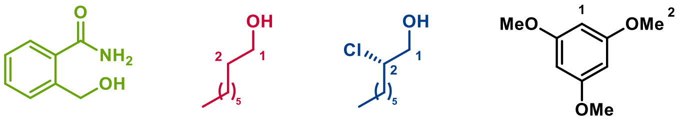

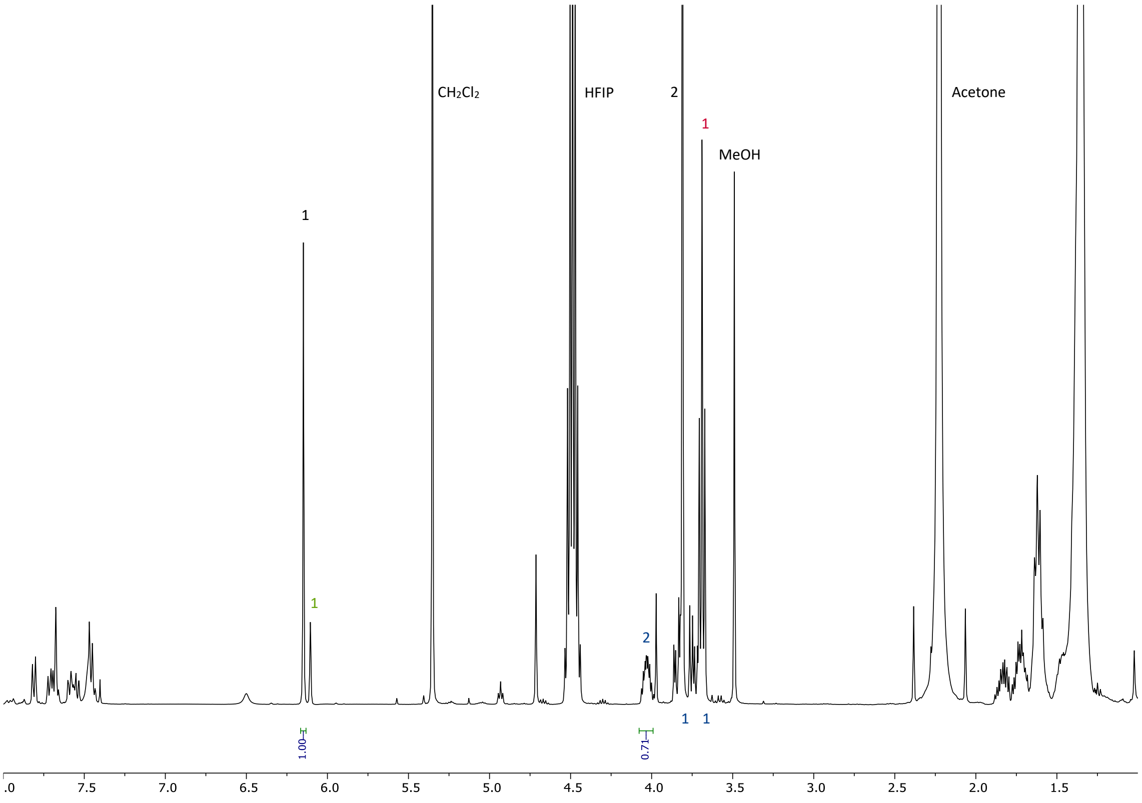

$[IS] = 0.50$ M

$v_{IS} = 500$ µL

$n_{IS} = 3$

signal(mCl) = 0.71

$[NCP] = 0.75$ M

$v_{NCP} = 1.0$ mL

Yield = 71%

Reaction 10.7.2. – Isovaleraldehyde

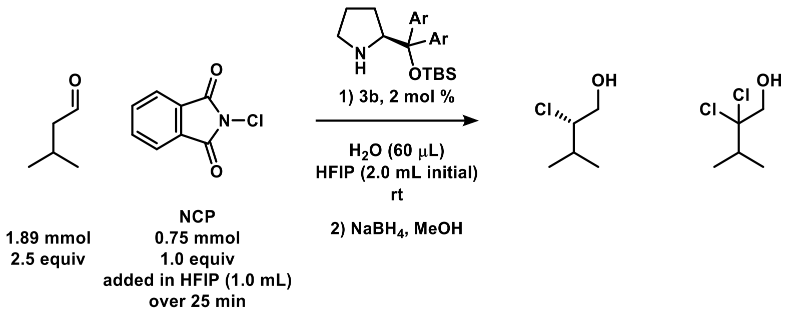

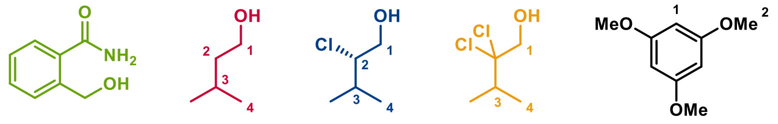

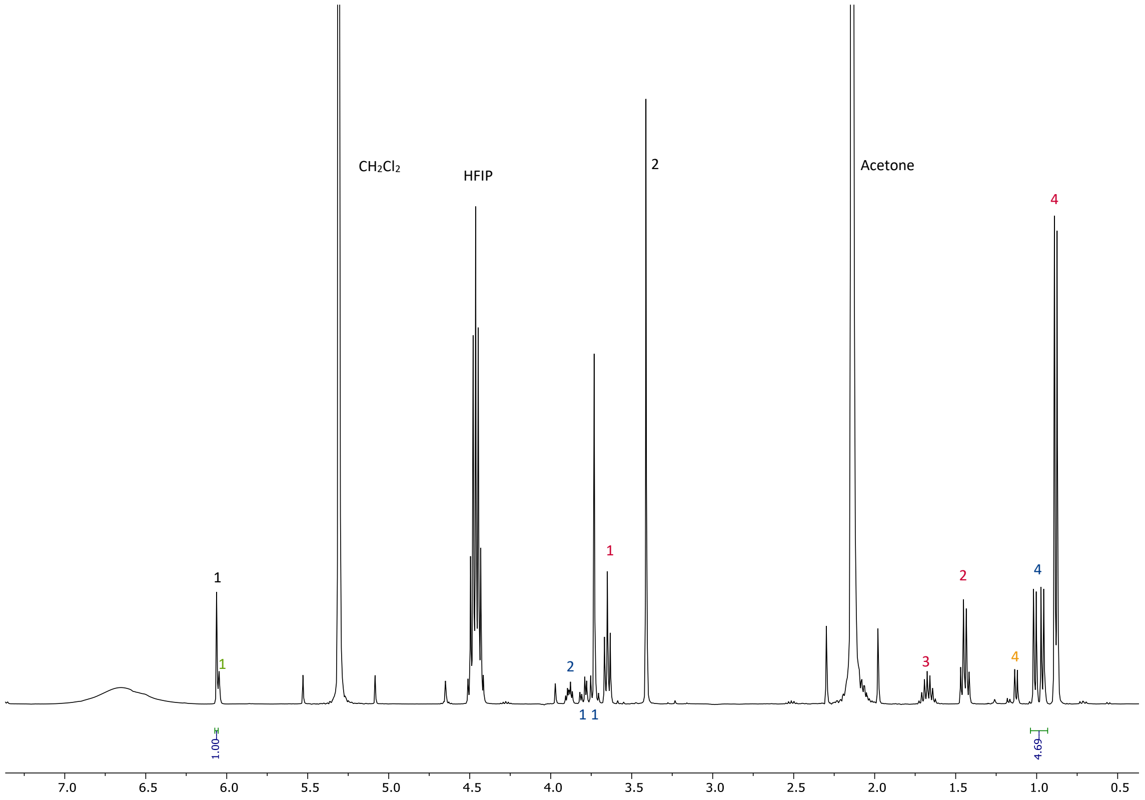

$[\mathrm{IS}] = 0.50\ \mathrm{M}$

$v_{\mathrm{IS}} = 500\ \mu\mathrm{L}$

$n_{\mathrm{IS}} = 3$

$\mathrm{signal(mCl)} = 4.69, 6\ \mathrm{protons}$

$[\mathrm{NCP}] = 0.75\ \mathrm{M}$

$v_{\mathrm{NCP}} = 1.0\ \mathrm{mL}$

$\mathrm{Yield} = 78\%$

Reaction 10.7.3. – Propanal

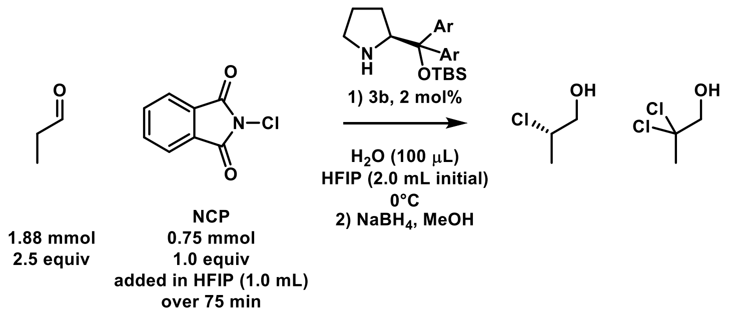

Propanal spectra was taken directly from the MeOH reduction mixture, as the product is water soluble and volatile. Clear peaks corresponding to the CH_3 protons are easily identified for both the monochlorinated and dichlorinated products, in addition to the alcohol of the starting material.

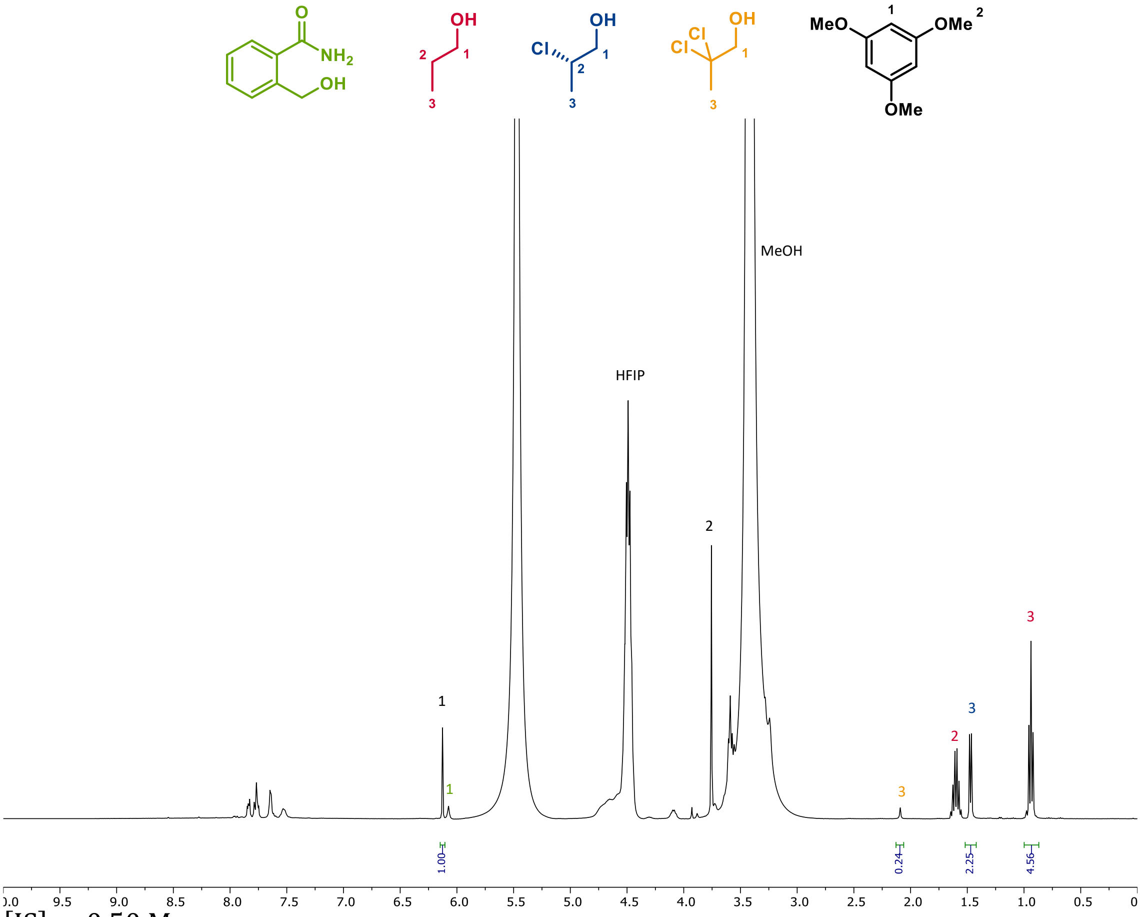

$[IS] = 0.50$ M

$v_{IS} = 500$ µL

$n_{IS} = 3$

signal(mCl) = 2.25 (3 protons)

$[NCP] = 0.75$ M

$v_{NCP} = 1$ mL

Yield = 75%

Reaction 10.7.4 – δ-valerolactol

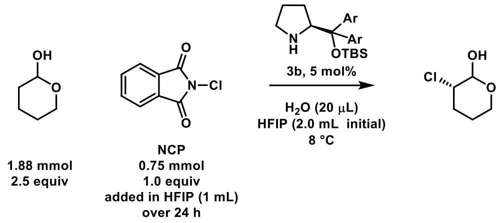

The qNMR was taken directly from the reaction mixture using tetrachloroethane (TCE) as the internal standard. The peaks were assigned using 2D NMR and the results of Rittner *et al.*[13]

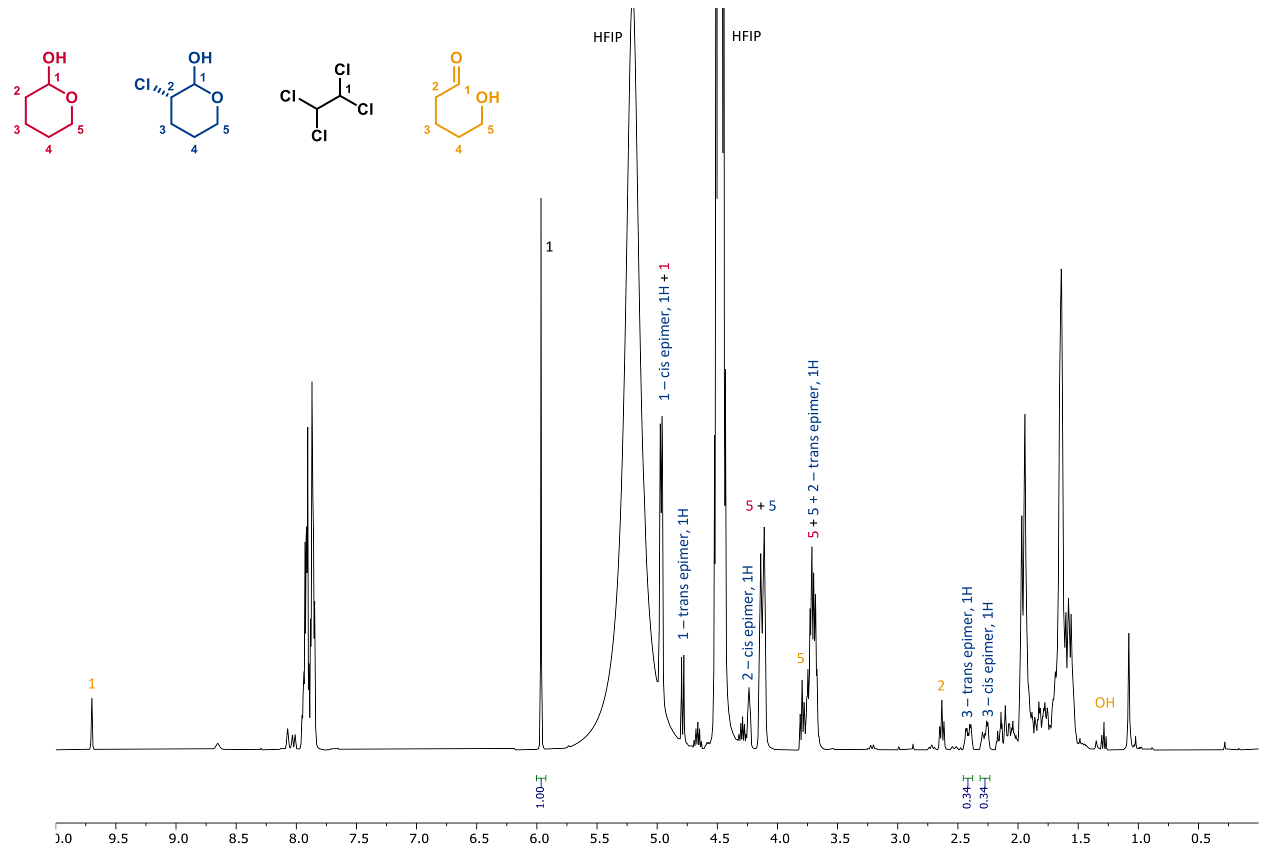

$[\mathrm{IS}] = 0.75\ \mathrm{M}$

$v_{\mathrm{IS}} = 500\ \mu\mathrm{L}$

$n_{\mathrm{IS}} = 2$

$\mathrm{signal(mCl)} = 0.68$

$[\mathrm{NCP}] = 0.75\ \mathrm{M}$

$v_{\mathrm{NCP}} = 1.0\ \mathrm{mL}$

$\mathrm{Yield} = 68\%$

Reaction 10.7.5 – Pentanal

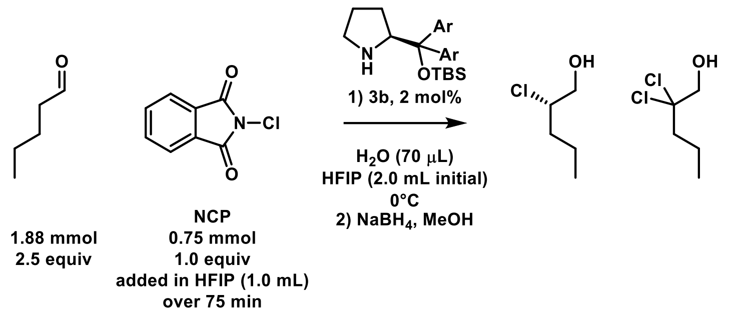

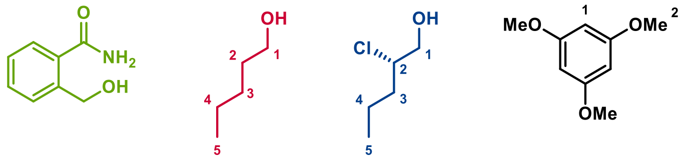

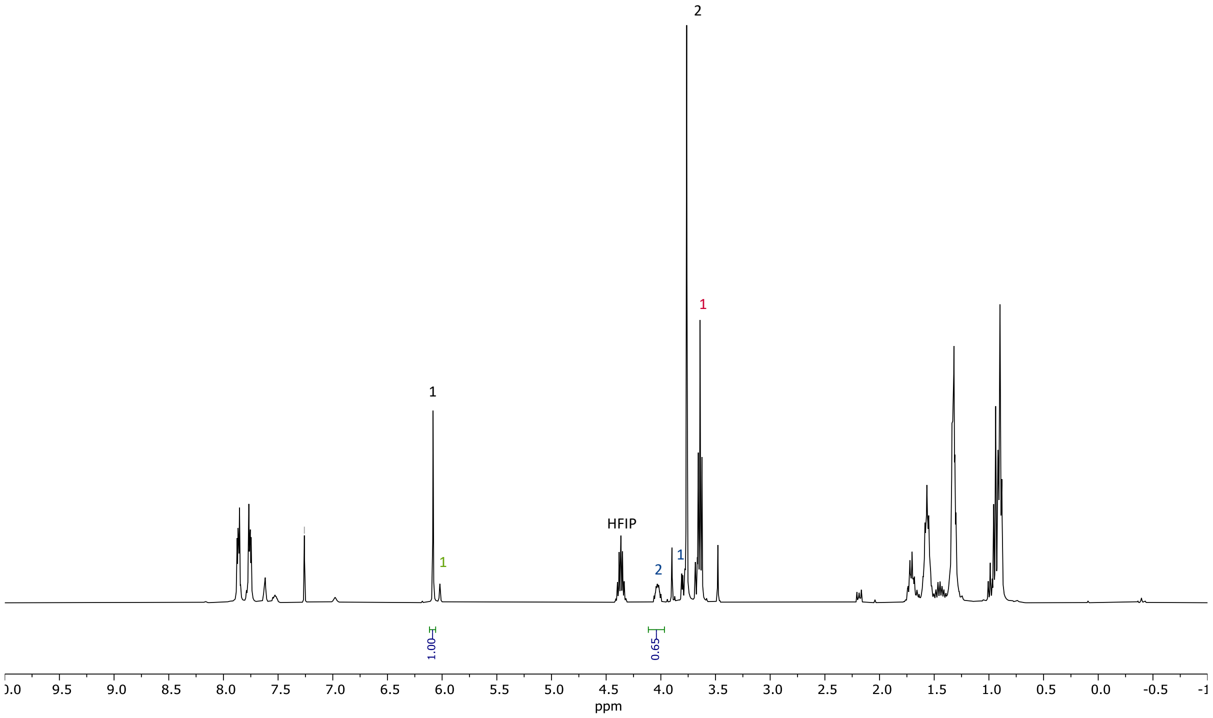

$[IS] = 0.50\ M$

$v_{IS} = 500\ \mu L$

$n_{IS} = 3$

$signal(mCl) = 0.65$

$[NCP] = 0.75\ M$

$v_{NCP} = 1.0\ mL$

$Yield = 65\%$

Reaction 10.7.6 – 5-bromopentanal

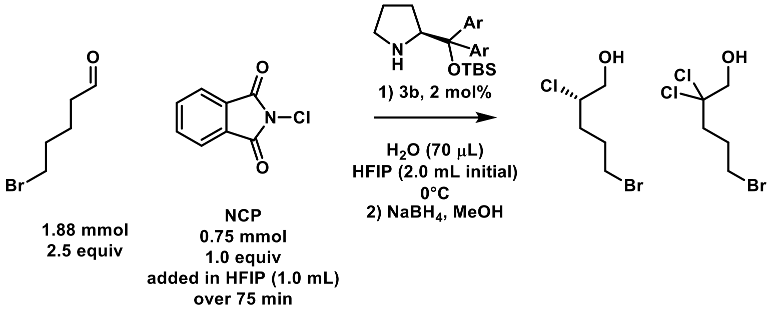

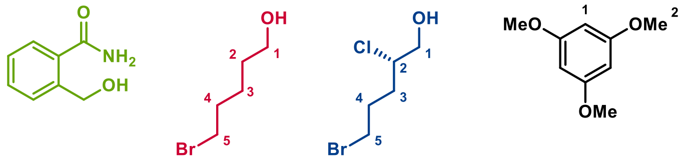

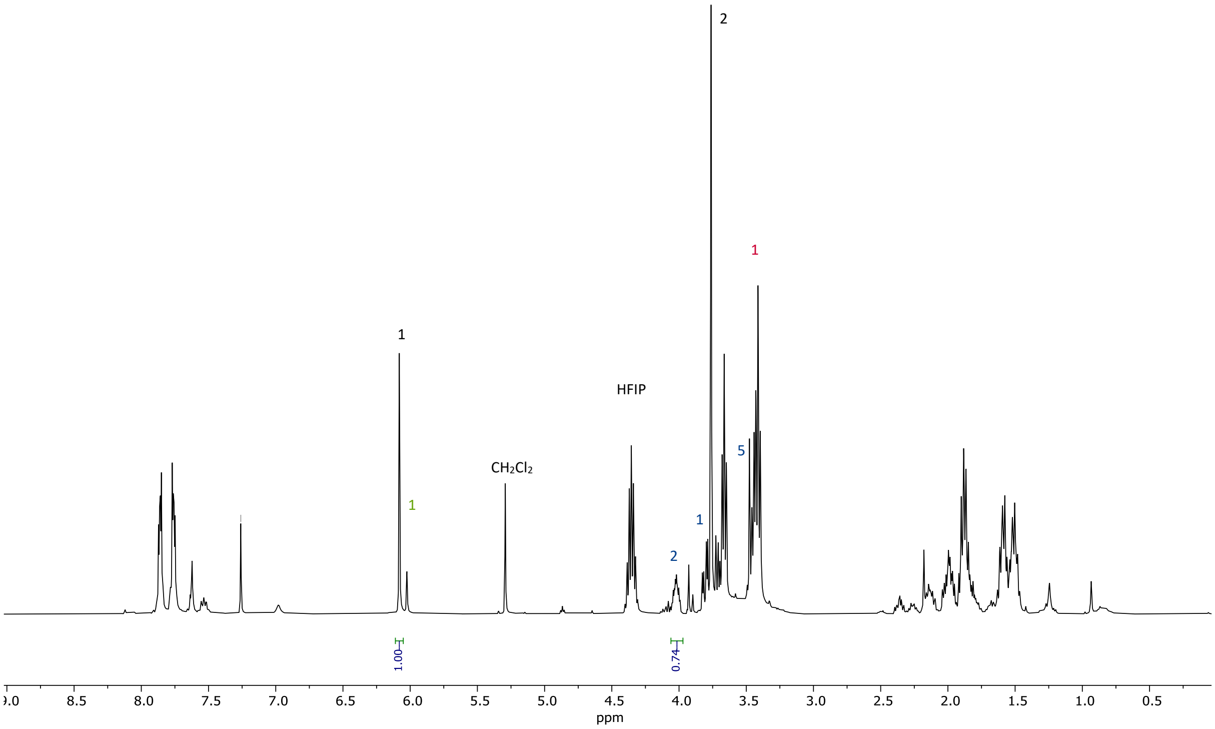

$[IS] = 0.50\ M$

$v_{IS} = 500\ \mu L$

$n_{IS} = 3$

$\text{signal}(mCl) = 0.74$

$[NCP] = 0.75\ M$

$v_{NCP} = 1.0\ mL$

$\text{Yield} = 74\%$

Reaction 10.7.7 – Dodecanal

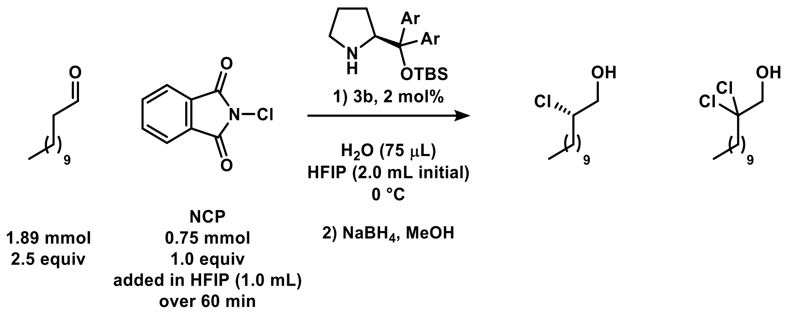

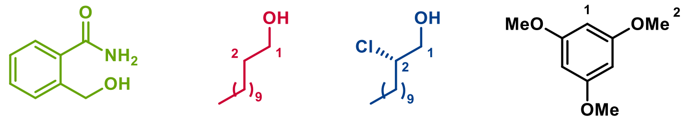

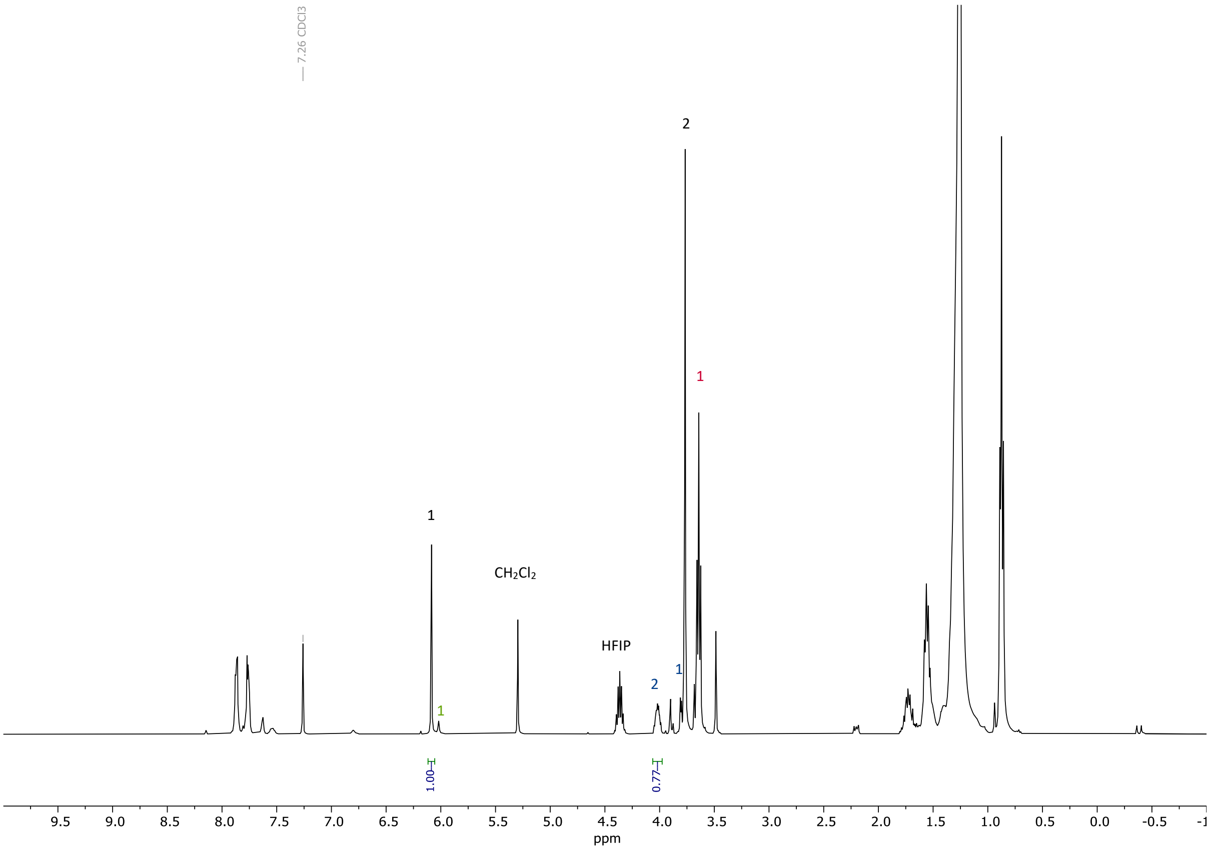

$[IS] = 0.50$ M

$v_{IS} = 500$ μL

$n_{IS} = 3$

signal(mCl) = 0.77

$[NCP] = 0.75$ M

$v_{NCP} = 1.0$ mL

Yield = 77%

13. NMR spectra of enamines and α-chlorinated products

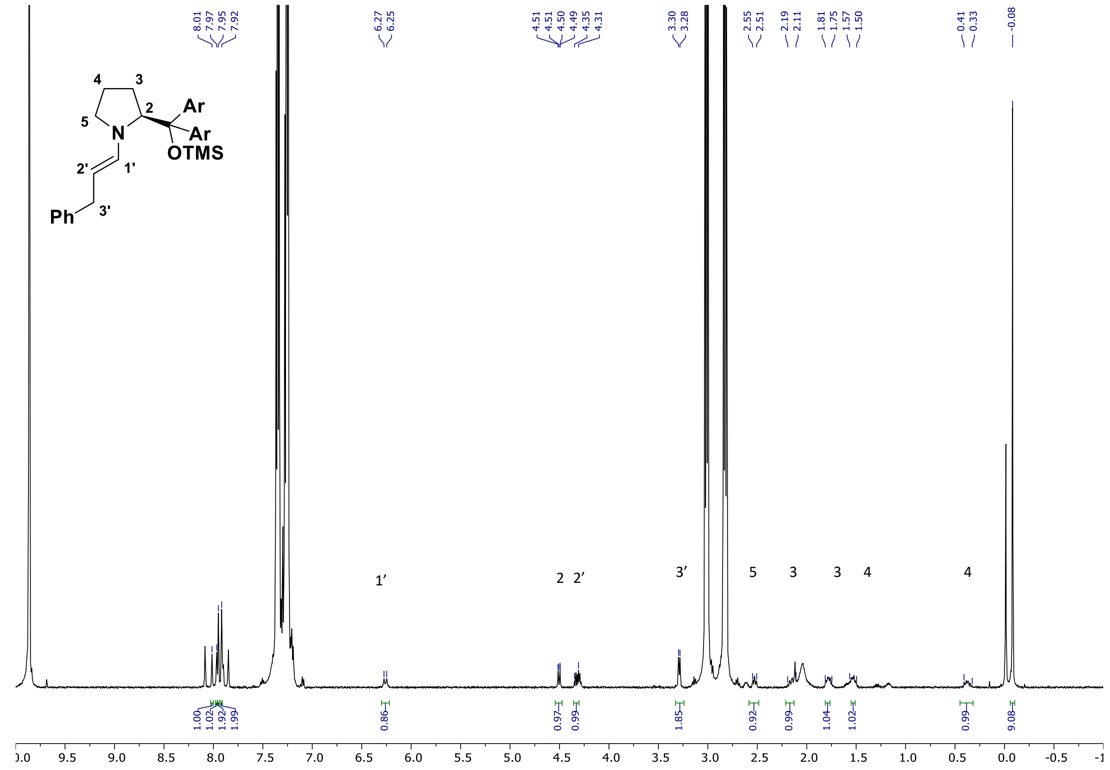

*Figure 44 – ^{1}H NMR (500 MHz, $CDCl_3$) spectrum of the enamine of hydrocinnamaldehyde and catalyst **3a**.*

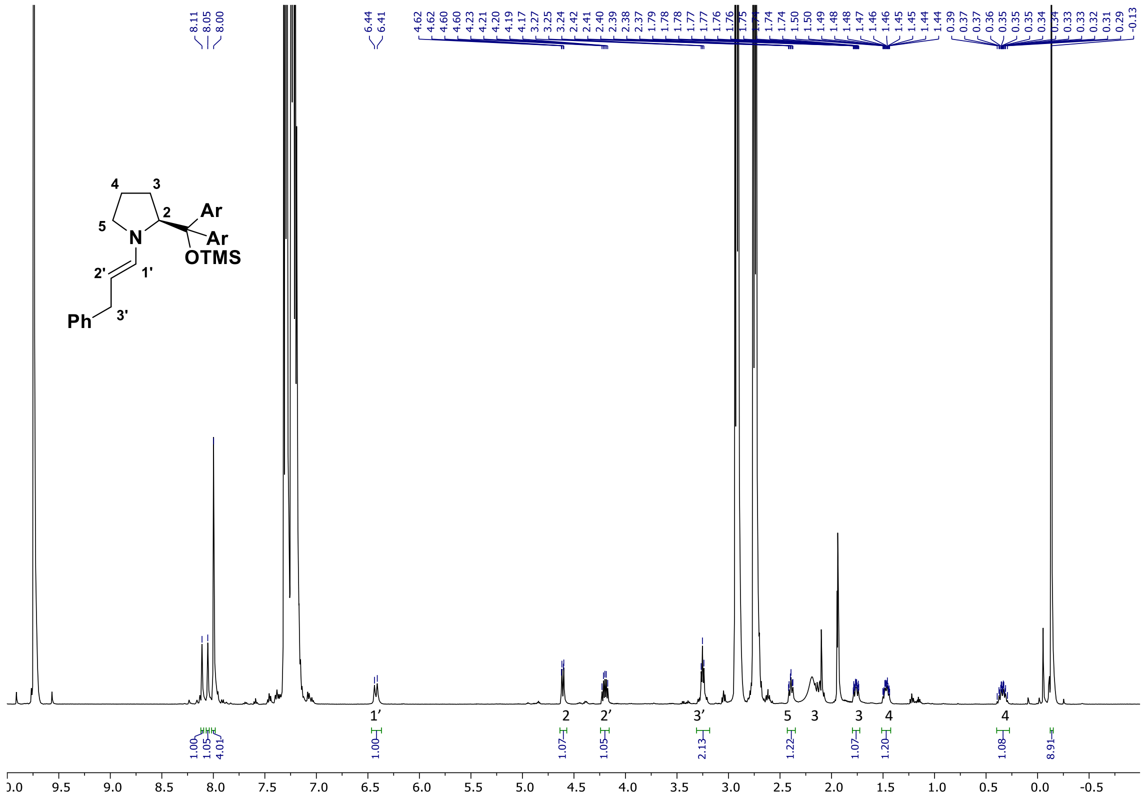

*Figure 45 – ^{1}H NMR (500 MHz, MeCN-d_3) spectrum of the enamine of hydrocinnamaldehyde and catalyst **3a**.*

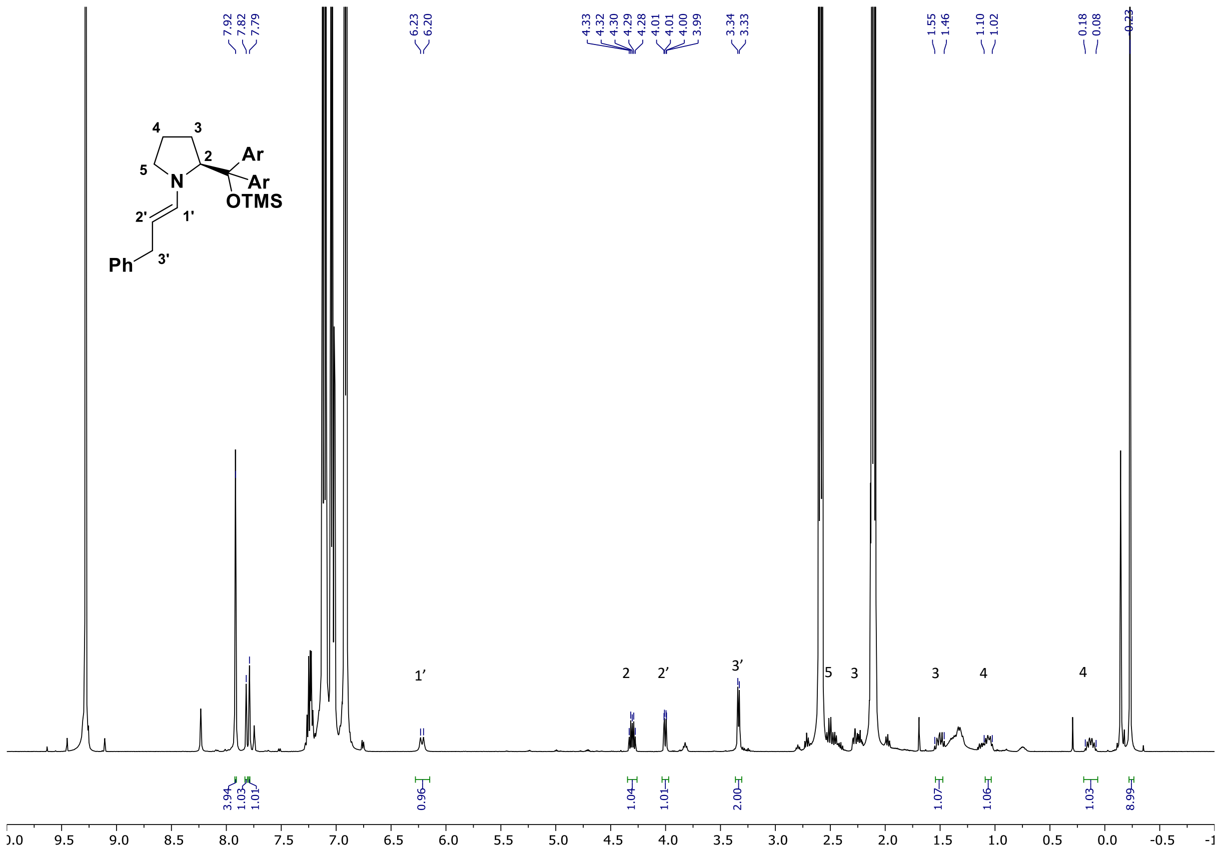

Figure 46 – ¹H NMR (500 MHz, toluene-d$_8$) spectrum of the enamine of hydrocinnamaldehyde and catalyst ***3a****.*

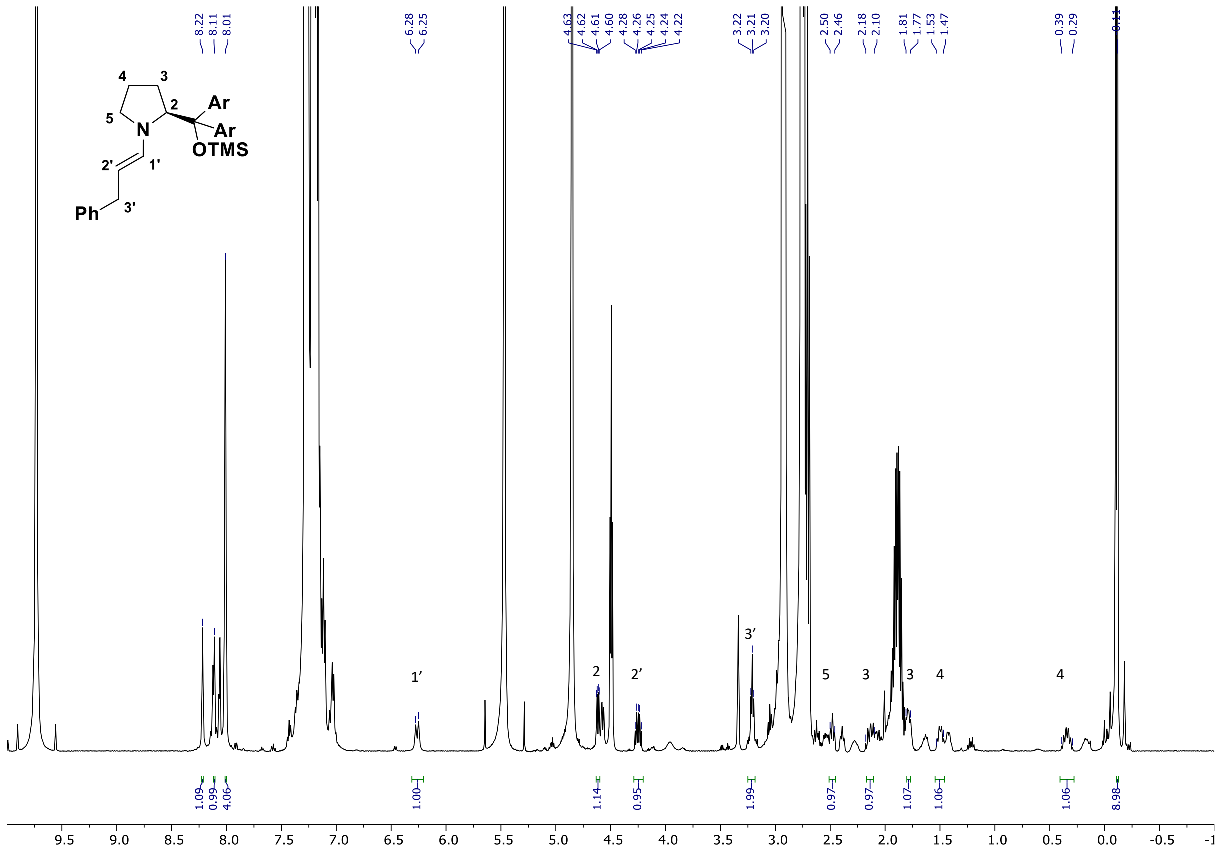

Figure 47 – ¹H NMR (500 MHz, methanol-d$_4$) spectrum of the enamine of hydrocinnamaldehyde and catalyst ***3a****. Some self-aldol peaks are observable.*

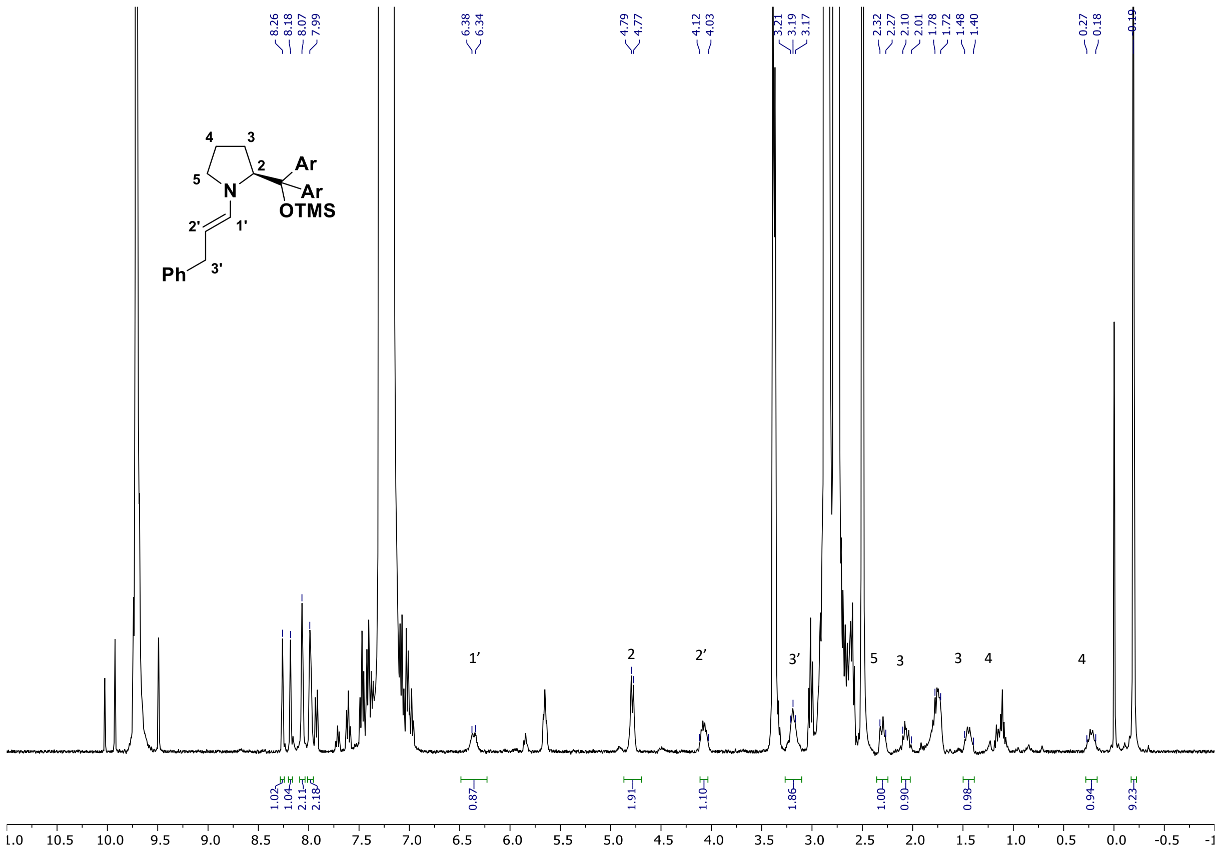

*Figure 48 – ^{1}H NMR (500 MHz, DMSO-d_6) spectrum of the enamine of hydrocinnamaldehyde and catalyst **3a**.*

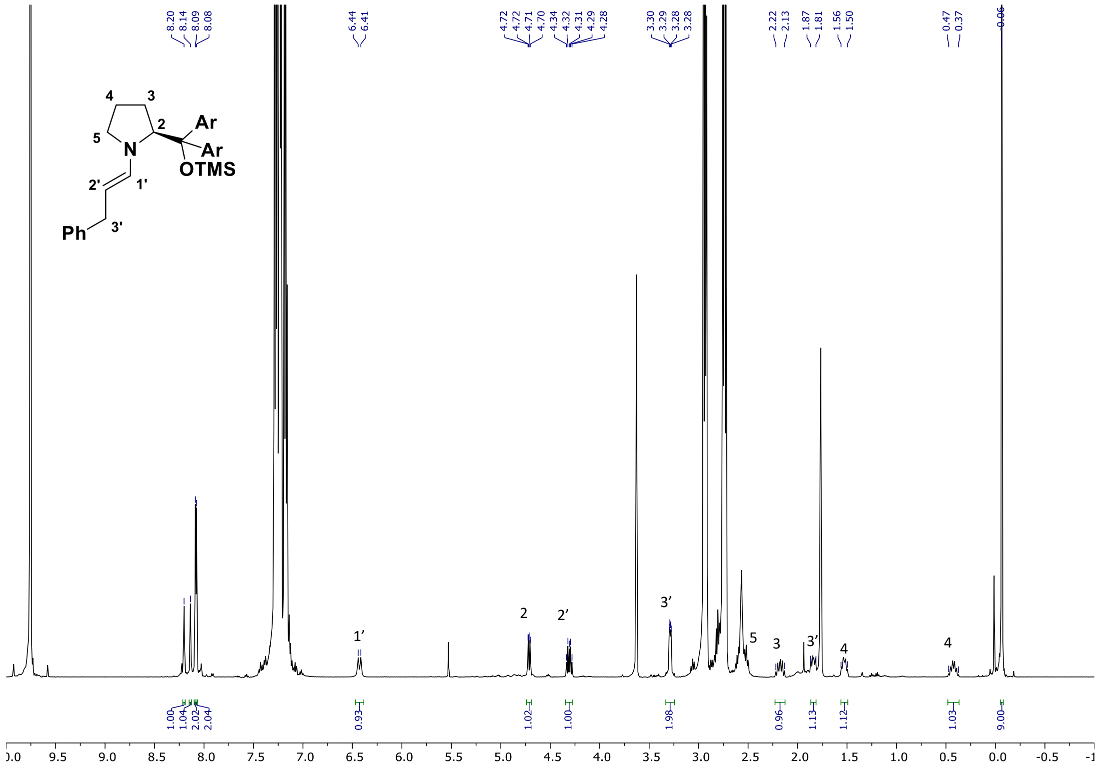

*Figure 49 – ^{1}H NMR (500 MHz, THF-d_8) spectrum of the enamine of hydrocinnamaldehyde and catalyst **3a**.*

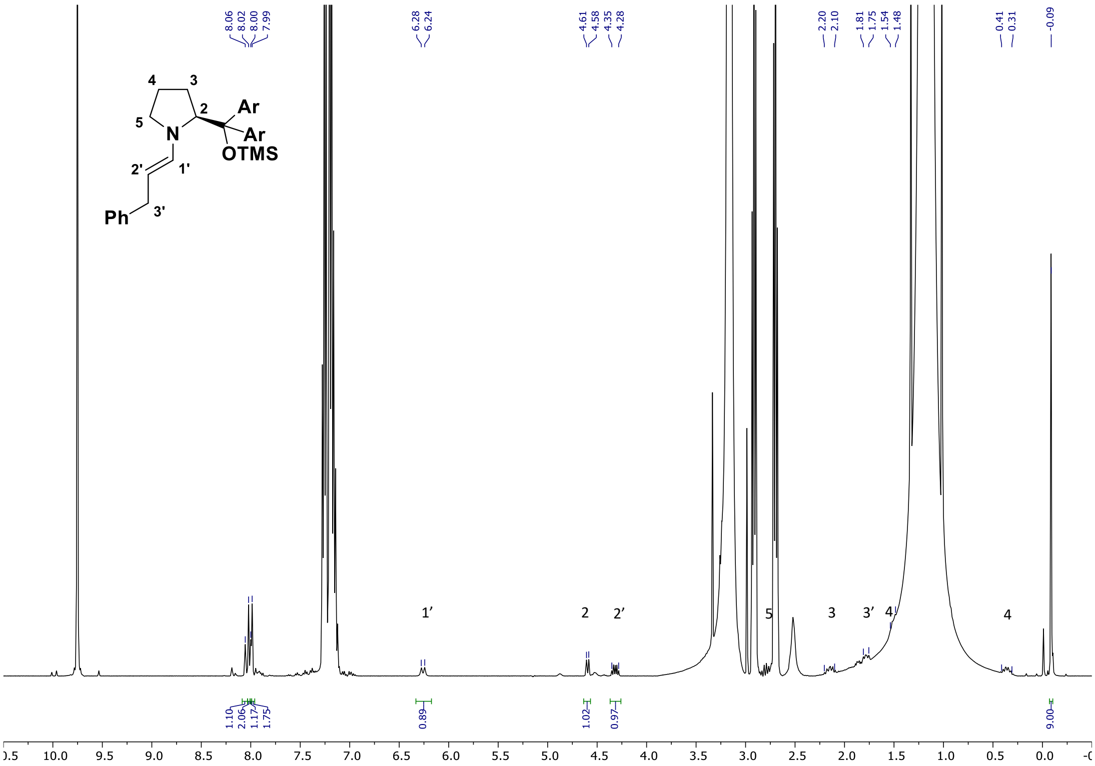

*Figure 50 – ^{1}H NMR (500 MHz, MTBE) spectrum of the enamine of hydrocinnamaldehyde and catalyst **3a**.*

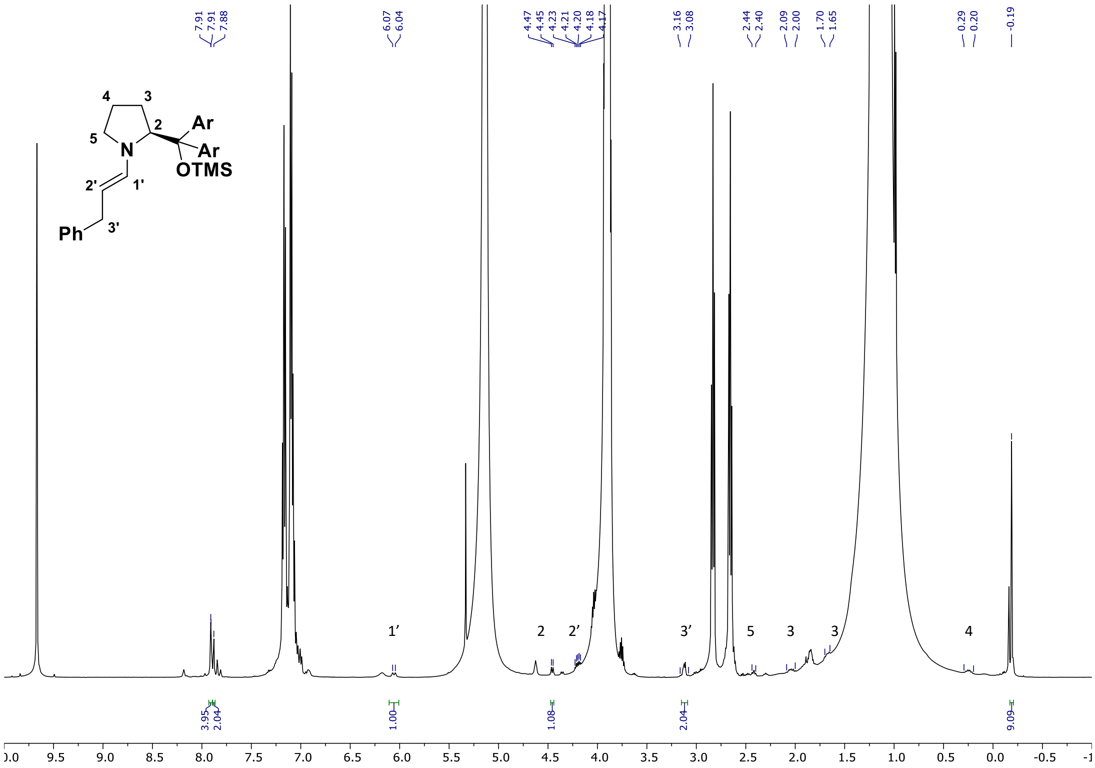

*Figure 51 – ^{1}H NMR (500 MHz, IPA) spectrum of the enamine of hydrocinnamaldehyde and catalyst **3a**.*

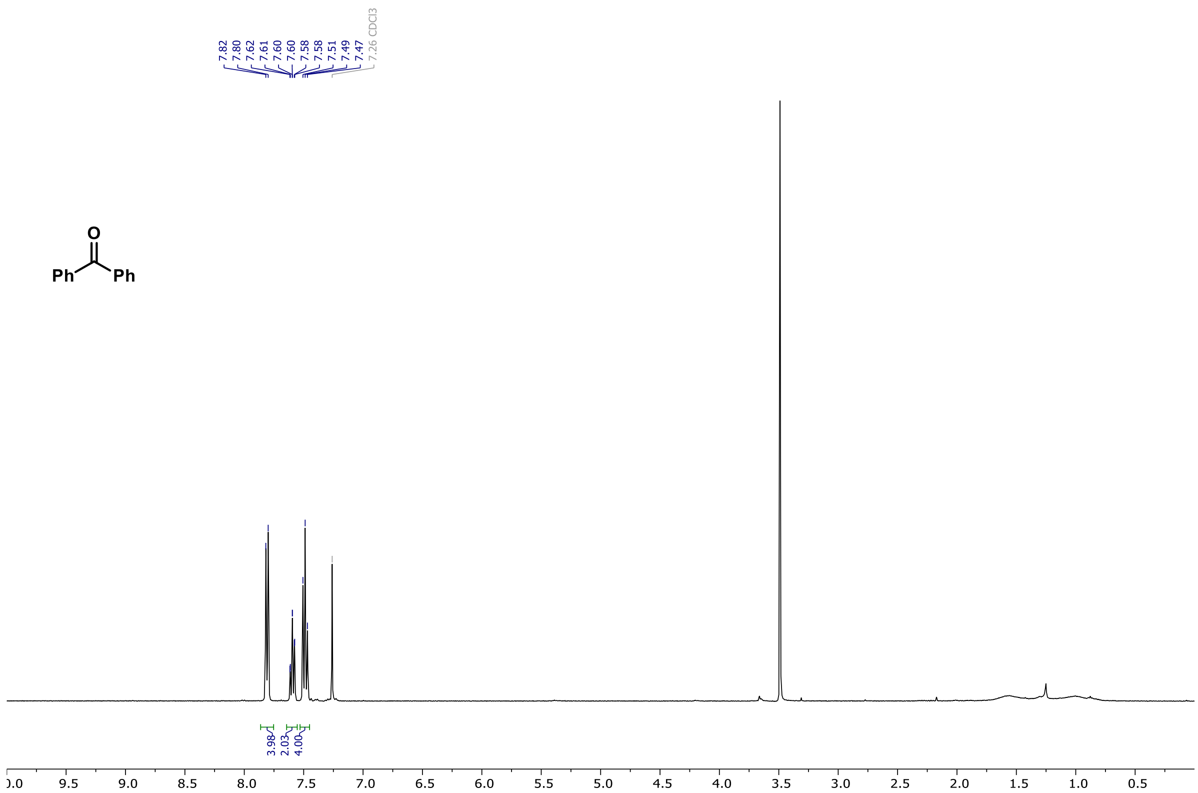

Figure 52 – ^{1}H NMR (500 MHz, $CDCl_3$) spectrum of benzophenone, recovered from catalyst deactivation experiments.

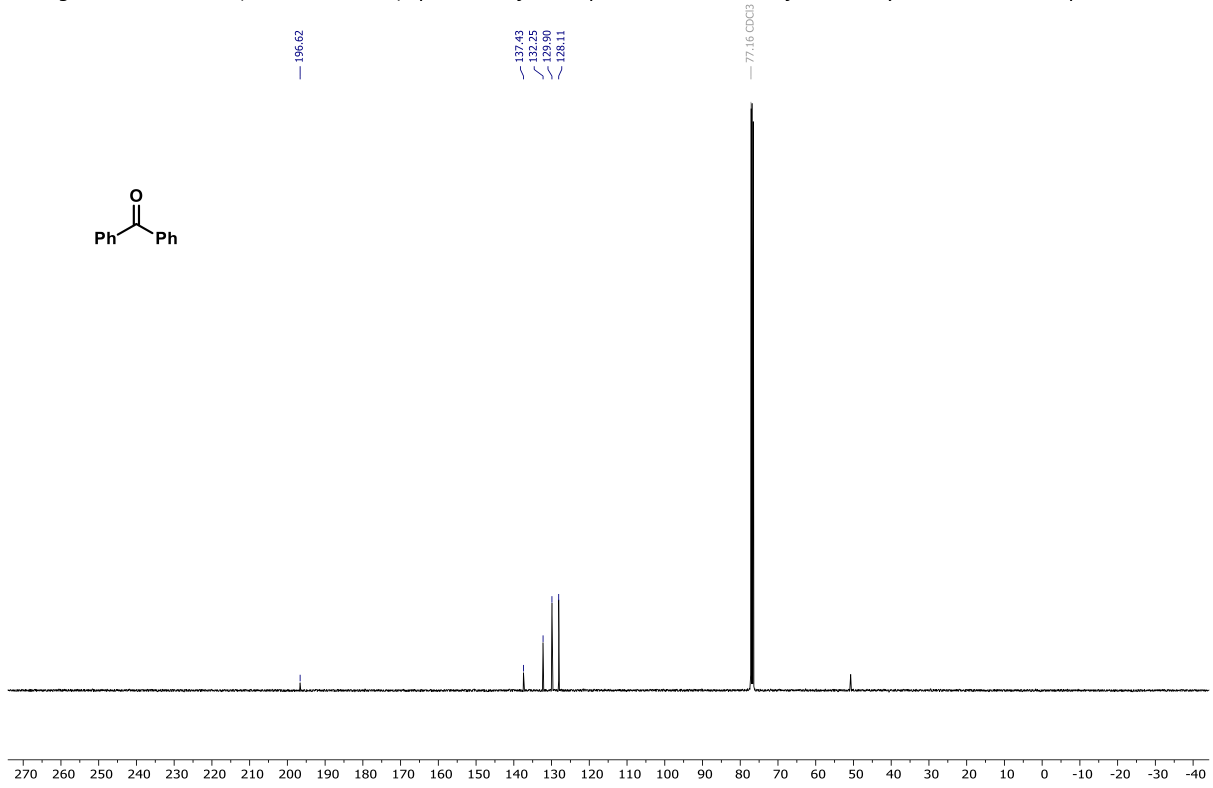

Figure 53 – ^{13}C NMR (125 MHz, $CDCl_3$) spectrum of benzophenone, recovered from catalyst deactivation experiments.

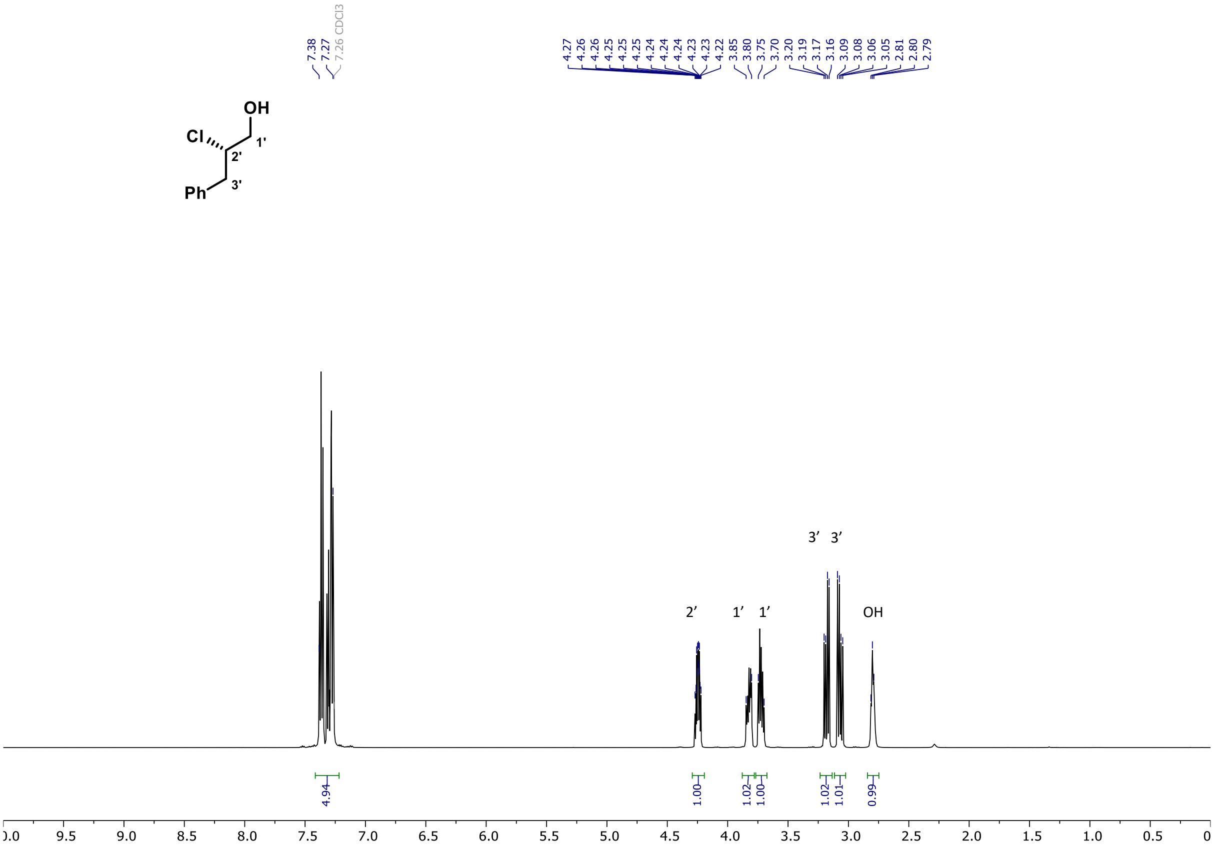

Figure 54 – ^{1}H NMR (500 MHz, $CDCl_3$) spectrum of 2-chloro-3-phenylpropan-1-ol.

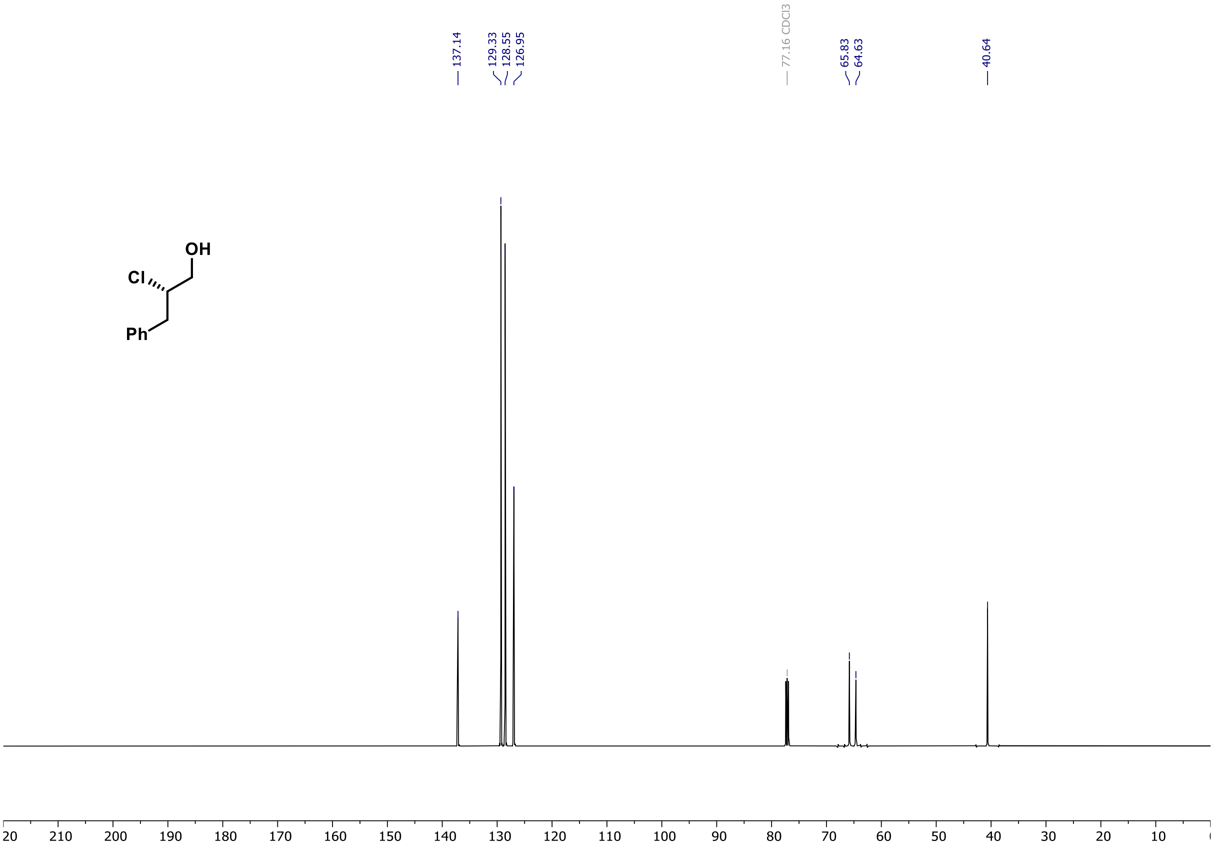

Figure 55 – ^{13}C NMR (125 MHz, $CDCl_3$) spectrum of 2-chloro-3-phenylpropan-1-ol.

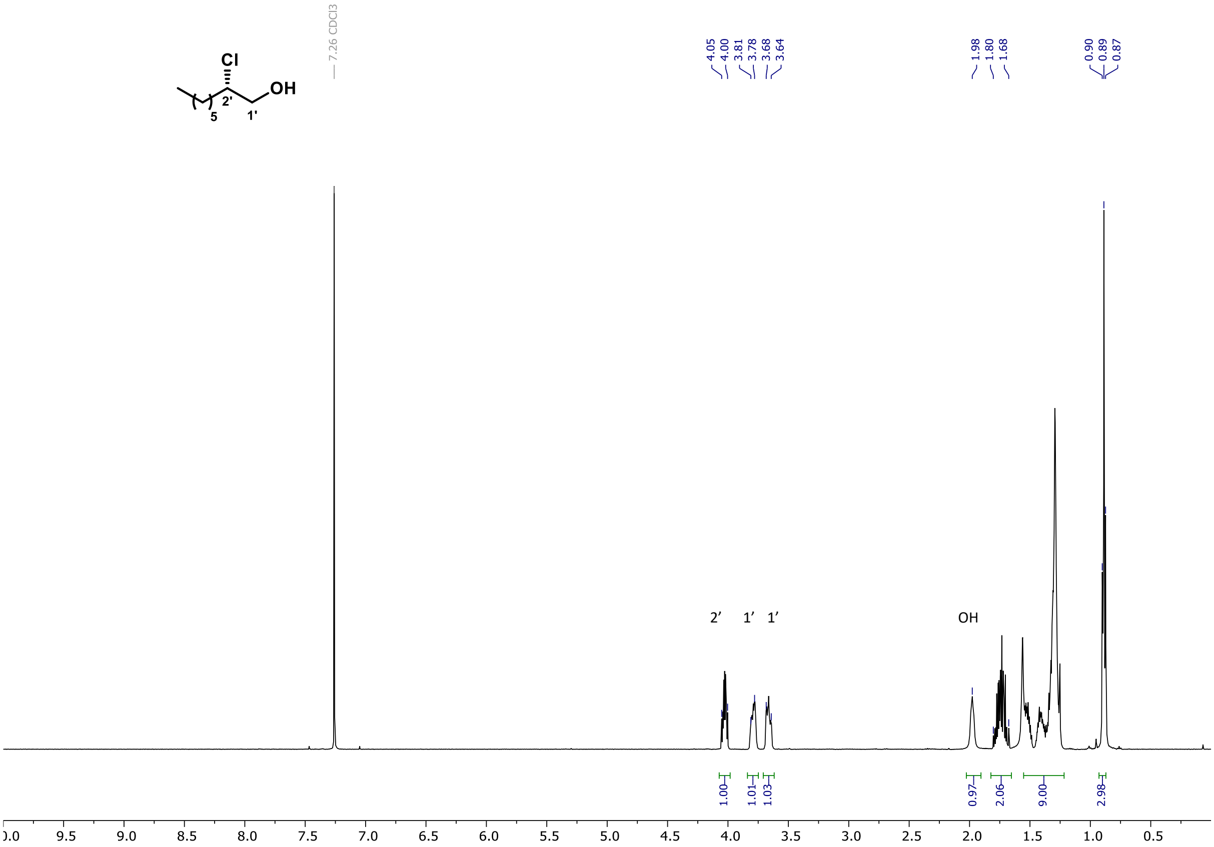

Figure 56 – ^{1}H NMR (500 MHz, $CDCl_3$) spectrum of 2-chloro-octan-1-ol.

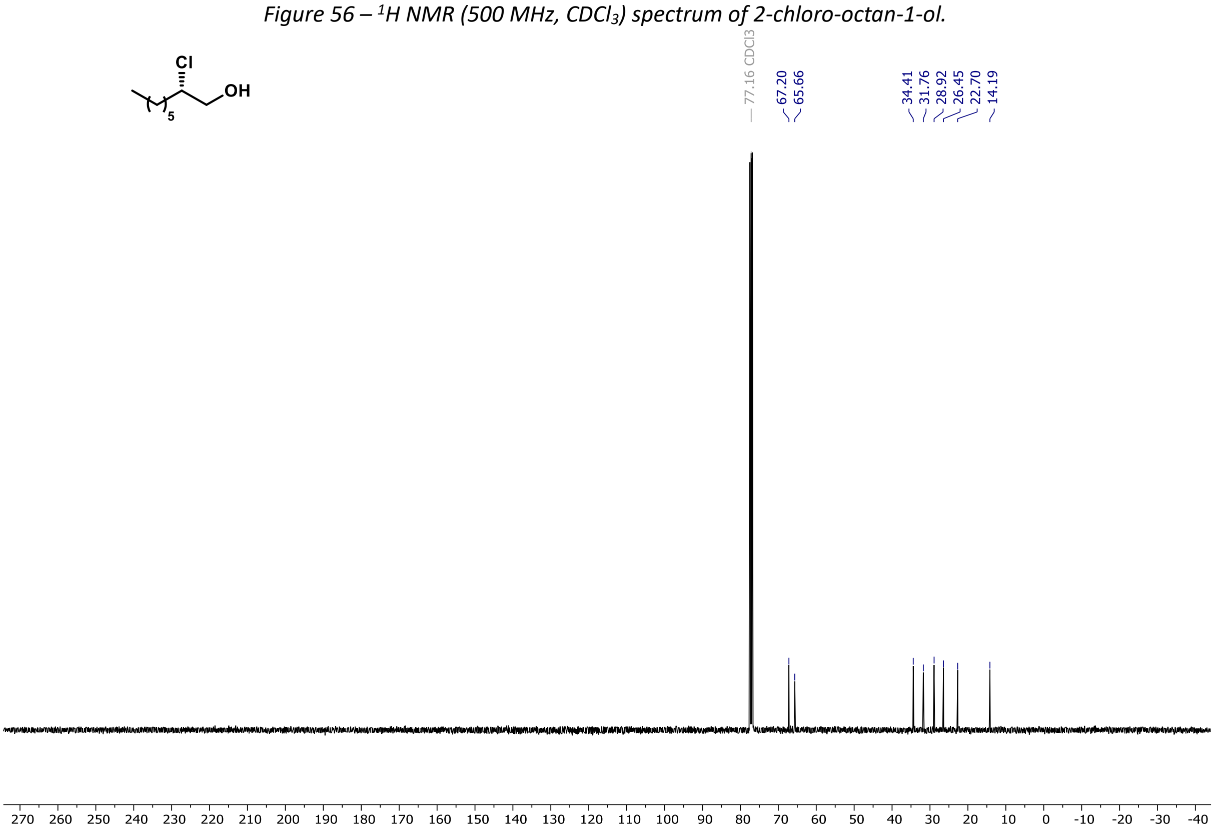

Figure 57 – ^{13}C NMR (125 MHz, $CDCl_3$) spectrum of 2-chloro-octan-1-ol.

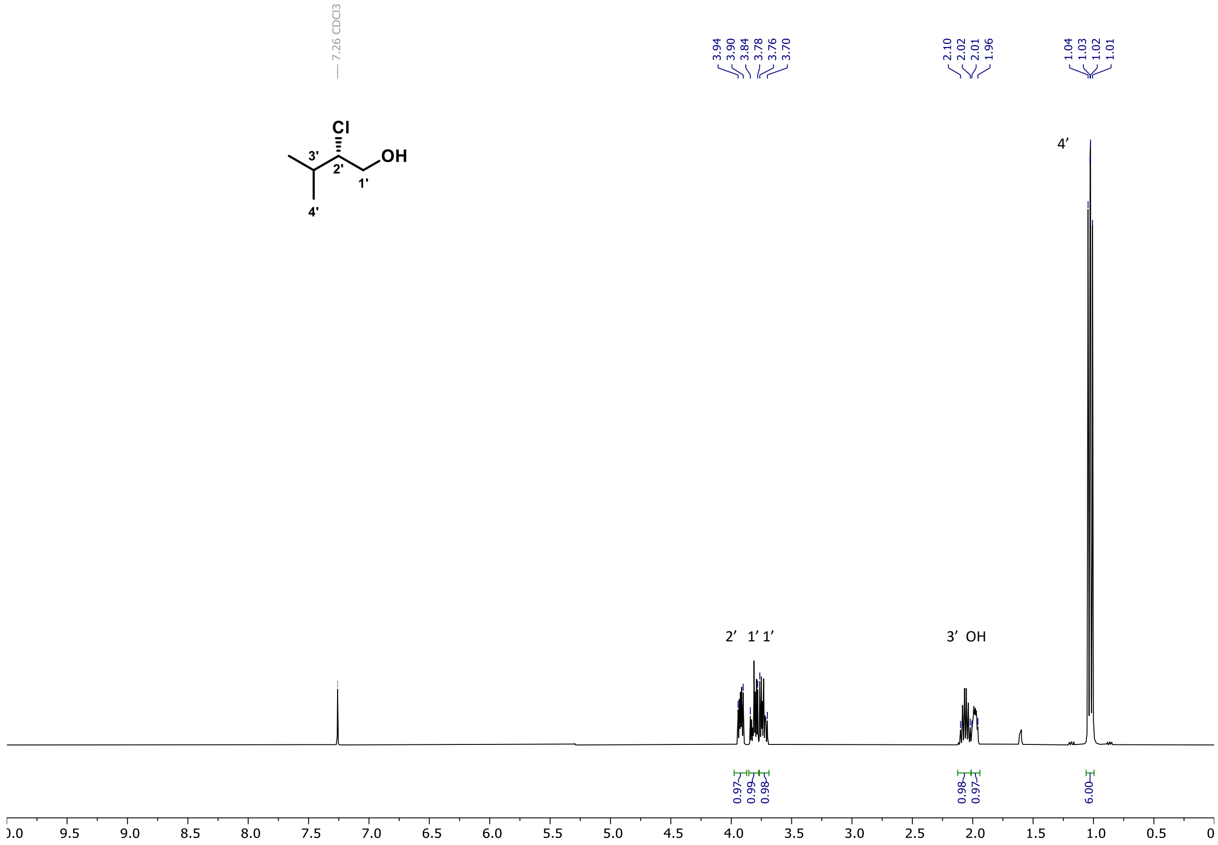

Figure 58 – ^{1}H NMR (500 MHz, $CDCl_3$) spectrum of 2-chloro-3-methylbutan-1-ol.

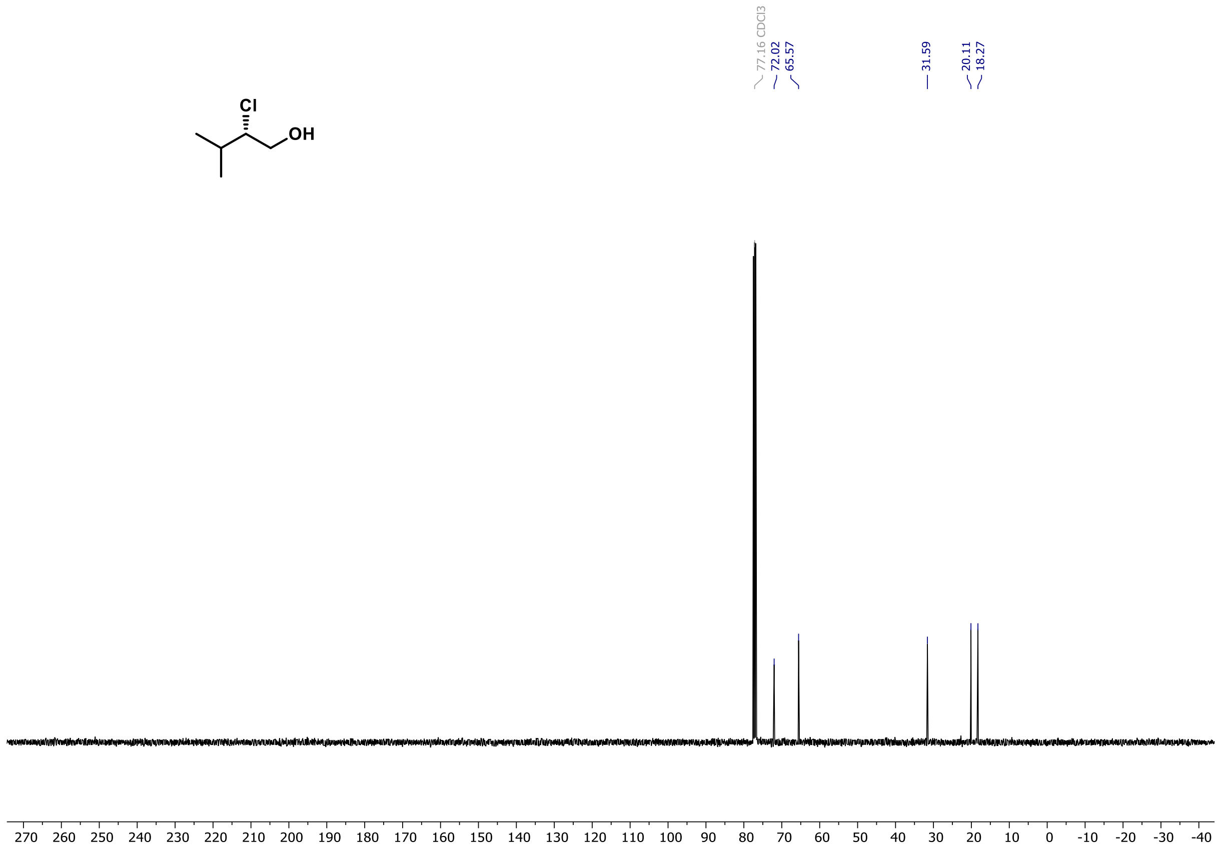

Figure 59 – ^{13}C NMR (125 MHz, $CDCl_3$) spectrum of 2-chloro-3-methylbutan-1-ol.

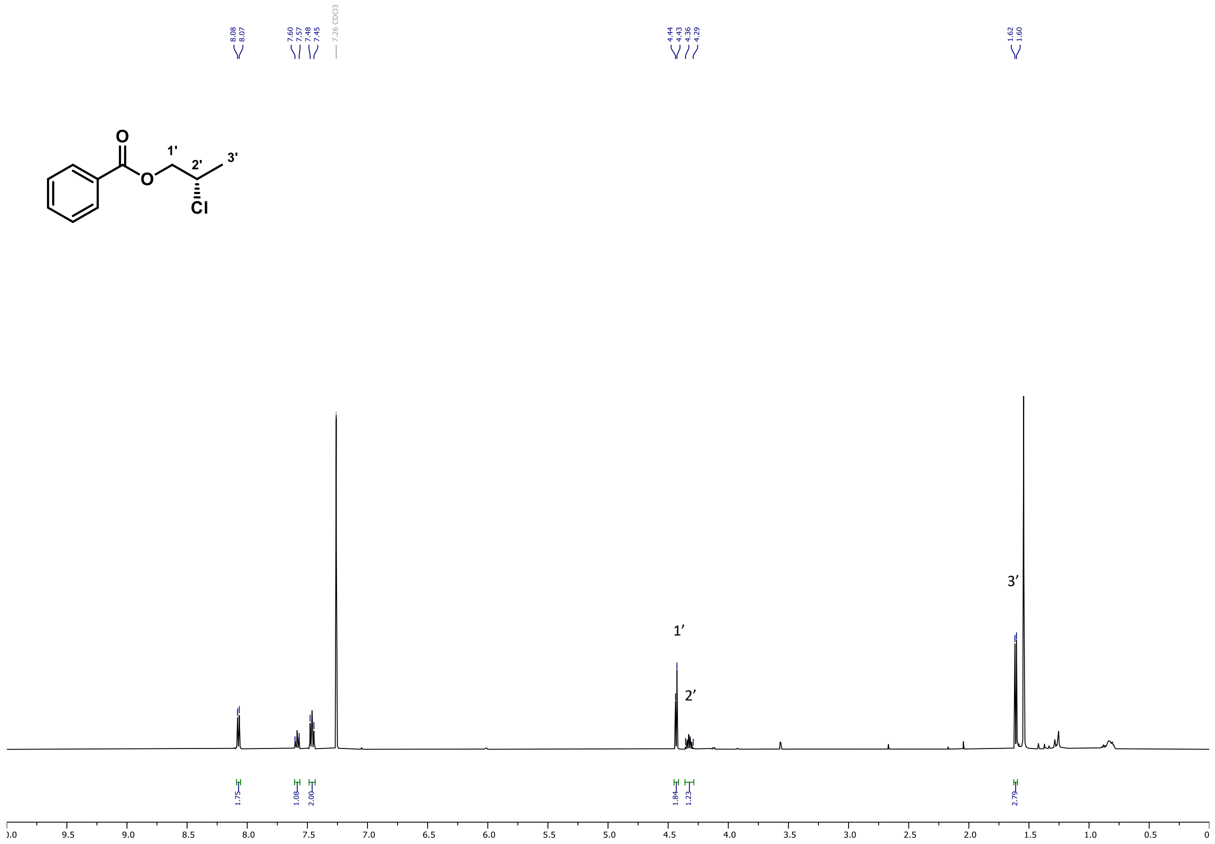

Figure 60 – ^{1}H NMR (500 MHz, $CDCl_3$) of 2-chloropropyl benzoate.

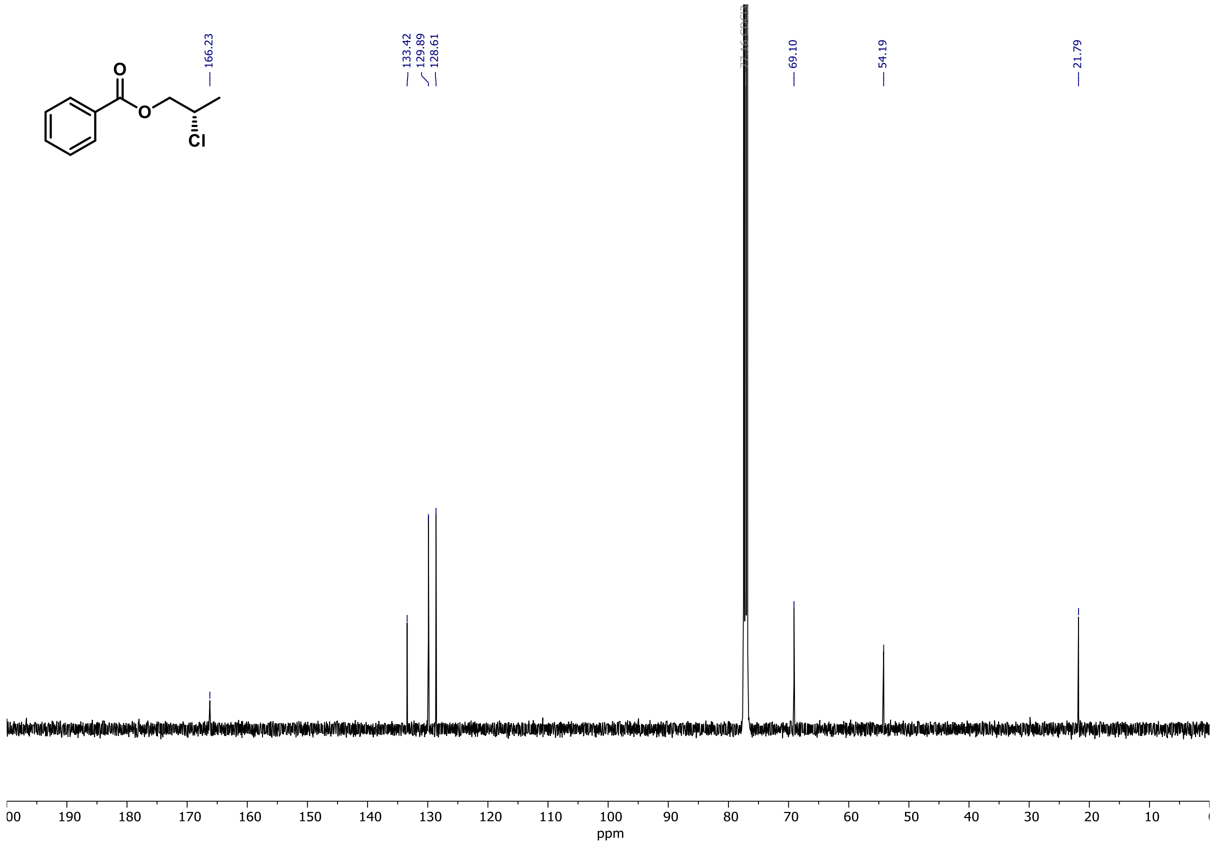

Figure 61 – ^{13}C NMR (125 MHz, $CDCl_3$) of 2-chloropropyl benzoate.

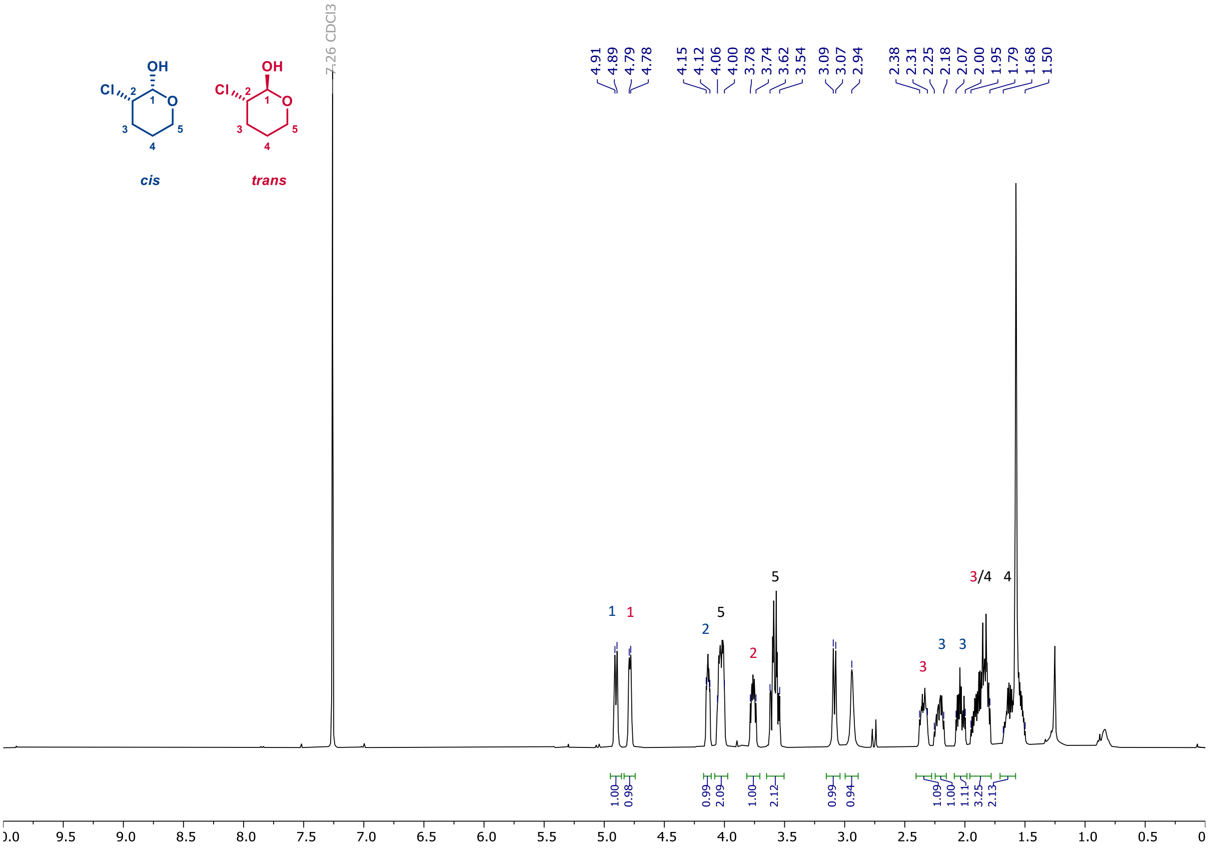

Figure 62 – ^{1}H NMR (500 MHz, $CDCl_3$) spectrum of 3-chlorotetrahydro-2H-pyran-2-ol.

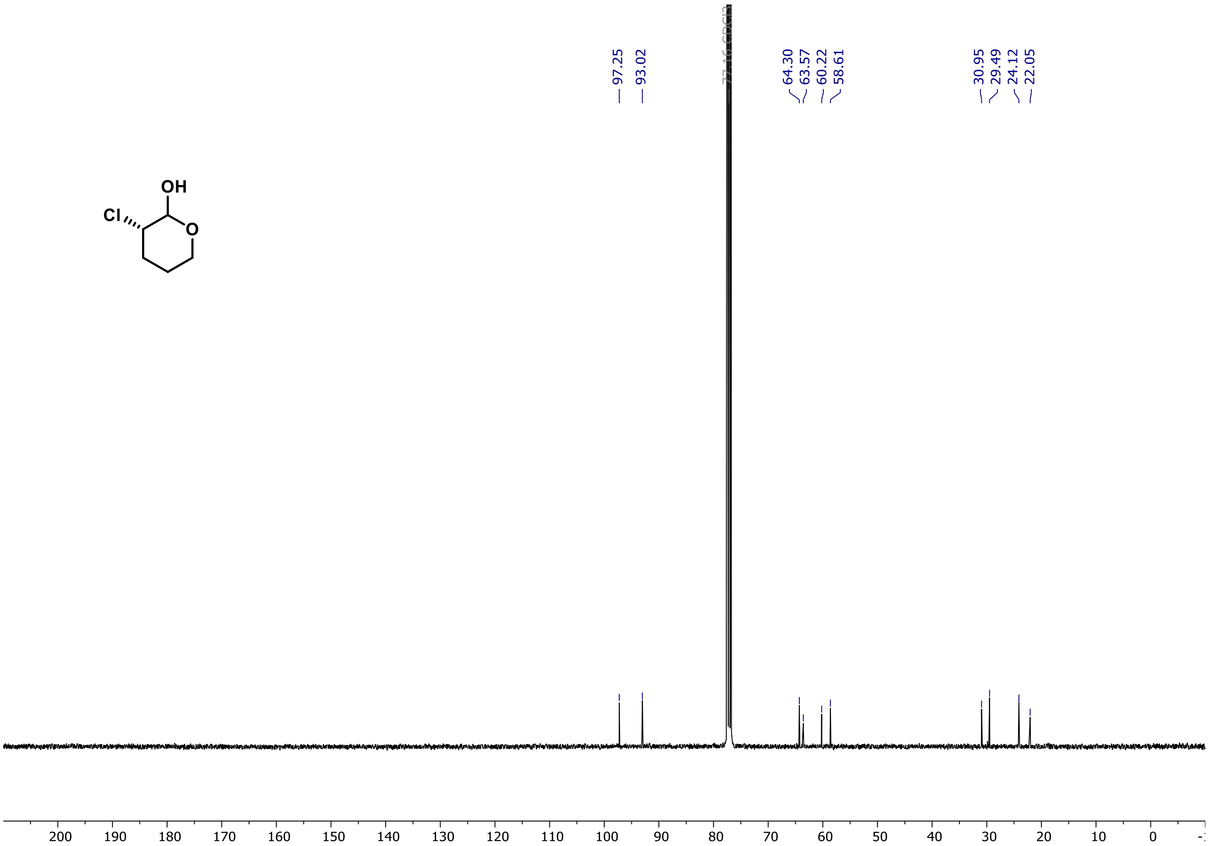

Figure 63 – ^{13}C NMR (125 MHz, $CDCl_3$) spectrum of 3-chlorotetrahydro-2H-pyran-2-ol.

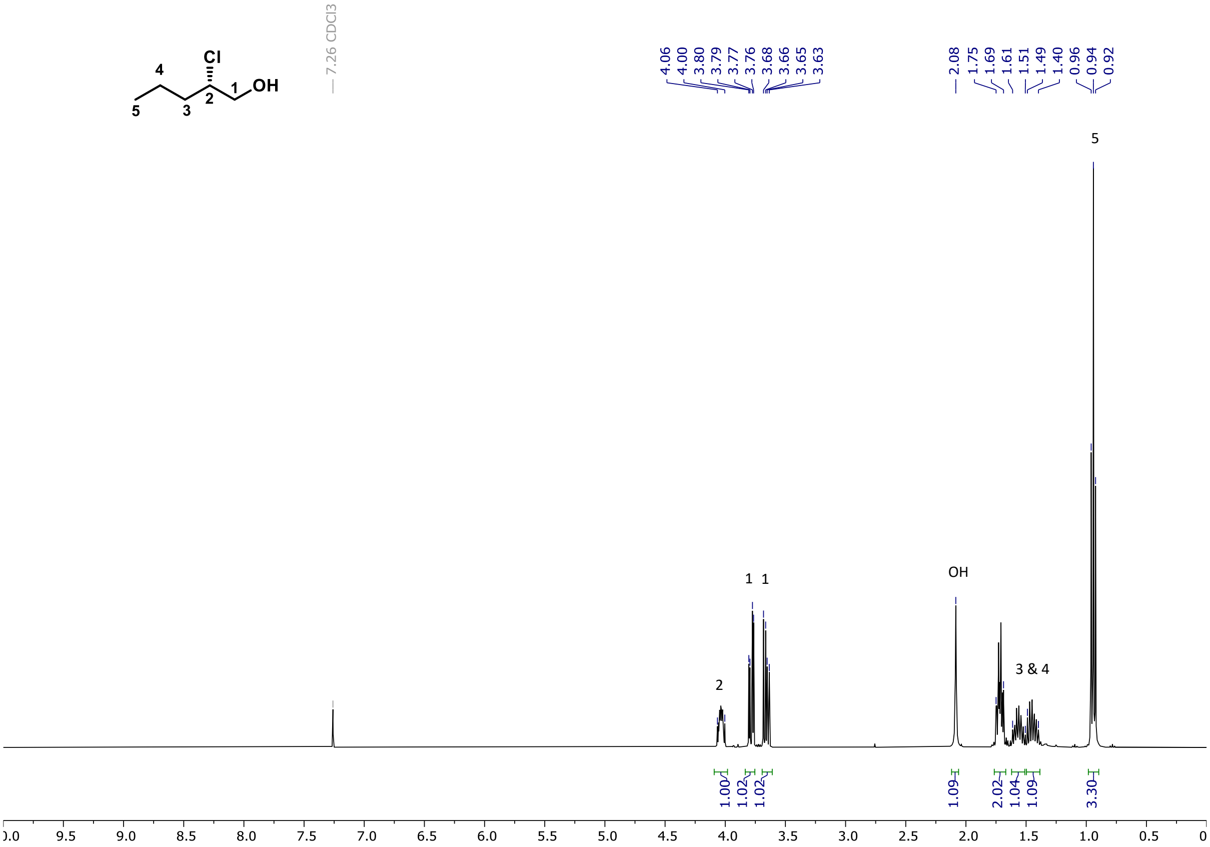

Figure 64 – ^{1}H NMR (400 MHz, $CDCl_3$) of 2-chloropentanol.

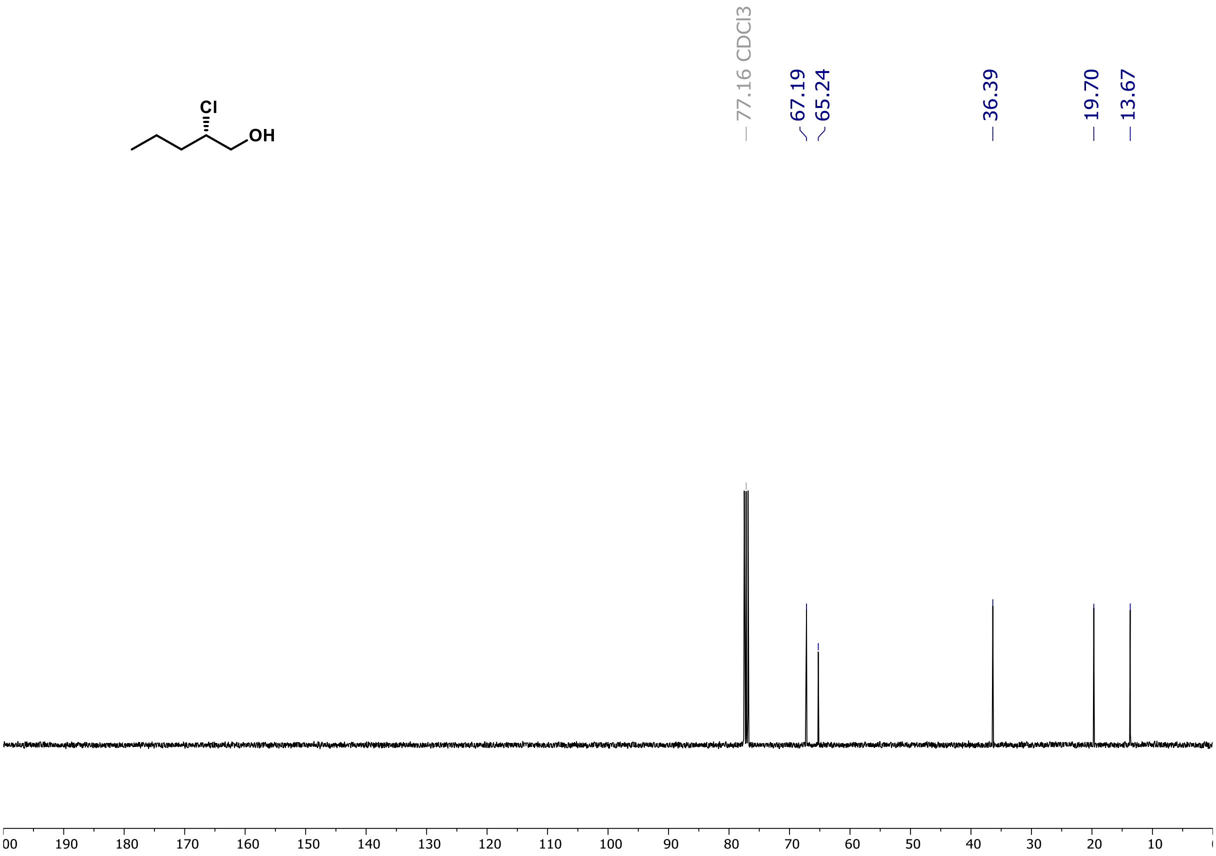

Figure 65 – ^{13}C NMR (101 MHz, $CDCl_3$) of 2-chloropentanol.

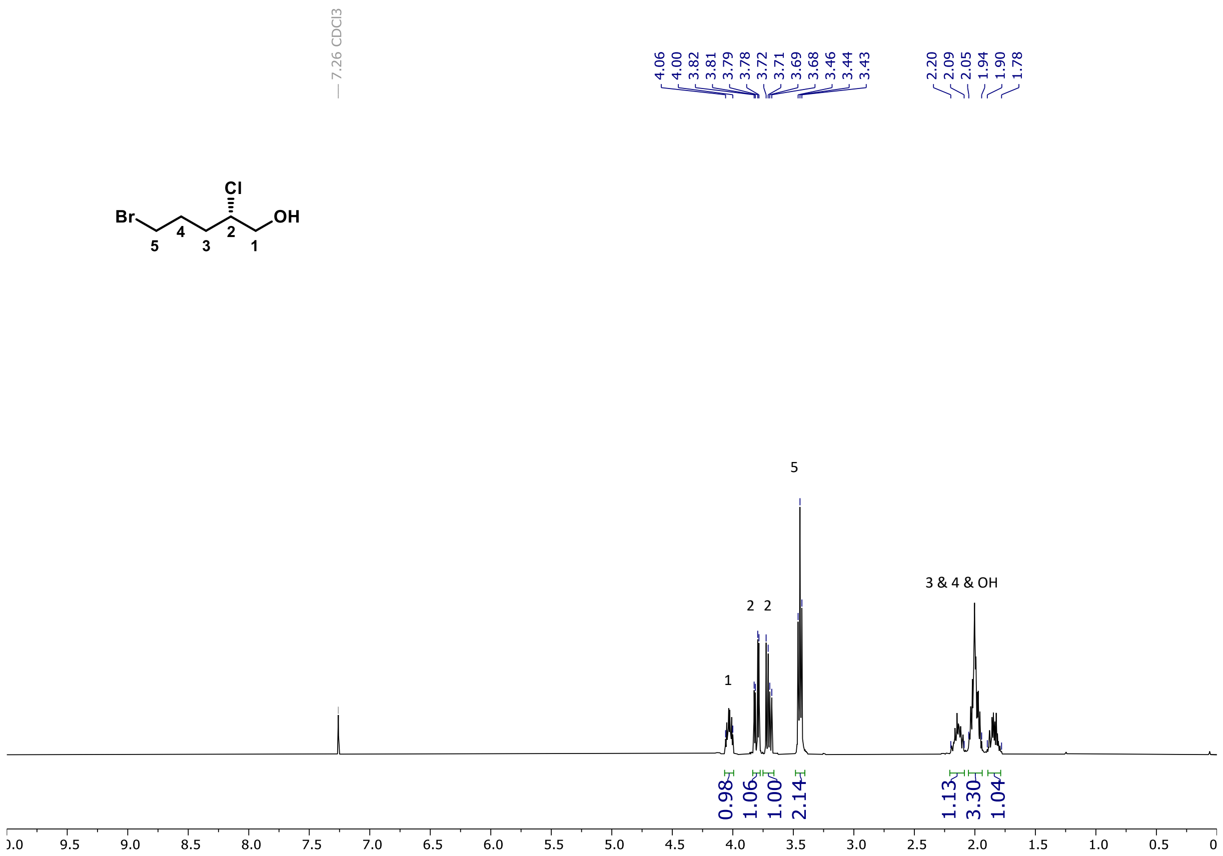

Figure 66 – ^{1}H NMR (400 MHz, $CDCl_3$) of 2-chloro-5-bromopentanol.

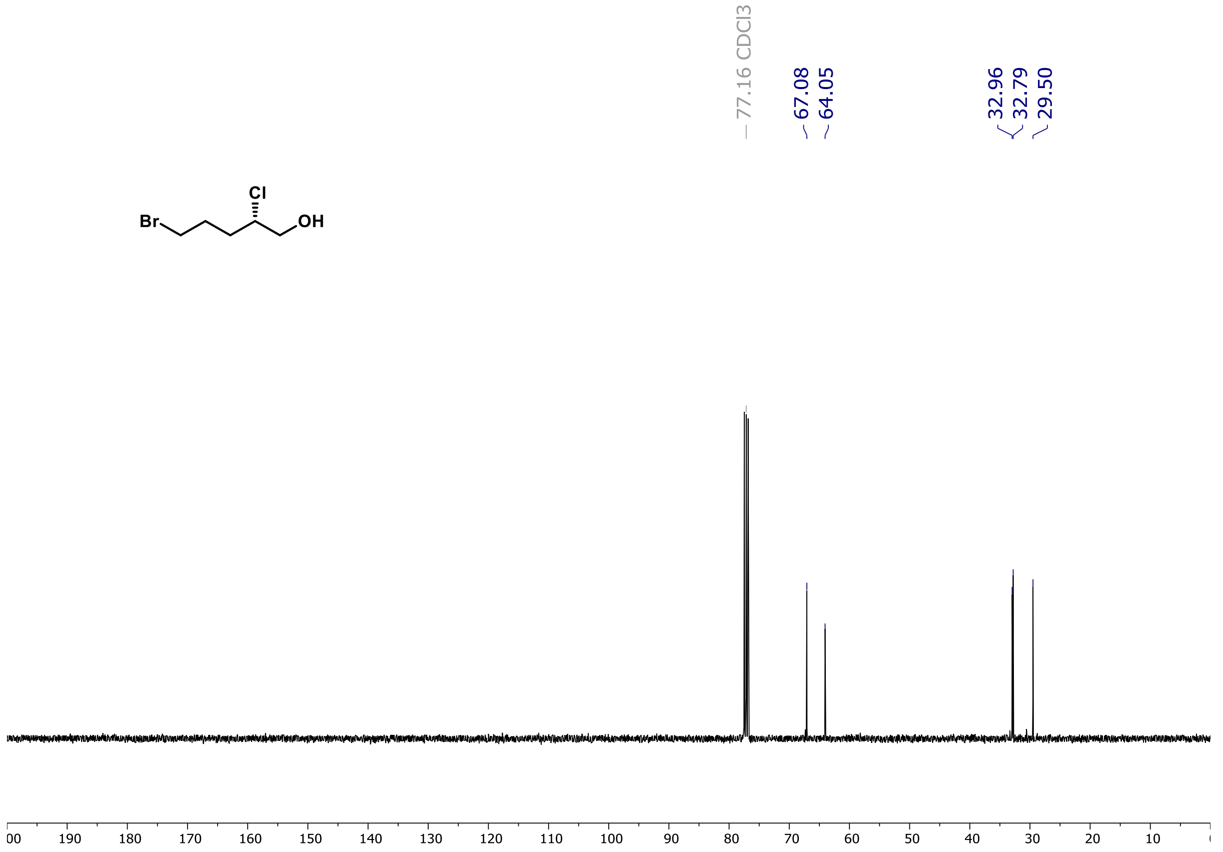

Figure 67 – ^{13}C NMR (101 MHz, $CDCl_3$) of 2-chloro-5-bromopentanol.

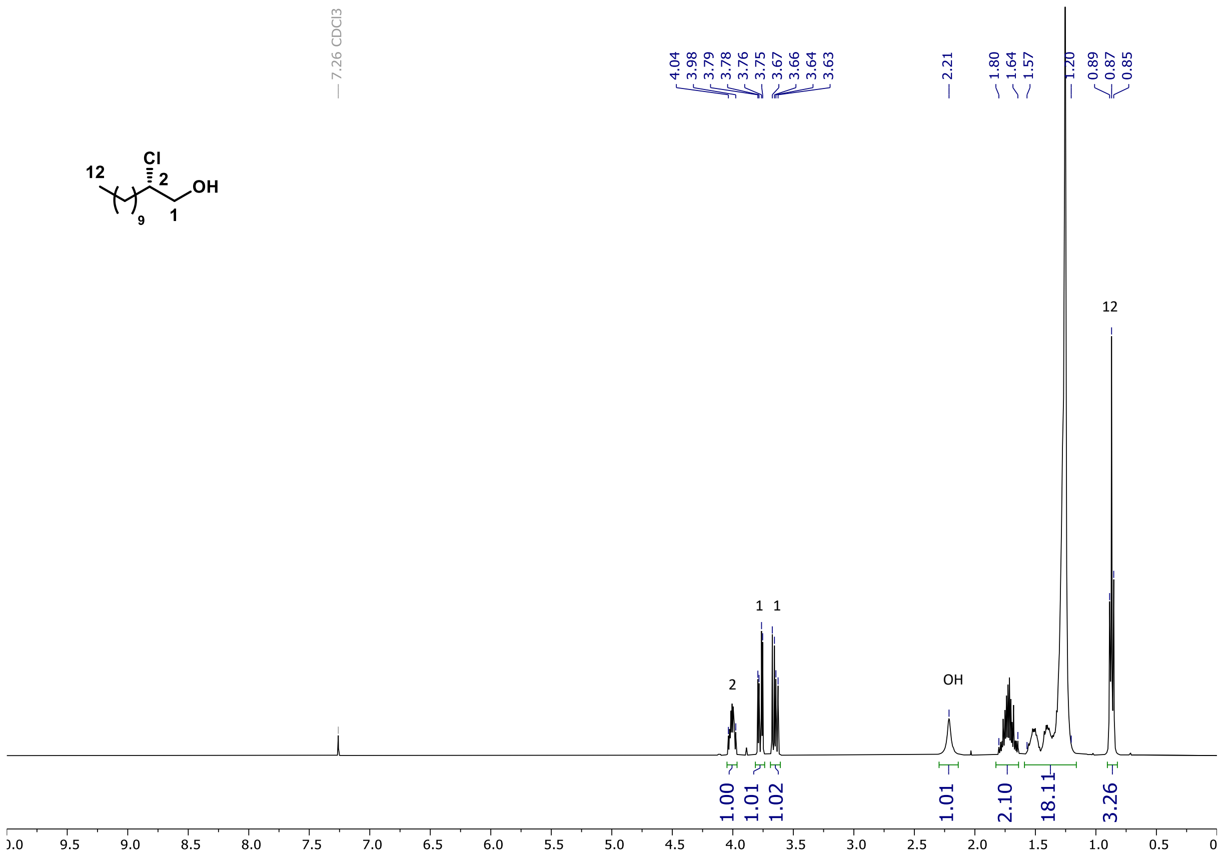

Figure 68 – ^{1}H NMR (400 MHz, $CDCl_3$) of 2-chlorododecanol.

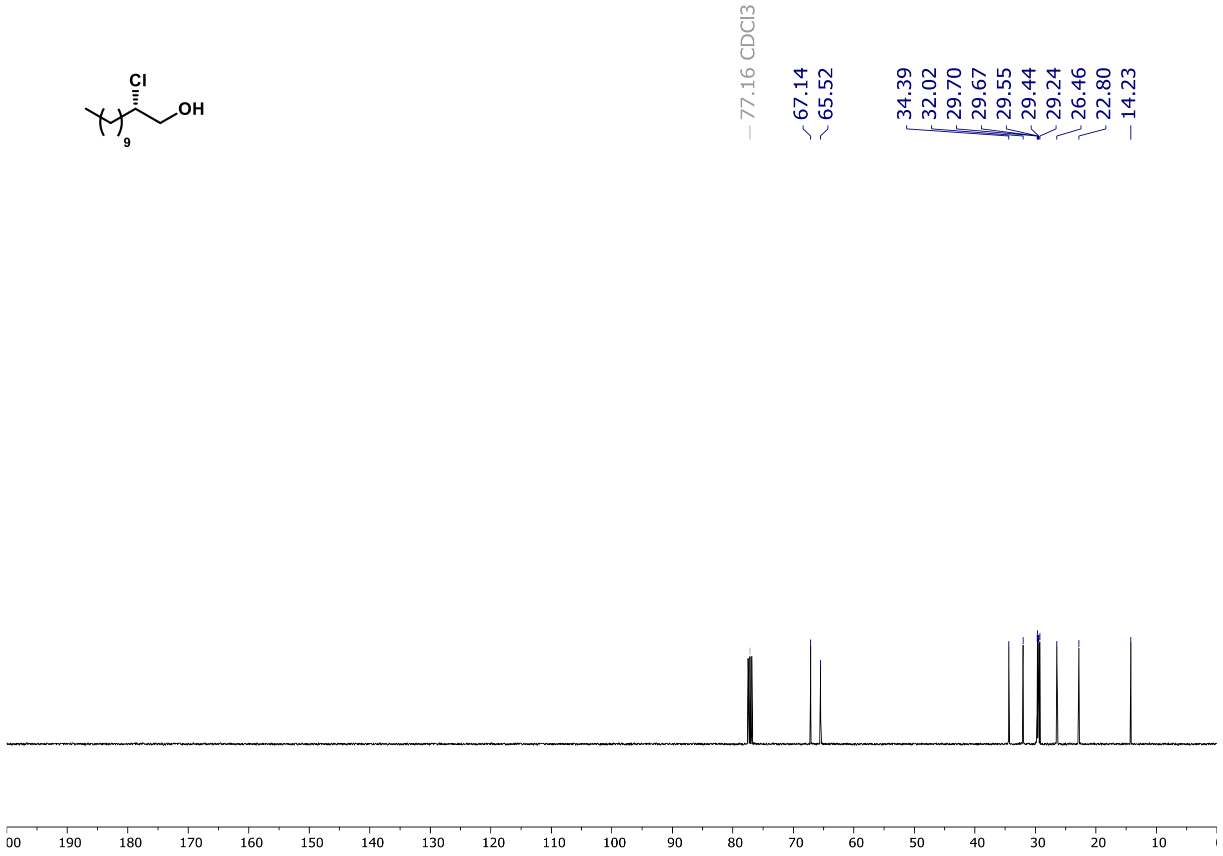

Figure 69 – ^{13}C NMR (101 MHz, $CDCl_3$) of 2-chlorododecanol.

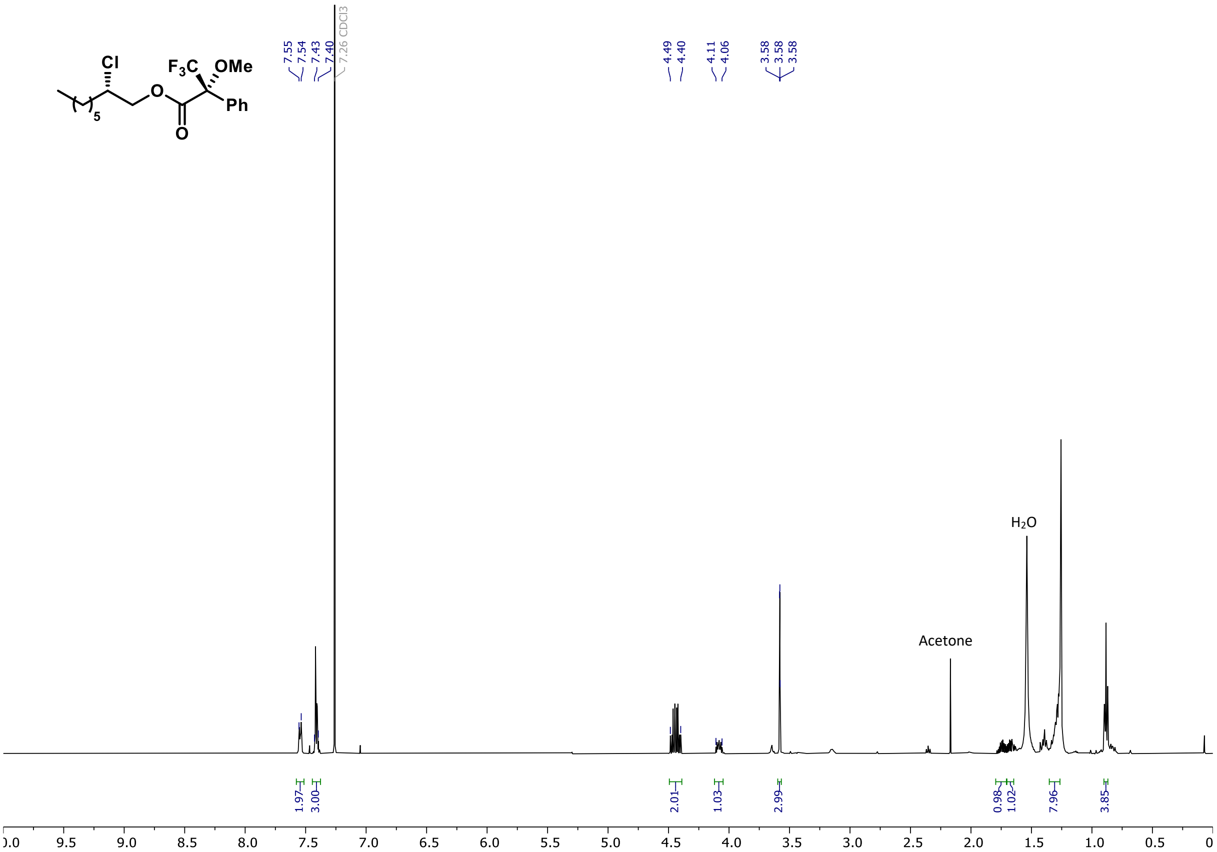

Figure 70 – ¹H NMR (500 MHz, $CDCl_3$) spectrum of the Mosher's acid derivatized 2-chloro-octanol.

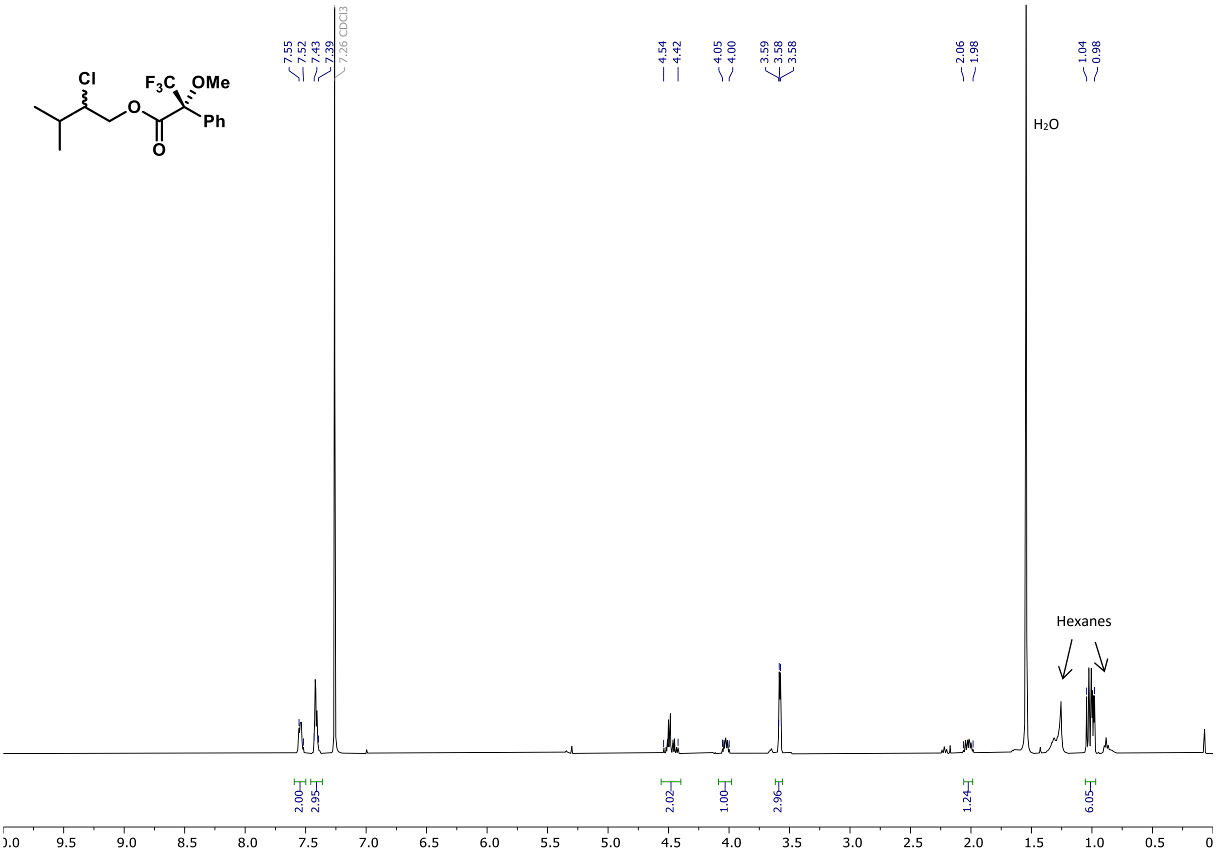

Figure 71 – ¹H NMR (500 MHz, $CDCl_3$) spectrum of the Mosher's acid derivatized 2-chloro-3-methylbutan-1-ol.

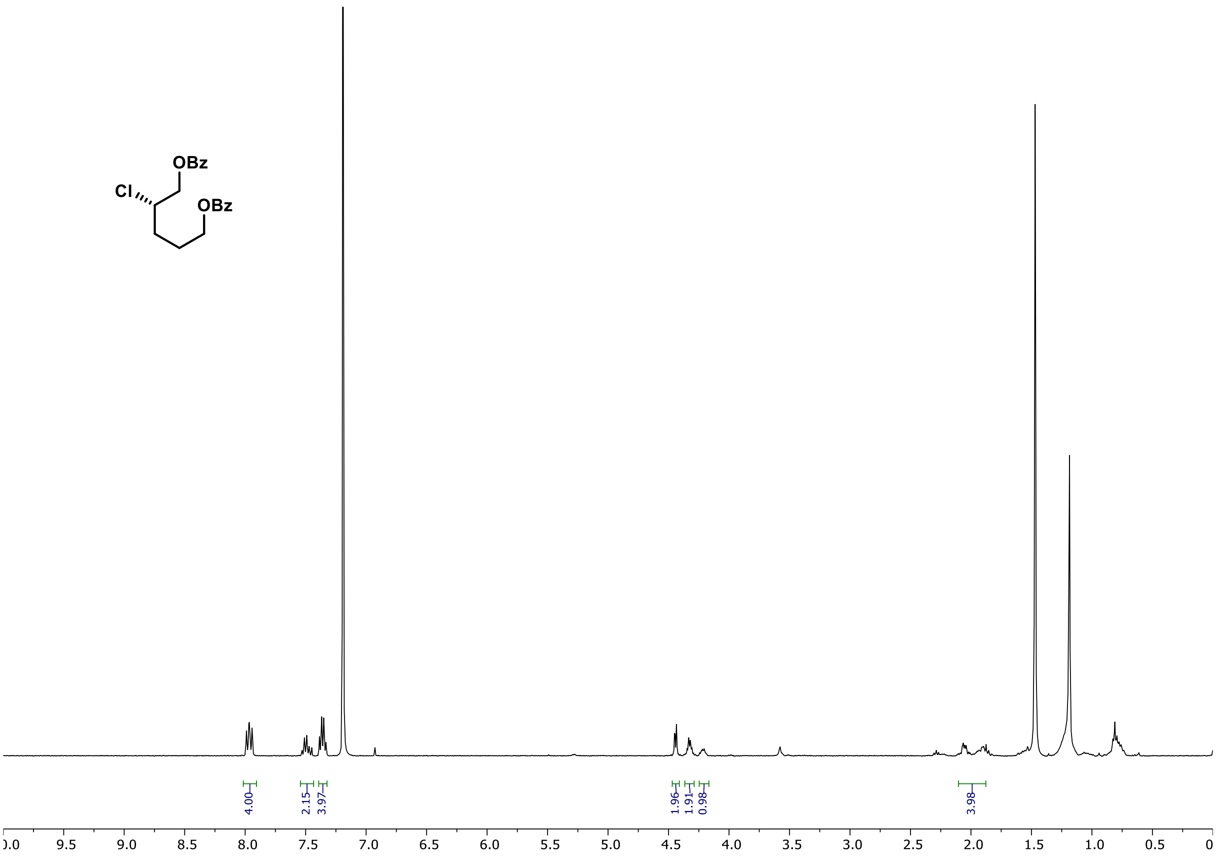

Figure 72 – Dibenzoylated derivative of (S)-2-chloropentane-1,5-diol.

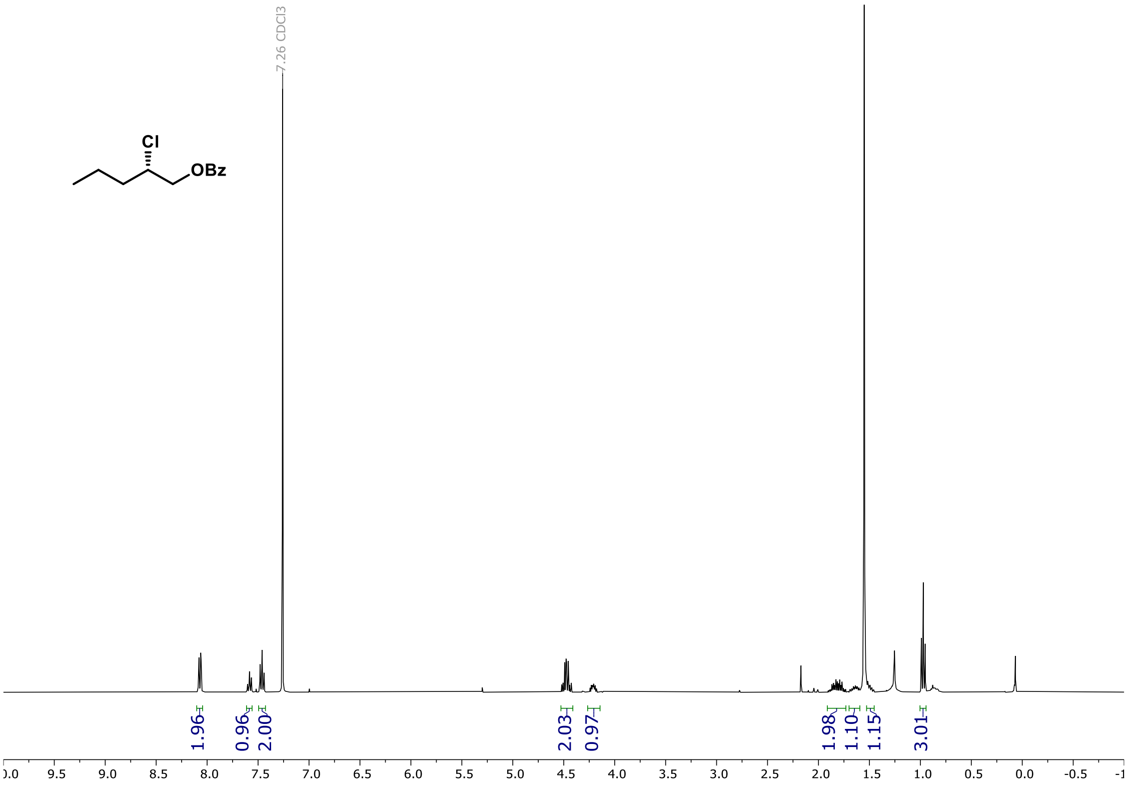

Figure 73 – Benzoylated derivative of 2-chloropentanol.

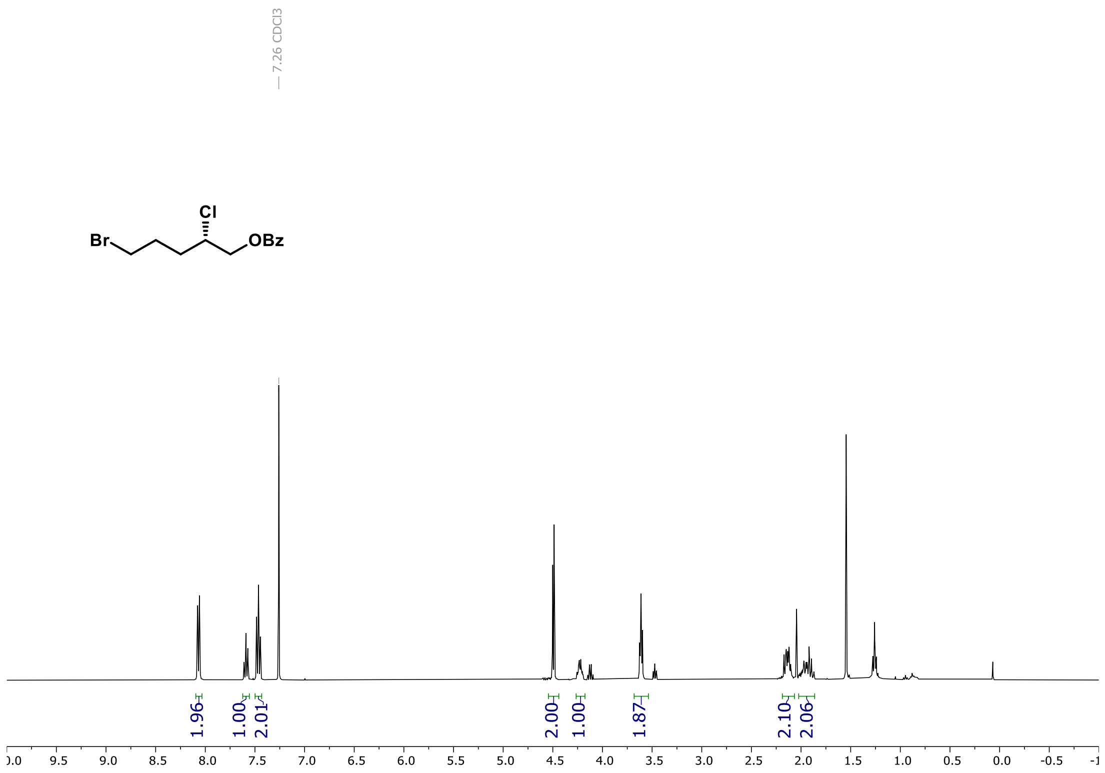

Figure 74 – Benzoylated derivative of 2-chloro-5-bromopentanol.

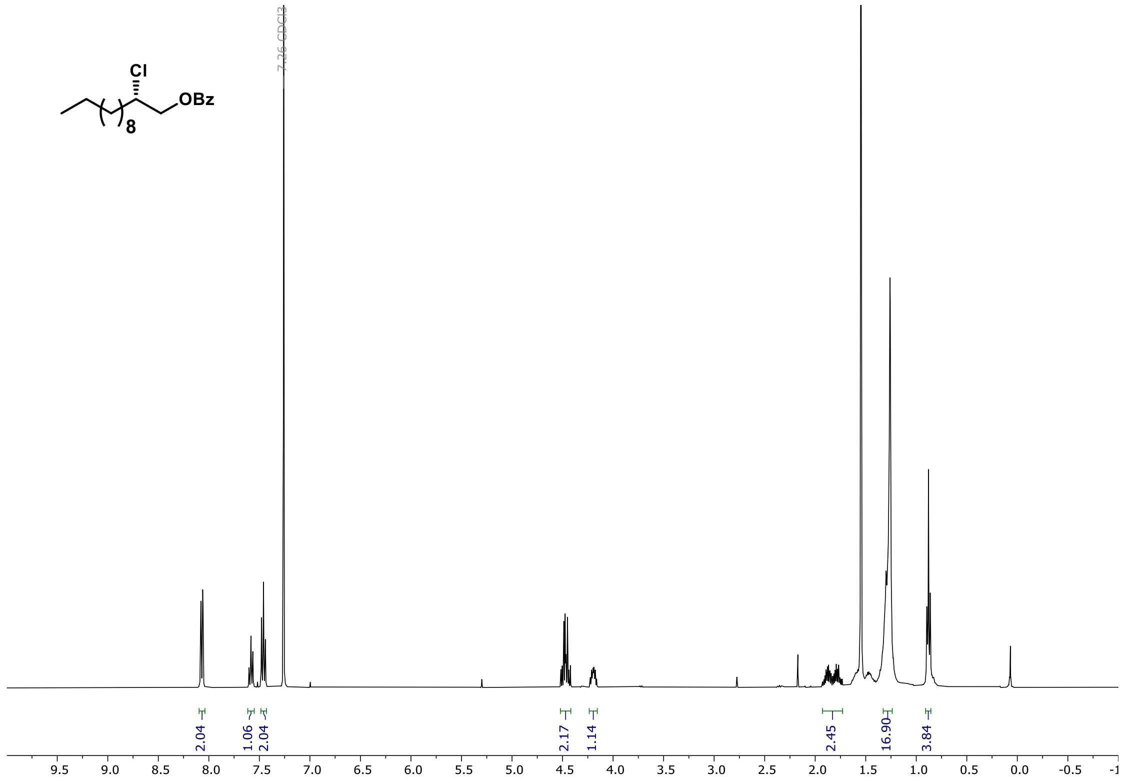

Figure 75 – Benzoylated derivative of 2-chlorododecanol.

14. Determination of the enantiomeric ratio of α-chloroalcohol products

Slow addition with complete consumption of NCS (Section 8.3.)

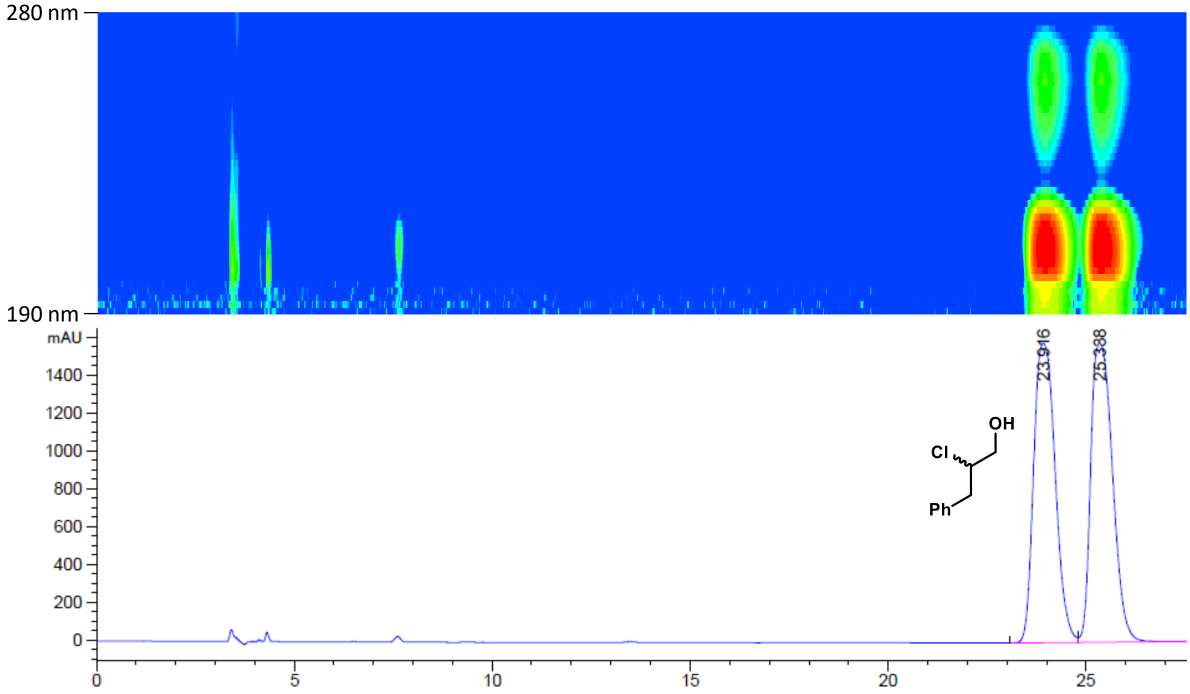

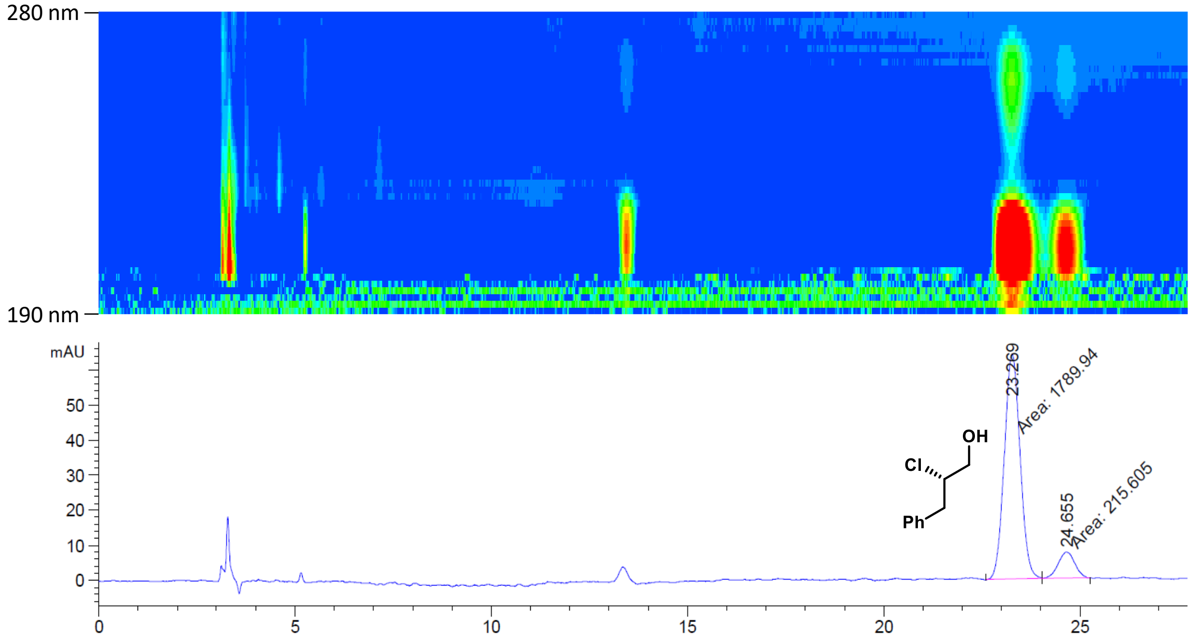

Peak #	RetTime [min]	Type	Width [min]	Area [mAU*s]	Height [mAU]	Area %
1	23.269	MM	0.4651	1789.93738	64.14368	89.2496
2	24.655	MM	0.4838	215.60464	7.42792	10.7504

Chiralpak OD-H, 4.6 x 250 mm, 208 nm detection, 1 mL/min Hexane:IPA 97:3

Standard reaction conditions (Section 10.1.)

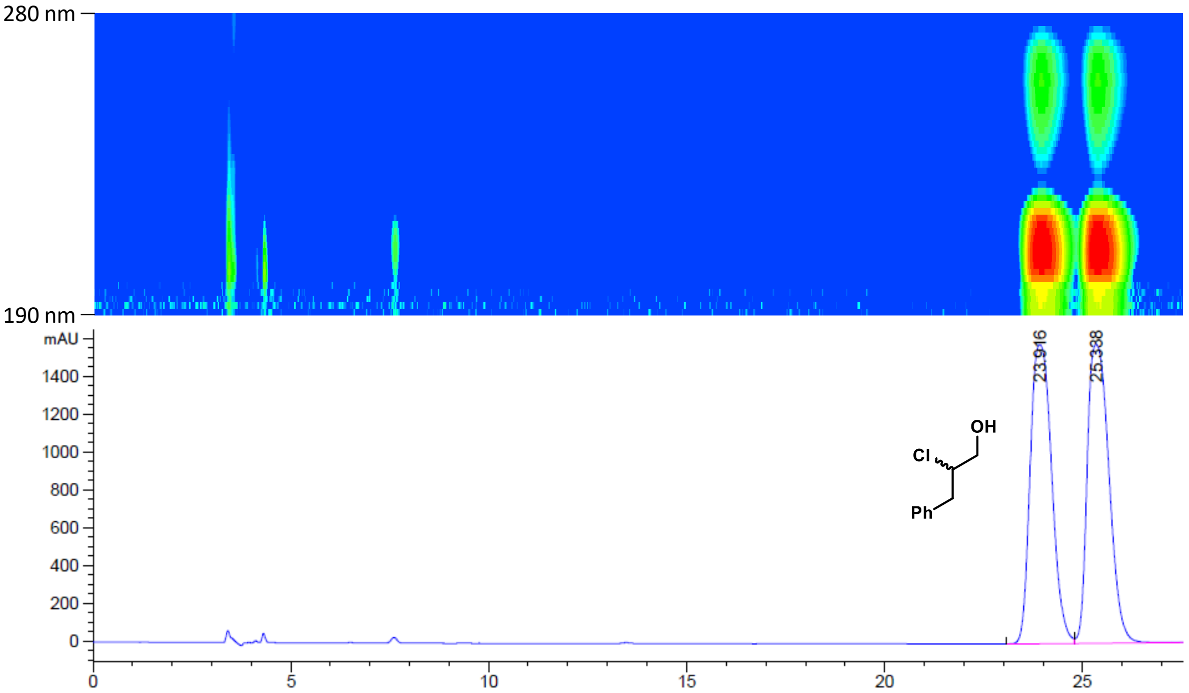

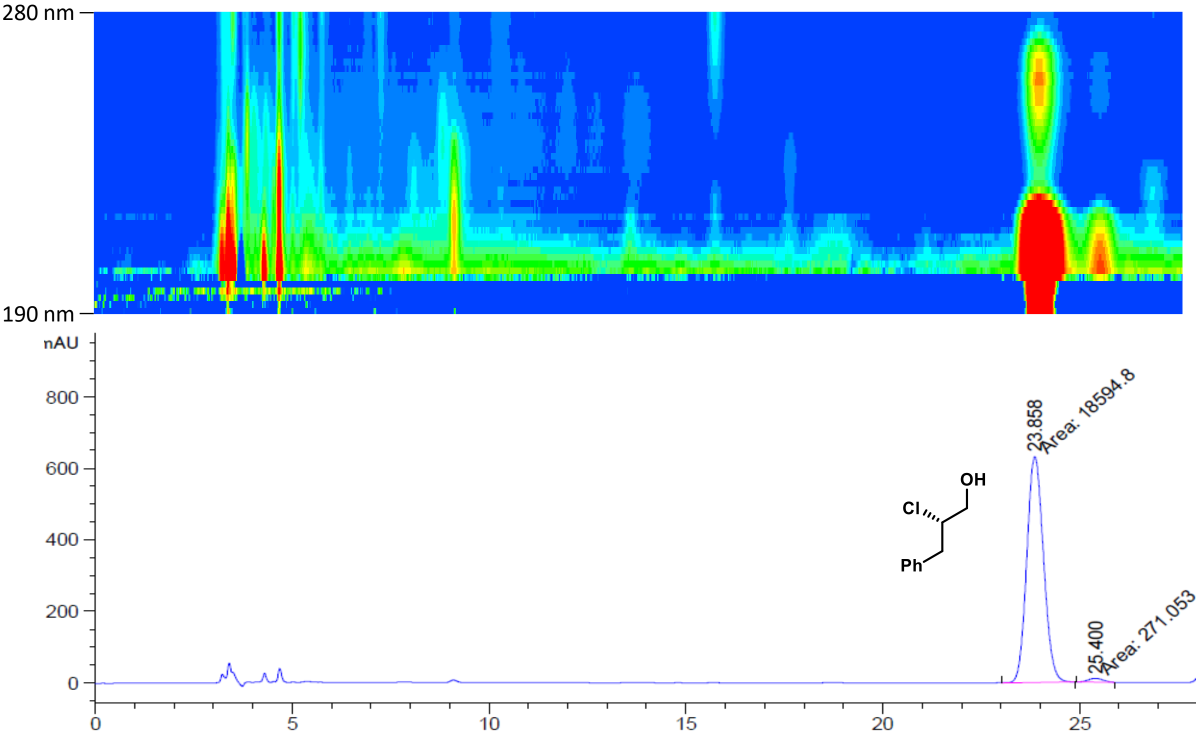

Peak #	RetTime [min]	Type	Width [min]	Area [mAU*s]	Height [mAU]	Area %
1	23.858	MM	0.4914	1.85948e4	630.71539	98.5633
2	25.400	MM	0.4593	271.05289	9.83492	1.4367

Chiralpak OD-H, 4.6 x 250 mm, 208 nm detection, 1 mL/min Hexane:IPA 97:3

Reaction with NCS as the chlorinating agent (Section 10.2.)

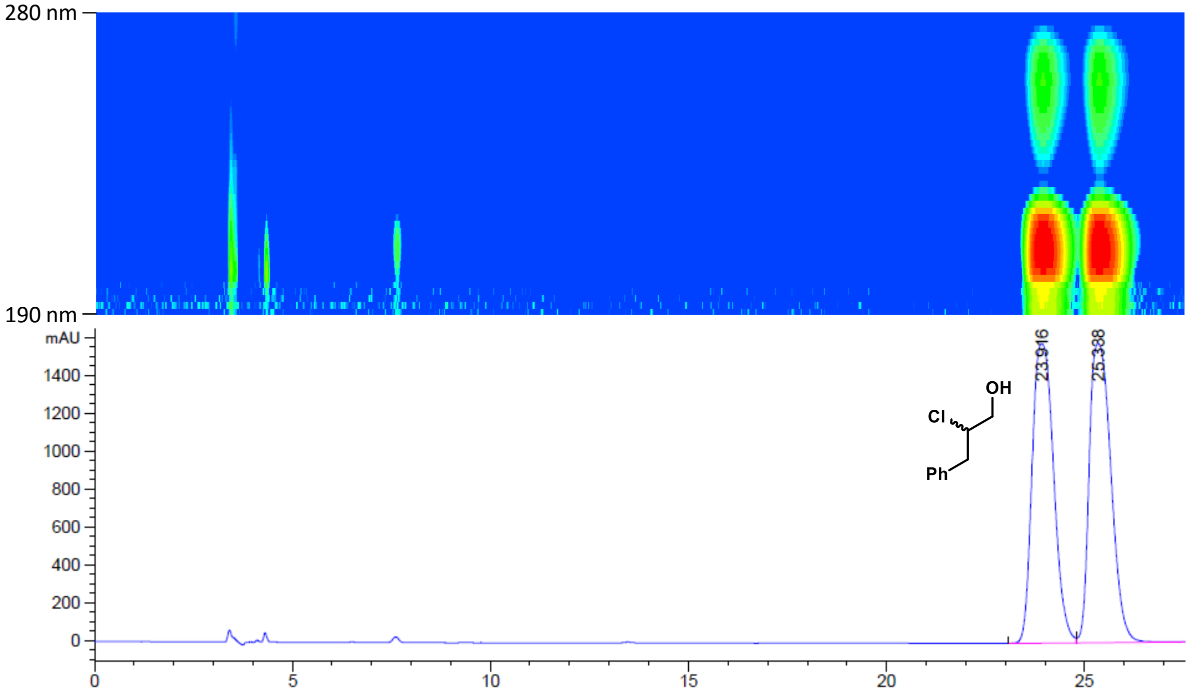

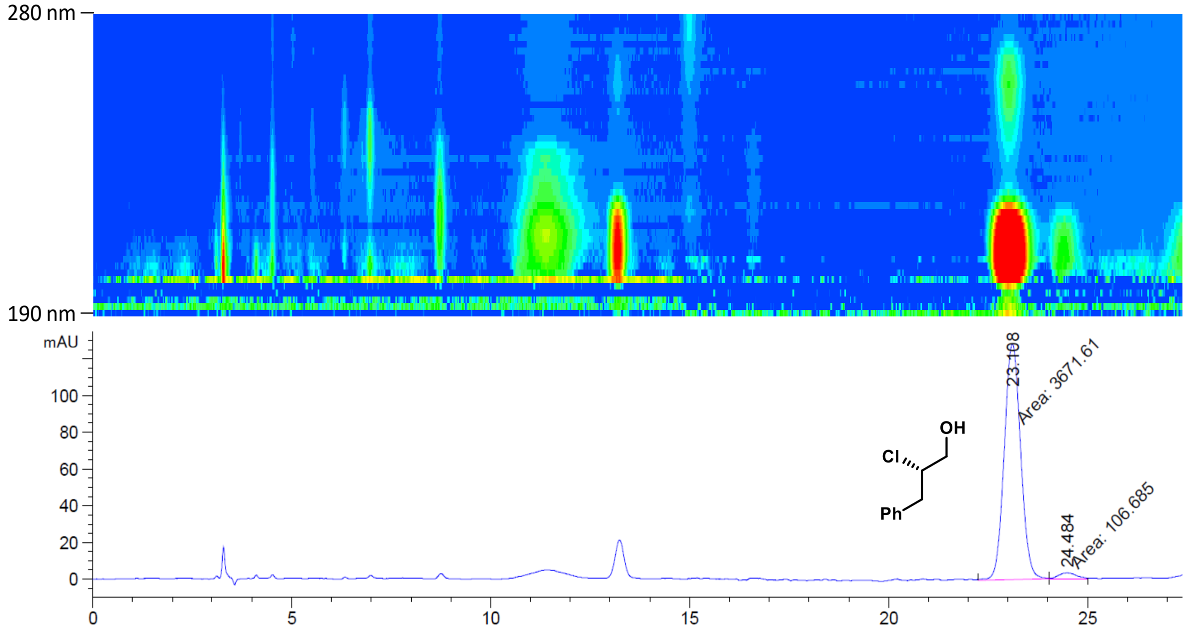

Peak #	RetTime [min]	Type	Width [min]	Area [mAU*s]	Height [mAU]	Area %
1	23.108	MM	0.4766	3671.61182	128.38962	97.1764
2	24.484	MM	0.5255	106.68460	3.38351	2.8236

Chiralpak OD-H, 4.6 x 250 mm, 208 nm detection, 1 mL/min Hexane:IPA 97:3

With catalyst **3a** (Section 10.3.)

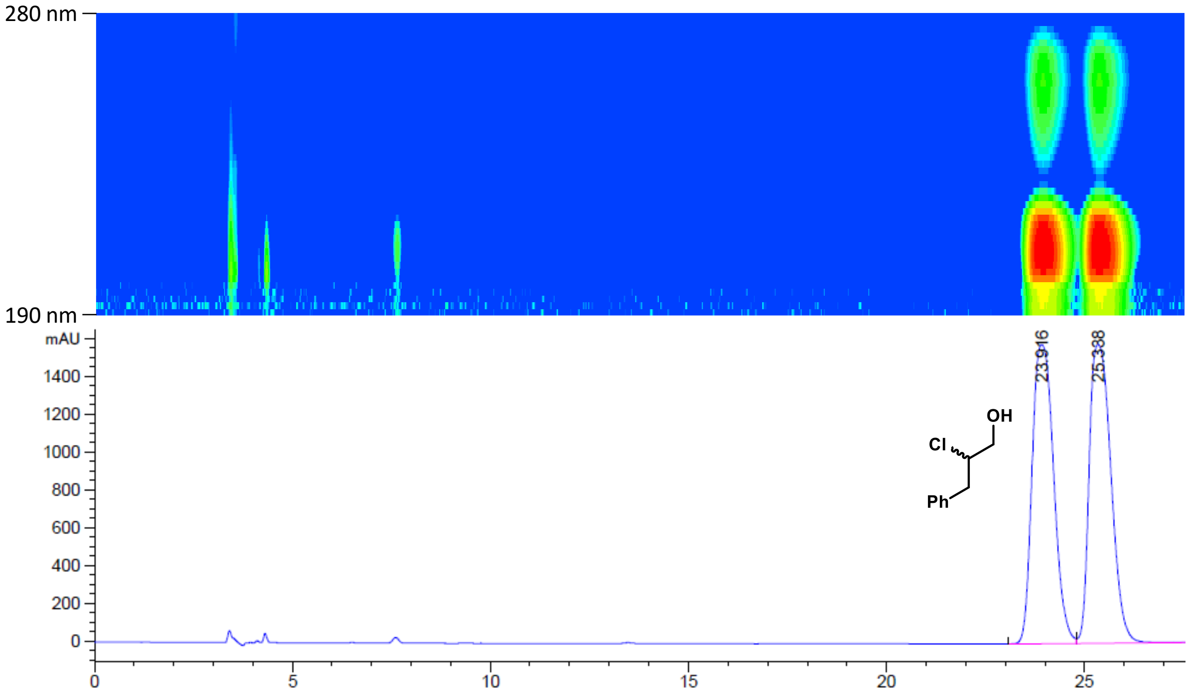

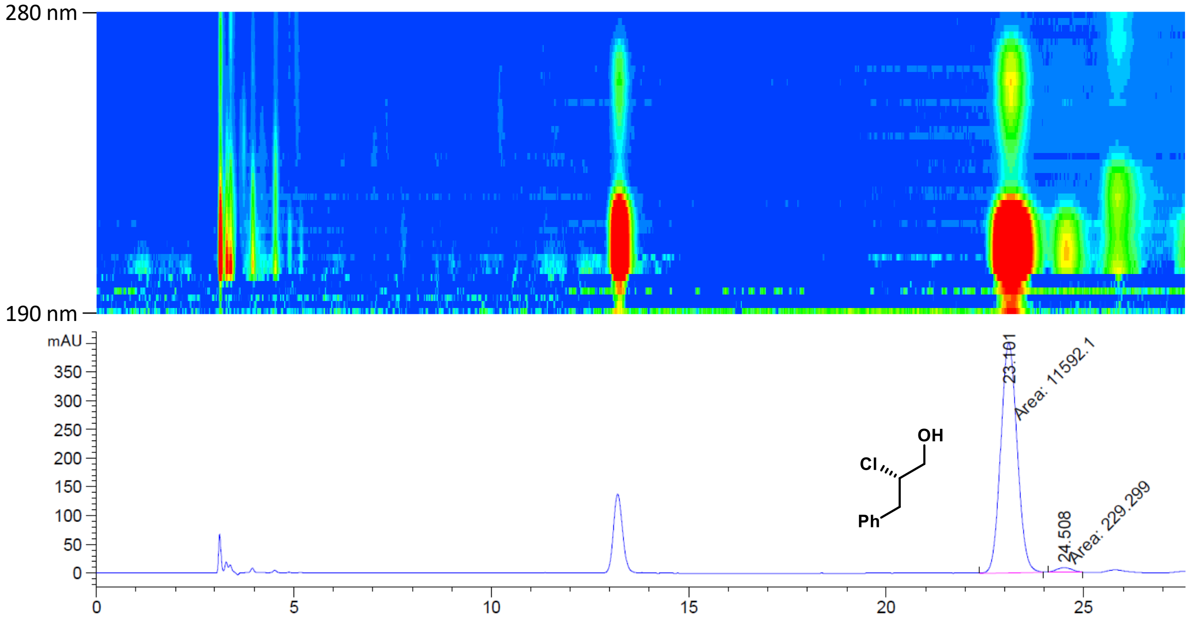

Peak #	RetTime [min]	Type	Width [min]	Area [mAU*s]	Height [mAU]	Area %
1	23.101	MM	0.4826	1.15921e4	400.32486	98.0603
2	24.508	MM	0.4663	229.29875	8.19482	1.9397

Chiralpak OD-H, 4.6 x 250 mm, 208 nm detection, 1 mL/min Hexane:IPA 97:3

Reaction with 1 mol% catalyst loading (Section 10.4.)

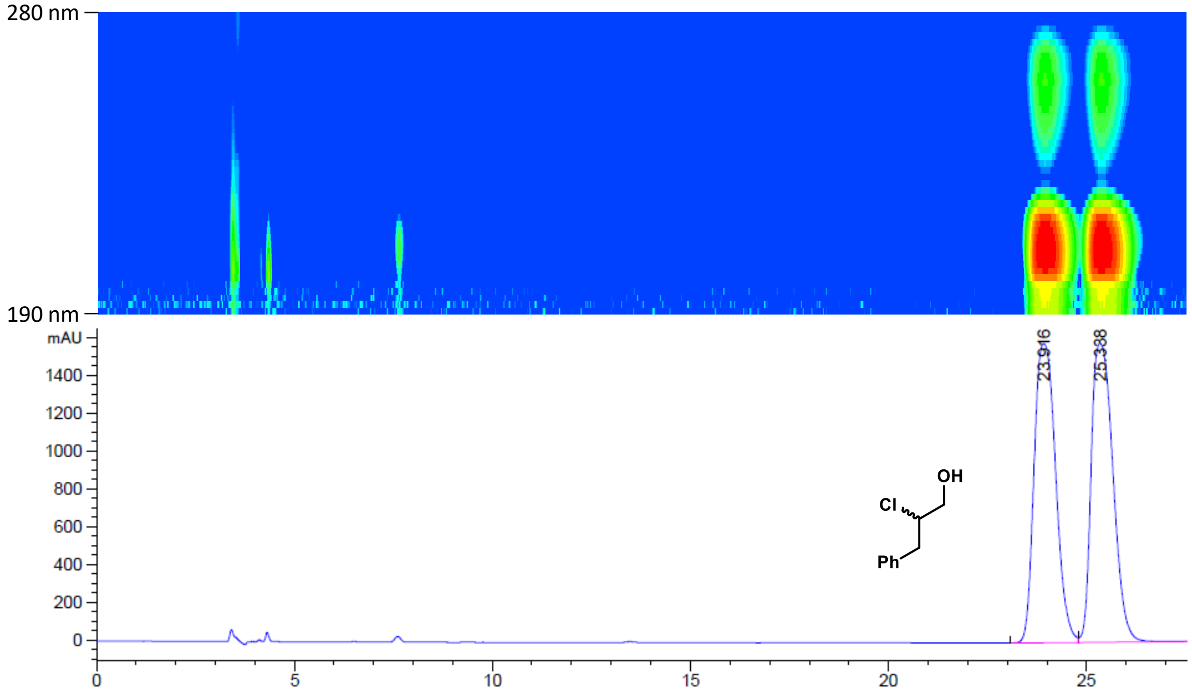

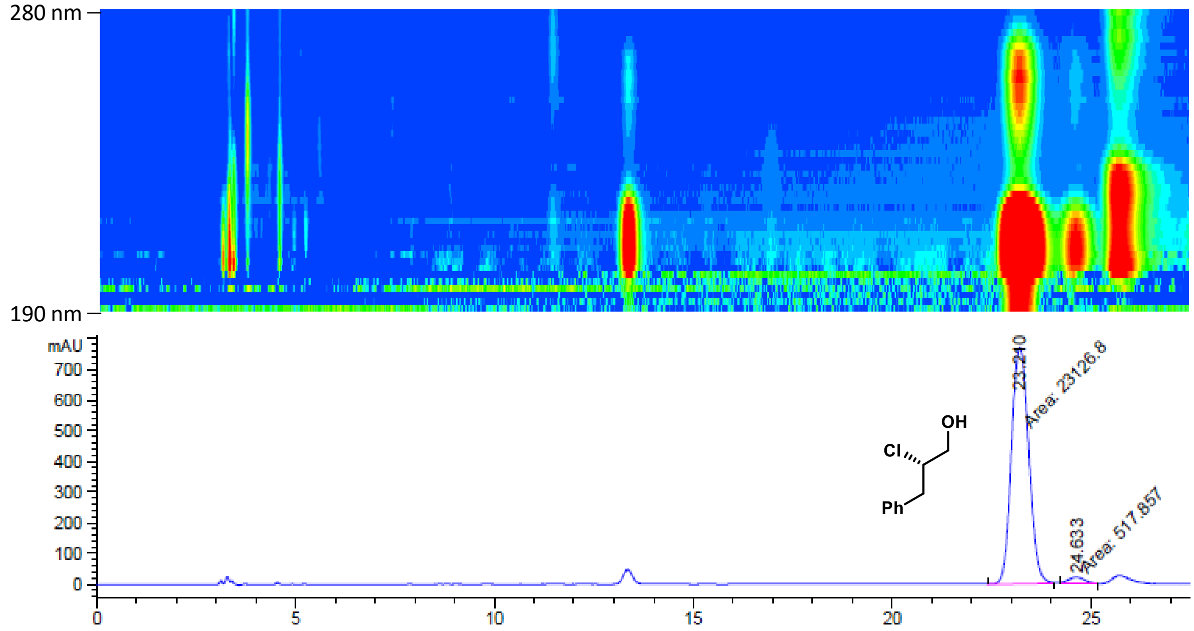

Peak #	RetTime [min]	Type	Width [min]	Area [mAU*s]	Height [mAU]	Area %
1	23.210	MM	0.5008	2.31268e4	769.71350	97.8098
2	24.633	MM	0.4515	517.85651	19.11471	2.1902

Chiralpak OD-H, 4.6 x 250 mm, 208 nm detection, 1 mL/min Hexane:IPA 97:3

Room temperature reaction (Section 10.5.)

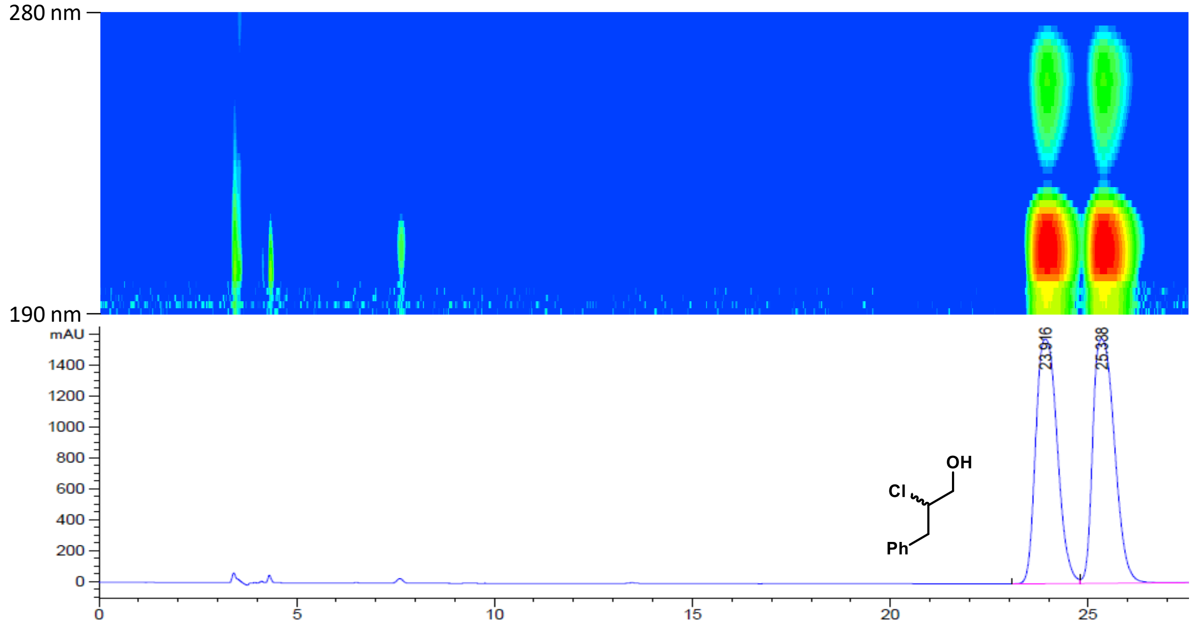

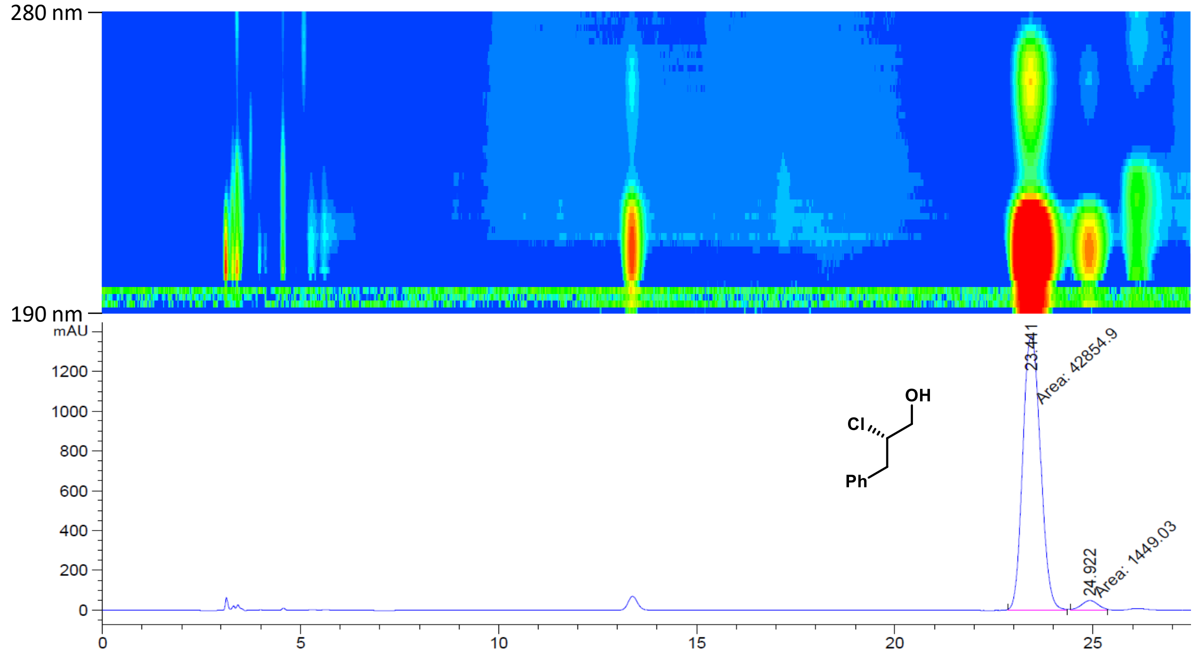

Peak #	RetTime [min]	Type	Width [min]	Area [mAU*s]	Height [mAU]	Area %
1	23.441	MM	0.5188	4.28549e4	1376.72119	96.7294
2	24.922	MM	0.4933	1449.02588	48.95361	3.2706

Chiralpak OD-H, 4.6 x 250 mm, 208 nm detection, 1 mL/min Hexane:IPA 97:3

Reaction with 1.0 equivalent of hydrocinnamaldehyde (Section 10.6.)

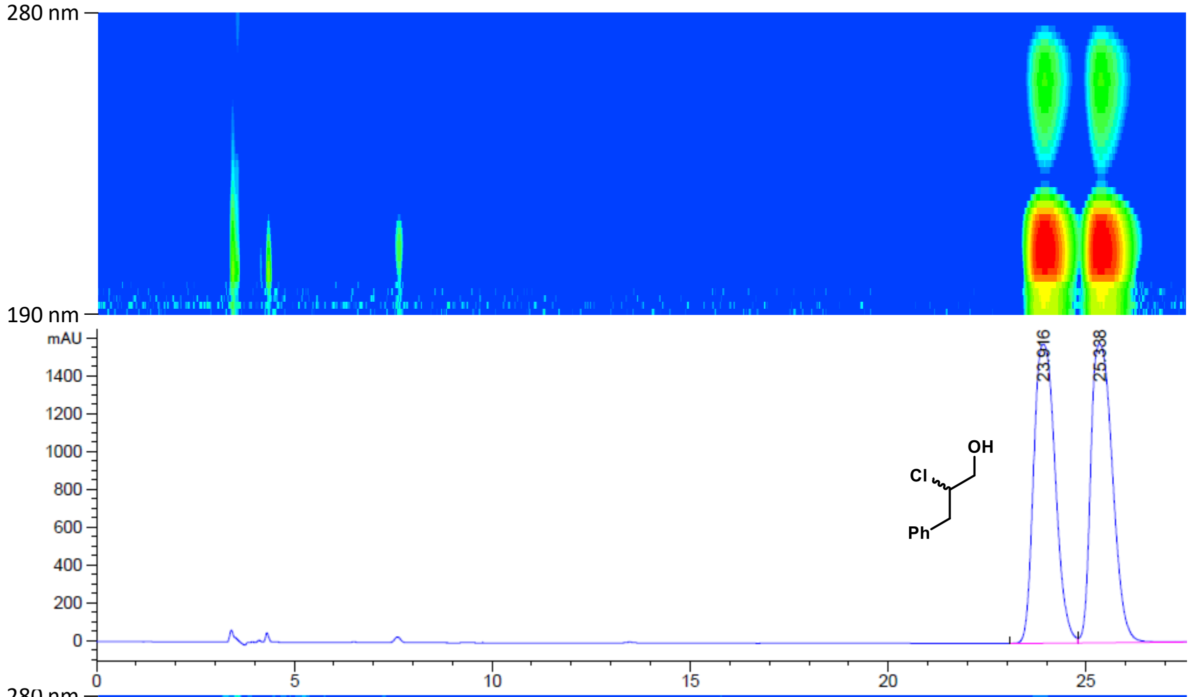

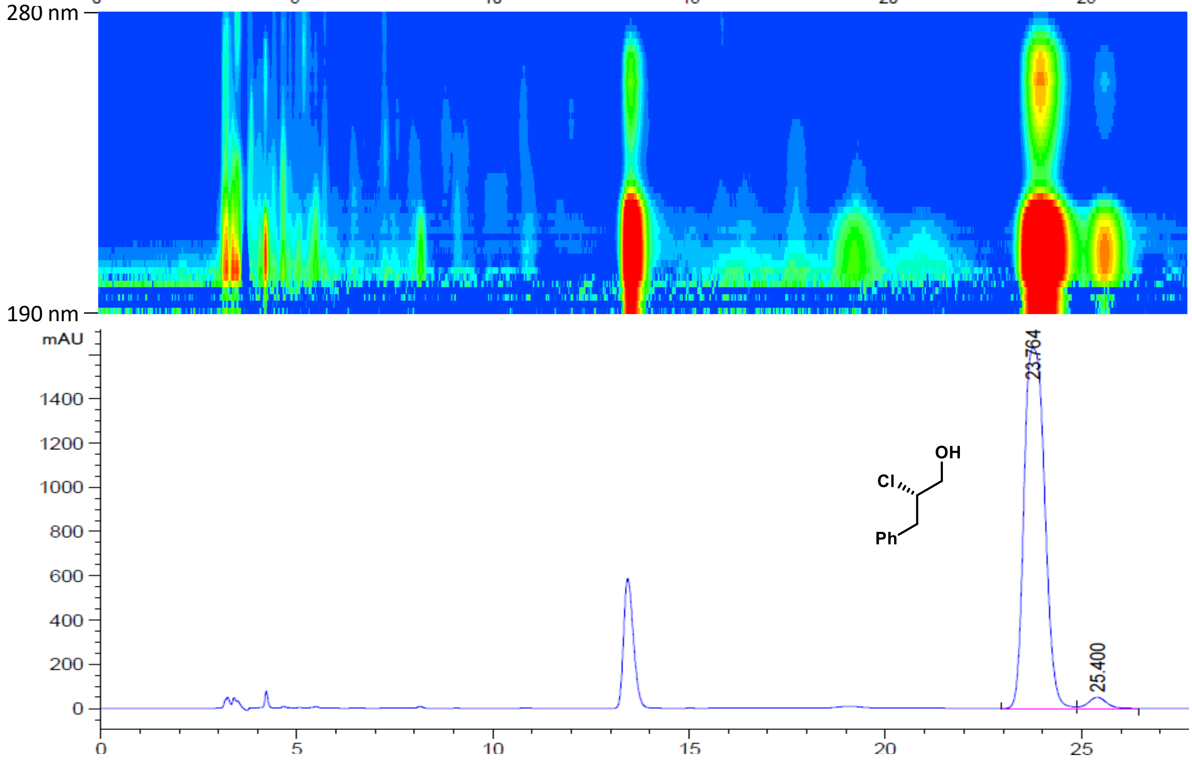

Peak #	RetTime [min]	Type	Width [min]	Area [mAU*s]	Height [mAU]	Area %
1	23.764	BV	0.5802	5.93014e4	1631.04102	97.3426
2	25.400	VB	0.4896	1618.89392	50.02361	2.6574

Chiralpak OD-H, 4.6 x 250 mm, 208 nm detection, 1 mL/min Hexane:IPA 97:3

Octanal (Section 10.7.1.)

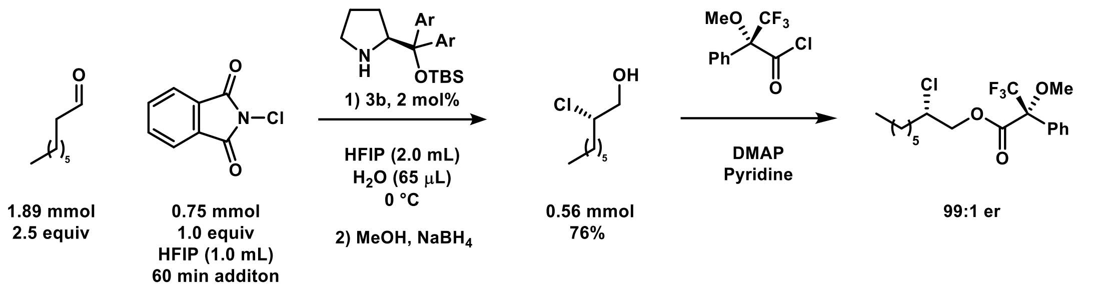

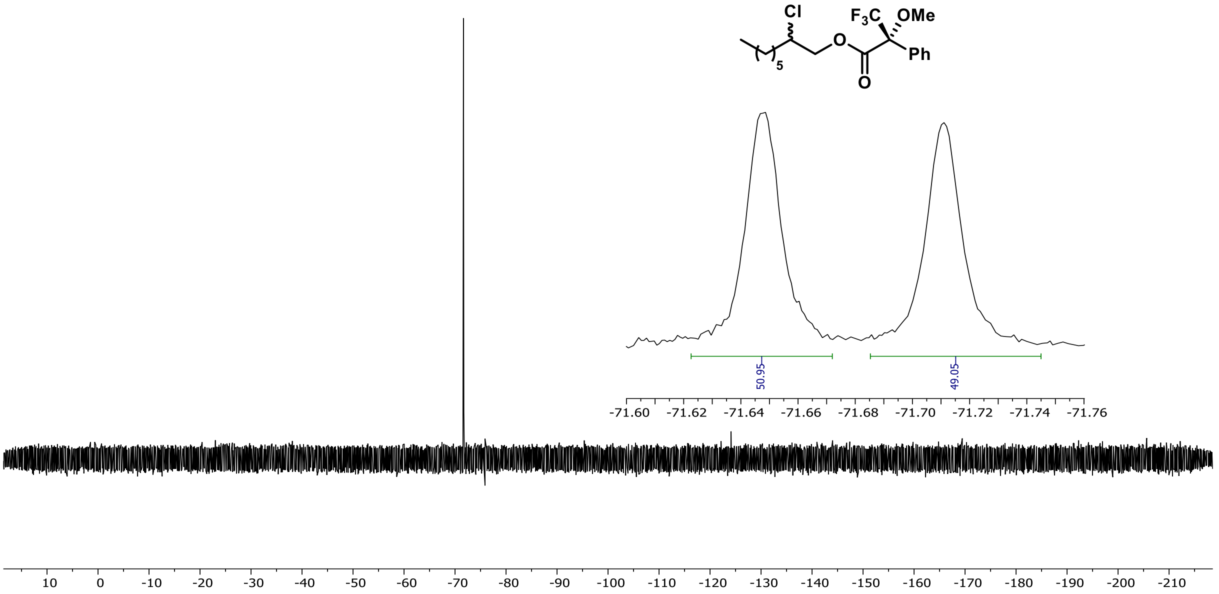

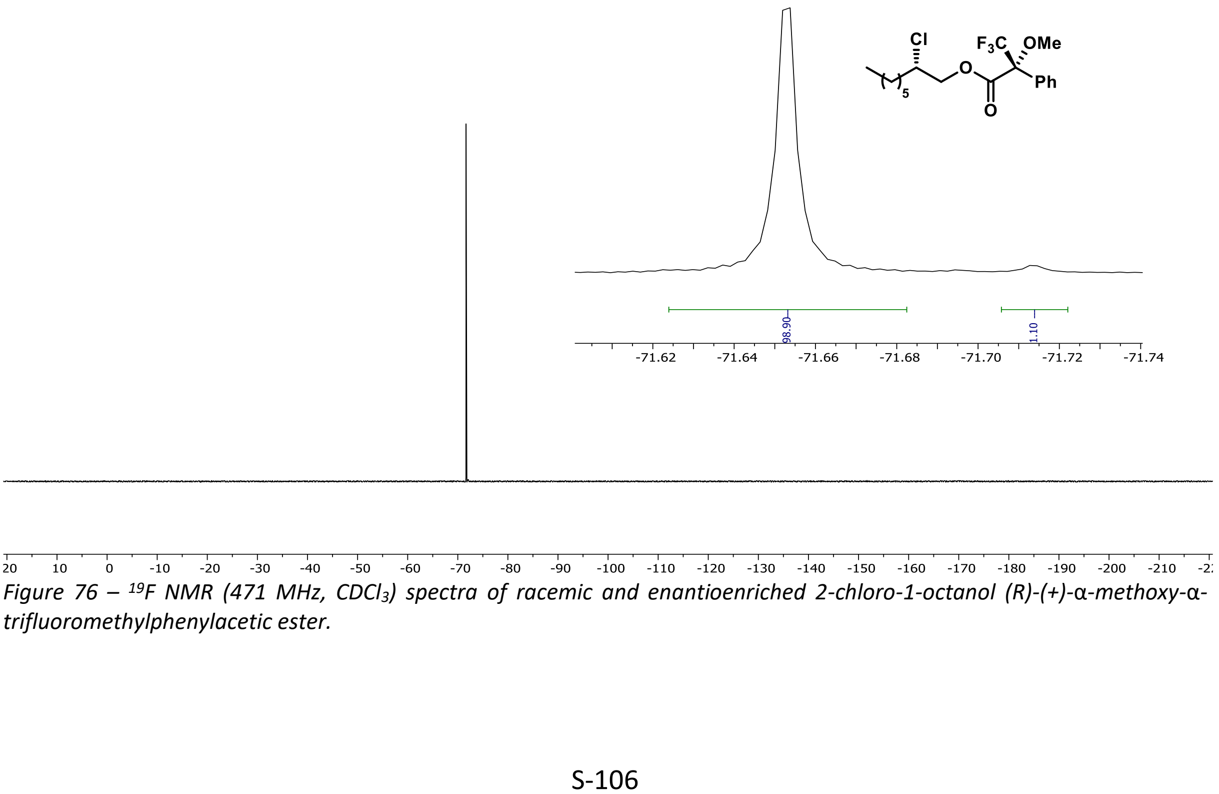

Figure 76 – ^{19}F NMR (471 MHz, $CDCl_3$) spectra of racemic and enantioenriched 2-chloro-1-octanol (R)-(+)-α-methoxy-α-trifluoromethylphenylacetic ester.

Isovaleraldehyde (Section 10.7.2.)

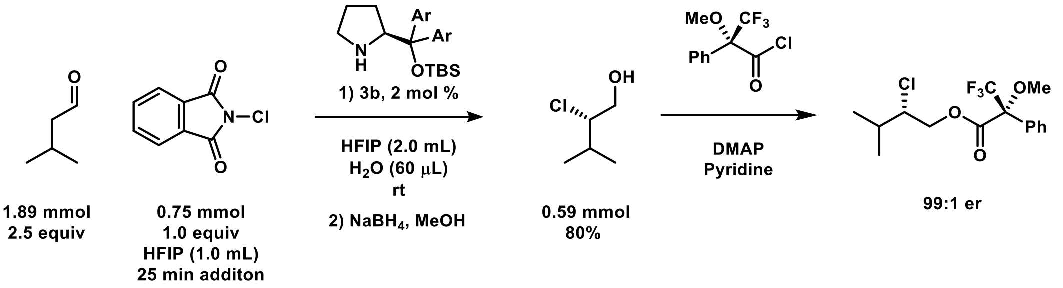

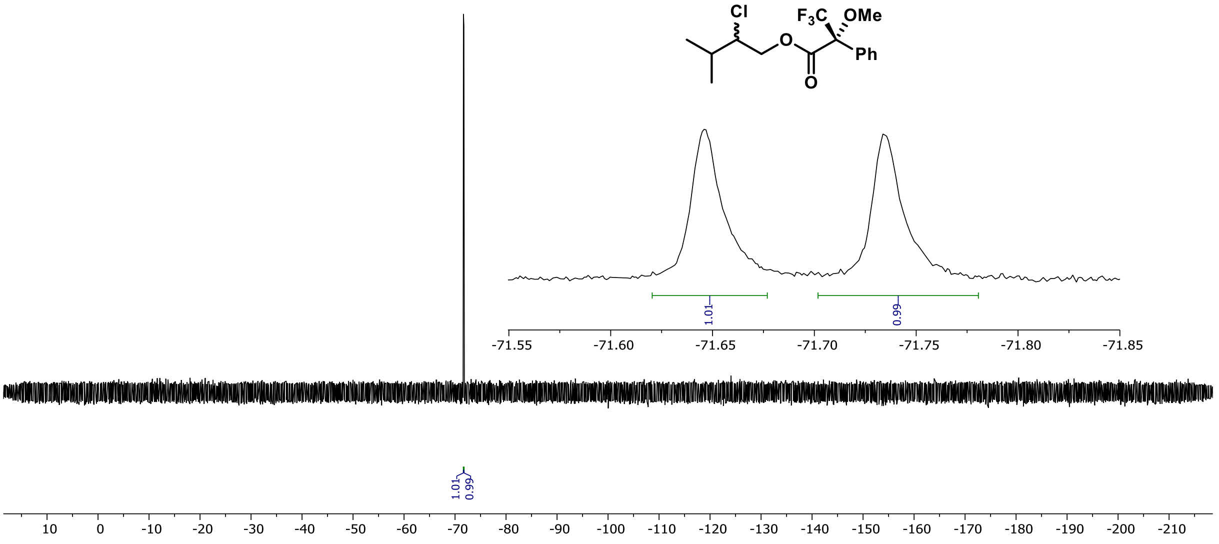

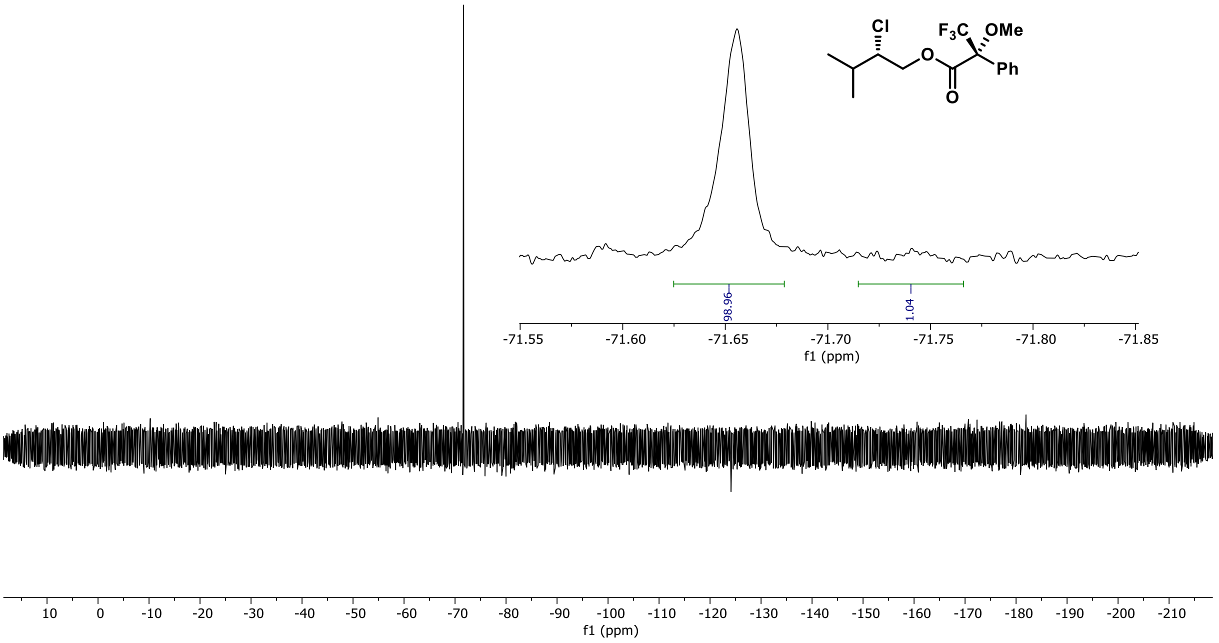

Figure 77 - ^{19}F NMR (471 MHz, $CDCl_3$) spectra of racemic and enantioenriched 2-chloro-3-methylbutan-1-ol (R)-(+)-α-methoxy-α-trifluoromethylphenylacetic ester.

Propanal (Section 10.7.3.)

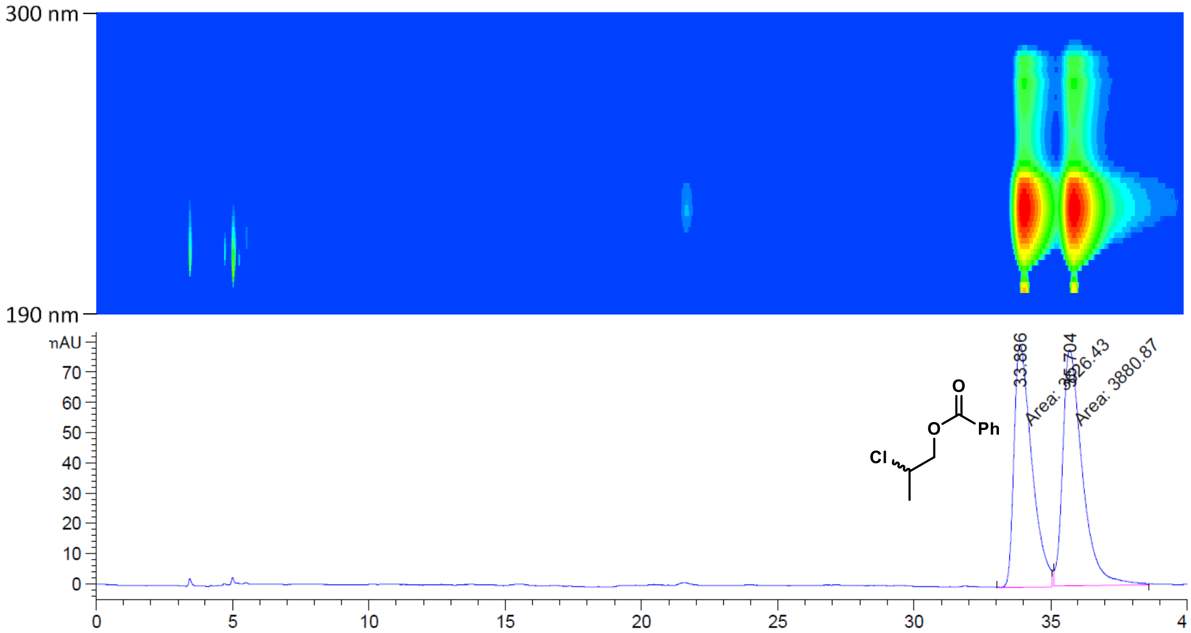

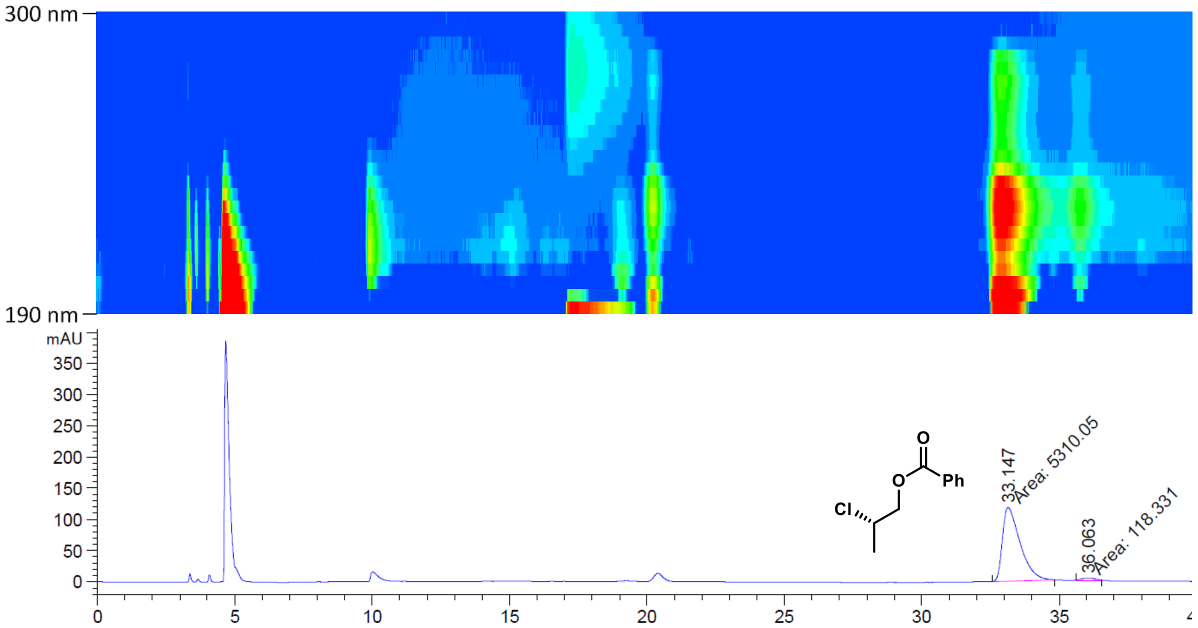

Peak #	RetTime [min]	Type	Width [min]	Area [mAU*s]	Height [mAU]	Area %
1	33.147	MM	0.7483	5310.04980	118.27438	97.8201
2	36.063	MM	0.5549	118.33125	3.55415	2.1799

Chiralpak OD-H, 4.6 x 250 mm, 220 nm detection, 1 mL/min Hexane:IPA 99:1

δ-Valerolactol (Section 10.7.4.)

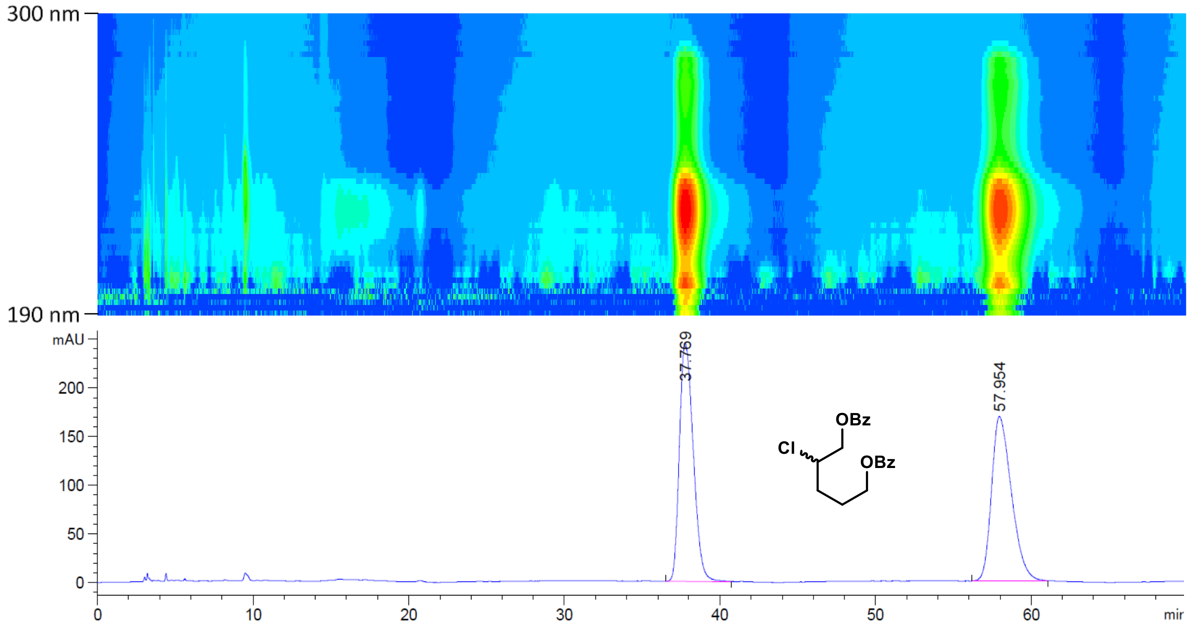

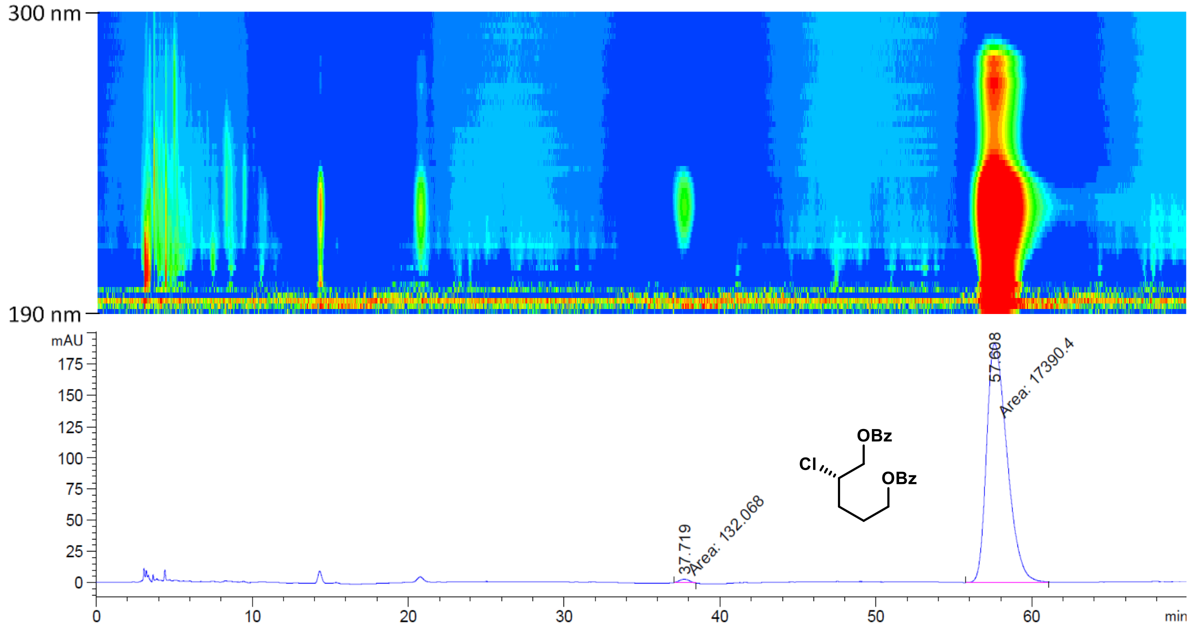

Peak #	RetTime [min]	Type	Width [min]	Area [mAU*s]	Height [mAU]	Area %
1	37.719	MM	0.7766	132.06825	2.83418	0.7537
2	57.608	MM	1.5163	1.73904e4	191.15370	99.2463

Chiralpak OD-H, 4.6 x 250 mm, 220 nm detection, 1 mL/min Hexane:IPA 98:2

Pentanal (Section 10.7.5.)

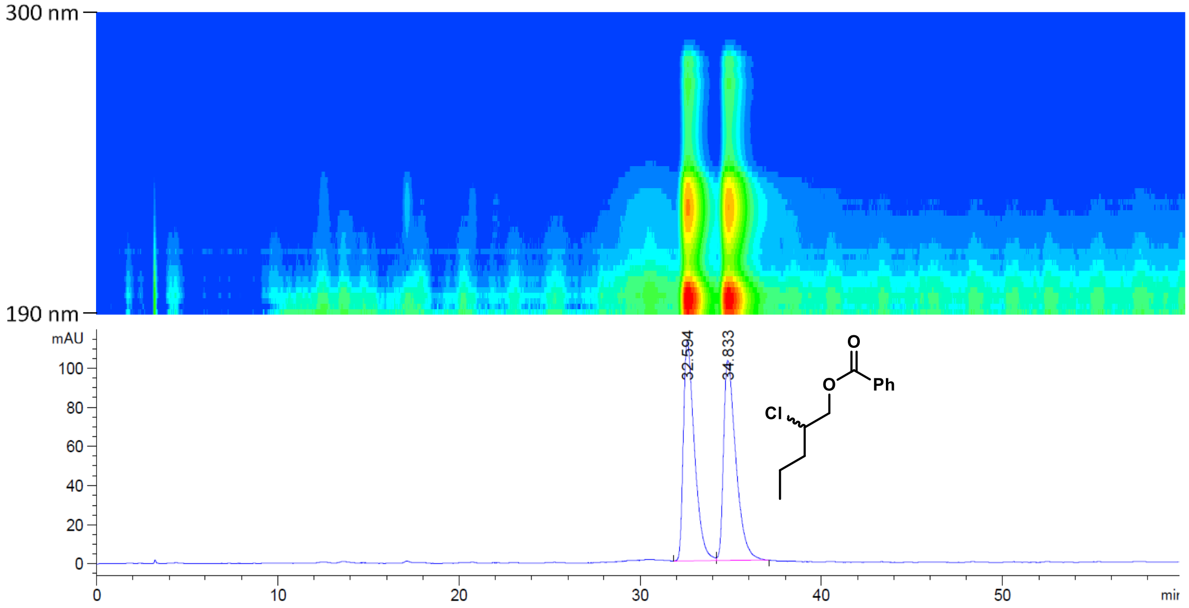

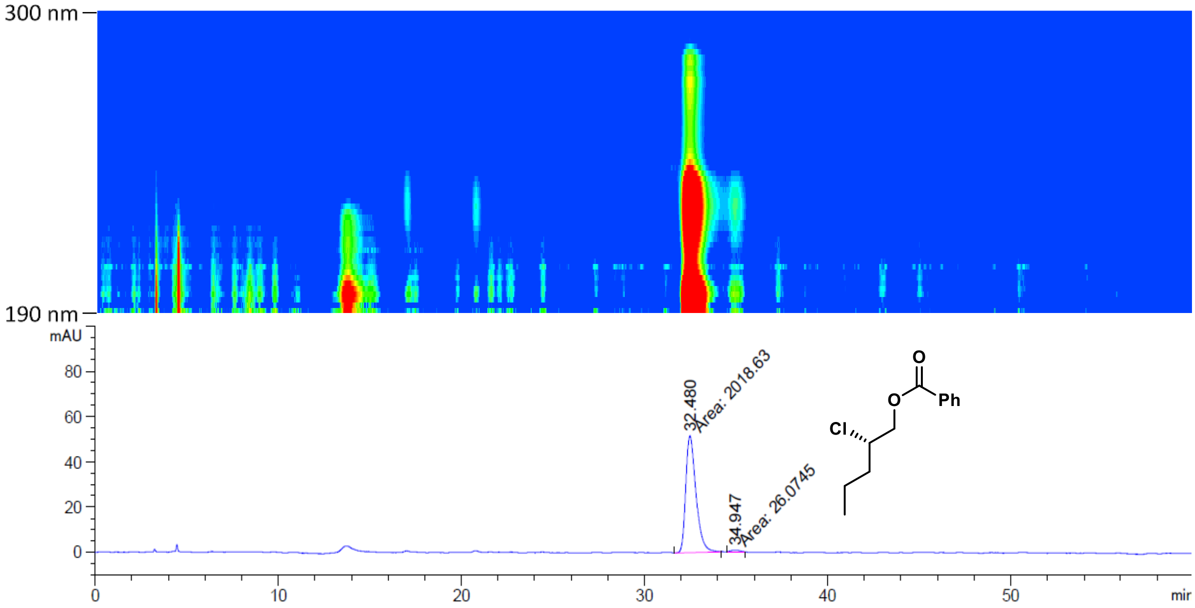

Peak #	RetTime [min]	Type	Width [min]	Area [mAU*s]	Height [mAU]	Area %
1	32.480	MM	0.6512	2018.63428	51.66745	98.7248
2	34.947	MM	0.5881	26.07453	7.38961e-1	1.2752

Chiralpak OD-H, 4.6 x 250 mm, 220 nm detection, 1 mL/min Hexane:IPA 99:1

5-Bromopentanal (Section 10.7.6.)

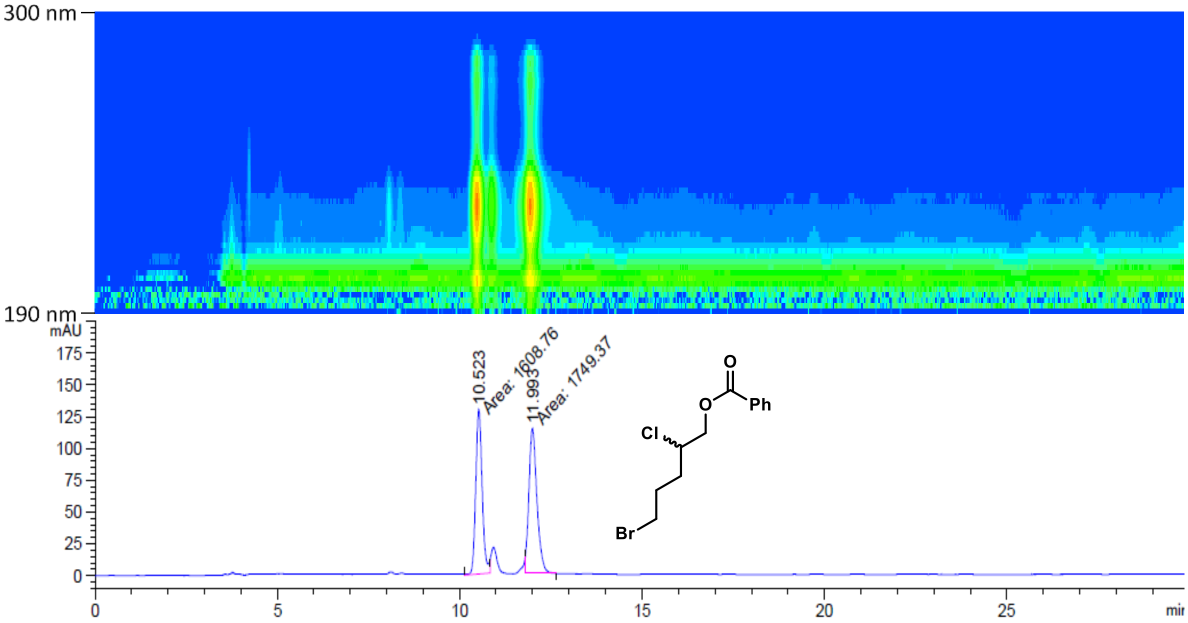

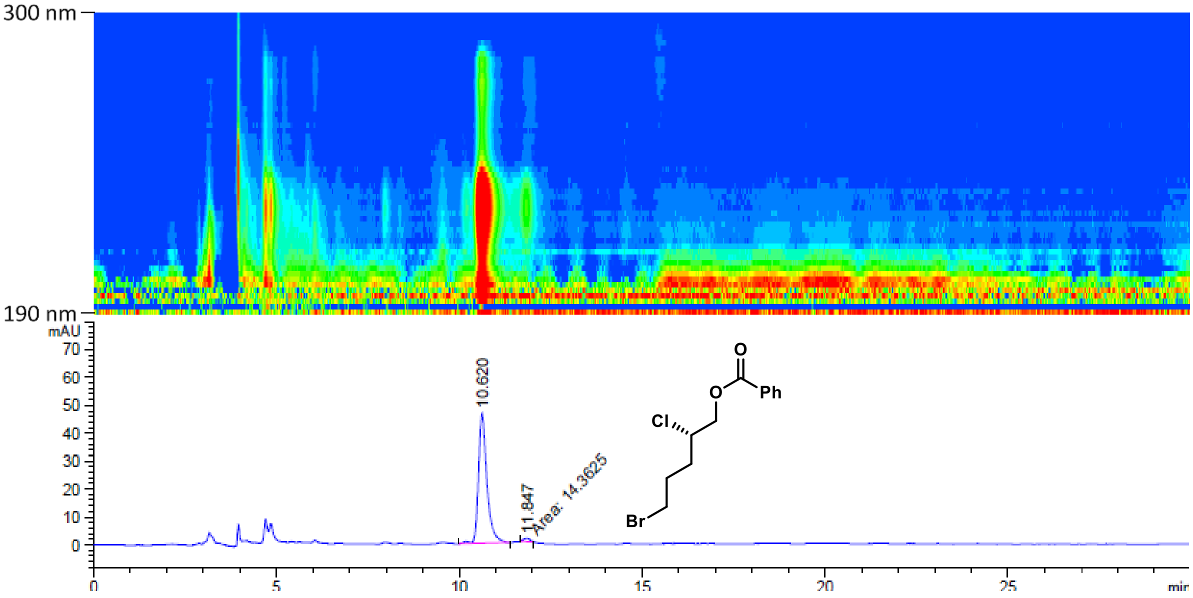

Peak #	RetTime [min]	Type	Width [min]	Area [mAU*s]	Height [mAU]	Area %
1	10.620	BB	0.2440	759.66650	46.64872	98.1444
2	11.847	MM	0.1954	14.36253	1.22529	1.8556

Chiralpak OD-H, 4.6 x 250 mm, 220 nm detection, 1 mL/min Hexane:IPA 98:2

Dodecanal (Section 10.7.7.)

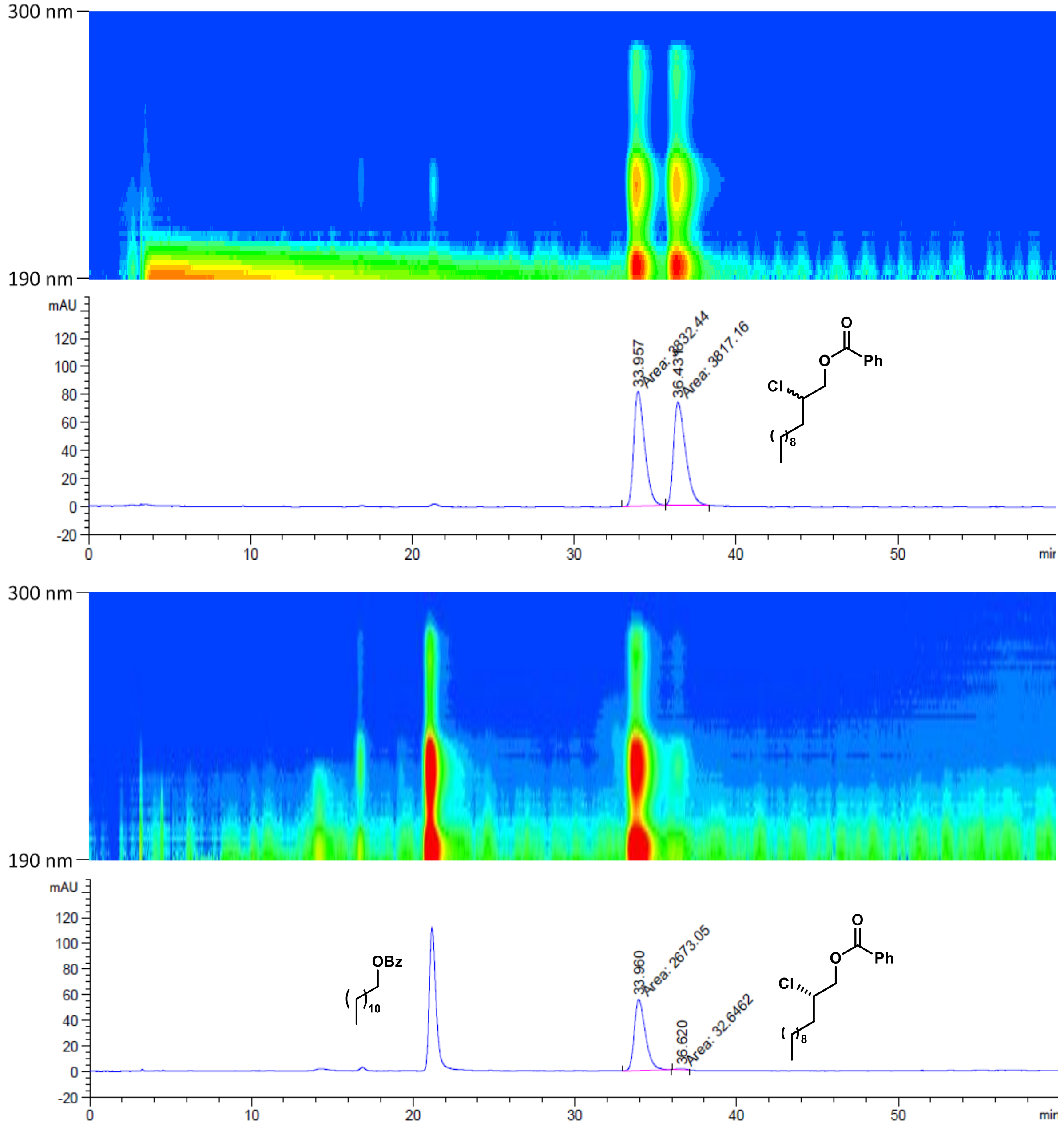

Peak #	RetTime [min]	Type	Width [min]	Area [mAU*s]	Height [mAU]	Area %
1	33.960	MM	0.8008	2673.04810	55.63396	98.7934
2	36.620	MM	0.6539	32.64624	8.32099e-1	1.2066

Chiralpak OD-H, 4.6 x 250 mm, 220 nm detection, 1 mL/min Hexane:IPA 99:1

15. References

1. *(a)* Burés, J.; Dingwall, P.; Armstrong, A.; Blackmond, D. *Angew. Chem. Int. Ed.*, **2014**, *53*, 8700–8704. *(b)* Burés, J.; Armstrong, A.; Blackmond, D. *Acc. Chem. Res.*, **2016**, *49*, 214–222. *(c)* Duschmal, J.; Wiest, J.; Wiesner, M.; Wennemers, H. *Chem. Sci.*, **2013**, *4*, 1312. *(d)* Seebach, D.; Sun, X.; Ebert, M.-O.; Bchweizer, B.; Purkayastha, N.; Beck, A. K.; Duschmale, J.; Wennemers, D.; Mukaiyama, T.; Benohoud, M.; Hayashi, Y.; Reiher, M. *Helv. Chim. Acta*, **2013**, *96*, 799–852. *(e)* Földes, T.; Madarász, A.; Révész, A.; Dobi, Z.; Varga, S.; Hamza, A.; Nagy, P. R.; Pihko, P. M.; Pápai, I. *J. Am. Chem. Soc.,* **2017**, *139*, 17052–17063. *(f)* Erkkilä, A.; Majander, I.; Pihko, P. M. *Chem. Rev.*, **2007**, *107*, 5416–5470.

2. Brochu, M. P.; Brown, S. P.; MacMillan, D. W. C. *J. Am. Chem. Soc.,* **2004**, *126*, 4108–4109.

3. Halland, N.; Braunton, A.; Bachmann, S.; Marigo, M.; Jørgensen, K. A. *J. Am. Chem. Soc.,* **2004**, *126*, 4790−4791.

5. Amatore, M.; Beeson, T. D.; Brown, S. P.; MacMillan, D. W. C. *Angew. Chem. Int. Ed.,* **2009**, *48*, 5121−5124.

6. Jimeno, C.; Cao, L.; Renaud, P. *J. Org. Chem.,* **2016**, *81*, 1251−1255.

7. Ponath, S.; Menger, M.; Grothues, L.; Weber, M.; Lentz, D.; Strohmann, C.; Christmann, M. *Angew. Chem., Int. Ed.,* **2018**, *57*, 11683–11687.

8. Seifert, F.-U.; Röschenthaler, G.-V. *J. Fluor. Chem*., **1994**, *68*, 169–174.

9. Hoops, S.; Sahle, S.; Gauges, R.; Lee, C.; Pahle, J.; Simus, N.; Singhal, M.; Xu, L.; Mendes, P.; Kummer, U. *Bioinformatics,* **2006**, *22*, 3067–3074.

10. Ninomiya, M.; Ando, Y.; Kudo, F.; Ohmori, K.; Suzuki, K. *Angew. Chem. Int. Ed.,* **2019**, *58*, 4264–4270.

11. a) Martínez-Carrión, A.; Howlett, M. G.; Alamillo-Ferrer, C.; Clayton, A. D.; Bourne, R. A.; Codina, A.; Vidal-Ferran, A.; Adams, R. W.; Burés, J. *Angew. Chem. Int. Ed.,* **2019**, *58*, 10189–10193. b) Alamillo-Ferrer, C.; Nielsen, C, D.-T.; Salzano, A.; Companyó, X.; Di Sanza, R.; Spivey, A. C.; Rzepa, H. S.; Burés, J. *J. Org. Chem.*, **2021**, *86*, 4326–4335.

12. Glass, B. D.; Goosen, A.; McCleland, C. W. *J. Chem. Soc., Perkin Trans.,* **1993**, *2*, 2175-2181.

13. Barbosa, T. M.; Viesser, R.; Abraham, R.; Rittner, R.; Tormena, C. *RSC Adv*., **2015**, *5*, 35412–35420.

14. Plazinska, A.; Pajak, K.; Rutkowska, E.; Jimenez, L.; Kozocas, J.; Koolpe, G.; Tanga, M.; Toll, L.; Wainer, I. W.; Jozwiak, K. *Bioorganic & Medicinal Chemistry*, **2014**, *22*, 234–246.

15. Garrett, C. E.; Fu, G. C. *J. Org. Chem.,* **1997**, *62*, 4534–4535.

16. Hutchinson, G.; Welsh, C. D. M.; Burés, J. *J. Org. Chem.* **2021**, *86*, 2012–2016.

17. De Buyck, L.; Verhé, R.; De Kimpe, N.; Courtheyn, D.; Schamp, N. *Bull. Soc. Chim. Belg.,* **1980**, *89*, 441–458
